# Coinage metal aluminyl complexes: probing regiochemistry and mechanism in the insertion and reduction of carbon dioxide†

**DOI:** 10.1039/d1sc04676d

**Published:** 2021-09-16

**Authors:** Caitilín McManus, Jamie Hicks, Xianlu Cui, Lili Zhao, Gernot Frenking, Jose M. Goicoechea, Simon Aldridge

**Affiliations:** Inorganic Chemistry Laboratory, Department of Chemistry, University of Oxford South Parks Road Oxford OX1 3QR UK simon.aldridge@chem.ox.ac.uk; Institute of Advanced Synthesis, School of Chemistry and Molecular Engineering, Jiangsu National Synergetic Innovation Center for Advanced Materials, Nanjing Tech University Nanjing 211816 P. R. China; Fachbereich Chemie, Philipps-Universität, Marburg D-35043 Marburg Germany

## Abstract

The synthesis of coinage metal aluminyl complexes, featuring M–Al covalent bonds, is reported *via* a salt metathesis approach employing an anionic Al(i) (‘aluminyl’) nucleophile and group 11 electrophiles. This approach allows access to both bimetallic (1 : 1) systems of the type (^*t*^Bu_3_P)MAl(NON) (M = Cu, Ag, Au; NON = 4,5-bis(2,6-diisopropylanilido)-2,7-di-*tert*-butyl-9,9-dimethylxanthene) and a 2 : 1 di(aluminyl)cuprate system, K[Cu{Al(NON)}_2_]. The bimetallic complexes readily insert heteroallenes (CO_2_, carbodiimides) into the unsupported M–Al bonds to give systems containing a M(CE_2_)Al bridging unit (E = O, NR), with the μ-κ^1^(C):κ^2^(E,E′) mode of heteroallene binding being demonstrated crystallographically for carbodiimide insertion in the cases of all three metals, Cu, Ag and Au. The regiochemistry of these processes, leading to the formation of M–C bonds, is rationalized computationally, and is consistent with addition of CO_2_ across the M–Al covalent bond with the group 11 metal acting as the nucleophilic partner and Al as the electrophile. While the products of carbodiimide insertion are stable to further reaction, their CO_2_ analogues have the potential to react further, depending on the identity of the group 11 metal. (^*t*^Bu_3_P)Au(CO)_2_Al(NON) is inert to further reaction, but its silver counterpart reacts slowly with CO_2_ to give the corresponding carbonate complex (and CO), and the copper system proceeds rapidly to the carbonate even at low temperatures. Experimental and quantum chemical investigations of the mechanism of the CO_2_ to CO/carbonate transformation are consistent with rate-determining extrusion of CO from the initially-formed M(CO)_2_Al fragment to give a bimetallic oxide that rapidly assimilates a second molecule of CO_2_. The calculated energetic barriers for the most feasible CO extrusion step (Δ*G*^‡^ = 26.6, 33.1, 44.5 kcal mol^−1^ for M = Cu, Ag and Au, respectively) are consistent not only with the observed experimental labilities of the respective M(CO)_2_Al motifs, but also with the opposing trends in M–C (increasing) and M–O bond strengths (decreasing) on transitioning from Cu to Au.

## Abstract 2

The differential reactivity of copper, silver and gold aluminyl compounds towards CO_2_ and other heteroallenes are probed by experimental and quantum chemical methods.

## Graphical abstract

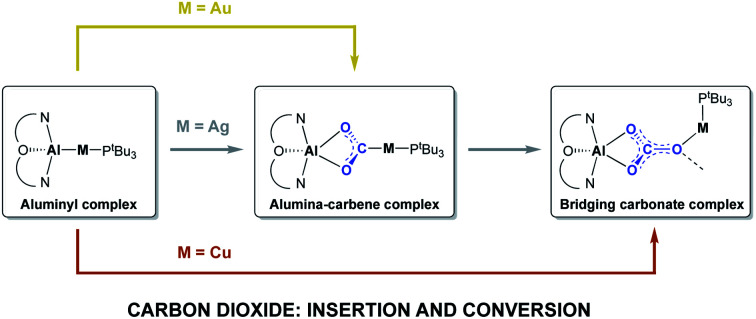

## Introduction

Combinations of metals – either in discrete complexes or in extended materials – have been shown to facilitate patterns of reactivity inaccessible to individual metals in isolation.^1^ Molecular heterobimetallic systems, for example, often possess reactivity distinct from complexes containing a single metal centre.^2^ Such attributes have been exploited to enable stoichiometric and catalytic transformations of kinetically challenging substrates, and to effect selectivity patterns distinct from monometallic systems.^3^ In the case of directly bonded bimetallics, the combination of two very different metals can lead to highly polarised bonds (and sites of differential Lewis acidity/basicity), which can produce synergistic effects in reactivity towards small molecules.^4^ Such systems also offer potential as single source precursors for metal alloys and functional materials.^5–8^

**Chart 1:**
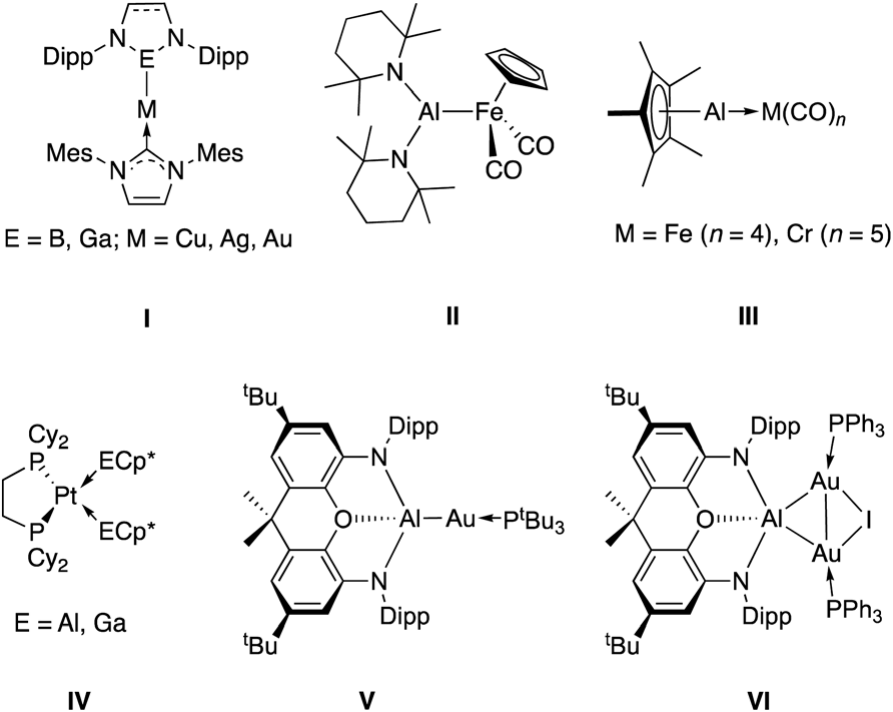
Selected examples of transition metal – group 13 complexes: coinage metal complexes made by metathesis using boryl or gallyl nucleophiles (**I**);^17,18^ an Fe–Al bond synthesized from [CpFe(CO)_2_]^−^ (**II**);^19^ transition metal complexes of AlCp* and GaCp* (**II** and **III**);^20,21^ previously communicated aluminium–gold complexes formed from an aluminyl nucleophile and Au(i) electrophiles (**V** and **VI**).^22^

Of particular interest are binuclear systems featuring elements from group 13 (Chart 1). The presence of a strongly Lewis acidic centre in close proximity to a d-block metal can assist in substrate activation, and has proven effective, for example, in mixed transition metal/aluminium systems for alkene polymerisation.^9^ In the case of directly bonded systems, the relative electropositivity of the group 13 elements typically means that ligands derived from them are very good σ-donors towards d-block metals.^10^ As such, the intermediacy of transition metal boryl complexes in metal catalysed C–H borylation chemistry, for example, owes much to the strong *trans* influence of this ligand class. This feature, combined with the availability of a formally vacant B-centred p-orbital, are key to both C–H bond breaking and subsequent C–B bond formation steps.^11,12^ More generally, the boryl ligand family has found extensive use across the Periodic Table with its strong donor properties facilitating a range of chemistries, including E–H activation^13^ and CO_2_ reduction.^14^ While a wide range of M–Ga bonded species have been synthesised using nucleophilic Ga(i) heterocycles,^15^ the chemistry of bimetallic aluminium-containing systems is less well studied.

A significant body of work has been carried out by Schnöckel and Fischer, using neutral Al(i) systems such as AlCp* to construct bonds typically to electron-rich transition metal carbonyl and olefin complexes.^16^ The resulting donor/acceptor M–Al bonds feature the AlCp* moiety acting as an L type ligand (isolobal with CO and PR_3_) and are relatively labile, rendering studies of their further chemistry challenging. As regards covalent M–Al bonds, a long-standing synthetic approach involves the metathesis reaction between an aluminium electrophile and a transition metal nucleophile, exemplified by the reaction between AlBr(tmp)_2_ and Na[CpFe(CO)_2_] to give CpFe(CO)_2_Al(tmp)_2_ reported by Nöth and co-workers.^19^ A shortcoming of this approach, however, is the relative scarcity of suitable electron-rich transition metal nucleophiles, which are typically limited to strongly pi accepting ancillary ligand sets (often carbonyls).

The recent development of nucleophilic aluminium ‘aluminyl’ anions allows access to a potential metathesis process in the opposite sense;^23^*i.e.* by the combination of a nucleophilic aluminium source with a readily available transition metal electrophile.^22,24,25^ We recently reported the synthesis of two aluminium–gold complexes (**V** and **VI**) prepared by salt metathesis between the potassium aluminyl complex, [K{Al(NON)}]_2_ (**1**, where NON = 4,5-bis(2,6-diiso-propylanilido)-2,7-di-*tert*-butyl-9,9-dimethyl-xanthene) and phosphine-supported Au(i) halides.^22^ These compounds represented the first examples of aluminium–gold covalent bonds, and bimetallic system **V** is particularly unusual in featuring a gold centre with nucleophilic character. **V** undergoes insertion of CO_2_ into the Al–Au bond to form the μ-κ^1^(C):κ^2^(O,O′) CO_2_ complex (^*t*^Bu_3_P)Au(CO_2_)Al(NON), which can also be thought of as a dioxocarbene complex of gold(i). Insertions of this kind are extremely rare; only a handful of such systems have been reported,^25,26^ and (^*t*^Bu_3_P)Au(CO_2_)Al(NON) represented the first structurally characterized example. Heterobimetallic CO_2_-bridged complexes of this nature had been prepared previously only *via* reactions of pre-formed metallacarboxylate nucleophiles with external metal electrophiles.^27^

From a broader perspective this unusual binding mode is interesting from the perspective of CO_2_ reduction chemistry, where the initial mode of binding to the metal(s) often determines the nature of the product formed *e.g.* CO or formate.^28^ CO_2_ produced by fossil fuels represents a large component of greenhouse gases; its utilisation as a chemical feedstock is therefore of increasing environmental importance, and understanding its potential modes of interaction with metal centres is therefore highly topical.

Given the unprecedented results seen with gold aluminyl complex **V**,^22^ we targeted (i) the synthesis of related compounds of the lighter group 11 elements *via* reactions of copper and silver electrophiles with the aluminium nucleophile **1**; and (ii) investigation of the reactivity of Cu–Al and Ag–Al bonds towards CO_2_ and other heteroallenes. No covalently bonded silver–aluminium complexes have been synthesised to date, and copper analogues have only been described very recently.^25^ It was anticipated that such complexes – if accessible – might display differing reactivity towards CO_2_ compared to **V**, owing to the variation in electronegativity of the metals on descending group 11.

## Results and discussion

### Synthesis of aluminium–coinage metal complexes

Our preliminary studies revealed that the reactions of the potassium aluminyl dimer **1** with the gold(i) halides (^*t*^Bu_3_P)AuI and (Ph_3_P)AuI lead to the formation of Au–Al covalent bonds *via* halide metathesis, and the isolation of the bi- and trimetallic systems **V** and **VI** respectively (Chart 1).^22^ A similar approach was therefore adopted for an initial exploration of the synthesis of copper and silver aluminyl complexes.

**Scheme 1:**
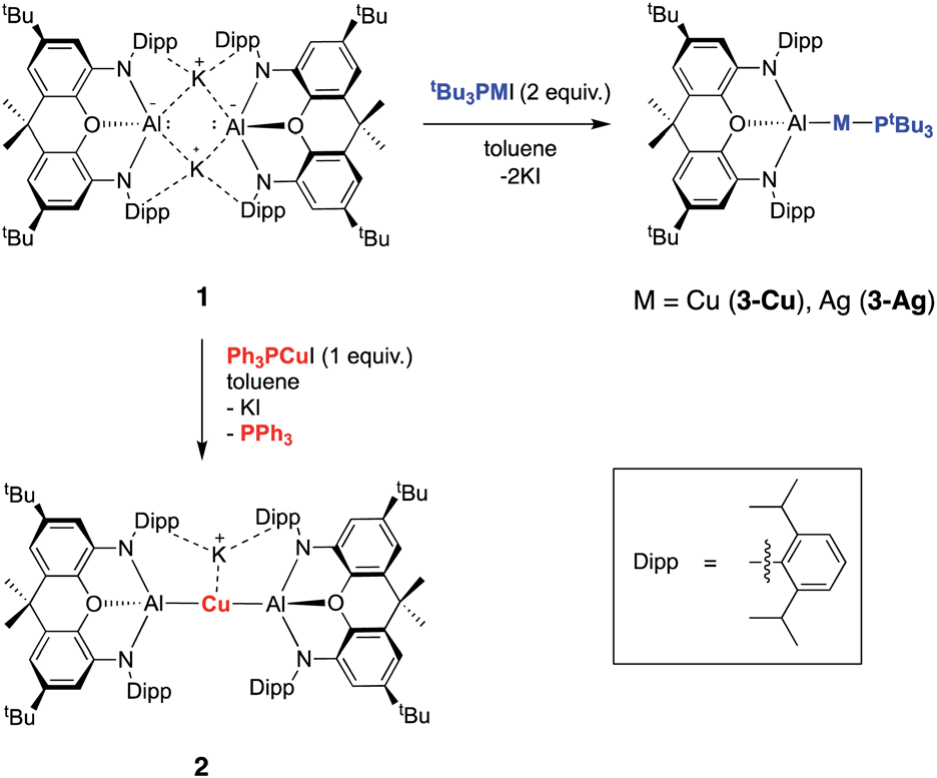
Synthesis of aluminium–silver and aluminium–copper complexes **2**, **3-Cu** and **3-Ag** from potassium aluminyl complex **1***via* salt metathesis.

**Fig. 1:**
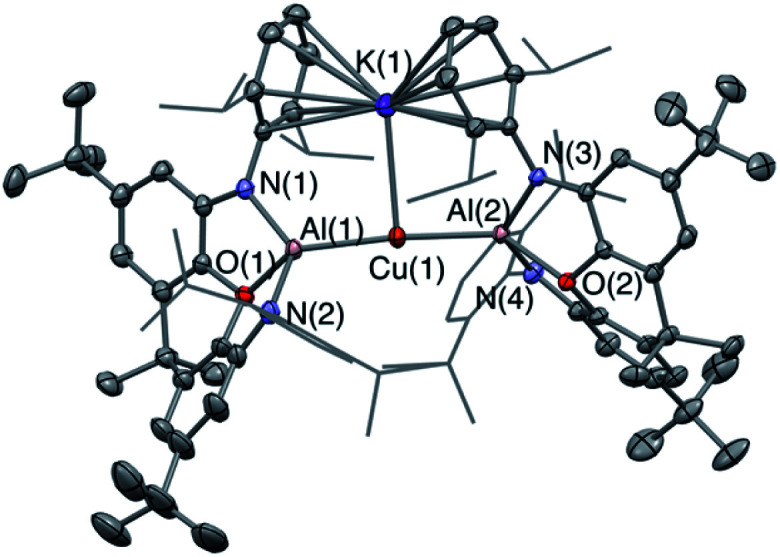
Molecular structure of **2** in the solid state as determined by X-ray crystallography. Thermal ellipsoids set at the 50% probability level. Hydrogen atoms omitted and ^i^Pr groups shown in wireframe for clarity. Key bond lengths (Å) and angles (°): Al(1)–O(1) 2.0428(15), Cu⋯K 3.0669(6), Cu(1)–Al(1) 2.4076(5), Cu(2)–Al(1) 2.4075(5), Al(1)–Cu(1)–Al(2) 174.88(2).

In contrast to the related gold chemistry, the reaction of dimeric **1** with one equivalent of (Ph_3_P)CuI generates the bisaluminyl cuprate complex K[Cu{Al(NON)}_2_] (**2**; Scheme 1). Dissociation of the phosphine ligand during the reaction is signalled by the appearance of a broad resonance at −6.0 ppm in the *in situ*^31^P NMR spectrum. **2** can subsequently be obtained by recrystallization from hexane in *ca.* 50% yield, characterized by standard spectroscopic and analytical methods, and its structure in the solid state determined by X-ray crystallography. The ^1^H NMR spectrum of the isolated product in d_6_-benzene features a pattern of resonances consistent with lower molecular symmetry for the (NON)Al fragment in solution compared to **1**. For example, whereas **1** is characterized by only one resonance for the Dipp ^i^Pr methine protons, **2** gives rise to four such signals. This lower symmetry is consistent with the structure determined crystallographically for **2** in the solid state which features a potassium cation sandwiched between the π systems of the flanking Dipp substituents on one side of the Al–Cu–Al axis (Fig. 1). Retention of this motif in benzene-d_6_ solution on the NMR timescale (as has been observed for the K^+^ cations in **1** itself)^23*a*^ accounts for the observed lower molecular symmetry. The K^+^ ion is held in place between two Dipp groups of opposing (NON) ligand frameworks, and the K⋯C distances (3.089(3)–3.296(3) Å) are slightly shorter than the equivalent contacts in **1** (3.226(3)–3.474(3) Å),^23*a*^ suggesting – if anything – a marginally stronger arene–potassium interaction.

From the perspective of coordination chemistry, the solid-state structure of **2** features a near-linear Al–Cu–Al fragment [∠(Al(1)–Cu(1)–Al(2)) = 174.88(2)°], in common with other sterically encumbered [CuX_2_]^−^ systems.^29^ The Cu–Al bond lengths (2.4076(5) and 2.4075(5) Å) are shorter than the sum of the respective covalent radii (2.53 Å) and are towards the shorter end of the range observed for solid state Cu/Al clusters, such as Cu_9_Al_4_, (2.4027(14) to 2.7189(14) Å).^30^ They are also similar to those reported very recently by Hill and co-workers for a cyclic (amino)alkyl carbene (cAAC)-supported copper aluminyl complex (2.4028(7) Å),^25^ but somewhat longer than the dispersion enhanced donor–acceptor bond measured for {HC(MeCMesN)_2_}CuAl{(NDippCMe)_2_CH} (2.3010(6) Å).^31^ The latter observation potentially reflects the mutually *trans* disposition of the (strongly donating) aluminyl ligands, and the fact that the positioning of the K^+^ counterion between the arene rings in **2** potentially constrains the approach of the (NON)Al units to the Cu(i) centre.

The reaction of **1** with two equivalents of (Ph_3_P)CuI (*i.e.* a 1 : 1 ratio Al : Cu) does not yield a compound containing a Cu–Al bond, but instead generates the mixed valence Cu(i)/Cu(0) cluster (Ph_3_P)_4_Cu_4_I_2_ (see ESI†). This chemistry contrasts with the corresponding reactivity of **1** towards (Ph_3_P)AuI, which yields the trimetallic system (NON)Al{Au(PPh_3_)}_2_I (**VI**; Chart 1), featuring an aluminium fragment bridging two formally Au(0) centres, and presumably reflects the greater electronegativity of gold *vs.* copper.^32^

With a view to generating 1 : 1 *bimetallic* systems, with retention of the M–P linkage, alternative ancillary phosphine ligands were investigated. In the case of the stronger σ-donor ^*t*^Bu_3_P, compounds of the type (^*t*^Bu_3_P)MAl(NON) (M = Cu (**3-Cu**) or Ag (**3-Ag**)) can be synthesized from the reactions of dimeric **1** with two equivalents of (^*t*^Bu_3_P)CuI or (^*t*^Bu_3_P)AgI, respectively. **3-Cu** and **3-Ag** have been characterised by ^1^H, ^31^P and ^13^C NMR spectroscopy and elemental microanalysis. The ^1^H spectra of both compounds feature a similar pattern of resonances to the (structurally characterized) gold analogue (^*t*^Bu_3_P)AuAl(NON) (**V**); the signals corresponding to the phosphine ^*t*^Bu groups and the NON ligand backbone confirm a ratio of one phosphine ligand per NON unit. The ^31^P NMR resonances for the three compounds are measured at +38.3, +58.9 and +75 ppm for **3-Cu**, **3-Ag** and **3-Au**, respectively, in line with other systems of the type (^*t*^Bu_3_P)MX.^33^ However, the single crystals of both compounds which could be obtained were too small for diffraction studies (even using synchrotron radiation). That said, the NMR and microanalytical data, together with the structure of the gallyl analogue (**3′-Ag**, see below) and the patterns of reactivity observed for **3-Cu** and **3-Ag** (see below), suggest strongly that these two compounds are isostructural with the gold system **3-Au**.

In the case of **3-Ag**, the ^31^P signal is a doublet arising from coupling to the spin-active silver nuclei. Unlike the starting material (^*t*^Bu_3_P)AgI, for example, distinct coupling to ^107^Ag and ^109^Ag cannot be resolved, presumably due to the influence of quadrupolar ^27^Al nucleus (I = 5/2). The mean ^1^*J*_PAg_ value (152 Hz), however, is very small indeed. By means of comparison, those measured for related Ag(i) compounds featuring mutually *trans* tertiary phosphine and N-heterocyclic carbene ligands (*e.g.*^1^*J*_PAg_ = 463.6, 536.0 Hz for [Ag(SIPr)(PCy_3_)][PF_6_]) are markedly larger,^33^ reflecting the very high *trans* influence of the strongly σ-donating aluminyl ligand (even compared to an NHC).

**Fig. 2:**
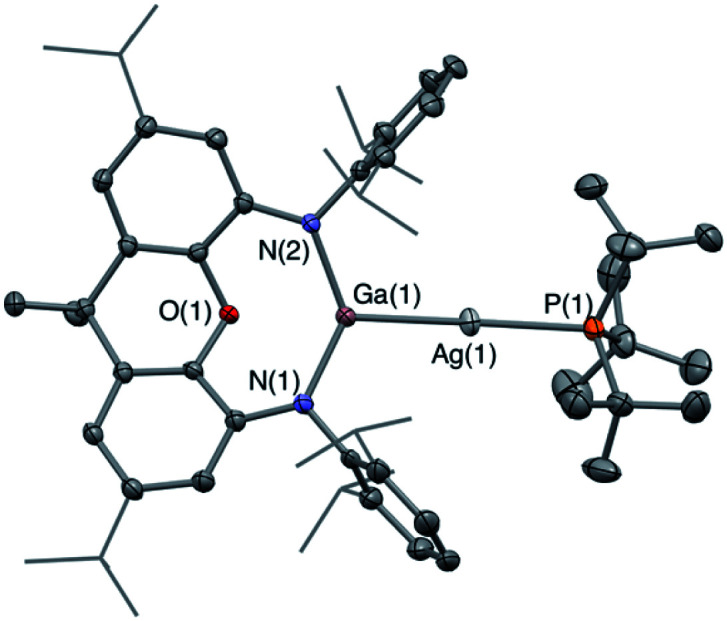
Molecular structure of **3′-Ag** in the solid state as determined by X-ray crystallography. Thermal ellipsoids set at the 50% probability level. Hydrogen atoms omitted and ^i^Pr groups shown in wireframe for clarity. Key bond lengths (Å) and angles (°): Ga(1)–O(1) 2.3834(14), Ga(1)–Ag(1) 2.4548(3), Ag(1)–P(1) 2.4355(6), Ga(1)–Ag(1)–P(1) 175.00(2).

The corresponding gallyl complex **3′-Ag** was prepared *via* the analogous reaction of the gallyl dimer K_2_[(NON)Ga]_2_ (ref. 23*a*) with (^*t*^Bu_3_P)AgI, and in this case crystals could be grown from toluene solution which were suitable for X-ray crystallography (Fig. 2). **3′-Ag** shows the expected linear Ga–Ag–P unit (∠(Al(1)–Cu(1)–Al(2)) = 175.00(2)°) analogous to the Al–Au–P motif found in **V** (∠(Al–Au–P) = 167.47(10)°). The ^31^P NMR spectrum of **3′-Ag** shows coupling to both Ag isotopes, with the signal at 65.4 ppm featuring larger coupling constants (^1^*J*_PAg_ = 278, 322 Hz) compared to that measured for **3-Ag**. This observation reflects the reduced *trans* influence of the gallyl ligand compared to its aluminyl counterpart, and (in turn) the higher electronegativity of gallium over aluminium.^32^

### Experimental studies of the reactivity of copper- and silver aluminyl compounds towards hetero-allenes

**Scheme 2:**
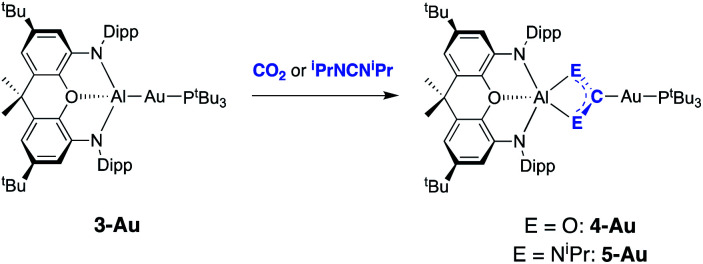
Insertion chemistry of CO_2_ and ^i^PrNCN^i^Pr with **3-Au**.^22^

In a preliminary communication we showed that **3-Au** reacts with carbon dioxide and diisopropylcarbodiimide to yield the respective insertion products featuring Au–C and Al–O or Al–N bonds (Scheme 2).^22^ The regiochemistry of the insertion process in each case is consistent with polarization of the metal–metal bond in the sense Au(*δ*−)–Al(*δ*+), and with the gold centre formally acting as the nucleophilic partner.^25,35^

**Fig. 3:**
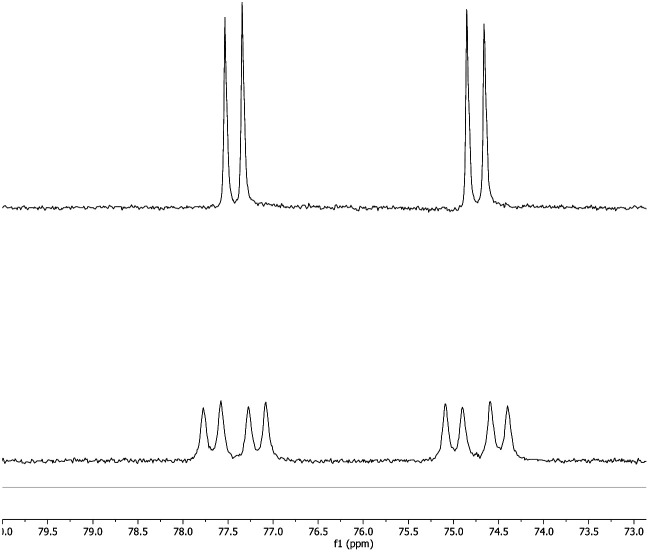
(Upper) ^31^P spectrum of **4-Ag**; (lower) ^31^P spectrum of **4-Ag-13C** prepared from **3-Ag** and ^13^CO_2_, showing additional two-bond coupling to ^13^C.

**Scheme 3:**
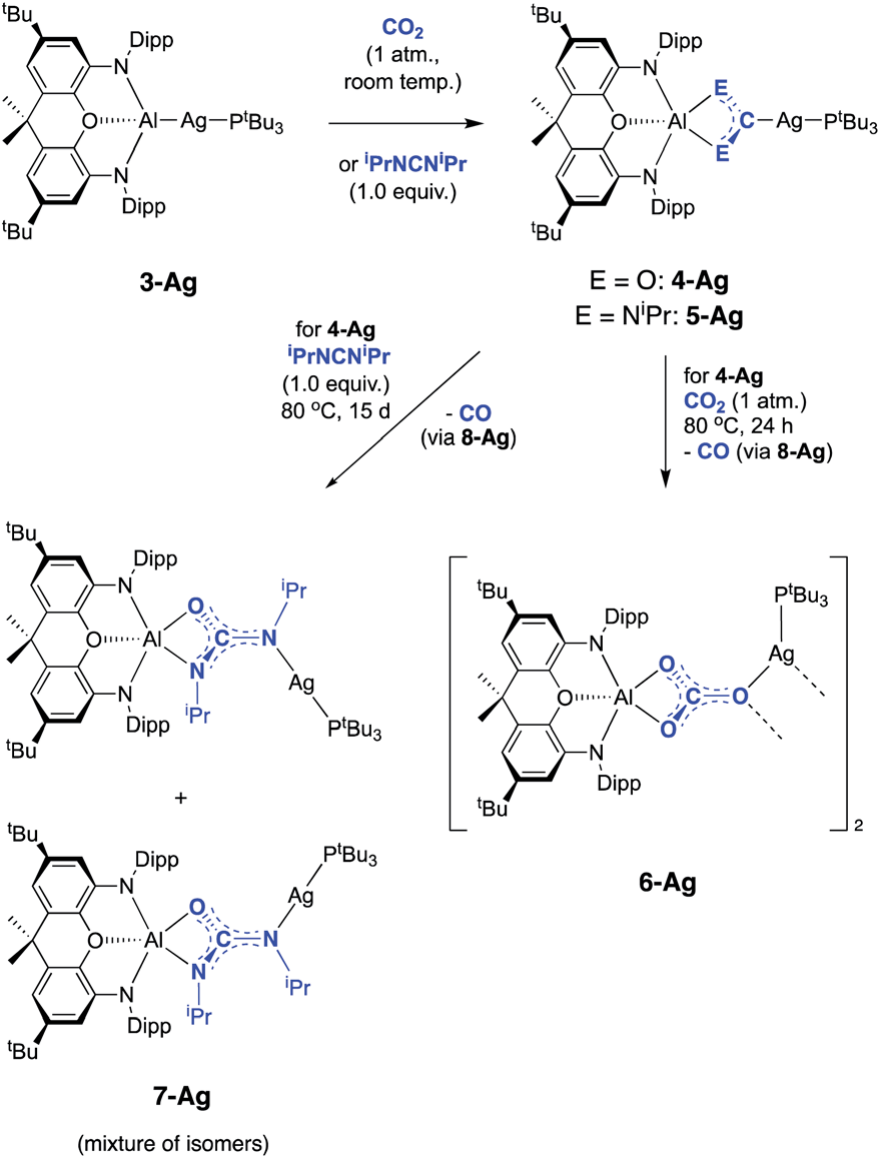
Reactions of silver–aluminium compound **3-Ag** with CO_2_ and ^i^PrNCN^i^Pr.

The potential for the lighter group 11 metal centres to act in a similar manner was therefore investigated. Silver aluminyl complex **3-Ag** was exposed to a CO_2_ atmosphere and an immediate reaction was observed. The ^1^H spectrum of the reaction mixture features a 1 : 1 ratio of signals corresponding to the (NON) and P^*t*^Bu_3_ fragments. The ^31^P NMR spectrum shows quantitative conversion to one species, with the signal at 76.0 ppm showing two sets of silver satellites, due to coupling to both ^107^Ag and ^109^Ag nuclei (^1^*J*_PAg_ = 406, 465 Hz). The magnitudes of these couplings are similar to those reported previously for complexes in which silver is bound to both an N-heterocyclic carbene (NHC) and a phosphine ligand.^34^ Furthermore, the corresponding ^31^P spectrum obtained from the reaction of **3-Ag** with ^13^CO_2_ (Fig. 3) shows additional splitting (^2^*J*_CP_ = 81 Hz) consistent with the presence of a linear P–Ag–C unit (*cf.*^2^*J*_CP_ = 62 Hz for a related NHC/phosphine complex reported by Braunstein and Danopoulos).^34*c*^ Consistently, the ^13^C spectrum of the product features an eight-line multiplet centred at 237.7 ppm due to coupling to ^31^P and both silver isotopes (^1^*J*_^107^AgC_ = 229 Hz, ^1^*J*_^109^AgC_ = 266 Hz, ^2^*J*_CP_ = 81 Hz). The low-field nature of this resonance is characteristic of metal bound carbene systems,^36^ and is close to that reported for the gold dioxo-carbene complex which is formed from the reaction of **V** and CO_2_ (*δ*_C_ = 242.3 ppm). A similar chemical shift of 239.5 ppm has also been reported for an iron bound dioxo-carbene complex.^27*a*^ The NMR evidence therefore suggests that the product of the reaction of **3-Ag** and CO_2_ is the silver dioxo-carbene complex, **4-Ag**, formed by insertion of CO_2_ into the Al–Ag bond in similar fashion to that seen with **3-Au** (Scheme 3).

**Fig. 4:**
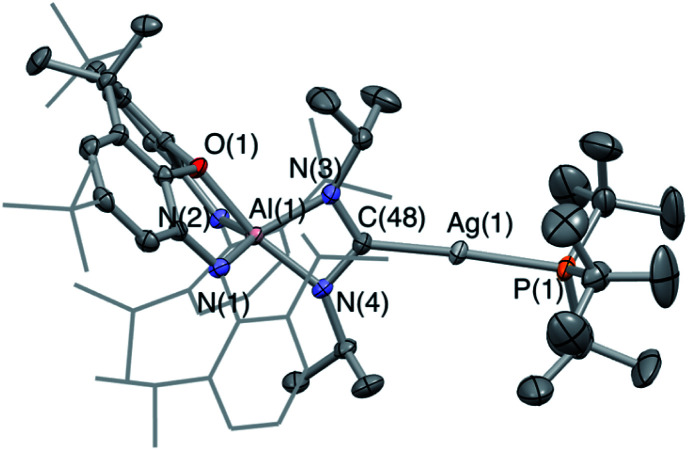
Molecular structure of **5-Ag** in the solid state as determined by X-ray crystallography. Thermal ellipsoids set at the 50% probability level. Hydrogen atoms omitted and ^i^Pr/^*t*^Bu shown in wireframe for clarity. Key bond lengths (Å) and angles (°): Ag(1)–C(48): 2.118(2), Ag(1)–P(1): 2.3851(6), N(3)–C(48): 1.346(3), N(4)–C(48): 1.345(3).

Although **4-Ag** could not be obtained as single crystals suitable for X-ray crystallography, the corresponding chemistry with carbodiimide substrates proved more amenable. The reaction of **3-Ag** with diisopropylcarbodiimide proceeds *via* a similar route, involving insertion into the Al–Ag bond to give **5-Ag** (Scheme 3). Spectroscopic evidence for this insertion process (and its regiochemistry) comes from (i) an increase in the number of resonances relating to the carbodiimide isopropyl substituents in the ^1^H NMR spectrum; (ii) the appearance of a signal in the ^31^P spectrum at 73.4 ppm showing two sets of silver satellites, with similar coupling constants to those measured for **4-Ag** (^1^*J*_PAg_ = 352, 408 Hz); and (iii) a ^13^C spectrum which shows an eight-line multiplet for the carbene ligand carbon (*δ*_C_ = 220.2 ppm; ^1^*J*_^107^AgC_ = 182 Hz, ^1^*J*_^109^AgC_ = 210 Hz, ^2^*J*_CP_ = 63 Hz) which is strikingly similar to those seen for both **4-Ag** and the product of the reaction of **3-Au** with ^i^PrNCN^i^Pr (*i.e.***5-Au**). These data are consistent with the formation of **5-Ag** by insertion of the diimide into the Al–Ag bond – an assertion which in this case could be confirmed by X-ray crystallography (Fig. 4).

The molecular structure of **5-Ag** confirms the connectivity implied by NMR measurements and shows that the two carbodiimide nitrogen atoms are bound to aluminium and the central carbon atom is coordinated to silver. As such, carbodiimide insertion into the Al–Ag bond is confirmed, with the same regioselectivity as with **5-Au**, *i.e.* with the silver centre of **3-Ag** formally acting as a nucleophile in attacking the electrophilic carbon centre of ^i^PrNCN^i^Pr. The C–N bond lengths (1.346(3) and 1.345(3) Å) lie between the typical values for single and double C–N bonds,^37^ implying that the reaction of **3-Ag** with ^i^PrNCN^i^Pr proceeds *via* a two-electron reduction of the ^i^PrNCN^i^Pr unit. Consistently, **5-Ag** can also be viewed as a Ag(i) complex of the bent (aluminate backbone supported) diisopropylamino carbene formed by the addition of two electrons to the ^i^PrNCN^i^Pr moiety. By extension, the reaction of **3-Ag** with CO_2_ generates (in **4-Ag**) the related silver dioxocarbene complex. The lower ^13^C chemical shift measured for the diamino carbene unit in **5-Ag** as compared to the dioxo species formed from CO_2_ insertion (220.2 *vs.* 237.7 ppm) finds precedent in the analogous gold compounds (219.9 *vs.* 242.3 ppm for **5-Au** and **4-Au**) and is consistent with increased π electron donation from nitrogen compared to oxygen.^38^ Although there is little further precedent for such silver dioxocarbene species, the coupling constants in the case of **5-Ag** align very closely with those reported by Braunstein for the NHC–Ag-phosphine [Ag_2_(**LtBu**)_2_][BF_4_]_2_ (**LtBu** = 3-butyl-1-(3-((di-*tert*-butyl-phosphino)methyl)phenyl)-imidazol-2-ylidene): ^1^*J*_^107^AgC_ = 190 Hz, ^1^*J*_^109^AgC_ = 219 Hz, ^2^*J*_CP_ = 62 Hz *cf.*^1^*J*_^107^AgC_ = 182 Hz, ^1^*J*_^109^AgC_ = 210 Hz, ^2^*J*_CP_ = 63 Hz in **5-Ag**.^34*c*^

While **4-Ag** is stable in solution at room temperature, it undergoes conversion to the corresponding carbonate complex **6-Ag** on heating to 80 °C for 24 h under a CO_2_ atmosphere (Scheme 3). This transformation occurs very slowly at room temperature, with no perceptible conversion observed by ^1^H NMR spectroscopy after 1 week. Spectroscopically, the ^13^C NMR spectrum of **6-Ag** is most diagnostic, with the carbonate carbon appearing as a singlet at 167.2 ppm. This shift is similar to that of the aluminium carbonate, formed from the reaction of **1** with CO_2_ (164.5 ppm).^23*a*^ Interestingly, the ^31^P NMR spectrum of **6-Ag** in d_8_-toluene at room temperature features a broad resonance at 74.0 ppm, which sharpens into a well-defined doublet (^1^*J*_PAg_ = 486 Hz) only on cooling to 203 K, suggesting the possibility that phosphine coordination might be labile at higher temperatures.

**Fig. 5:**
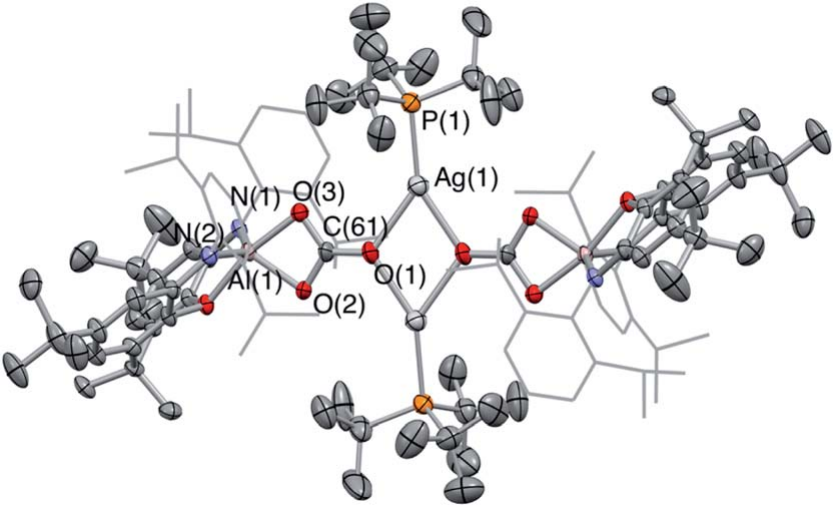
Molecular structure of **6-Ag** in the solid state as determined by X-ray crystallography. Thermal ellipsoids shown at 50%. Hydrogen atoms and solvent molecules omitted and Dipp groups shown in wireframe for clarity. Key bond lengths (Å) and angles (°); the corresponding parameters obtained by quantum chemical calculations are given in square parentheses (see below): Ag(1)–O(1): 2.249(4) [2.275(4)], O(3)–C(61): 1.307(6) [1.319(8)], O(2)–C(61): 1.297(5) [1.316(5)], O(1)–C(61): 1.266(6) [1.271(4)].

**6-Ag** can be obtained as single crystals by recrystallization from benzene, and X-ray crystallography reveals its structure to be a centrosymmetric dimer in the solid state, with two silver and two oxygen atoms constituting a planar diamond-shaped core (Fig. 5). The C–O distances are indicative of greater double bond character in the C–O bond projected towards the silver centres (*d*(O(1)–C(61)) = 1.266(6) Å, *cf. d*(O(3)–C(61)) = 1.307(6) and *d*(O(2)–C(61)) = 1.297(5) Å), consistent with the idea of greater localization of the negative charge in the [CO_3_]^2−^ moiety on the oxygen atoms bonded to the harder aluminium centre (*i.e.* O(2) and O(3)).

**Scheme 4:**
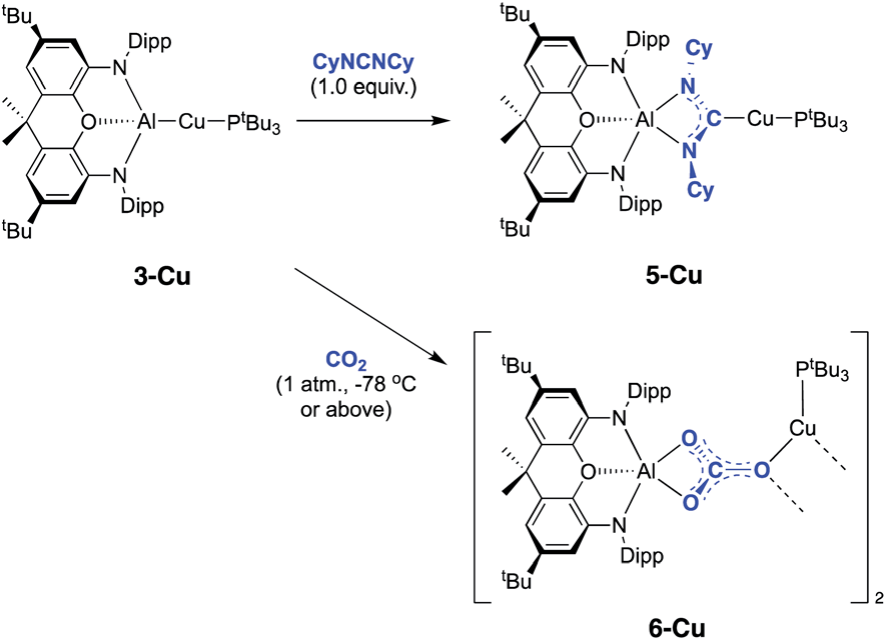
Reactions of copper-aluminium compound **3-Cu** with CO_2_ and CyNCNCy.

**Fig. 6:**
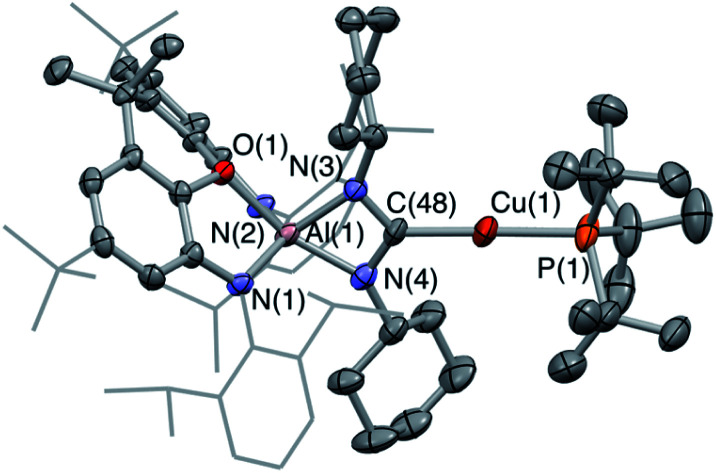
Molecular structure of **5-Cu** in the solid state as determined by X-ray crystallography. Thermal ellipsoids set at the 50% probability level. Hydrogen atoms omitted and Dipp groups shown in wireframe for clarity. Key bond lengths (Å) and angles (°): Cu(1)–C(48): 1.952(4), Al(1)–N(4): 1.908(3), Al(1)–N(3): 1.921(3).

Copper aluminyl complex **3-Cu** was also reacted with both CO_2_ and carbodiimide reagents (Scheme 4). In the case of the latter, CyNCNCy was preferred over its *iso*propyl analogue (due to enhanced crystallinity of the product), and a single new resonance appears in the ^31^P spectrum of the reaction mixture at 59.6 ppm, accompanied by loss of the signal associated with **3-Cu**. The ^13^C NMR spectrum shows a doublet at 215.5 ppm (^2^*J*_CP_ = 57.5 Hz), *i.e.* at a chemical shift very similar to those measured for the silver and gold carbodiimide insertion products **5-Ag** and **5-Au** (220.2 and 219.9 ppm, respectively). In addition, single crystals of **5-Cu** could be grown from benzene solution, and X-ray diffraction confirms that CyNCNCy undergoes insertion into the Al–Cu bond of **3-Cu** in similar manner to its heavier congeners (Fig. 6). Similar reactivity has recently been reported by Hill and co-workers for a cAAC-supported Cu aluminyl complex, although a related NHC-ligated system displays alternative selectivity for the insertion process.^25^

Comparisons of the products of carbodiimide insertion into the respective M–Al bonds (*i.e.***5-Cu**, **5-Ag** and **5-Au**), reveal properties consistent with descriptions of the three compounds as M(I) complexes featuring mutually *trans* phosphine and carbene ligands.^36^ As such, the linear geometries at M, the M–P distances and the ^13^C chemical shifts (Table 1) find precedent in the corresponding parameters reported for [(R_3_P)M(IDipp)]^+^ (M = Cu, Ag, Au; IDipp = 1,3-(diisopropylphenyl)imidazolylidene; R = Cy/^*t*^Bu).^39^ The close similarities presumably reflect the fact that the angles at the carbenic carbon defined by the two α-N substituents (108.5(3) to 109.8(9)°) are relatively wide compared to other carbene ligands featuring a four-membered heterocycle,^40^ being more in line with those measured for imidazolylidene systems. This in turn presumably reflects the relatively large size of the aluminium atom in the heterocycle backbone and the polar nature of the Al–N bonds.

**Table 1:** Selected structural and spectroscopic features of carbodiimide insertion products **5-Cu**, **5-Ag** and **5-Au**

M	*d*(M–C) (Å)	*d*(M–P) (Å)	∠(C–M–P) (°)	*δ*_C_ (ppm)	*δ*_P_ (ppm)
Cu	1.952(4)	2.2163(13)	179.04(13)	215.5	59.6
Ag	2.118(2)	2.3851(6)	176.76(8)	220.2	73.4
Au	2.158(10)	2.316(3)	175.7(3)	219.9	90.6

**Fig. 7:**
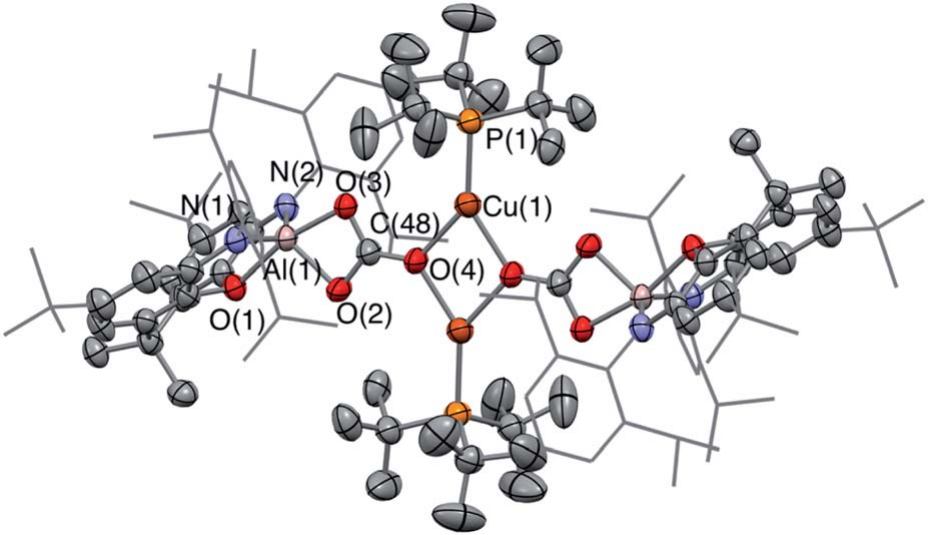
Molecular structure of **6-Cu** in the solid state as determined by X-ray crystallography. Thermal ellipsoids set at the 50% probability level. Hydrogen atoms omitted and Dipp groups shown in wireframe format for clarity. Key bond lengths (Å) and angles (°): Cu(1)–O(4): 2.062(3), C(48)–O(3): 1.874(3), C(48)–O(4): 1.268(6), C(48)–O(2): 1.296(6).

In the reaction of **3-Cu** with CO_2_, the ^31^P NMR spectrum of the reaction mixture shows immediate loss of the signal associated with the starting material, and the appearance of a single resonance at *δ*_P_ = 62.5 ppm. The ^13^C NMR spectrum, however, shows no highly deshielded doublet diagnostic of a carbene-like moiety. Instead, a singlet at *δ*_C_ = 170 ppm implies that the product of this reaction, even under mild conditions, features a carbonate group. This hypothesis can be confirmed by X-ray crystallography, which shows that the carbonate product formed (**6-Cu**), is isostructural with its silver analogue **6-Ag** (Fig. 7). The reaction was also studied *in situ* using low temperature multinuclear NMR spectroscopy. However, it was found that full conversion to **6-Cu** is observed even at −78 °C and no carbene-like intermediate could be observed even under such conditions.

A number of general observations can be made concerning the reactivity of the copper, silver and gold complexes towards CO_2_ and carbodiimides. In the latter case, all three systems, **3-Cu**, **3-Ag** and **3-Au**, can be shown by X-ray crystallography to undergo insertion of the heteroallene *via* a process which implies nucleophilic character at the group 11 metal centre. In none of these cases is any further reactivity observed with excess carbodiimide. In the case of CO_2_, however, onward reactivity appears to be possible (to an extent depending on the identity of the group 11 metal), proceeding from the analogous first-formed dioxocarbene species to yield a carbonate. In the case of gold, the carbene-like species **4-Au** shows no hint of onward reactivity with excess CO_2_, even under forcing conditions. In the silver case, further reaction with CO_2_ is possible to give **6-Ag**, but this reaction proceeds slowly and only at elevated temperatures. For the copper system, conversion to the carbonate species **6-Cu***via* reaction with two equivalents of CO_2_ occurs so rapidly even at low temperatures that the initial insertion product, **4-Cu**, cannot be directly observed. Superficially, this trend reflects the respective oxo- and carbophilicities of the group 11 elements, with M–O bond strengths decreasing and M-C bonds becoming stronger on going from copper to gold.^41^

### Probes of the mechanism of CO_2_ insertion and transformation

#### Experimental studies

**Fig. 8:**
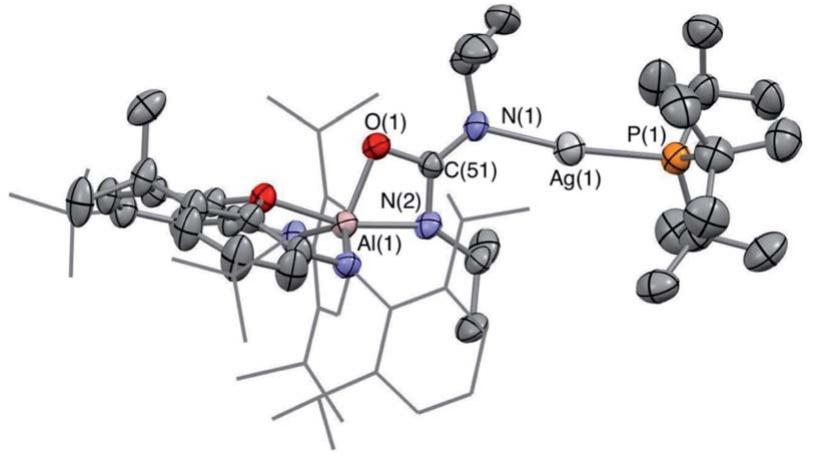
Molecular structure of **7-Ag** as determined by X-ray crystallography. Thermal ellipsoids set at the 50% probability level. Hydrogen atoms omitted and Dipp groups shown in wireframe format for clarity. Key bond lengths (Å) and angles (°): Ag(1)–P(1): 2.3572(14), Ag(1)–N(1): 2.130(4), N(1)–C(51): 1.304(6), N(2)–C(51): 1.339(6), Al(1)–O(1): 1.842(3), O(1)–C(51): 1.344(5).

Experimental probes of the mechanism of carbonate formation focussed on the silver system, given the (unique) accessibility of both carbene complex **4-Ag** and carbonate **6-Ag**. Initially we focussed on ‘crossover’ experiments employing CO_2_ and ^i^PrNCN^i^Pr in stepwise fashion. While the carbodiimide insertion product **5-Ag** is unreactive towards CO_2_ under all conditions investigated, the reaction of **4-Ag** with ^i^PrNCN^i^Pr proceeds slowly, and over the course of 15 d the ^31^P resonance associated with **4-Ag** gives way to two sets of signals in a *ca.* 2 : 1 ratio at 78.8 and 80.1 ppm (both with ^1^*J*_^107^AgP_ = 540, ^1^*J*_^109^AgP_ = 623 Hz). The ^13^C spectrum shows two signals also in an approximately 2 : 1 ratio at 167.8 and 167.2 ppm (*i.e.* at chemical shifts similar to the carbonate resonances in **6-Ag** and **6-Cu**), and also shows a signal for free carbon monoxide (at *δ*_C_ = 184.5 ppm). X-ray crystallography reveals that the silver-containing product of this reaction is the ureate compound **7-Ag** (Fig. 8 and Scheme 3) formed as a result of net CO extrusion from **4-Ag** and uptake of the carbodiimide. In the solid state, the product is characterized by a short central C–N bond (*d*(N(1)–C(51)) = 1.304(6) Å) featuring a *trans* arrangement of the O and Ag(P^*t*^Bu_3_) substituents. Restricted rotation about the C=N bond in solution then provides a rationale for the two products observed by NMR (*i.e.* with the second species featuring the alternative *cis* alignment of the O and Ag(P^*t*^Bu_3_) functions).

**Fig. 9:**
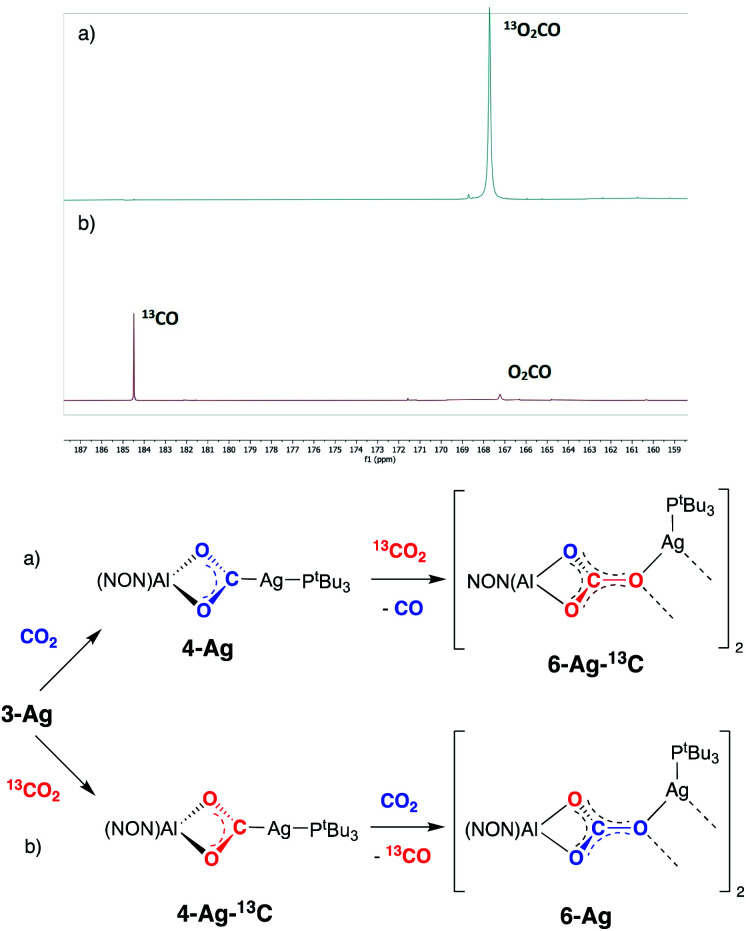
^13^CO_2_ ‘crossover’ labelling experiments (and their associated spectra) carried out with **4-Ag** and **4-Ag-13C**: (a) addition of ^13^CO_2_ to **4-Ag** to yield **6-Ag-13C** and CO; (b) addition of unlabelled CO_2_ to **4-Ag-13C** to yield **6-Ag** and ^13^CO.

While the formation of **7-Ag** from **4-Ag** and ^i^PrNCN^i^Pr implies that CO is lost from the dioxocarbene compound, in an overall process also involving uptake of the carbodiimide, we sought to obtain experimental evidence for similar steps in the formation of the carbonate compound **6-Ag**. An *in situ*^13^C NMR spectrum of the reaction mixture comprised of **4-Ag** and CO_2_ at 80 °C is consistent with the evolution of carbon monoxide. In addition, details of the CO extrusion process and uptake of a second CO_2_ molecule were probed using ^13^CO_2_ labelling studies (Fig. 9). These involved two separate experiments: (a) exposure of a sample of **4-Ag** to ^13^CO_2_ at 80 °C and (b) exposure of a sample of **4-Ag-13C** (itself prepared from **3-Ag** and ^13^CO_2_) to unlabelled CO_2_ under similar conditions. The resulting ^13^C NMR spectra clearly show that the extruded CO originates from the first CO_2_ unit (*i.e.* from **4-Ag** or **4-Ag-13C**), and that the carbon incorporated into the resulting carbonate complex arises from the second molecule of CO_2_.

**Scheme 5:**
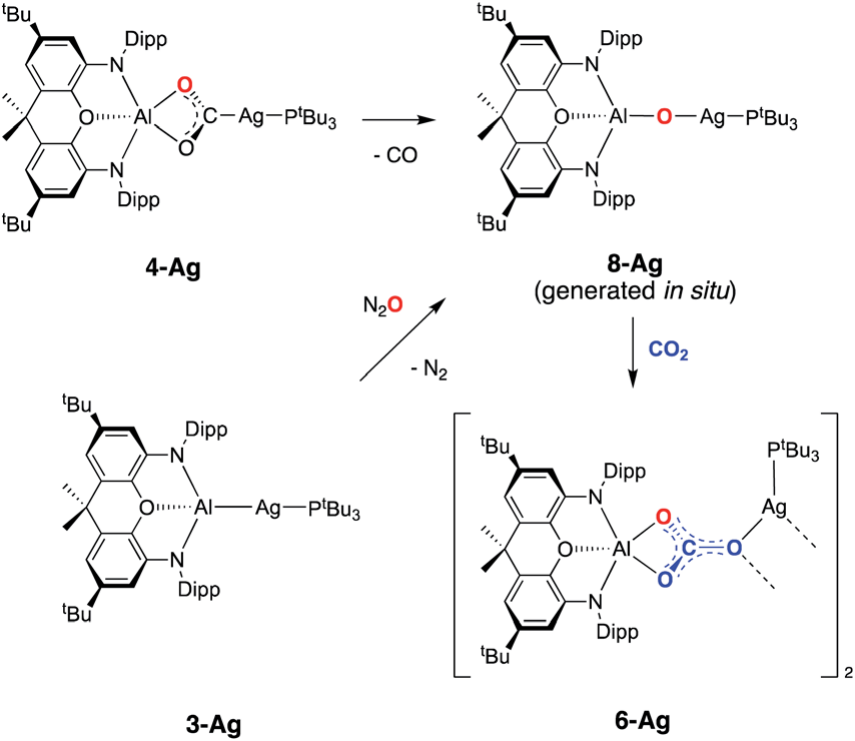
Potential pathway for the formation of carbonate complex **6-Ag***via* an oxide intermediate.

On this basis, a potential intermediate in the conversion of **4-Ag** to **6-Ag** is a bimetallic species featuring a bridging oxide between the aluminium and silver centres. Such a species would be formed *via* CO extrusion from **4-Ag**, and then assimilate the second molecule of CO_2_ to give the carbonate product **6-Ag** (Scheme 5).^42^ A similar mechanism for CO_2_ reduction to CO by a digermyne complex has been proposed by Jones and Frenking.^26*b*^

To obtain experimental evidence for this proposal, the proposed intermediate (^*t*^Bu_3_P)AgOAl(NON) (**8-Ag**) was independently synthesised by exposing **3-Ag** to an N_2_O atmosphere at −78 °C (Scheme 5). The reaction was followed *in situ* by ^1^H NMR spectroscopy, which shows conversion under such conditions to a single species which features a 1 : 1 ratio of NON and P^*t*^Bu_3_ ligands. The corresponding ^31^P spectrum features a single signal at 82.0 ppm displaying silver satellites (^1^*J*_^107^AgP_ = 551, ^1^*J*_^109^AgP_ = 640 Hz). The greatly increased magnitudes of the ^1^*J*_PAg_ coupling constants (compared to **3-Ag**) implies that the aluminyl ligand is no longer directly bonded to silver, and that it has been replaced by an appreciably poorer σ-donor. Although **8-Ag** proved to be too thermally fragile to be isolated in bulk quantities, it could be generated *in situ* for reactivity studies by degassing the reaction mixture to remove excess N_2_O. At this point, exposure to an atmosphere of CO_2_ led to an immediate reaction and quantitative formation of carbonate compound **6-Ag**, as evidenced by ^1^H and ^31^P NMR spectroscopy, and X-ray crystallography (Scheme 5). In similar fashion, the reaction of **8-Ag** with diisopropylcarbodiimide at room temperature rapidly forms ureate complex **7-Ag**, with the same apparent selectivity for the two isomers as seen in the reaction with **4-Ag**.

#### Quantum chemical studies

**Fig. 10:**
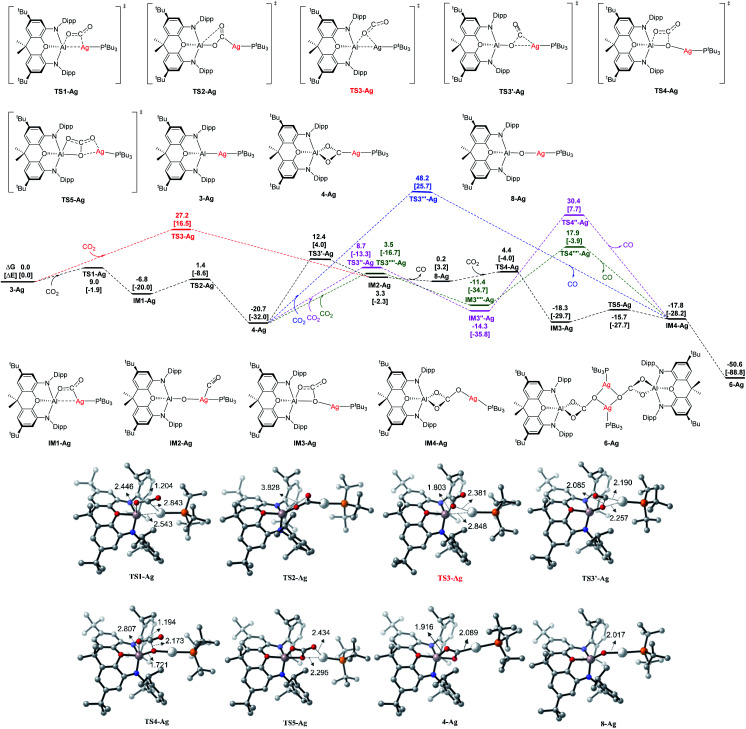
Computed Gibbs energy profiles (in kcal mol^−1^) for Ag at the BP86+D3(BJ)/def2-TZVPP (SMD, solvent = benzene)//BP86/def2-SVP level, and the electronic energies are given in bracket for reference. Other structures not shown along the reaction pathways are given in Fig. S18.† Key bond distances are given in Å for some optimized key intermediate and transition states. Color code, C: grey, N: blue, O: red, P: orange, Al: pink, Ag: white.

We calculated the reaction course of the CO_2_ addition to compound **3-Ag** sketched in Scheme 5 using density functional theory (DFT) at the BP86+D3(BJ)/def2-TZVPP level using BP86/def2-SVP optimized geometries. Fig. 10 shows the computed reaction profiles along with some key optimized structures. Solvent effects of benzene have been estimated with the SMD continuum solvation model. Computational details are given in ESI.†

The addition of one molecule of CO_2_ to **3-Ag** takes place with very low activation energies *via* the intermediate **IM1-Ag**, giving the dioxocarbene complex **4-Ag** as the first product. The calculations suggest that CO_2_ uptake starts with side-on [2 + 2] addition of one C=O bond to the Al–Ag bond (with a regiochemistry reflecting polarization in the sense Ag(*δ*−)–Al(*δ*+)),^25,35,43^ and that the intermediate **IM1-Ag** then rearranges *via***TS2-Ag** to give dioxocarbene complex **4-Ag**. From this species, the second part of the reaction sequence **4-Ag** → **6-Ag** proceeds *via* initial CO extrusion, yielding **8-Ag** as an intermediate (featuring an Ag–O–Al unit), preceded by **IM2-Ag** as a weakly bonded silver–CO complex precursor. Subsequent reaction steps from **8-Ag** have low activation barriers, leading first to **IM3-Ag***via* uptake of a second CO_2_ molecule across the Ag–O bond, followed by isomerization to the monomer **Im4-Ag**, which then dimerizes to give **6-Ag** as the final product.

The highest energy barrier for the overall reaction is the rearrangement/CO extrusion step from **4-Ag** to **IM2-Ag***via***TS3′-Ag**, which has a value of Δ*G*^‡^ = 33.1 kcal mol^−1^. The alternative one-step pathway **3-Ag** → **IM2-Ag***via***TS3-Ag** has a lower overall barrier of Δ*G*^‡^ = 27.2 kcal mol^−1^, but the uptake of CO_2_ in the initial step, leading to **4-Ag** is both kinetically and thermodynamically much more favorable. The compound **4-Ag** is thus the energy reference species for the CO_2_ addition. The most favorable reaction pathway is given in black lines in Fig. 10. Energetically less favorable courses are shown in red, blue and green.

We also calculated the analogous reactions of CO_2_ with the copper and gold aluminyl complexes **3-Cu** and **3-Au**. The calculated energy profiles are shown in Fig. S19 and S20 of ESI.† The theoretical data suggest that the reaction sequence in the case of the copper species follows the analogous pathway from **3-Cu** to **6-Cu** as the silver species with a lower activation barrier for the rate determining step (**4-Cu** → **IM2-Cu***via***TS3-Cu**) of Δ*G*^‡^ = 27.4 kcal mol^−1^, which agrees with the experimental finding of much more facile carbonate formation in the case of copper. The calculations for the gold homologues give a similar reaction profile for the initial CO_2_ uptake leading to **4-Au**, but the subsequent rearrangement has a very high barrier of Δ*G*^‡^ = 44.5 kcal mol^−1^ with concomitant loss of CO leading to **IM3-Au** (Fig. S20†). This finding is also in agreement with the experimental results, *i.e.* with the fact that **4-Au** can be isolated without complications arising from further onward reactivity.

## Conclusions

The reactions of group 11 metal aluminyl compounds of the type (^*t*^Bu_3_P)MAl(NON) (M = Cu, Ag, Au) with carbon dioxide and with carbodiimides are shown to proceed *via* insertion into the polar metal-aluminium bonds to yield species featuring μ-κ^1^(C):κ^2^(E,E′) bridging units and M–C/Al–E bonds, and which can be thought of as aluminium-functionalized carbene adducts of the respective coinage metals. While the products of carbodiimide insertion, (^*t*^Bu_3_P)M{C(NR)_2_}Al(NON), are stable to further reaction in the presence of excess of the heteroallene (and have been structurally characterized for all three metals), the corresponding CO_2_ insertion products are labile to an extent dependent on the nature of M. Thus, (^*t*^Bu_3_P)Au(CO_2_)Al(NON) is inert to further reaction under all conditions examined (and can be structurally characterized), while (^*t*^Bu_3_P)Ag(CO_2_)Al(NON) can be generated at room temperature but slowly converts into the corresponding carbonate (^*t*^Bu_3_P)Ag(CO_3_)Al(NON) (and CO), and (^*t*^Bu_3_P)Cu(CO_2_)Al(NON) has been identified only in quantum chemical experiments as part of a facile route to (^*t*^Bu_3_P)Cu(CO_3_)Al(NON). Both experimental and computational studies are consistent with a mechanism for the formation of the carbonate species which proceeds *via* rate-limiting extrusion of CO from (^*t*^Bu_3_P)M(CO_2_)Al(NON) (M = Cu, Ag) to give a reactive oxide species containing a M–O–Al unit, which then rapidly assimilates further CO_2_. The comparative labilities of the species (^*t*^Bu_3_P)M(CO_2_) Al(NON) (M = Cu, Ag, Au) can be quantified by activation energies of Δ*G*^‡^ = 26.6, 33.1, 44.5 kcal mol^−1^, respectively, for the most feasible CO extrusion step, and rationalized in turn on the basis of the relative carbophilicities of the coinage metals.^41*a*^

## Data availability

See ESI for complete synthetic, quantum chemical and crystallographic details.†

## Author contributions

C. McManus: carried out the experimental investigation; J. Hicks carried out the experimental investigation; X. Cui: carried out the quantum chemical investigation; L. Zhao: conceptualization and supervision of quantum chemical investigation; G. Frenking: conceptualization and supervision of quantum chemical investigation; J. M. Goicoechea: conceptualization and supervision of experimental investigation; S. Aldridge: conceptualization and supervision of experimental investigation.

## Conflicts of interest

There are no conflicts to declare.

## Supplementary Material

SC-012-D1SC04676D-s001

SC-012-D1SC04676D-s002
