# Supplementary material for: Coinage metal aluminyl complexes: probing regiochemistry and mechanism in the insertion and reduction of carbon dioxide
Source: Chem Sci. 2021 Sep 16;12(40):13458–68. doi: 10.1039/d1sc04676d (PMC8528051; doi:10.1039/d1sc04676d)
Supplement: SC-012-D1SC04676D-s001 [file SC-012-D1SC04676D-s001.pdf]

Electronic Supplementary Material (ESI) for Chemical Science.
This journal is © The Royal Society of Chemistry 2021

Coinage metal aluminyl complexes: probing regiochemistry and mechanism in the insertion and reduction of carbon dioxide

Caitilín McManus, Jamie Hicks, Xianlu Cui, Lili Zhao,* Gernot Frenking,* Jose M. Goicoechea* and Simon Aldridge*

Inorganic Chemistry Laboratory, Department of Chemistry, University of Oxford, South Parks Road, Oxford, OX1 3QR, UK
Fachbereich Chemie, Philipps-Universitaẗ Marburg, D-35043 Marburg, Germany
Institute of Advanced Synthesis, School of Chemistry and Molecular Engineering, Jiangsu National Synergetic Innovation Center for Advanced Materials, Nanjing Tech University, Nanjing 211816, P. R. China.

Supporting Information (57 pages)

1. General experimental considerations s2
2. Syntheses of novel compounds s3
3. Representative ^{1}H and ^{13}C NMR spectra of new compounds s7
4. Crystallographic details s15
5. Computational details including xyz files for optimized structures s18
6. References for supporting information s57

1. General experimental considerations

All manipulations were carried out using standard Schlenk line or dry-box techniques under an atmosphere of argon or dinitrogen. Solvents were degassed by sparging with argon and dried by passing through a column of the appropriate drying agent. NMR spectra were measured in benzene-d_6 (which was dried over potassium) or toluene-d_8 (which was dried over CaH_2), with the solvent then being distilled under reduced pressure and stored under argon in Teflon valve ampoules. NMR samples were prepared under argon in 5 mm Wilmad 507-PP tubes fitted with J. Young Teflon valves. ^{1}H, $^{13}C\{^{1}H\}$ and $^{31}P\{^{1}H\}$ NMR spectra were recorded on Bruker Avance III HD nanobay 400 MHz or Bruker Avance III 500 MHz spectrometers at ambient temperature and referenced internally to residual protio-solvent (^{1}H) or solvent (^{13}C) resonances and are reported relative to tetramethylsilane (δ = 0 ppm). ^{31}P resonances are referenced externally to H_3PO_4 (85 %). Assignments were confirmed using two-dimensional ^{1}H-^{1}H and ^{13}C-^{1}H NMR correlation experiments. Chemical shifts are quoted in δ (ppm) and coupling constants in Hz. Elemental analyses were carried out by Elemental Microanalysis Ltd., Devon. A number of bimetallic systems reported in this study consistently (over 4+ measurements) gave analytical data that are within acceptable error bounds for N and H, but marginally low in C - potentially due to carbide formation.

$[K\{Al(NON)\}]_2$ (**1**) and $[K\{Ga(NON)\}]_2$ were prepared via minor modifications to the literature methods.[s1] $(Ph_3P)CuI$, $(^{t}Bu_3P)CuI$ and $(^{t}Bu_3P)AgI$ were prepared following the general synthetic procedure outlined by Goel, Beauchamp et al.[s2] All other reagents were used as received.

2. Syntheses of novel compounds

K[{(NON)Al}$_2$Cu (2): A suspension of (Ph$_3$P)CuI (0.060 g 0.066 mmol) and **1** (0.200g, 0.136 mmol) in benzene (10 mL) was stirred at room temperature for 16 h. The resulting mixture was filtered and the volatiles removed under vacuum. The residue was dissolved in hexane (10 mL), filtered, concentrated to 5 mL and heated to 50 °C. Slow cooling to room temperature yielded very air sensitive yellow crystals of **2**, as the hexane hemi-solvate. Yield: 60 mg, 53 %. ^{1}H NMR (400 MHz, C$_6$D$_6$, 373 K): δ_H = 1.00 (d, $^3J_{HH}$ = 6.9 Hz, 12H, CH(C*H*$_3$)$_2$), 1.27 (s, 18H, C(C*H*$_3$)$_3$), 1.42 (d, $^3J_{HH}$ = 6.8 Hz, 12H, CH(C*H*$_3$)$_2$), 1.65 (s, 6H, C(C*H*$_3$)$_2$), 3.77 (sept, $^3J_{HH}$ = 6.8 Hz, 4H, C*H*(CH$_3$)$_2$), 6.36 (d, $^4J_{HH}$ = 1.9 Hz, 2H, XA-*o*-C*H*), 6.76 (d, $^4J_{HH}$ = 1.9 Hz , 2H, XA-*p*-C*H*), 7.24-26 (m, 6H, Ar*H*). ^{13}C{^{1}H} NMR (126 MHz, C$_7$D$_8$, 373 K): δ_C = 25.4, 26.2 (CH(*C*H$_3$)$_2$), 29.9 (*C*H(CH$_3$)$_2$), 31.9 (xan-C(*C*H$_3$)$_2$), 32.1 (C(*C*H$_3$)$_3$), 35.4 (*C*(CH$_3$)$_3$), 38.0 (xan-*C*(CH$_3$)$_2$), 106.5, 109.1, 124.4, 124.5, 128.5, 134.4, 134.7, 143.4, 143.9, 148.6, 150.3 (Ar-*C*). The ^{1}H and ^{13}C NMR spectra of **2** are relatively broad at room temperature and suffer from reduced compound solubility as the temperature is lowered. We therefore report the spectra at 373 K, at which temperature, the signals are relatively sharp, and are consistent with rapid exchange. Elemental microanalysis: calc. for C$_{94}$H$_{124}$Al$_2$CuKN$_4$O$_2$: C 75.34 %, H 8.34 %, N 3.74 %, meas.: C 74.81 %, H 8.72 %, N 3.30 %

(NON)AlAgP^{t}Bu$_3$ (3-Ag): A suspension of (^{t}Bu$_3$P)AgI (0.110 g, 0.271 mmol) and **1** (0.200 g, 0.136 mmol) in toluene (10 mL) was stirred at room temperature for 16 h. The resulting mixture was filtered to give a pale green solution which was concentrated to 3 mL and slowly cooled to 3 °C. Colourless microcrystals of **3-Ag** were isolated by filtration and washed with pentane. Yield: 0.120 mg, 46 %. ^{1}H NMR (400 MHz, C$_6$D$_6$, 298 K): δ_H = 0.91 (d, $^3J_{HP}$ = 10.7 Hz, 27H, PC(C*H*$_3$)$_3$), 1.23 (d, $^3J_{HH}$ = 6.9 Hz, 12H, CH(C*H*$_3$)$_2$), 1.27 (s, 18H, C(C*H*$_3$)$_3$), 1.42 (d, $^3J_{HH}$ = 6.8 Hz, 12H, CH(C*H*$_3$)$_2$), 1.65 (s, 6H, C(C*H*$_3$)$_2$), 3.77 (sept, $^3J_{HH}$ = 6.8 Hz, 4H, C*H*(CH$_3$)$_2$), 6.36 (d, $^4J_{HH}$ = 1.9 Hz, 2H, XA-*o*-C*H*), 6.76 (d, $^4J_{HH}$ = 1.9 Hz , 2H, XA-*p*-C*H*), 7.24-26 (m, 6H Ar*H*). ^{13}C{^{1}H} NMR (126 MHz, C$_6$D$_6$, 298 K): δ_C = 25.5, 25.7 (CH(*C*H$_3$)$_2$) 27.8 (*C*(CH$_3$)$_2$), 29.3 (*C*H(CH$_3$)$_2$), 31.8 (C(*C*H$_3$)$_3$), 32.1 (d, $^2J_{CP}$ = 6.0 Hz, P{C(*C*H$_3$)$_3$}$_3$), 35.1 (*C*(CH$_3$)$_3$), 36.7 (dd, $^2J_{CAg}$ = 4.0, 6.8 Hz, P{*C*(CH$_3$)$_3$}$_3$), 37.2(*C*(CH$_3$)$_2$), 105.8, 110.5, 123.9, 125.6, 128.3, 132.6, 142.2, 142.8, 143.3, 148.0, 148.3 (Ar*C*). ^{31}P{^{1}H} NMR (162 MHz, C$_6$D$_6$, 298 K): δ_P = 58.9 (d, $^1J_{PAg}$ = 160.6 Hz). Elemental microanalysis: calc. for C$_{59}$H$_{89}$AgAlN$_2$OP: C 70.29 %, H 8.90 %, N 2.78 %, meas.: C 69.38 %, H 8.52 %, N 2.81 %

(NON)CuAgP^{t}Bu$_3$ (3-Cu): A suspension of (^{t}Bu$_3$P)CuI (0.107 g, 0.271 mmol) and **1** (0.200 g 0.136 mmol) in toluene (10 mL) was stirred at room temperature for 16 h. The resulting mixture was filtered to give a pale green solution which was concentrated to 3 mL and slowly cooled to 3 °C. Colourless microcrystals of **3-Cu** were isolated by filtration and washed with pentane. Yield: 110 mg, 42 %. ^{1}H NMR (400 MHz, C$_6$D$_6$, 298 K): δ_H = 0.90 (d, $^3J_{HP}$ = 12.2 Hz, 27H, PC(C*H*$_3$)$_3$), 1.21 (d, $^3J_{HH}$ = 6.9 Hz, 12H, CH(C*H*$_3$)$_2$), 1.28 (s, 18H, C(C*H*$_3$)$_3$), 1.45 (d, $^3J_{HH}$ = 7.0 Hz, 12H, CH(C*H*$_3$)$_2$), 1.67 (s, 6H, C(C*H*$_3$)$_2$), 3.79 (sept, $^3J_{HH}$ = 7.0 Hz, 4H, C*H*(CH$_3$)$_2$), 6.31 (d, $^4J_{HH}$ = 1.9 Hz, 2H, XA-*o*-C*H*), 6.76 (d, $^4J_{HH}$ = 1.9 Hz , 2H, XA-*p*-C*H*), 7.19-27 (m, 6H Ar*H*). ^{13}C{^{1}H} NMR (126 MHz, C$_6$D$_6$, 298 K): δ = 25.3, 25.9 (CH(*C*H$_3$)$_2$) 27.7 (*C*(CH$_3$)$_2$), 29.3 (*C*H(CH$_3$)$_2$), 31.9 (C(*C*H$_3$)$_3$), 32.1 (d, $^2J_{CP}$ = 6.0 Hz, P{C(*C*H$_3$)$_3$}$_3$), 35.1 (*C*(CH$_3$)$_3$), 36.8 (*C*(CH$_3$)$_2$), 37.3 (P{*C*(CH$_3$)$_3$}$_3$), 105.7, 110.6, 124.0, 125.7, 133.7, 142.6, 143.2, 143.79, 147.8, 148.1 (Ar-*C*). ^{31}P{^{1}H} NMR (162 MHz, C$_6$D$_6$, 298 K): δ_P = 38.3. Elemental microanalysis: calc. for C$_{59}$H$_{89}$CuAlN$_2$OP: C 73.52 %, H 9.31 %, N 2.91 %, found: C 72.72 %, H 8.75 %, N 3.05 %

(NON)GaAgP^{t}Bu$_3$ (3'-Ag) A solution of K_2[Ga(NON)]$_2$ (200 mg, 0.128 mmol) and (^{t}Bu$_3$P)AgI (108 mg, 0.256) in toluene (10 mL) was stirred at room temperature for 4 h. The resulting mixture was filtered to give a yellow solution which was concentrated to ca. 5mL. Crystals of **3'-Ag** (as the toluene bis solvate) suitable for X-ray crystallography were obtained by slow cooling to 3 °C. Yield: 210 mg, 78 %. ^{1}H NMR (400 MHz, C_6D_6, 298 K): δ_H = (d, $^3J_{HP}$ = 12.1 Hz, 27H, PC(C*H*$_3$)$_3$), 1.25 (d, $^3J_{HH}$ = 6.7 Hz, 12H, CH(C*H*$_3$)$_2$), 1.33 (s, 18H, C(C*H*$_3$)$_3$), 1.35 (d, $^3J_{HH}$ = 7.1 Hz, 12H, CH(C*H*$_3$)$_2$), 1.74 (s, 6H, C(C*H*$_3$)$_2$), 3.80 (sept, $^3J_{HH}$ = 6.8 Hz, 4H, C*H*(CH$_3$)$_2$), 6.39 (d, $^4J_{HH}$ = 2.0 Hz, 2H, XA-*o*-C*H*), 6.81 (d, $^4J_{HH}$ = 2.0 Hz , 2H, XA-*p*-C*H*), 7.20-7.27 (m, 6H Ar*H*). ^{13}C{^{1}H} NMR (126 MHz, C_6D_6, 298 K): δ_C = 25.3, 25.9 (CH(*C*H$_3$)$_2$) 27.7 (*C*(CH$_3$)$_2$), 29.3 (*C*H(CH$_3$)$_2$), 31.9 (C(*C*H$_3$)$_3$), 32.1 (d, $^2J_{CP}$ = 6.0 Hz, P{C(*C*H$_3$)$_3$}$_3$), 35.1 (*C*(CH$_3$)$_3$), 36.8 (*C*(CH$_3$)$_2$), 37.3 (P{*C*(CH$_3$)$_3$}$_3$), 105.7, 110.6, 124.0, 125.7, 133.7, 142.6, 143.2, 143.8, 147.8, 148.1 (Ar*C*). ^{31}P{^{1}H} NMR (162 MHz, C_6D_6, 298 K): δ_P = 65.4 (dd, $^1J_{P\text{-}107Ag}$ = 278, $^1J_{P\text{-}109Ag}$ = 322 Hz). Elemental microanalysis: calc. for $C_{59}H_{89}AgGaN_2OP$: C 67.43 %, H 8.54 %, N 2.67 %; meas.: C 66.81 %, H 8.14 %, N 2.64 %.

(NON)Al(O$_2$C)AgP^{t}Bu$_3$ (4-Ag) A solution of **3-Ag** (100 mg, 0.100 mmol) in benzene was degassed and exposed to CO_2 (ca. 1.0 atm). After 15 minutes, volatiles were removed under vacuum and the residue washed with pentane to give **4-Ag** as a white solid. Yield: 80 mg, 76 %. ^{1}H NMR (400 MHz, C_6D_6, 298 K): δ_H = 0.81 (d, $^3J_{HP}$ = 12.7 Hz, 27H, PC(C*H*$_3$)$_3$), 1.23 (d, $^3J_{HH}$ = 6.9 Hz, 12H, CH(C*H*$_3$)$_2$), 1.28 (s, 18H, C(C*H*$_3$)$_3$), 1.49 (d, $^3J_{HH}$ = 6.8 Hz, 12H, CH(C*H*$_3$)$_2$), 1.60 (s, 6H, C(C*H*$_3$)$_2$), 3.90 (sept, $^3J_{HH}$ = 6.8 Hz, 4H, C*H*(CH$_3$)$_2$), 6.38 (d, $^4J_{HH}$ = 1.9 Hz, 2H, XA-*o*-C*H*), 6.75 (d, $^4J_{HH}$ = 1.9 Hz, 2H, XA-*p*-C*H*), 7.21-7.32 (m, 6H Ar*H*). ^{13}C{^{1}H} NMR (126 MHz, C_6D_6, 298 K): δ_C = 25.4, 25.9 (CH(*C*H$_3$)$_2$) 27.8 (*C*(CH$_3$)$_2$), 28.4 (*C*H(CH$_3$)$_2$), 31.9 (C(*C*H$_3$)$_3$), 32.3 (b) (P{C(*C*H$_3$)$_3$}$_3$), 35.2 (*C*(CH$_3$)$_3$), 36.5 (dd, $^2J_{CAg}$ = 2.5, 7.0 Hz , P{*C*(CH$_3$)$_3$}$_3$), 37.1 (*C*(CH$_3$)$_2$), 106.4, 110.7, 124.7, 125.6, 132.4, 140.6, 142.6, 144.7, 147.6, 148.3 (Ar-*C*), 237.8 (ddd, $^1J_{C107Ag}$ = 231 Hz, $^1J_{C109Ag}$ = 267 Hz, $^2J_{CP}$ = 81 Hz, *C*Ag). ^{31}P{^{1}H} NMR (162 MHz, C_6D_6, 298 K): δ_P = 76.0 (dd, $^1J_{P\text{-}107Ag}$ = 403, $^1J_{P\text{-}109Ag}$ = 463 Hz).

(NON)Al{(N^{i}Pr)$_2$C}AgP^{t}Bu$_3$ (5-Ag): To a solution of **3-Ag** (80 mg, 0.079 mmol) in benzene (5 mL) was added diisopropylcarbodiimide (0.012 ml, 0.079 mmol) at room temperature. The reaction mixture was stirred for 3 h and volatiles then removed under vacuum. The resulting solid was dissolved in pentane and the solution concentrated to 1 mL. On cooling to − 30 °C colourless crystals of **5-Ag** (as the pentane solvate) suitable for X-ray crystallography formed over the course of 18 h. Yield: 45 mg, 49%. ^{1}H NMR (400 MHz, C_6D_6, 298 K): δ_H = 0.44 (d, $^3J_{HP}$ = 6.6 Hz, 6H, NCH(C*H*$_3$)$_2$), 1.02 (d, $^3J_{HP}$ = 12.9 Hz, 27H, PC(C*H*$_3$)$_3$), 1.19 (d, $^3J_{HH}$ = 6.6 Hz, 6H, CH(C*H*$_3$)$_2$), 1.32 (s, 18H, C(C*H*$_3$)$_3$), 1.39 (d, $^3J_{HH}$ = 6.7 Hz, 6H, CH(C*H*$_3$)$_2$), 1.40 (d, $^3J_{HH}$ = 6.6 Hz, 6H, CH(C*H*$_3$)$_2$), 1.51 (d, $^3J_{HP}$ = 6.5 Hz, 6H, NCH(C*H*$_3$)$_2$), 1.55 (d, $^3J_{HH}$ = 6.8 Hz, 6H, CH(C*H*$_3$)$_2$), 1.70 (s, 3H, C(C*H*$_3$)$_2$), 1.90 (s, 3H, C(C*H*$_3$)$_2$), 3.24 (sept, $^3J_{HH}$ = 6.6 Hz, 1H, NC*H*(CH$_3$)$_2$), 3.74 (sept, $^3J_{HH}$ = 6.8 Hz, 2H, C*H*(CH$_3$)$_2$), 3.91 (sept, $^3J_{HH}$ = 6.6 Hz, 1H, NC*H*(CH$_3$)$_2$), 4.24 (sept, $^3J_{HH}$ = 6.8 Hz, 2H, C*H*(CH$_3$)$_2$), 6.22 (d, $^4J_{HH}$ = 1.8 Hz, 2H, XA-*o*-C*H*), 6.76 (d, $^4J_{HH}$ = 2.0 Hz , 2H, XA-*p*-C*H*), 7.28-7.44 (m, 6H Ar*H*). ^{13}C{^{1}H} NMR (126 MHz, C_6D_6, 298 K): δ_C = 23.5 (C(*C*H$_3$)$_2$), 25.0 (NCH(*C*H$_3$)$_2$), 25.2, 25.9, 26.3, 26.5 (CH(*C*H$_3$)$_2$) 26.8, 27.0 (*C*H(CH$_3$)$_2$), 29.0 (NCH(*C*H$_3$)$_2$), 32.0 (PC(*C*H$_3$)$_3$), 32.1 (C(*C*H$_3$)$_3$), 34.0 (C(*C*H$_3$)$_2$), 35.2 (*C*(CH$_3$)$_3$), 36.8 (dd, $^1J_{CP}$ = 5.9 Hz, $^2J_{CAg}$ = 1.5 Hz, P*C*(CH$_3$)$_3$), 37.0 (*C*(CH$_3$)$_2$), 52.2 (d, $^3J_{CAg}$ = 4.4 Hz, N*C*H(CH$_3$)$_2$), 54.1 (d, $^3J_{CAg}$ = 4.4 Hz, N*C*H(CH$_3$)$_2$), 105.5, 110.8, 124.0, 124.5, 125.5, 128.4, 128.3, 131.3, 139.6, 145.3, 145.5, 147.1, 147.3, 148.7 (Ar*C*), 219.9 (ddd, $^1J_{C107Ag}$ = 182, $^1J_{C109Ag}$ = 210, $^2J_{CP}$ = 63 Hz, *C*Ag). ^{31}P{^{1}H} NMR (162 MHz, C_6D_6, 298 K): δ_P = 73.4 (dd, $^1J_{P\text{-}107Ag}$ = 352, $^1J_{P\text{-}109Ag}$ = 408 Hz). Elemental microanalysis: calc. for $C_{66}H_{103}AgAlN_4OP$: C 69.88 %, H 9.15 %, N 4.94 %; meas.: C 69.66 %, H 9.40 %, N 4.59 %.

(NON)Al{(NCy)$_2$C}CuP^{t}Bu$_3$ (5-Cu): A solution of **3-Cu** (40 mg, 0.041 mmol) and dicyclohexylcarbodiimide (8 mg, 0.041 mmol) in benzene (5 mL) was stirred at room temperature for 16 h. The reaction mixture was concentrated to 0.5 mL and filtered. Colourless crystals of **5-Cu** (as the benzene solvate) suitable for X-ray crystallography formed on standing at room temperature. Yield: 44 mg, 90 %. ^{1}H NMR (400 MHz, C_6D_6, 298 K): δ_H = 1.10 (d, $^3J_{HP}$ = 12.9 Hz, 27H, PC(C*H*$_3$)$_3$), 1.17 (d, $^3J_{HH}$ = 6.6 Hz, 6H, CH(C*H*$_3$)$_2$), 1.31 (s, C(C*H*$_3$)$_3$), 1.43 (d, $^3J_{HH}$ = 6.6 Hz, 6H, CH(C*H*$_3$)$_2$), 1.51 (d, $^3J_{HH}$ = 6.8 Hz, 6H, CH(C*H*$_3$)$_2$), 1.69 (s, 3H, C(C*H*$_3$)$_2$), 1.74 (br, 6H, Cy*H*), 2.00 (s, 3H, C(C*H*$_3$)$_2$), 2.23 (br, Cy*H*), 2.78 (m, 1H, NC*H* Cy), 3.60 (m, 1H, NC*H* Cy), 3.73 (sept, $^3J_{HH}$ = 6.8 Hz, 2H, C*H*(CH$_3$)$_2$), 4.21 (sept, $^3J_{HH}$ = 6.8 Hz, 2H, C*H*(CH$_3$)$_2$), 6.19 (d, $^4J_{HH}$ = 1.8 Hz, 2H, XA-*o*-C*H*), 6.76 (d, $^4J_{HH}$ = 2.0 Hz, 2H, XA-*p*-C*H*), 7.23-7.42 (m, 6H Ar*H*). ^{13}C {^{1}H} NMR (126 MHz, C_6D_6, 298 K): δ_C = 25.2, 25.9, (CH(*C*H$_3$)$_2$) 26.1, 26.3 (CH(*C*H$_3$)$_2$), 26.6, 26.7 (*C*H$_2$ Cy), 26.8, (CH(*C*H$_3$)$_2$), 27.0, (*C*H(CH$_3$)$_2$), 29.2 (*C*H$_2$ Cy) 32.0 (C(*C*H$_3$)$_3$, 32.1, 32.3, 32.4, 32.5, 33.8, 33.9, (*C*H$_2$ Cy), 35.1, (C(*C*H$_3$)$_3$, 35.6, 36.6, 37.0, (*C*(CH$_3$)$_2$), 37.6 (d, $^1J_{CP}$ = 5 Hz, P*C*(CH$_3$)$_3$), 59.6, 62.3 (N*C* Cy), 105.5, 110.9, 123.8, 124.4, 125.4, 128.5, 131.5, 139.8, 145.5, 145.6, 147.0, 147.2, 148.7 (Ar-*C*), 215.5 (d, $^2J_{CP}$ = 58 Hz, *C*-Cu). ^{31}P{^{1}H} NMR (162 MHz, C_6D_6, 298 K): δ = 59.6 (s). Elemental microanalysis: calc. for $C_{72}H_{111}CuAlN_4OP$: C 73.90 %, H 9.56 %, N 4.79 %; meas.: C 72.88 %, H 9.06 %, N 4.84 %.

Preparation of (NON)Al(O$_2$CO)AgP^{t}Bu$_3$ (6-Ag): A solution of **4-Ag** (100 mg, 0.095 mmol) in benzene (2 mL) was degassed and exposed to CO_2 (ca. 1 atm). The reaction mixture was left at room temperature for 18 h, after which time clean conversion to **6-Ag** was observed by ^{31}P NMR spectroscopy. The reaction mixture was heated to 80 °C for 24 h before being cooled to room temperature. Volatiles were removed under vacuum and the product extracted into hexane. Colourless crystals formed on standing at room temperature. Yield: 58 mg, 57 %. Single crystals suitable for X-ray crystallography were grown from warm benzene. ^{1}H NMR (400 MHz, C_7D_8, 298 K): δ_H = 1.05 (br d, $^3J_{HP}$ = 11.2 Hz, 27H, PC(C*H*$_3$)$_3$), 1.25 (d, $^3J_{HH}$ = 6.7 Hz, 12H, CH(C*H*$_3$)$_2$), 1.29 (s, 18H, C(C*H*$_3$)$_3$), 1.49 (d, $^3J_{HH}$ = 6.7 Hz, 12H, CH(C*H*$_3$)$_2$), 1.61 (s, 6H, C(C*H*$_3$)$_2$), 3.93 (sept, $^3J_{HH}$ = 6.6 Hz, 4H, C*H*(CH$_3$)$_2$), 6.31 (d, $^4J_{HH}$ = 1.7 Hz, 2H, XA-*o*-C*H*), 6.73 (d, $^4J_{HH}$ = 1.7 Hz , 2H, XA-*p*-C*H*), 7.20-32 (m, 6H Ar*H*). ^{13}C{^{1}H} NMR (126 MHz, C_6D_6, 298 K): δ_C = 25.5, 26.0 (CH(*C*H$_3$)$_2$) 28.6 (*C*(CH$_3$)$_2$), 30.7 (*C*H(CH$_3$)$_2$), 31.9 (C(*C*H$_3$)$_3$), 32.2 (d, $^2J_{CP}$ = 5.0 Hz, P{C(*C*H$_3$)$_3$}$_3$), 35.2 (*C*(CH$_3$)$_3$), 37.2 (P{*C*(CH$_3$)$_3$}$_3$), 37.1 (*C*(CH$_3$)$_2$), 106.1, 110.5, 123.9, 125.3, 128.3, 132.5, 140.6, 143.7, 143.9, 145.2, 147.8, 148.1 (Ar-*C*); 167.2 (O$_2$-*C*-O). ^{31}P{^{1}H} NMR (162 MHz, C_7D_8, 298 K): δ_P = 74.0 (br). ^{31}P{^{1}H} NMR (162 MHz, C_7D_8, 203 K): δ_P = 74.0 (d, $^1J_{PAg}$ = 486 Hz). Elemental microanalysis: calc. for **6-Ag**·C_6H_6, $C_{66}H_{95}AgAlN_2O_4P$: C 69.15 %, H 8.35 %, N 2.44 %; meas.: C 68.75 %, H 8.75 %, N 2.82 %.

Alternative synthesis of 6-Ag *via* 8: A solution of **3-Ag** (20 mg, 0.020 mmol) in toluene was degassed and exposed to N_2O (ca. 1 atm) at −78 °C. The solution was slowly allowed to reach room temperature, at which point ^{1}H and ^{31}P NMR spectroscopy showed conversion to a single new product. The solution was degassed again to remove N_2O and the reaction vessel back-filled with CO_2 (ca. 1 atm). Crystals of **6-Ag** formed upon standing at room temperature, which gave rise to identical spectroscopic signals to samples derived from **4-Ag**.

(NON)Al(O$_2$CO)CuP^{t}Bu$_3$ (6-Cu): A solution of **3-Cu** (100 mg, 0.103 mmol) in benzene (2 mL) in a J. Young's ampoule was degassed and exposed to CO_2 (ca. 1 atm). After 15 min, volatiles were removed under vacuum and the residue extracted into pentane. Colourless crystals formed on standing at room temperature. Yield: 65 mg, 61%. Crystals suitable for X-

ray crystallography were grown from warm benzene. ^{1}H NMR (400 MHz, C_6D_6, 298 K): δ_H = 0.86 (d, $^{3}J_{HP}$ = 12.7 Hz, 27H, PC(CH_3)$_3$), 1.23 (d, $^{3}J_{HH}$ = 6.9 Hz, 12H, CH(CH_3)$_2$), 1.27 (s, 18H, C(CH_3)$_3$), 1.45 (d, $^{3}J_{HH}$ = 7.0 Hz, 12H, CH(CH_3)$_2$), 1.60 (s, 6H, C(CH_3)$_2$), 3.87 (sept, $^{3}J_{HH}$ = 6.8 Hz, 4H, CH(CH$_3$)$_2$), 6.40 (d, $^{4}J_{HH}$ = 1.9 Hz, 2H, XA-*o*-CH), 6.75 (d, $^{4}J_{HH}$ = 1.9 Hz , 2H, XA-*p*-CH), 7.23-7.32 (m, 6H ArH). ^{13}C{^{1}H} NMR (126 MHz, C_6D_6, 298 K): δ_C = 25.2, 25.9 (CH(CH$_3$)$_2$) 26.7 (C(CH$_3$)$_2$), 28.6 (CH(CH$_3$)$_2$), 31.9 (C(CH$_3$)$_3$), 35.2 (C(CH$_3$)$_3$), 36.5 (d, $^{2}J_{CP}$ = 6.0 Hz, P{C(CH$_3$)$_3$}$_3$), 37.1 (P{C(CH$_3$)$_3$}$_3$), 106.7, 110.6, 124.0, 125.5, 132.4, 140.3, 142.8, 144.7, 147.8, 148.4 (ArC), 170.1 (O$_2$$C$O). ^{31}P{^{1}H} NMR (162 MHz, C_6D_6, 298 K): δ_P = 62.5 (s). Elemental microanalysis: calc. for **6-Cu**·C_6H_6, $C_{66}H_{95}AlCuN_2O_4P$: C 71.93 %, H 8.64 %, N 2.54 %; meas.: C 71.44 %, H 8.14 %, N 2.88 %.

Synthesis of (NON)Al{O(^{i}PrN)CN^{i}Pr}Ag (7-Ag): To solution of **4-Ag** (60 mg, 0.057 mmol) in benzene was added diisopropylcarbodiimide (8.8 µl, 0.057 mmol). The reaction was heated to 80 °C for 15 d, after which time the solvent was removed in vacuo. Crystals suitable for X-ray diffraction were obtained by slow cooling of a pentane solution to −30 °C. In solution **7-Ag** exists as two isomers in an approximate 2:1 ratio. ^{1}H NMR (400 MHz, C_6D_6, 298 K): (major isomer) δ_H = 0.33 (d, $^{3}J_{HH}$ = 7.2 Hz, NCH(CH$_3$)$_2$), 0.91 (d, $^{3}J_{HP}$ = 13.6 Hz, 27H, PC(CH_3)$_3$), 0.99 (s, 6H, C(CH_3)$_2$), 1.27 (s, 18H, C(CH_3)$_3$), 1.45 (d, $^{3}J_{HH}$ = 6.27, 6H, CH(CH$_3$)$_2$), 3.25 (sept, $^{3}J_{HH}$ = 5.98, 1H, CH(CH$_3$)$_2$), 3.54 (sept, $^{3}J_{HH}$ = 6.62, 1H, CH(CH$_3$)$_2$), 3.73 (sept, $^{3}J_{HH}$ = 6.62, 1H, CH(CH$_3$)$_2$), 4.15 (sept, $^{3}J_{HH}$ = 6.51, 2H, CH(CH$_3$)$_2$), 6.29 (d, $^{4}J_{HH}$ = 1.6 Hz, 2H, XA-*o*-CH), 6.75 (d, $^{4}J_{HH}$ = 1.9 Hz , 2H, XA-*p*-CH)(overlap with minor), 7.10-7.40 (m, 6H, ArH); (minor isomer): 0.02 (d, $^{3}J_{HH}$ = 7.2 Hz, NCH(CH$_3$)$_2$), 0.91 (d, $^{3}J_{HP}$ = 13.6 Hz, 27H, PC(CH_3)$_3$), 0.94 (s, 6H, C(CH_3)$_2$), 1.33 (s, 18H, C(CH_3)$_3$), 2.31 (sept, $^{3}J_{HH}$ = 6.55, 1H, CH(CH$_3$)$_2$, 0.65 (sept, $^{3}J_{HH}$ = 6.61, 1H, CH(CH$_3$)$_2$, 3.60, (sept, $^{3}J_{HH}$ = 6.31, 2H, CH(CH$_3$)$_2$, 4.05 (sept, $^{3}J_{HH}$ = 6.52, 1H, CH(CH$_3$)$_2$, 6.24 (d, $^{4}J_{HH}$ = 1.8 Hz, 2H, XA-*o*-CH), 6.75 (d, $^{4}J_{HH}$ = 1.9 Hz, 2H, XA-*p*-CH) (overlap with major isomer), 7.10-7.40 (m, 6H, ArH). ^{13}C{^{1}H} NMR (126 MHz, C_6D_6, 298 K): δ_C = 21.6, (CH$_3$), 22.4 (N-CH(CH$_3$)$_2$) 23.3, 24.5, 24.7, 25.2, 26.3, 26.6, 27.6, 28.0, 28.7, 29.0, 29.1, 29.2 (CH$_3$), 32.0 (CH(CH$_3$)$_2$), 32.5 (PC(CH$_3$)$_3$), 35.2 (CH(CH$_3$)$_2$), 37.0 (d, $^{2}J_{CP}$ = 7.0 Hz, PC(CH$_3$)$_3$), 43.8, 44.0, 47.3, 48.9 (CHCH$_3$), 105.9, 106.8, 110.7, 111.0, 123.6, 123.9, 124.3, 124.5, 125.6, 126.0, 132.3, 140.2, 140.6, 143.5, 144.5, 145.1, 145.3, 147.1, 147.2, 147.8, 148.5, 149.3 (C-Ar) 167.2 (dd, $^{1}J_{AgC}$ = 6.5 Hz, $^{2}J_{PC}$ = 2.3 Hz, C-Ag [minor]), 167.8, (dd, $^{1}J_{AgC}$ = 6.5 Hz, $^{2}J_{PC}$ = 2.3 Hz, C-Ag [major]). ^{31}P{^{1}H} NMR (162 MHz, C_6D_6): δ_P = 80.1 (dd, $^{1}J_{P\text{-}107Ag}$ = 540, $^{1}J_{P\text{-}109Ag}$ = 623 Hz) (major), 78.8 (dd, $^{1}J_{P\text{-}107Ag}$ = 540, $^{1}J_{P\text{-}109Ag}$ = 623 Hz) (minor).

In situ generation of (NON)AlOAgP^{t}Bu$_3$ (8): A solution of **3-Ag** (20 mg, 0.020 mmol) in toluene was degassed and exposed to N_2O (ca. 1 atm) at −78 °C then allowed to reach room temperature. This product was not isolated due to instability on prolonged standing at room temperature. ^{1}H NMR (400 MHz, C_7D_8, 298 K): δ_H = 0.87 (br d, $^{3}J_{HP}$ = 9.7 Hz, 27H, PC(CH_3)$_3$), 1.22 (br, 12H, CH(CH_3)$_2$), 1.24 (s, 18H, C(CH_3)$_3$), 1.48 (d, $^{3}J_{HH}$ = 7.2 Hz, 12H, CH(CH_3)$_2$), 1.59 (s, 6H, C(CH_3)$_2$), 3.93 (sept, $^{3}J_{HH}$ = 6.6 Hz, 4H, CH(CH$_3$)$_2$), 6.27 (d, $^{4}J_{HH}$ = 1.9 Hz, 2H, XA-*o*-CH), 6.67 (d, $^{4}J_{HH}$ = 1.9 Hz , 2H, XA-*p*-CH), 7.10-23 (m, 6H ArH). ^{31}P{^{1}H} NMR (162 MHz, C_7D_8, 298 K): δ_P = 82.0 (br dd, $^{1}J_{P107Ag}$ = 551, $^{1}J_{P109Ag}$ = 640 Hz).

3. Representative ^1H and ^{13}C NMR spectra of new compounds

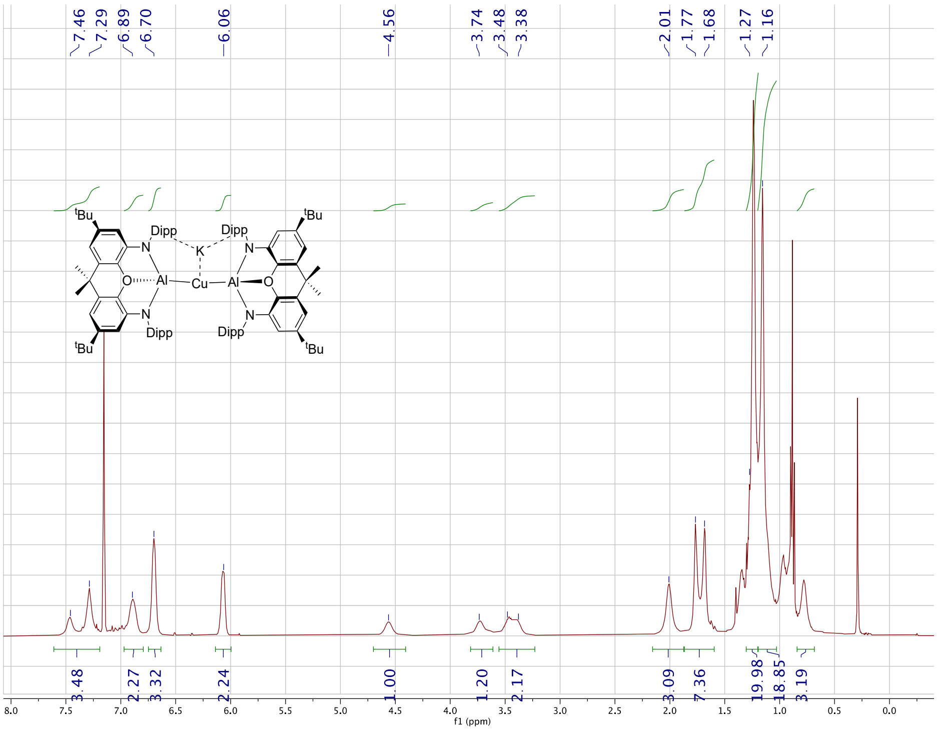

Figure s1: ^1H NMR spectrum of **2** in C_6D_6 at 298 K.

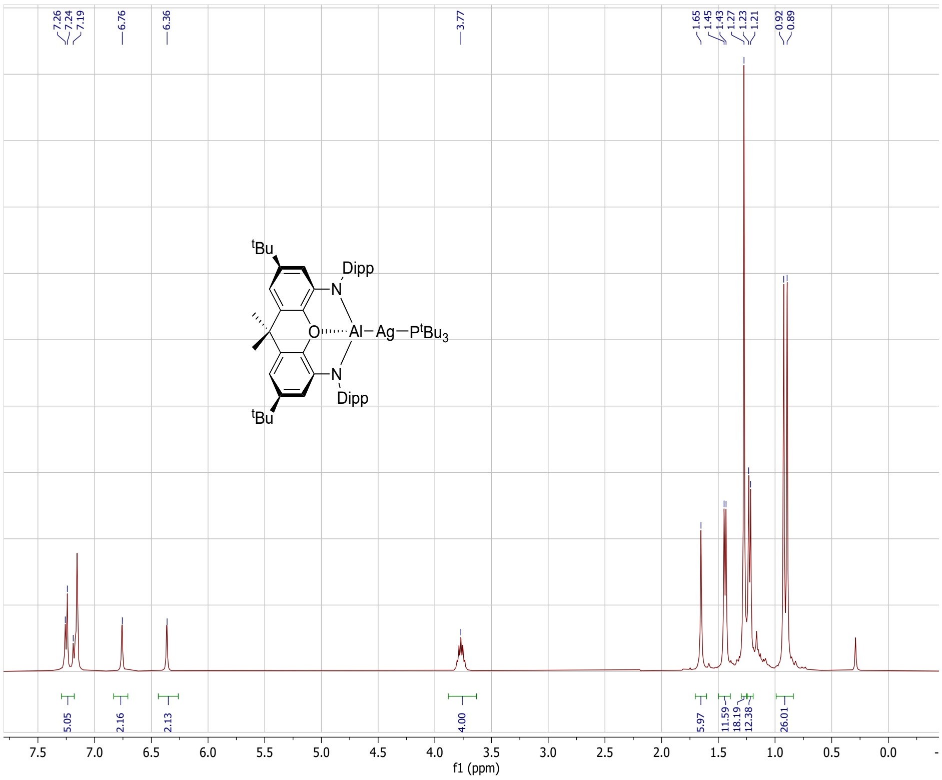

Figure s2: ^1H NMR spectrum of **3-Ag** in C_6D_6 at 298 K.

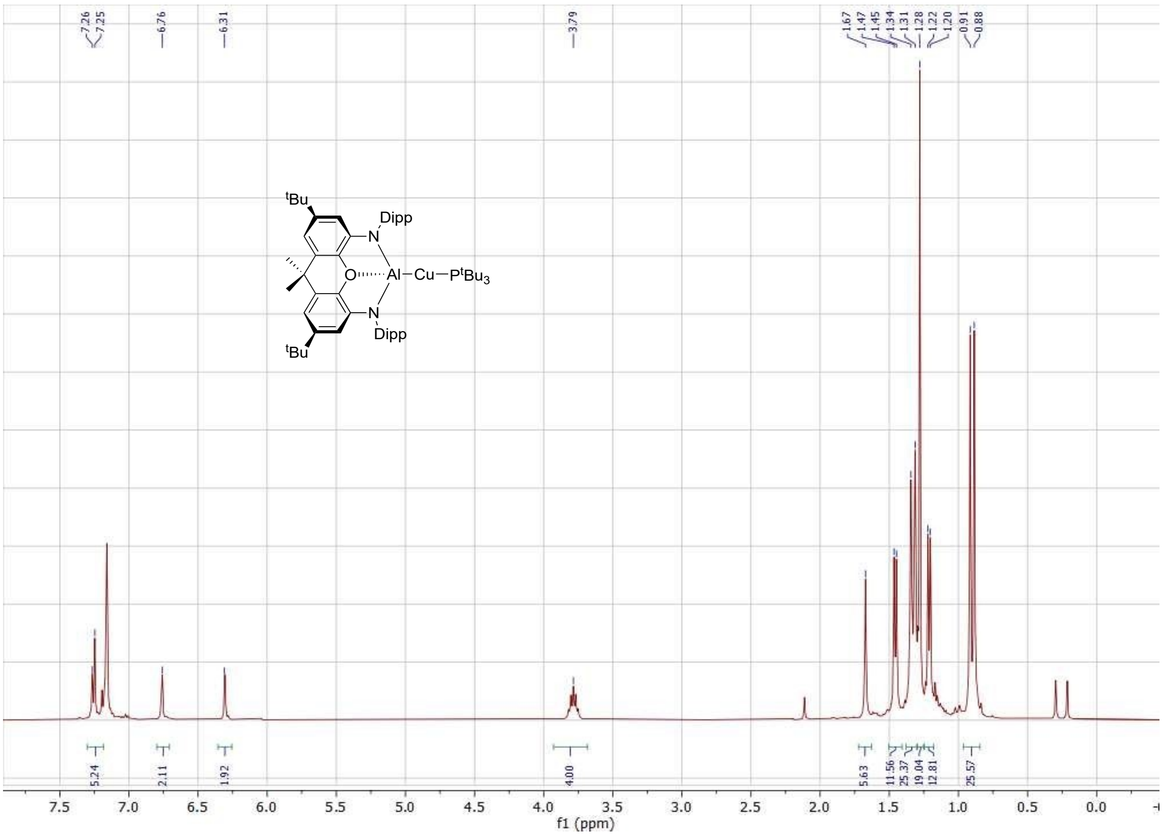

Figure s3: ^{1}H NMR spectrum of **3-Cu** in C_6D_6 at 298 K.

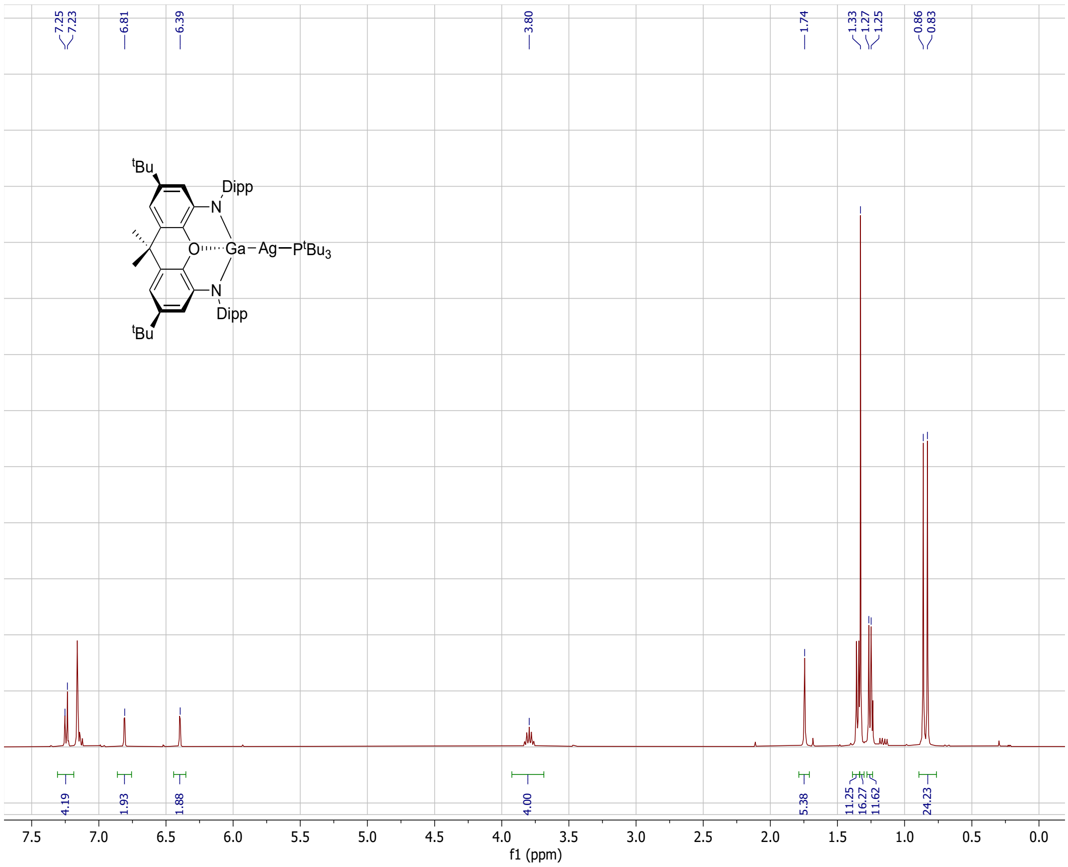

Figure s4: ^{1}H NMR spectrum of **3'-Ag** in C_6D_6 at 298 K.

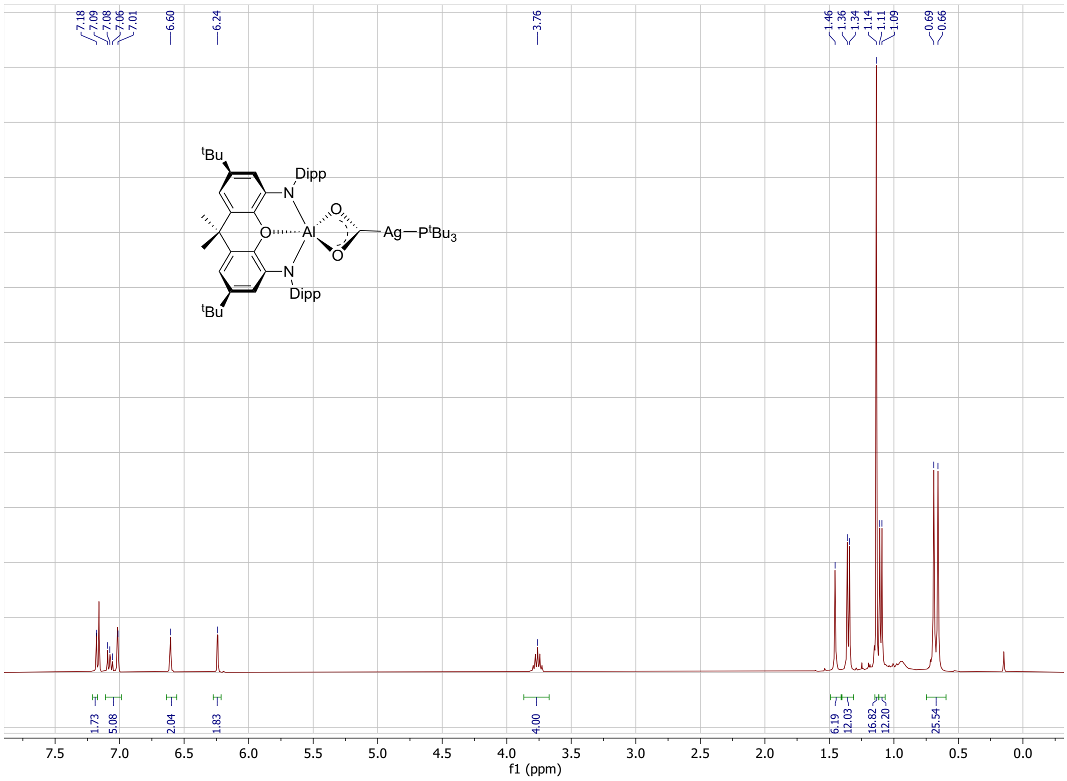

Figure s5: ^{1}H NMR spectrum of **4-Ag** in C_6D_6 at 298 K.

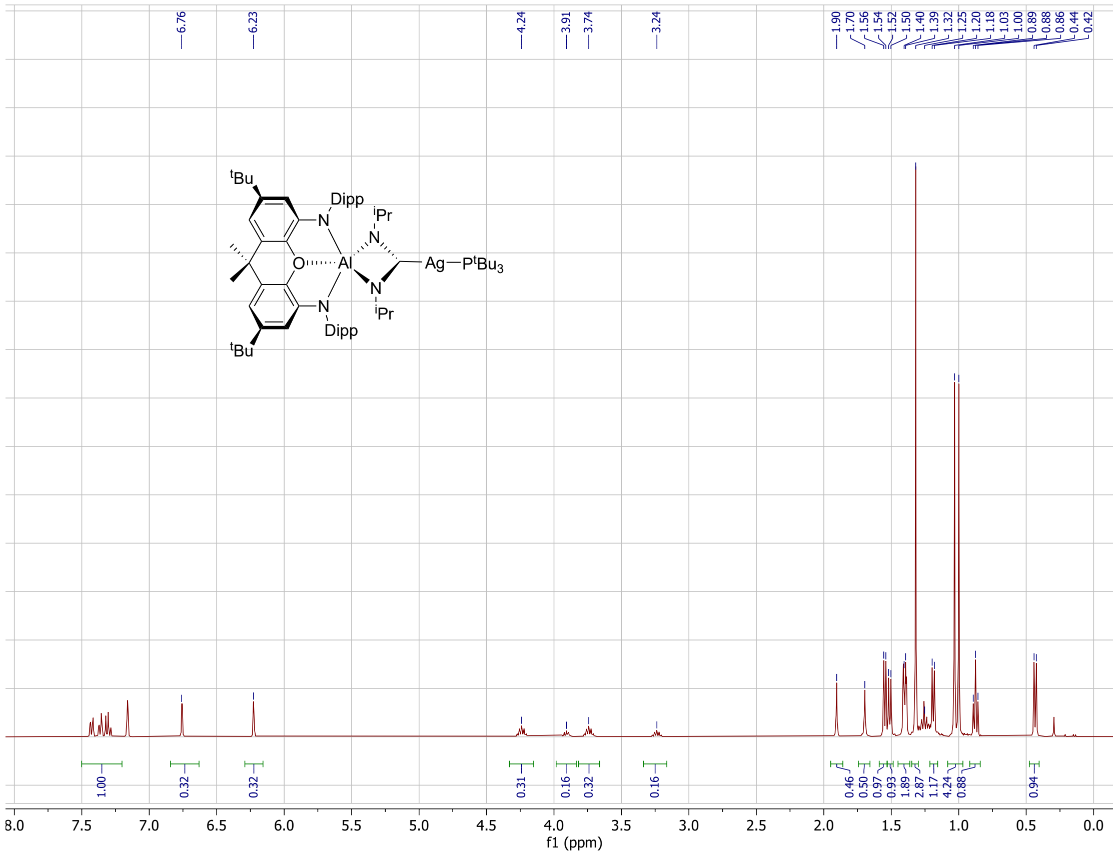

Figure s6: ^{1}H NMR spectrum of **5-Ag** in C_6D_6 at 298 K.

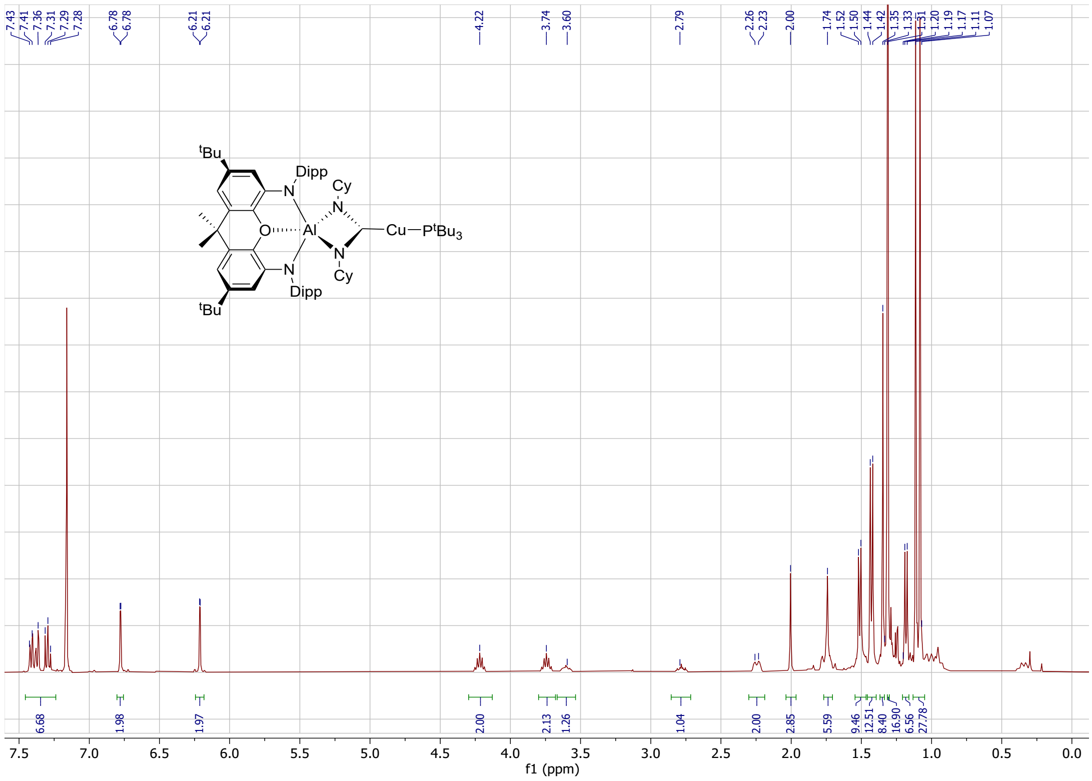

Figure s7: ^{1}H NMR spectrum of **5-Cu** in C_6D_6 at 298 K.

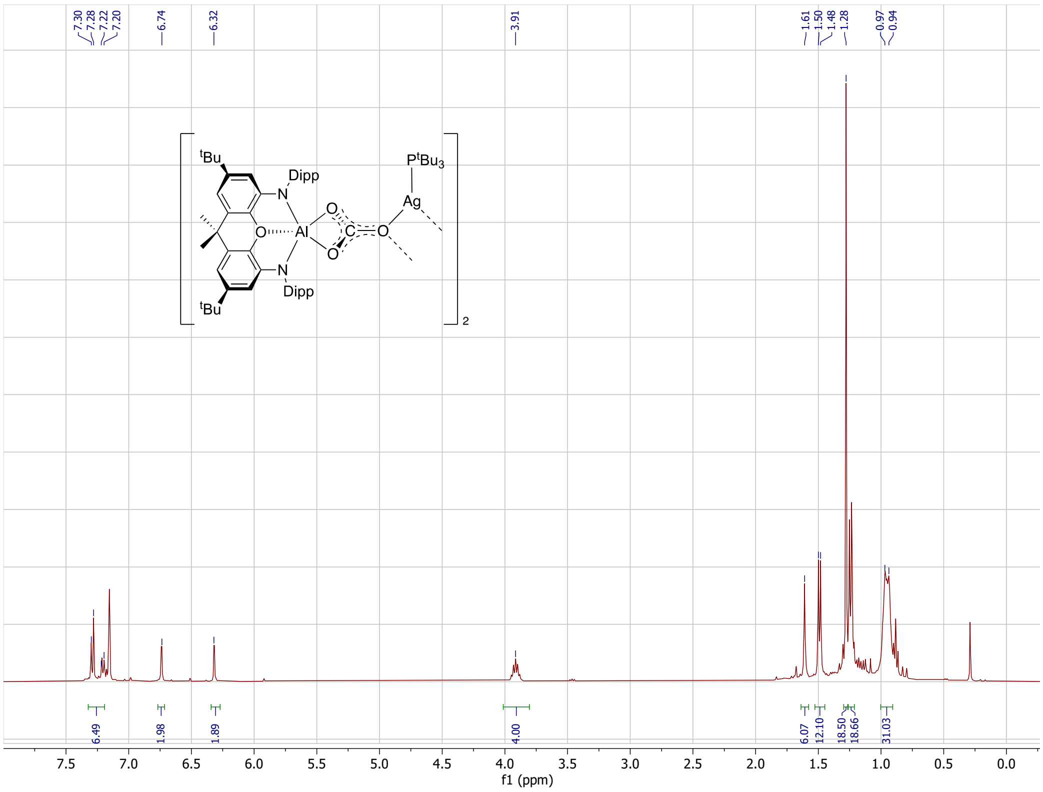

Figure s8: ^{1}H NMR spectrum of **6-Ag** in C_6D_6 at 298 K.

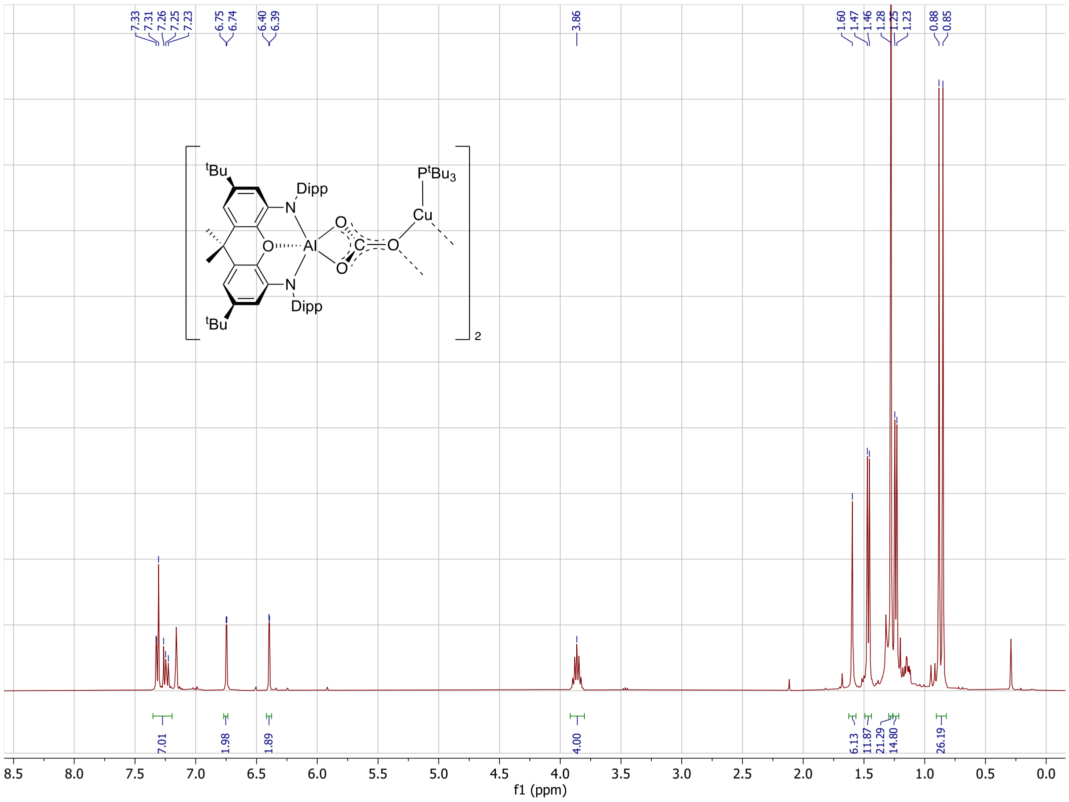

Figure s9: ^{1}H NMR spectrum of **6-Cu** in C_6D_6 at 298 K.

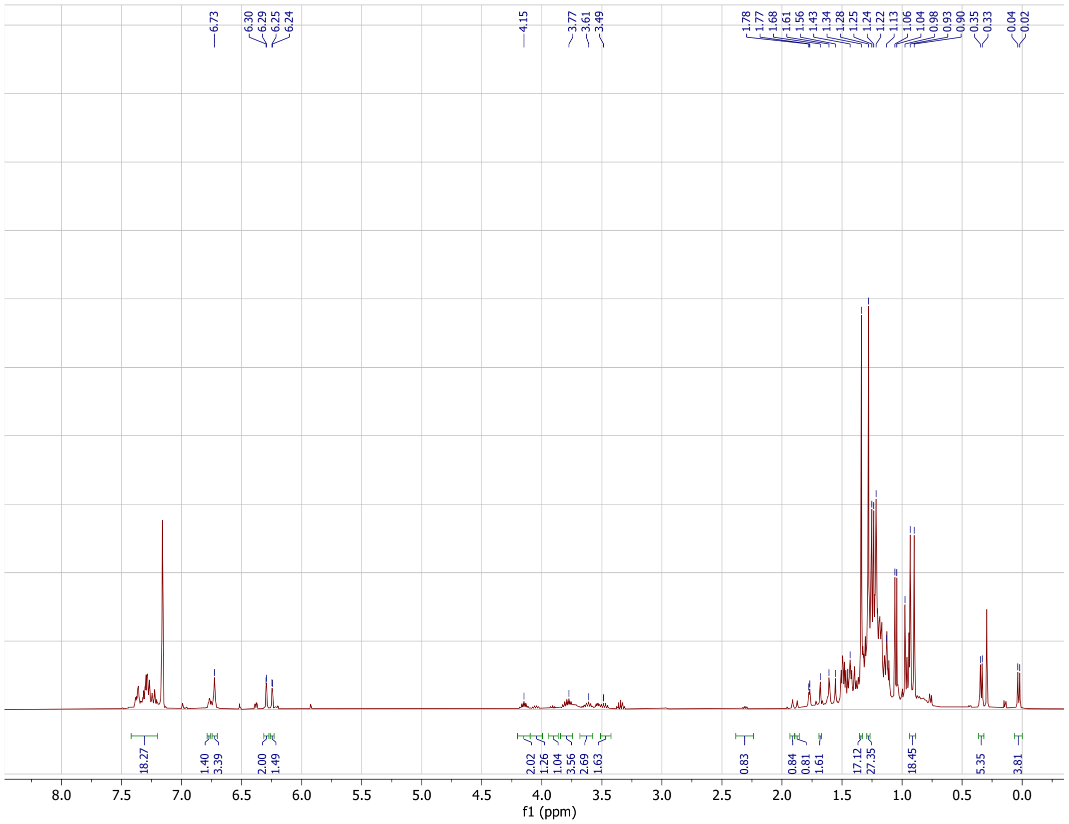

Figure s10: ^{1}H NMR spectrum of **7-Ag** in C_6D_6 at 298 K.

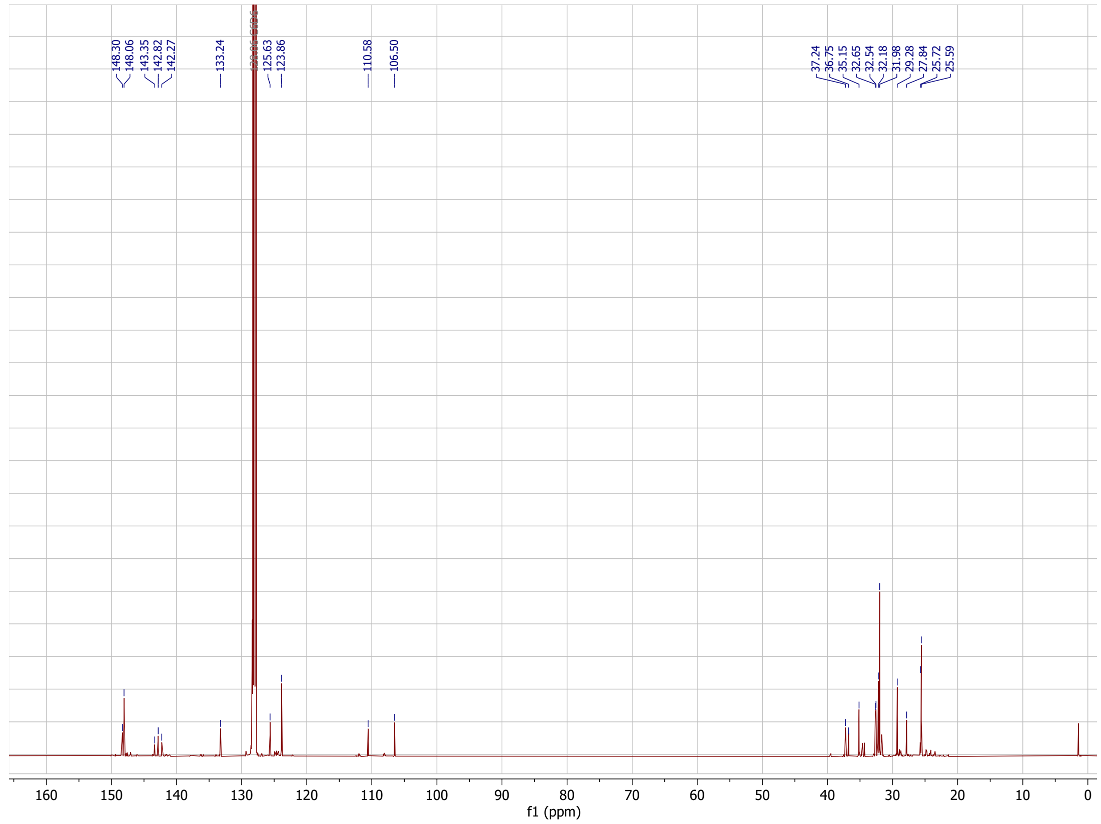

Figure s11: ^{13}C NMR spectrum of **3-Ag** in C_6D_6 at 298 K.

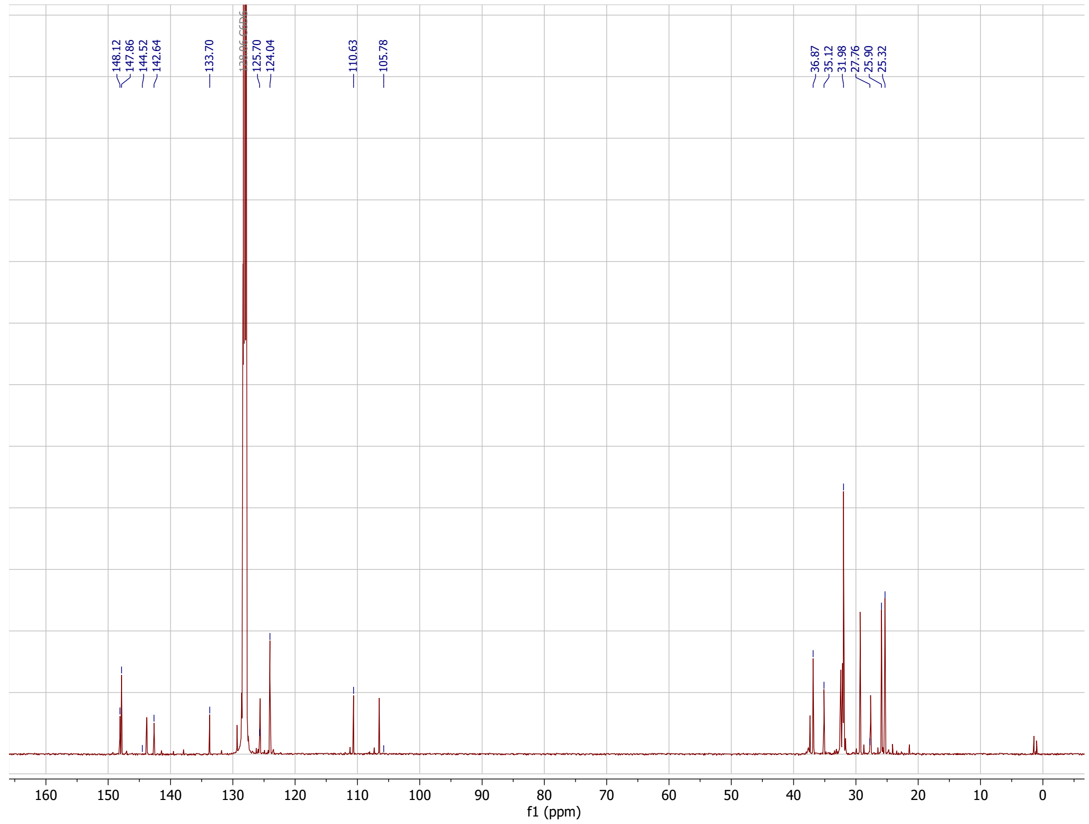

Figure s12: ^{13}C NMR spectrum of **3-Cu** in C_6D_6 at 298 K.

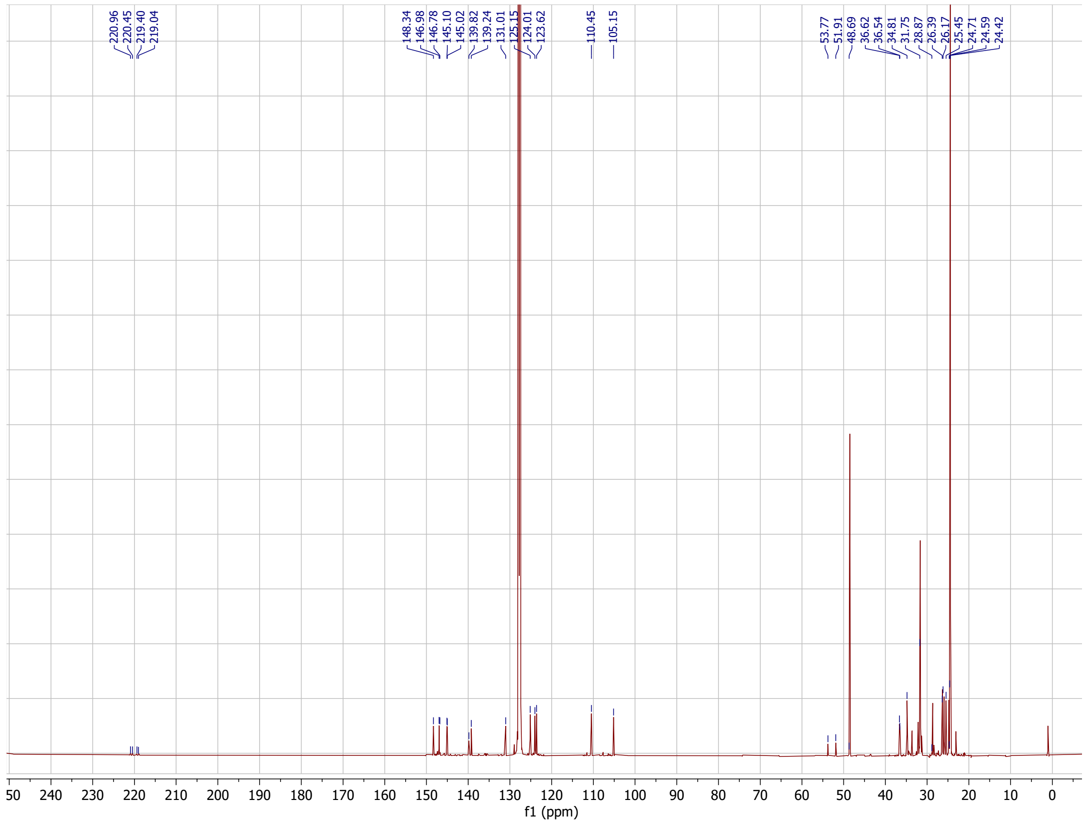

Figure s13: ^{13}C NMR spectrum of **4-Ag** in C_6D_6 at 298 K.

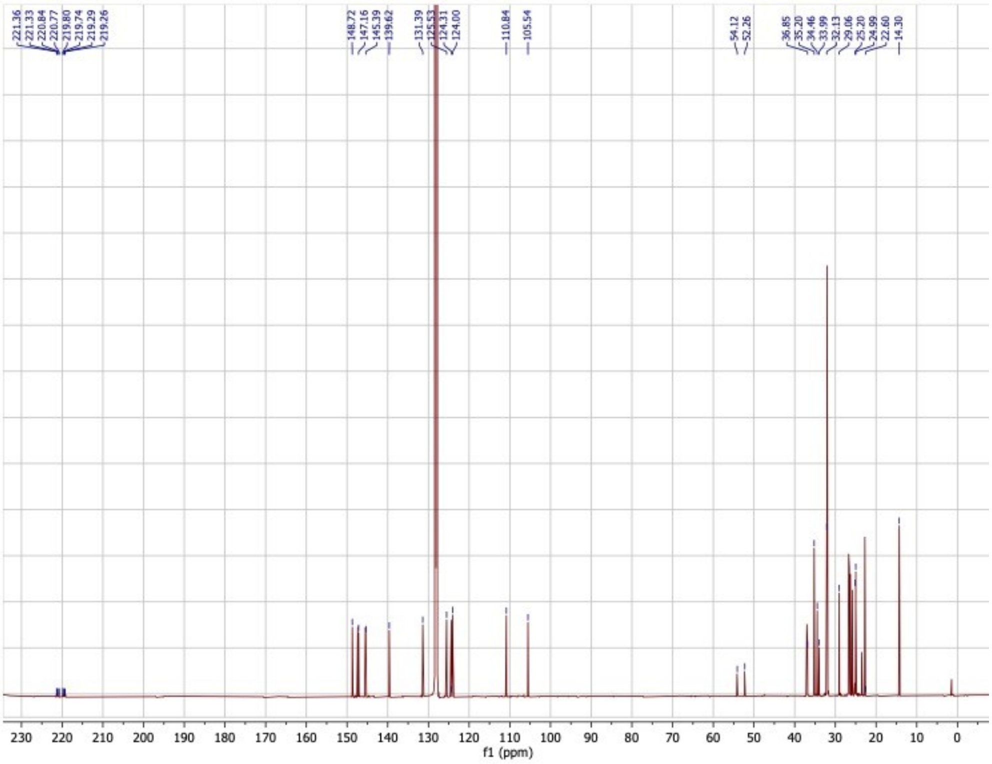

Figure s14: ^{13}C NMR spectrum of **5-Ag** in C_6D_6 at 298 K.

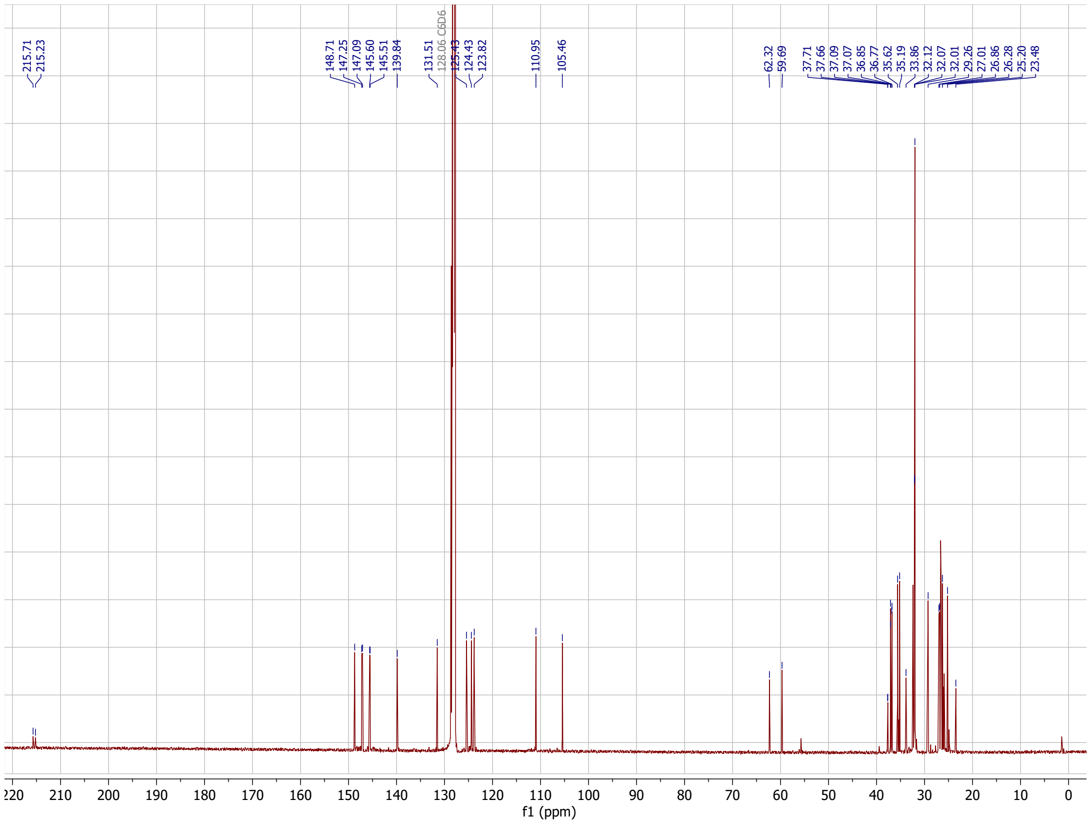

Figure s15: ^{13}C NMR spectrum of **5-Cu** in C_6D_6 at 298 K.

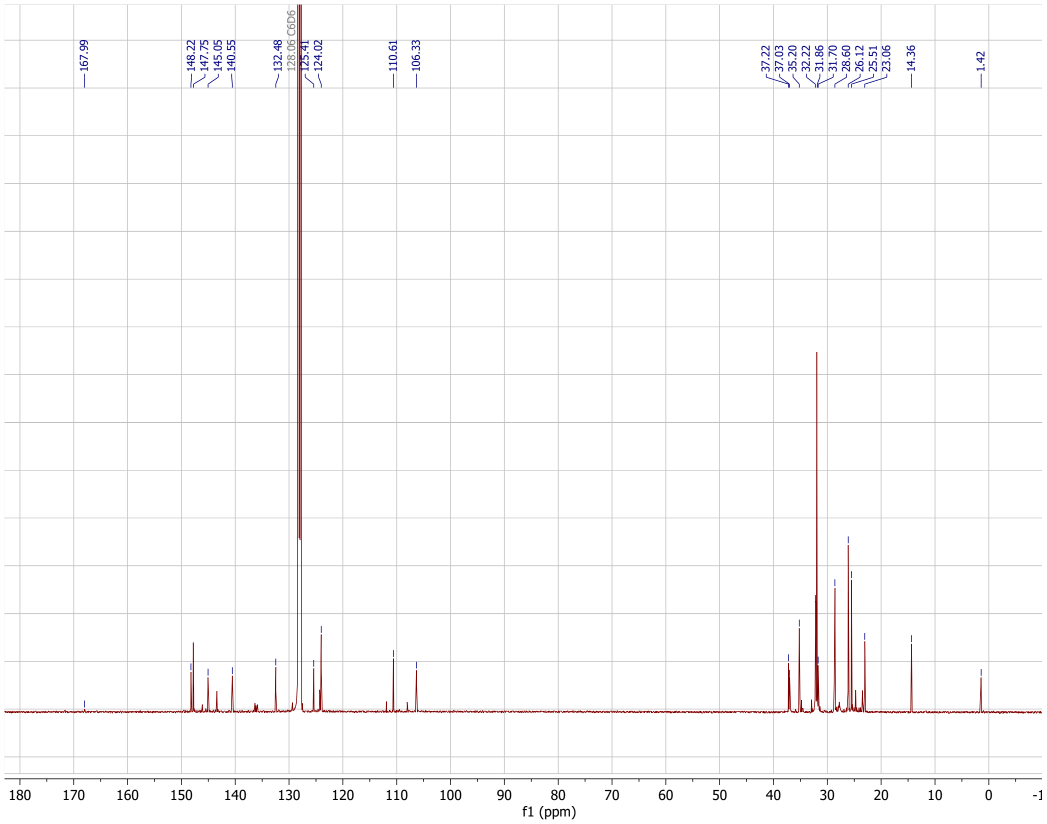

Figure s16: ^{13}C NMR spectrum of **6-Ag** in C_6D_6 at 298 K.

4. X-ray crystallographic studies

Single-crystal X-ray diffraction data were collected using an Oxford Diffraction Supernova dual-source diffractometer equipped with a 135 mm Atlas CCD area detector. Crystals were selected under Paratone-N oil, mounted on Micromount loops and quench-cooled using an Oxford Cryosystems open flow N_2 cooling device.[s3] Data were collected at 150 K using mirror monochromated Cu (λ = 1.5418 Å) or Mo (λ = 0.71073 Å) K_α radiation. All crystallographic data were processed using the CrysAlisPro package, including unit cell parameter refinement and inter-frame scaling (which was carried out using SCALE3 ABSPACK within CrysAlisPro).[s4] Equivalent reflections were merged and diffraction patterns processed with the CrysAlisPro suite. Structures were subsequently solved using ShelXT 2018 and refined on F^2 using the ShelXL 2018 package and XSeed.[s5,s6]

Table s1. Crystallographic and refinement parameters for the structures of compounds **2**·0.5(C_6H_{14}), **3'**·2C_7H_8, **5-Ag**·C_5H_{12}, **5-Cu**·C_6H_6, **6-Ag**·2.5(C_6H_6), **6-Cu**·2.5(C_6H_6), **7-Ag** and $(Ph_3P)_4Cu_4I_2$·0.5(OEt_2).

	**2**·0.5(C_6H_{14})	**3'**·2C_7H_8	**5-Ag**·C_5H_{12}	**5-Cu**·C_6H_6
Formula	$C_{97}H_{131}Al_2CuKN_4O_2$	$C_{73}H_{105}AgGaN_2OP$	$C_{71}H_{115}AgAlN_4OP$	$C_{78}H_{117}AlCuN_4OP$
M_r	1541.65	1235.14	1206.48	1248.24
Cell setting	Triclinic	Triclinic	Orthorhombic	Monoclinic
Space group	*P-1*	*P*-1	$P2_12_12_1$	$P2_1$/n
a/Å	12.8390(3)	12.8040(6)	17.30440(10)	12.6572(4)
b/Å	18.1142(3)	14.3764(5)	18.14880(10)	19.3809(6)
c/Å	22.4774(5)	20.7258(7)	22.24090(10)	30.2695(12)
α/°	97.788(2)	82.027(3)	90	90
β/°	101.490(2)	72.271(3)	90	98.787(4)
γ/°	107.207(2)	70.022(4)	90	90
V/Å^3	4786.76(18)	3412.7(3)	6984.86(6)	7338.2(4)
Z	2	2	4	4
Indep. reflections	19763	17762	14559	15157
R_{int}	0.0370	0.0248	0.0373	0.0779
Parameters	1147	715	791	949
R_1 (all data/$I > 2\sigma(I)$)	0.0686/0.0556	0.0586/0.0407	0.0322/0.0308	0.1236/0.0790
wR_2 (all data/$I > 2\sigma(I)$)	0.1750/0.1600	0.1005/0.0904	0.0813/0.0800	0.2153/0.1856
GooF	1.033	1.023	1.043	1.031
Residual max/min	1.40/–0.46	0.90/–0.65	0.322/–0.40	0.80/–0.42
T/K	150(2)	150(2)	150(2)	150(2)
CCDC Deposition No.	2085387	2085388	2085389	2085390

Table s1 contd.

	**6-Ag**·2.5(C_6H_6)	**6-Cu**·2.5(C_6H_6)	**7-Ag**	$(Ph_3P)_4Cu_4I_2$·0.5(OEt_2)
Formula	$C_{75}H_{104}AgAlN_2O_4P$	$C_{150}H_{202}Al_2Cu_2N_4O_8P_2$	$C_{66}H_{103}AgAlN_4O_2P$	$C_{74}H_{65}P_4Cu_4I_2O_{0.5}$
M_r	1263.42	2432.12	1150.34	1594.10
Cell setting	Triclinic	Triclinic	Monoclinic	Monoclinic
Space group	*P*–1	*P*–1	$P2_1$/c	*C2/c*
a/Å	13.0018(4)	13.0313(4)	14.3910(8)	13.5914(3)
b/Å	15.7611(7)	15.8057(7)	19.5868(6)	19.1763(3)
c/Å	19.2597(10)	18.8852(9)	26.9617(9)	27.3025(5)
α/°	113.433(4)	112.876(4)	90	90
β/°	99.104(3)	98.229(3)	102.038(5)	102.636(2)
γ/°	91.609(3)	92.821(3)	90	90
V/Å^3	3557.5(3)	3522.6(3)	7432.7(5)	6943.6(2)
Z	2	1	4	4
Indep. reflections	14719	14058	15310	7154
R_{int}	0.1058	Twin model	0.0510	0.0231
Parameters	813	847	705	418
R_1 (all data/$I > 2\sigma(I)$)	0.1077/0.0615	0.1523/0.0976	0.1053/0.0920	0.0367/0.0317
wR_2 (all data/$I > 2\sigma(I)$)	0.1768/0.1427	0.3033/0.2468	0.2760/0.2628	0.0894/0.0856
GooF	1.061	1.002	1.028	1.047
Residual max/min	1.07/–1.17	1.33/–0.48	0.87/–0.243	1.27/–0.39
T/K	150(2)	150(2)	150(2)	150(2)
CCDC Deposition No.	2085391	2085392	2085393	2085394

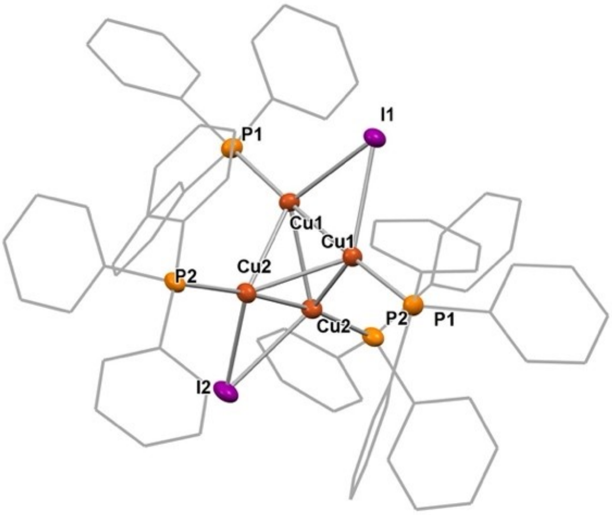

Figure s17: Molecular structure of $(Ph_3P)_4Cu_4I_2$ in the solid state as determined by X-ray crystallography. Hydrogen atoms omitted and phenyl rings shown in wireframe format for clarity; thermal ellipsoids drawn at the 50% probability level.

5. Computational Details

Geometry optimizations were carried out using the Gaussian 16 program[s7] with the BP86[s8,s9] functional and the def2-SVP basis set.[s10-s12] The energetics were further improved by using the larger basis set def2-TZVPP,[s12,s13] and dispersion corrections by Grimme with Becke-Johnson damping D3(BJ).[s14,s15] Solvation effects were taken into account using the SMDmodel,[s16] model using the experimentally used solvent benzene, denoted as the BP86+D3(BJ)/def2-TZVPP (SMD, solvent=benzene) //BP86/def2-SVP level. Intrinsic reaction coordinate (IRC)[s17] calculations were conducted to verify the critical reaction steps. Electronic energies without ZPE corrections are also given for reference in the related schemes. The structures were illustrated using CYLview.[s18]

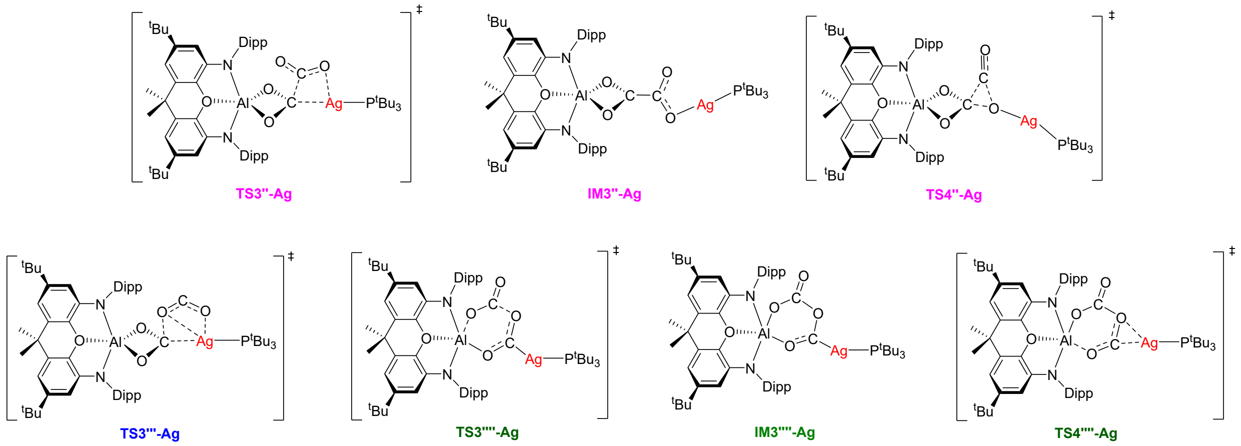

Figure s18: Schematic drawing of the structures not shown in Figure 10.

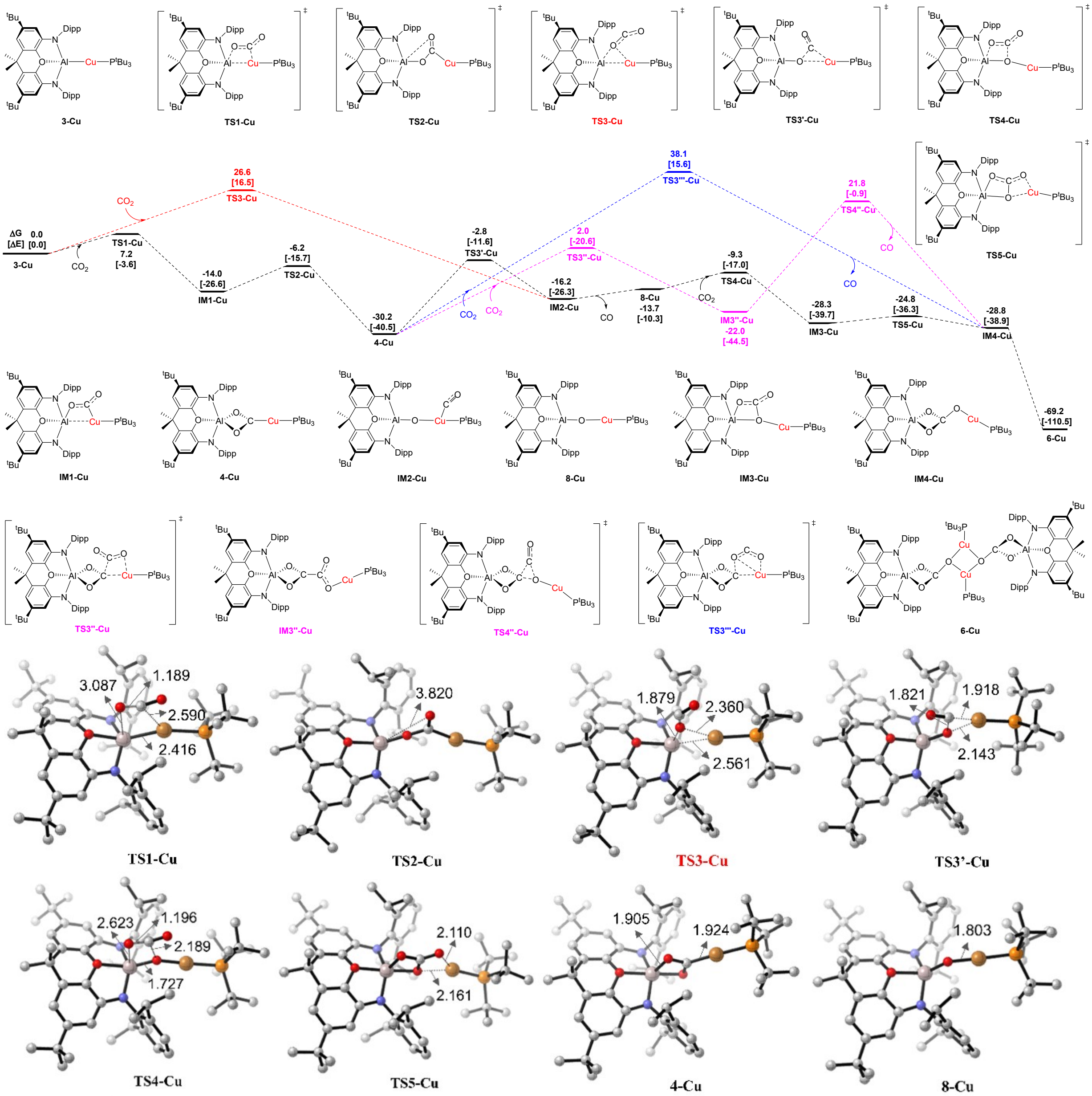

Figure s19: Computed Gibbs energy profiles (in kcal mol^{-1}) for Cu at the BP86+D3(BJ)/def2-TZVPP (SMD, solvent=benzene)//BP86/def2-SVP level (electronic energies are given in brackets for reference). Key bond distances are given in Å for some key intermediates and transition states. Color code, C: grey, N: blue, O: red, P: orange, Al: pink, Cu: brown.

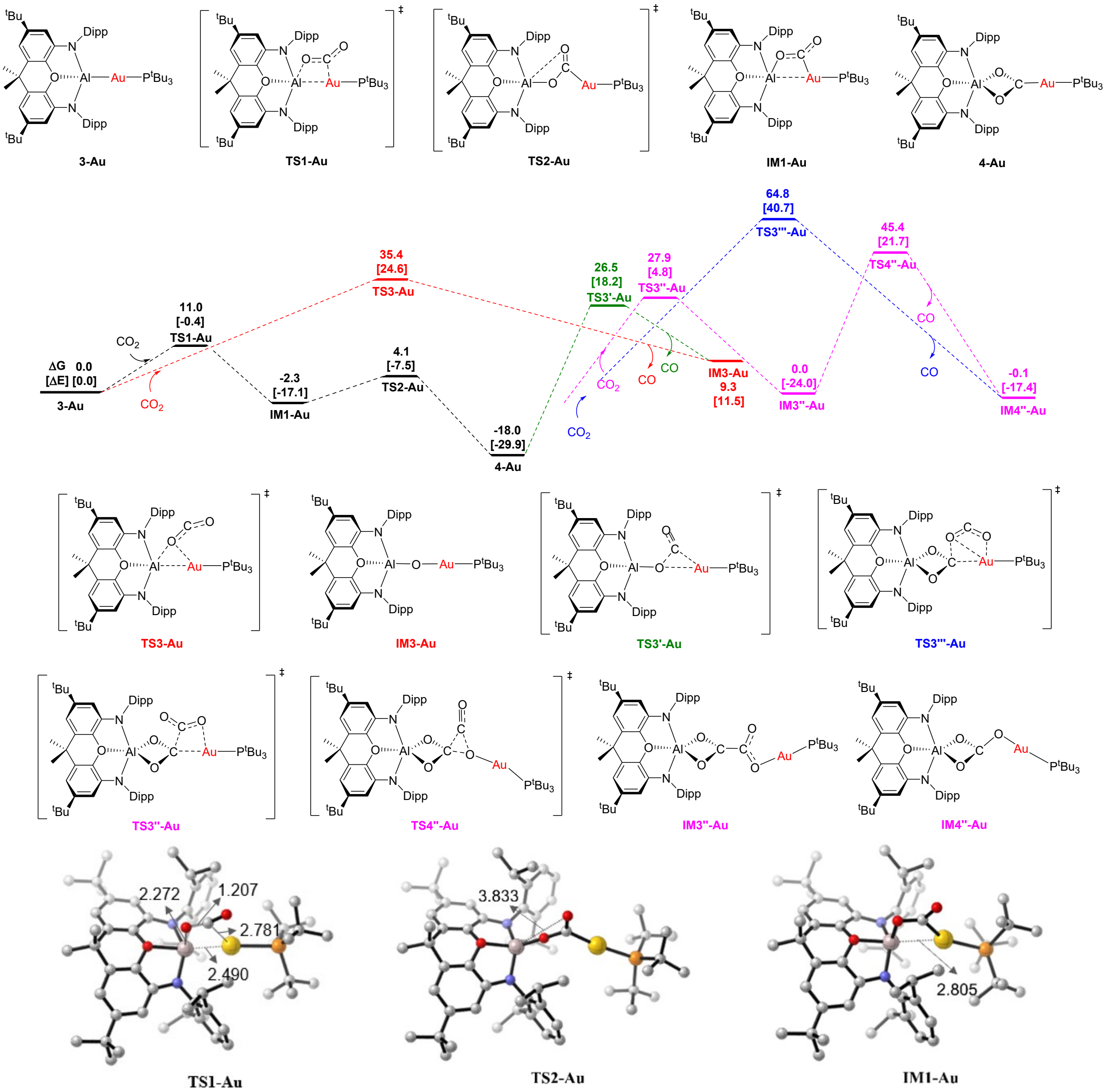

Figure s20: Computed Gibbs energy profiles (in kcal mol^{-1}) for Au at the BP86+D3(BJ)/def2-TZVPP (SMD, solvent=benzene)//BP86/def2-SVP level (electronic energies are given in brackets for reference). Key bond distances are given in Å for some key intermediates and transition states. Color code, C: grey, N: blue, O: red, P: orange, Al: pink, Au: yellow.

Table s2. Coordinates and energies (Hartree) of calculated structures at the BP86+D3(BJ)/def2-TZVPP (SMD, solvent=benzene)//BP86/def2-SVP level.

3-Ag
E= -3219.0047991

P	4.751521	-0.027534	-0.531691
Al	-0.280860	-0.001378	-0.082357
O	-2.201238	0.007935	-0.944138
N	-0.917467	1.815056	0.325729
N	-0.942717	-1.805413	0.345764
C	-2.939243	1.185553	-0.741807
C	-2.210809	2.174607	-0.049260
C	-2.899758	3.391823	0.176818
H	-2.365924	4.205837	0.689441
C	-4.255021	3.552871	-0.206935
C	-4.933827	2.481702	-0.833613
H	-5.990832	2.587924	-1.105609
C	-4.277563	1.259426	-1.113803
C	-4.904237	0.021196	-1.794097
C	-4.292320	-1.223176	-1.111957
C	-4.957070	-2.435182	-0.827484
H	-6.016427	-2.539803	-1.101274
C	-4.289018	-3.514043	-0.193174
C	-2.939237	-3.364193	0.198429
H	-2.411266	-4.174709	0.717479
C	-2.237941	-2.151095	-0.035600
C	-2.951217	-1.161754	-0.737273
C	-5.070812	-4.825437	0.071274
C	-6.299618	-4.522984	0.968015
H	-5.984155	-4.099262	1.944224
H	-6.875269	-5.452644	1.166780
H	-6.990736	-3.796107	0.494553
C	-5.552034	-5.417250	-1.279279
H	-6.215106	-4.715459	-1.825318
H	-6.120377	-6.357916	-1.113621
H	-4.691287	-5.648738	-1.940912
C	-4.209519	-5.890576	0.782804
H	-3.320831	-6.175442	0.182526
H	-4.808544	-6.810750	0.946807
H	-3.855301	-5.542990	1.775109
C	-4.497230	0.017790	-3.298172
H	-3.395275	0.010685	-3.417610
H	-4.891694	0.921888	-3.807484
H	-4.903306	-0.881575	-3.806764
C	-6.439733	0.029782	-1.689854
H	-6.872407	-0.857365	-2.194793
H	-6.862741	0.921489	-2.194874
H	-6.780470	0.031683	-0.634816
C	-4.965733	4.893872	0.106340
C	-4.986500	5.117125	1.641260
H	-3.962990	5.155919	2.066366
H	-5.532355	4.297861	2.154130
H	-5.491521	6.075394	1.890201
C	-4.202361	6.060594	-0.571309
H	-4.171499	5.929686	-1.673200
H	-3.156362	6.136052	-0.210662
H	-4.700608	7.030104	-0.355086
C	-6.423542	4.918055	-0.401508
H	-6.888081	5.898068	-0.164606
H	-7.044436	4.133600	0.078730
H	-6.481491	4.779051	-1.501133
C	-0.028787	2.875248	0.697458
C	0.346297	3.056973	2.065055
C	1.215482	4.117793	2.396454
H	1.505358	4.271525	3.447143
C	1.711148	4.990019	1.416984
H	2.380592	5.818308	1.700379
C	1.352017	4.797912	0.076524
H	1.751485	5.478379	-0.693714
C	0.491521	3.749302	-0.309882
C	0.158197	3.576012	-1.794538
H	-0.556240	2.733077	-1.878285
C	1.408521	3.188658	-2.610943
H	2.176082	3.992204	-2.590555
H	1.144137	3.005275	-3.674387
H	1.866984	2.259966	-2.206377
C	-0.528194	4.822441	-2.391030
H	-1.451517	5.079785	-1.834522
H	-0.809787	4.642679	-3.450380
H	0.141141	5.709456	-2.372581
C	-0.221437	2.155286	3.163251
H	-0.374635	1.153223	2.705001
C	0.722370	1.977538	4.365366
H	1.731075	1.636803	4.052913
H	0.310027	1.224540	5.068014
H	0.844607	2.918527	4.943294
C	-1.608137	2.650611	3.632152
H	-1.529642	3.662282	4.085960
H	-2.033513	1.966729	4.397448
H	-2.327111	2.708939	2.791306
C	-0.074390	-2.871840	0.745965
C	0.424248	-3.787651	-0.234435
C	1.265970	-4.839866	0.181722
H	1.649549	-5.551546	-0.568023
C	1.628444	-4.995807	1.525965
H	2.284467	-5.826601	1.832343
C	1.152047	-4.084926	2.479360
H	1.441792	-4.212126	3.533621
C	0.299744	-3.020258	2.118053
C	-0.258928	-2.083013	3.190623
H	-0.388064	-1.088058	2.709861
C	-1.659840	-2.541908	3.654874
H	-2.369547	-2.608720	2.806731
H	-2.081758	-1.832047	4.398112
H	-1.604359	-3.543515	4.133659
C	0.675693	-1.897486	4.398430
H	0.774057	-2.828137	4.997281
H	0.270807	-1.121959	5.080552
H	1.693955	-1.582556	4.089707
C	0.091632	-3.652560	-1.723182
H	-0.632086	-2.820397	-1.829548
C	-0.579801	-4.919243	-2.293265
H	0.099624	-5.798013	-2.260501
H	-0.867896	-4.763708	-3.354612
H	-1.497976	-5.176345	-1.727182
C	1.341933	-3.269073	-2.541998
H	1.782742	-2.323184	-2.157606
H	1.083459	-3.118254	-3.612060
H	2.121708	-4.059485	-2.491908
C	5.433288	-0.450801	-2.310506
C	6.919384	-0.107629	-2.547100
H	7.594200	-0.636812	-1.846332
H	7.126299	0.977617	-2.472015
H	7.203854	-0.419209	-3.576956
C	5.221078	-1.954342	-2.599671
H	4.172739	-2.270036	-2.426838
H	5.890764	-2.609403	-2.009994
H	5.447958	-2.138283	-3.672704
C	4.546476	0.313108	-3.326142
H	4.837602	0.010938	-4.356323
H	4.649244	1.410996	-3.262323
H	3.473163	0.063075	-3.191063
C	5.367947	-1.378907	0.731041
C	4.467655	-2.626301	0.547698
H	4.608651	-3.133681	-0.422597
H	3.390067	-2.377199	0.648900
H	4.712820	-3.362929	1.343439
C	5.108428	-0.874023	2.169375
H	5.290873	-1.715627	2.872658
H	4.056081	-0.551004	2.309029
H	5.779047	-0.046479	2.470874
C	6.851479	-1.782294	0.597489
H	7.542782	-0.928274	0.733591
H	7.076814	-2.257070	-0.377640
H	7.094370	-2.532085	1.383111
C	5.432921	1.717901	0.014076

C	6.907840	1.735982	0.468105
H	7.604494	1.391477	-0.320871
H	7.081168	1.123064	1.374069
H	7.192871	2.780063	0.726985
C	5.253387	2.721541	-1.147948
H	5.487477	3.739498	-0.766992
H	4.208750	2.745728	-1.517704
H	5.932177	2.530634	-2.001329
C	4.525287	2.227077	1.162117
H	4.820868	3.268774	1.414162
H	4.608025	1.629245	2.086895
H	3.456287	2.250749	0.862953
Ag	2.194945	-0.011030	-0.455316

TS1-Ag

E= -3407.6860356

P	4.740645	-0.037059	-0.094001
Al	-0.376415	-0.009186	-0.305439
O	-2.417216	0.027804	-0.823063
N	-0.904820	1.839721	0.095049
N	-0.970152	-1.837268	0.097027
C	-3.097166	1.217949	-0.559853
C	-2.240514	2.218520	-0.056293
C	-2.862153	3.450692	0.260664
H	-2.237216	4.266270	0.653552
C	-4.263585	3.624811	0.123722
C	-5.055658	2.552349	-0.346133
H	-6.141585	2.673977	-0.437906
C	-4.478017	1.308531	-0.698103
C	-5.235859	0.077429	-1.243720
C	-4.520605	-1.182759	-0.707280
C	-5.140757	-2.408228	-0.363505
H	-6.230075	-2.492309	-0.457249
C	-4.386313	-3.509610	0.101278
C	-2.980220	-3.383495	0.242125
H	-2.384214	-4.221104	0.633556
C	-2.316680	-2.170848	-0.065374
C	-3.137568	-1.139974	-0.567296
C	-5.061098	-4.845717	0.503321
C	-4.828936	-5.100143	2.015868
H	-3.749447	-5.163004	2.262081
H	-5.302749	-6.055516	2.329155
H	-5.265859	-4.283284	2.627421
C	-6.583731	-4.833709	0.247421
H	-7.099260	-4.050367	0.840747
H	-7.021725	-5.810988	0.539679
H	-6.822485	-4.668382	-0.823826
C	-4.447950	-6.012657	-0.311911
H	-4.594194	-5.858572	-1.401386
H	-4.927842	-6.975739	-0.034315
H	-3.359471	-6.119548	-0.128919
C	-5.130468	0.081477	-2.798635
H	-4.072523	0.064428	-3.129204
H	-5.606419	0.993239	-3.216690
H	-5.637481	-0.810229	-3.223379
C	-6.721638	0.101386	-0.839771
H	-7.256192	-0.776893	-1.254264
H	-7.225574	1.000750	-1.247357
H	-6.848308	0.099274	0.261763
C	-4.891865	4.981459	0.532245
C	-4.642702	5.225784	2.043710
H	-3.560358	5.253741	2.283775
H	-5.102145	4.422412	2.656634
H	-5.083714	6.195364	2.361170
C	-4.245549	6.128894	-0.285177
H	-4.404074	5.981673	-1.373899
H	-3.152901	6.199054	-0.109401
H	-4.691320	7.106942	-0.003014
C	-6.415558	5.019826	0.285425
H	-6.819737	6.009978	0.583030
H	-6.952813	4.251788	0.879501
H	-6.665900	4.865732	-0.784841
C	0.028959	2.878619	0.421987
C	0.509199	3.016329	1.763482
C	1.418349	4.054961	2.056190
H	1.791278	4.173603	3.084945
C	1.844800	4.954122	1.068054
H	2.545665	5.765659	1.322474
C	1.369687	4.815288	-0.241857
H	1.706283	5.523198	-1.017091
C	0.468964	3.787387	-0.592460
C	-0.012156	3.694244	-2.043688
H	-0.638494	2.783025	-2.124282
C	1.159723	3.540185	-3.035331
H	1.808465	4.442106	-3.049986
H	0.779567	3.392715	-4.068513
H	1.801365	2.672873	-2.781702
C	-0.890198	4.900757	-2.440743
H	-1.772554	4.997464	-1.778680
H	-1.259757	4.787228	-3.482129
H	-0.316954	5.851802	-2.391778
C	0.012130	2.096127	2.881436
H	-0.170576	1.101326	2.416748
C	1.028019	1.898325	4.019916
H	2.018333	1.571518	3.642928
H	0.662175	1.127088	4.728421
H	1.177859	2.827517	4.610295
C	-1.337762	2.581989	3.456568
H	-1.223551	3.583461	3.925135
H	-1.705970	1.882424	4.237073
H	-2.116955	2.659656	2.673828
C	-0.073976	-2.907857	0.429182
C	0.345462	-3.826151	-0.585290
C	1.216420	-4.879025	-0.233126
H	1.538221	-5.593322	-1.008843
C	1.678914	-5.035000	1.079196
H	2.357264	-5.864984	1.335041
C	1.267742	-4.130316	2.068782
H	1.630113	-4.264129	3.099269
C	0.388790	-3.066379	1.774919
C	-0.093406	-2.138741	2.894102
H	-0.204785	-1.127278	2.442417
C	-1.485563	-2.556382	3.420330
H	-2.245577	-2.577400	2.615971
H	-1.835639	-1.849765	4.202801
H	-1.441306	-3.569899	3.874562
C	0.891173	-2.018875	4.070364
H	0.964326	-2.965176	4.647938
H	0.543689	-1.240285	4.780180
H	1.911811	-1.744638	3.736030
C	-0.130652	-3.720781	-2.037432
H	-0.739669	-2.798040	-2.118575
C	-1.030558	-4.910234	-2.437295
H	-0.475108	-5.871857	-2.390473
H	-1.397581	-4.787484	-3.478520
H	-1.914887	-4.992362	-1.775892
C	1.045173	-3.587984	-3.027065
H	1.701170	-2.731626	-2.773303
H	0.669622	-3.435249	-4.061156
H	1.678530	-4.500846	-3.039468
C	5.526137	1.322745	-1.251583
C	6.989997	1.694283	-0.934646
H	7.678052	0.830330	-1.012567
H	7.107423	2.140193	0.072316
H	7.334906	2.457715	-1.667163
C	5.432150	0.850714	-2.721237
H	4.400672	0.555910	-3.000494
H	6.119218	0.014385	-2.953943
H	5.721882	1.700114	-3.377883
C	4.633627	2.583932	-1.143820
H	4.962773	3.320800	-1.908960
H	4.686997	3.083831	-0.161101
H	3.567506	2.348728	-1.341508
C	5.447926	-1.785821	-0.588959
C	4.665935	-2.254663	-1.840163
H	4.834854	-1.626382	-2.732115
H	3.574805	-2.282663	-1.642479
H	4.985863	-3.289121	-2.094094
C	5.118538	-2.806570	0.524816
H	5.370924	-3.822342	0.149648
H	4.038821	-2.816257	0.780508
H	5.706423	-2.649082	1.449596
C	6.963691	-1.824046	-0.876753
H	7.572559	-1.497205	-0.011666
H	7.246216	-1.205787	-1.751257

H	7.260339	-2.870301	-1.112727
C	5.232884	0.345324	1.753101
C	6.682750	-0.012719	2.142172
H	7.434550	0.526724	1.534075
H	6.889478	-1.097911	2.063720
H	6.852646	0.271757	3.204554
C	4.988805	1.843824	2.044780
H	5.091898	2.006651	3.140018
H	3.966962	2.168396	1.758959
H	5.722920	2.508104	1.549656
C	4.243210	-0.435996	2.651578
H	4.405277	-0.138561	3.710961
H	4.364029	-1.532038	2.593019
H	3.191192	-0.196669	2.389512
C	0.851627	-0.021660	-2.860830
O	1.906829	-0.024586	-3.399563
O	-0.346928	-0.016849	-2.751016
Ag	2.165862	-0.032355	-0.339867

IM1-Ag

E= -3407.7148887

P	4.721495	-0.021487	0.103766
Al	-0.569695	-0.003565	-0.681949
O	-2.551596	0.018549	-0.935610
N	-0.935138	1.801530	-0.075965
N	-0.974002	-1.799964	-0.075277
C	-3.191035	1.208079	-0.570307
C	-2.280612	2.189998	-0.127716
C	-2.867837	3.427941	0.232189
H	-2.209661	4.244853	0.562776
C	-4.275524	3.609754	0.211584
C	-5.115929	2.537245	-0.167862
H	-6.205058	2.664057	-0.154768
C	-4.577465	1.291146	-0.569433
C	-5.379530	0.048989	-1.028198
C	-4.604356	-1.211622	-0.573658
C	-5.169481	-2.447124	-0.176000
H	-6.261066	-2.550899	-0.163871
C	-4.352172	-3.538174	0.201025
C	-2.941103	-3.385982	0.223793
H	-2.300661	-4.217018	0.553938
C	-2.327460	-2.159692	-0.131979
C	-3.216424	-1.158047	-0.573779
C	-4.960029	-4.901196	0.619709
C	-4.566477	-5.215293	2.086583
H	-3.466658	-5.270463	2.217124
H	-4.992043	-6.191652	2.403884
H	-4.948436	-4.434166	2.776627
C	-6.501111	-4.909385	0.525825
H	-6.963001	-4.157394	1.198721
H	-6.890271	-5.904750	0.825875
H	-6.854613	-4.711833	-0.507476
C	-4.416908	-6.018490	-0.307877
H	-4.683097	-5.821372	-1.367291
H	-4.845882	-7.003439	-0.023673
H	-3.313027	-6.105837	-0.248764
C	-5.447763	0.052438	-2.585004
H	-4.433826	0.042092	-3.032697
H	-5.973651	0.960598	-2.946923
H	-5.993363	-0.842761	-2.950013
C	-6.810313	0.063394	-0.458694
H	-7.382165	-0.818977	-0.809796
H	-7.363060	0.959051	-0.806747
H	-6.811128	0.061501	0.650090
C	-4.854639	4.983796	0.635037
C	-4.455242	5.283851	2.103296
H	-3.354556	5.315909	2.234335
H	-4.853419	4.507957	2.790071
H	-4.860729	6.267467	2.424457
C	-4.287778	6.093103	-0.287862
H	-4.556932	5.905529	-1.348250
H	-3.182403	6.157694	-0.227372
H	-4.696662	7.085584	-0.000290
C	-6.395169	5.024834	0.540581
H	-6.763388	6.027185	0.843771
H	-6.873166	4.280575	1.210807
H	-6.752215	4.838143	-0.493516
C	0.018326	2.829102	0.241926
C	0.473266	2.994292	1.589986
C	1.384181	4.034351	1.873497
H	1.737142	4.177899	2.906140
C	1.837390	4.903580	0.870485
H	2.539658	5.716111	1.118055
C	1.386245	4.736484	-0.444315
H	1.741046	5.423472	-1.229706
C	0.479467	3.710581	-0.787253
C	-0.004674	3.608276	-2.236895
H	-0.585813	2.668860	-2.326237
C	1.158263	3.526129	-3.247122
H	1.764583	4.457428	-3.258064
H	0.762846	3.378867	-4.273829
H	1.834308	2.675663	-3.031369
C	-0.947180	4.777757	-2.599926
H	-1.825375	4.820059	-1.925910
H	-1.323022	4.664026	-3.638877
H	-0.419881	5.754591	-2.539264
C	-0.040416	2.101714	2.724739
H	-0.248786	1.104889	2.276366
C	0.977485	1.906803	3.862778
H	1.965336	1.570680	3.490066
H	0.607224	1.146239	4.580405
H	1.134149	2.841170	4.442920
C	-1.371821	2.621339	3.313941
H	-1.231993	3.629494	3.760769
H	-1.732703	1.943062	4.116276
H	-2.167007	2.695611	2.548334
C	-0.046701	-2.850415	0.246352
C	0.399492	-3.740011	-0.782487
C	1.274086	-4.792495	-0.435924
H	1.616225	-5.486053	-1.221174
C	1.707409	-4.977868	0.882278
H	2.383724	-5.811098	1.133139
C	1.271799	-4.098711	1.884301
H	1.613922	-4.254488	2.918748
C	0.393829	-3.031648	1.597230
C	-0.097134	-2.124133	2.730299
H	-0.257823	-1.117403	2.284095
C	-1.455939	-2.589032	3.302309
H	-2.245615	-2.626239	2.528500
H	-1.795705	-1.899565	4.104315
H	-1.364035	-3.604147	3.745750
C	0.914377	-1.977307	3.881333
H	1.020207	-2.918856	4.461373
H	0.569630	-1.202195	4.596103
H	1.920807	-1.686671	3.521029
C	-0.067674	-3.618001	-2.236070
H	-0.618400	-2.660705	-2.327443
C	-1.042790	-4.756034	-2.612599
H	-0.547189	-5.749211	-2.549771
H	-1.403969	-4.627350	-3.655006
H	-1.928828	-4.772623	-1.947698
C	1.107719	-3.568408	-3.233984
H	1.812468	-2.746118	-3.001430
H	0.728662	-3.396202	-4.262949
H	1.679171	-4.521482	-3.248844
C	5.505214	1.241226	-1.162796
C	6.970195	1.625153	-0.865133
H	7.651177	0.752320	-0.857783
H	7.084246	2.163081	0.096462
H	7.326993	2.313807	-1.662531
C	5.409882	0.657094	-2.591766
H	4.374586	0.356091	-2.855383
H	6.092218	-0.198674	-2.758153
H	5.711153	1.453144	-3.307148
C	4.623892	2.514939	-1.155442
H	4.988646	3.200464	-1.951160
H	4.649557	3.071798	-0.202608
H	3.562836	2.282746	-1.383180
C	5.403436	-1.812261	-0.262293
C	4.610171	-2.372274	-1.468687
H	4.735292	-1.788044	-2.396831
H	3.524322	-2.431639	-1.246890
H	4.961504	-3.407449	-1.672439
C	5.085129	-2.743782	0.929292
H	5.328493	-3.785350	0.626463

H	4.008520	-2.731503	1.196484
H	5.686653	-2.521110	1.831748
C	6.917405	-1.873350	-0.557943
H	7.533989	-1.481271	0.274122
H	7.192980	-1.327555	-1.481016
H	7.209520	-2.935625	-0.712211
C	5.208725	0.497105	1.914485
C	6.662214	0.171721	2.319388
H	7.409285	0.674998	1.676113
H	6.872854	-0.915344	2.308925
H	6.832805	0.524099	3.360808
C	4.959765	2.012096	2.093989
H	5.068769	2.257117	3.173067
H	3.935389	2.311885	1.790479
H	5.688612	2.638394	1.544829
C	4.230009	-0.225147	2.869397
H	4.375792	0.164684	3.900490
H	4.377655	-1.318678	2.905673
H	3.176566	-0.031904	2.582385
C	1.119506	-0.016766	-2.328085
O	1.964100	-0.023419	-3.214595
O	-0.181018	-0.009665	-2.485831
Ag	2.260921	-0.015359	-0.482425

TS2-Ag

E= -3407.6966964

P	-5.720365	-0.189580	0.071783
Al	1.060229	-0.117823	-0.511431
O	2.984987	0.186471	-0.983048
N	1.172937	1.721543	-0.042669
N	1.739736	-1.804102	0.031435
C	3.451448	1.479391	-0.673123
C	2.431251	2.317384	-0.179951
C	2.831345	3.639748	0.125738
H	2.067986	4.349463	0.476850
C	4.188384	4.038633	0.015194
C	5.165284	3.095775	-0.383516
H	6.221882	3.386347	-0.423277
C	4.808416	1.771480	-0.732453
C	5.772850	0.640576	-1.161292
C	5.217268	-0.680648	-0.577231
C	5.975545	-1.767716	-0.080975
H	7.069562	-1.692411	-0.081168
C	5.349538	-2.936052	0.415482
C	3.933707	-3.015291	0.447284
H	3.432357	-3.907905	0.849837
C	3.131583	-1.939946	-0.001045
C	3.838410	-0.847388	-0.542412
C	6.173723	-4.128930	0.962368
C	5.814341	-5.415513	0.175743
H	4.739039	-5.670883	0.266064
H	6.396354	-6.280762	0.559411
H	6.043906	-5.300018	-0.904005
C	7.695053	-3.898962	0.834099
H	8.005931	-3.760858	-0.222364
H	8.242338	-4.780138	1.229133
H	8.033003	-3.014211	1.412606
C	5.842263	-4.334280	2.463794
H	6.094370	-3.428487	3.053933
H	6.422315	-5.186842	2.878046
H	4.766440	-4.550760	2.623492
C	7.209594	0.911429	-0.679661
H	7.265334	0.993294	0.424613
H	7.600646	1.850809	-1.119567
H	7.891334	0.099928	-1.004567
C	5.766655	0.538958	-2.716095
H	6.424734	-0.289423	-3.051745
H	6.131119	1.485901	-3.166124
H	4.747158	0.345237	-3.105393
C	4.563247	5.504607	0.349043
C	3.816021	6.454728	-0.622734
H	2.715348	6.347002	-0.540360
H	4.096304	6.247083	-1.676515
H	4.068074	7.514453	-0.402804
C	4.151226	5.833617	1.807183
H	4.667601	5.165560	2.527778
H	3.059453	5.722186	1.965943
H	4.419020	6.882129	2.059230
C	6.077982	5.768839	0.209475
H	6.298032	6.829059	0.453444
H	6.438203	5.585126	-0.823989
H	6.675342	5.138981	0.901036
C	0.077663	2.579946	0.321749
C	-0.648610	3.272236	-0.696686
C	-1.676799	4.156007	-0.306964
H	-2.236763	4.702416	-1.082667
C	-1.996156	4.357844	1.042972
H	-2.794321	5.063835	1.324527
C	-1.294464	3.656440	2.032458
H	-1.552093	3.813481	3.092342
C	-0.256734	2.760404	1.698490
C	0.492877	2.032719	2.816718
H	1.084840	1.225267	2.334961
C	-0.461807	1.374374	3.831948
H	0.110454	0.763334	4.561320
H	-1.198899	0.713638	3.332306
H	-1.028411	2.129726	4.417224
C	1.491971	2.962865	3.539641
H	2.235466	3.387732	2.836888
H	2.046613	2.408030	4.326181
H	0.963508	3.807979	4.031705
C	-0.324415	3.109623	-2.183385
H	0.301264	2.198834	-2.280555
C	-1.582077	2.893507	-3.047662
H	-2.195434	2.052460	-2.665325
H	-1.292648	2.647607	-4.090460
H	-2.219381	3.803096	-3.089585
C	0.501808	4.303586	-2.713415
H	-0.075517	5.250744	-2.638846
H	0.763861	4.152792	-3.782278
H	1.445402	4.434818	-2.146702
C	0.948378	-3.000975	0.138825
C	0.237994	-3.281695	1.345209
C	-0.528344	-4.463391	1.418806
H	-1.079287	-4.696761	2.342309
C	-0.598816	-5.354369	0.338656
H	-1.199975	-6.274474	0.418759
C	0.090323	-5.062762	-0.844357
H	0.018638	-5.756551	-1.697761
C	0.865108	-3.891450	-0.976682
C	1.578826	-3.631476	-2.307473
H	2.108783	-2.661274	-2.220539
C	2.646057	-4.710527	-2.595175
H	3.386839	-4.785032	-1.773735
H	3.196848	-4.471974	-3.529759
H	2.183547	-5.712041	-2.730010
C	0.591065	-3.505002	-3.487480
H	0.003167	-4.437511	-3.627894
H	1.150645	-3.326400	-4.430978
H	-0.119179	-2.663892	-3.349736
C	0.327935	-2.343605	2.552261
H	0.397663	-1.306530	2.152437
C	1.615564	-2.595252	3.368672
H	1.617652	-3.625764	3.784705
H	1.689870	-1.885234	4.220107
H	2.524849	-2.476985	2.747162
C	-0.906487	-2.399988	3.469055
H	-1.847148	-2.238574	2.903119
H	-0.838568	-1.618674	4.252984
H	-0.988580	-3.375143	3.994677
C	-5.699385	-0.227622	2.018168
C	-7.006069	0.232902	2.697411
H	-7.879629	-0.381353	2.404493
H	-7.243624	1.294006	2.486608
H	-6.892907	0.140938	3.800452
C	-5.363623	-1.661914	2.487427
H	-4.441624	-2.049986	2.007411
H	-6.186017	-2.381892	2.312411
H	-5.179886	-1.636704	3.583615
C	-4.521326	0.665173	2.482221
H	-4.396899	0.548776	3.581137
H	-4.673035	1.739682	2.279094
H	-3.569539	0.362515	1.997352
C	-6.737337	-1.709670	-0.604931
C	-5.825638	-2.960392	-0.516984

H	-5.606210	-3.275932	0.518206
H	-4.858325	-2.796299	-1.037573
H	-6.339837	-3.808836	-1.019522
C	-7.018576	-1.492239	-2.109970
H	-7.440615	-2.433355	-2.524938
H	-6.091158	-1.270241	-2.677343
H	-7.758156	-0.691883	-2.304689
C	-8.068113	-1.979743	0.127411
H	-8.762196	-1.117926	0.086084
H	-7.919750	-2.253187	1.190555
H	-8.580625	-2.839848	-0.357772
C	-6.538954	1.470033	-0.540777
C	-8.077604	1.516764	-0.431380
H	-8.438588	1.379833	0.606630
H	-8.572449	0.760550	-1.071842
H	-8.434293	2.512907	-0.775486
C	-5.938221	2.651636	0.255673
H	-6.284382	3.597980	-0.214353
H	-4.829088	2.657006	0.226107
H	-6.267129	2.676689	1.312398
C	-6.112834	1.684795	-2.015594
H	-6.458021	2.690591	-2.341317
H	-6.546793	0.946359	-2.712421
H	-5.008774	1.652505	-2.129288
C	-1.651332	-0.595057	-1.888542
O	-1.670824	-0.940630	-3.064188
O	-0.451150	-0.383913	-1.278965
Ag	-3.451693	-0.347933	-0.831696

4-Ag

E= -3407.7340109

P	5.874208	-0.026716	-0.481459
Al	-0.910044	-0.005159	0.104009
O	-2.624991	0.010620	-0.967796
N	-1.458125	1.816535	0.398542
N	-1.496953	-1.813715	0.407931
C	-3.365534	1.199910	-0.875452
C	-2.689930	2.194968	-0.137015
C	-3.375590	3.427884	-0.018086
H	-2.884217	4.247839	0.526386
C	-4.680201	3.597582	-0.548894
C	-5.312807	2.519125	-1.210294
H	-6.332074	2.636631	-1.597286
C	-4.655889	1.276665	-1.384927
C	-5.229892	0.034108	-2.105808
C	-4.679057	-1.220680	-1.388416
C	-5.353469	-2.448959	-1.212711
H	-6.376527	-2.556371	-1.599933
C	-4.738822	-3.542202	-0.549743
C	-3.437787	-3.395322	-0.014878
H	-2.956880	-4.217543	0.530617
C	-2.731991	-2.169595	-0.135019
C	-3.385459	-1.166716	-0.876810
C	-5.525255	-4.870484	-0.413353
C	-6.838770	-4.614976	0.370765
H	-6.624530	-4.227283	1.388534
H	-7.419117	-5.556877	0.476903
H	-7.489819	-3.875944	-0.139311
C	-5.865794	-5.413526	-1.825793
H	-6.482975	-4.697872	-2.406516
H	-6.434299	-6.365725	-1.751677
H	-4.942115	-5.610196	-2.409180
C	-4.722598	-5.955179	0.336469
H	-3.775419	-6.205570	-0.184382
H	-5.321899	-6.887320	0.403673
H	-4.472398	-5.645841	1.372173
C	-4.713138	0.031412	-3.575878
H	-3.605341	0.020486	-3.613186
H	-5.065517	0.937824	-4.111310
H	-5.083440	-0.865890	-4.114563
C	-6.769686	0.047833	-2.113275
H	-7.168011	-0.836973	-2.649445
H	-7.152473	0.941241	-2.646419
H	-7.185136	0.049715	-1.085342
C	-5.387720	4.963592	-0.361303
C	-5.551096	5.256583	1.152973
H	-4.573084	5.297518	1.673980
H	-6.161826	4.471407	1.645645
H	-6.056679	6.233944	1.309322
C	-4.535623	6.083619	-1.011848
H	-4.403027	5.902423	-2.098986
H	-3.526887	6.153603	-0.556591
H	-5.028818	7.071610	-0.886290
C	-6.789382	4.992402	-1.008041
H	-7.253335	5.989321	-0.855432
H	-7.469718	4.238975	-0.559598
H	-6.745188	4.809881	-2.101926
C	-0.627084	2.860348	0.931390
C	-0.547148	3.066936	2.342484
C	0.249224	4.123845	2.830033
H	0.313200	4.295234	3.915816
C	0.957414	4.966017	1.961744
H	1.565579	5.792351	2.364398
C	0.891835	4.743949	0.580526
H	1.459022	5.399436	-0.100925
C	0.116827	3.696108	0.040043
C	0.099871	3.497759	-1.478454
H	-0.461064	2.564036	-1.682818
C	1.519064	3.305764	-2.053221
H	2.142433	4.217505	-1.928043
H	1.468614	3.084987	-3.140571
H	2.040781	2.458562	-1.561211
C	-0.627709	4.652404	-2.201164
H	-1.669066	4.764901	-1.838987
H	-0.667340	4.466036	-3.295720
H	-0.104449	5.620802	-2.045197
C	-1.329640	2.189351	3.320945
H	-1.495838	1.215133	2.812618
C	-0.564417	1.909186	4.627054
H	0.443887	1.492256	4.428285
H	-1.121238	1.178684	5.249216
H	-0.442195	2.825249	5.243782
C	-2.722724	2.786075	3.623107
H	-2.627037	3.776811	4.118233
H	-3.298036	2.121469	4.302734
H	-3.317896	2.924142	2.698595
C	-0.692859	-2.869262	0.958942
C	0.046829	-3.725602	0.083955
C	0.800821	-4.778948	0.643051
H	1.365855	-5.449239	-0.025604
C	0.847874	-4.988626	2.026956
H	1.440379	-5.819110	2.444119
C	0.139606	-4.130193	2.879037
H	0.186298	-4.293977	3.966918
C	-0.635947	-3.066782	2.372500
C	-1.424499	-2.175265	3.333392
H	-1.579585	-1.205089	2.813966
C	-2.824234	-2.762455	3.624395
H	-3.408116	-2.907561	2.693700
H	-3.404776	-2.088045	4.289784
H	-2.739102	-3.748439	4.130846
C	-0.673895	-1.887160	4.646197
H	-0.561654	-2.798743	5.271391
H	-1.236374	-1.150599	5.256290
H	0.337857	-1.474580	4.456406
C	0.042998	-3.546236	-1.436991
H	-0.498513	-2.604524	-1.656624
C	-0.704115	-4.696367	-2.147588
H	-0.200418	-5.672457	-1.976100
H	-0.736212	-4.524050	-3.244694
H	-1.748992	-4.782980	-1.787540
C	1.468385	-3.390104	-2.007039
H	2.004552	-2.547261	-1.523242
H	1.427238	-3.182859	-3.097477
H	2.073331	-4.311898	-1.866636
C	6.451131	-0.474449	-2.286385
C	7.922795	-0.129245	-2.597598
H	8.634449	-0.641170	-1.920982
H	8.124407	0.958914	-2.552380
H	8.159116	-0.457552	-3.633737
C	6.223175	-1.984429	-2.529515
H	5.181708	-2.291090	-2.301630
H	6.918411	-2.628543	-1.957662
H	6.398367	-2.192889	-3.607290
C	5.514685	0.267560	-3.273861

H	5.749008	-0.071087	-4.306726
H	5.631562	1.365358	-3.253092
H	4.447187	0.033661	-3.076749
C	6.551360	-1.344088	0.782770
C	5.658733	-2.606284	0.671485
H	5.756136	-3.133415	-0.293759
H	4.585824	-2.363216	0.822925
H	5.956008	-3.320596	1.469998
C	6.355657	-0.806483	2.219334
H	6.585117	-1.628061	2.932015
H	5.307709	-0.493521	2.406356
H	7.030253	0.035531	2.466294
C	8.031771	-1.729663	0.580420
H	8.715023	-0.861492	0.655264
H	8.212076	-2.228917	-0.391829
H	8.328233	-2.451863	1.372702
C	6.515533	1.748673	0.000955
C	8.011055	1.811939	0.376006
H	8.673073	1.466945	-0.441886
H	8.246914	1.223186	1.284074
H	8.283050	2.867535	0.597212
C	6.244623	2.718771	-1.172325
H	6.460876	3.752260	-0.824827
H	5.183179	2.697354	-1.494014
H	6.889223	2.531469	-2.052476
C	5.654345	2.254682	1.186279
H	5.924717	3.313244	1.392750
H	5.811315	1.687509	2.120443
H	4.570273	2.226863	0.947416
C	1.372876	-0.028199	-0.163906
O	0.790854	-0.021693	1.001780
O	0.527432	-0.023085	-1.163003
Ag	3.452355	-0.034250	-0.363257

TS3-Ag

E= -3407.6568025

P	4.810095	-0.019629	-0.117282
Al	-0.500035	-0.004722	-0.374086
O	-2.410945	0.015770	-0.943887
N	-0.976414	-1.801080	0.157520
N	-0.938292	1.801807	0.156572
C	-3.123178	-1.158951	-0.651154
C	-2.313769	-2.150892	-0.068550
C	-2.975500	-3.370387	0.233179
H	-2.383460	-4.190469	0.659047
C	-4.366640	-3.517049	0.022454
C	-5.119270	-2.429120	-0.490673
H	-6.205354	-2.535313	-0.621994
C	-4.501345	-1.209502	-0.840457
C	-5.193808	0.045435	-1.423502
C	-4.474581	1.285820	-0.841726
C	-5.065989	2.519078	-0.493850
H	-6.149463	2.648662	-0.625716
C	-4.290077	3.591195	0.017896
C	-2.902548	3.414598	0.229684
H	-2.292995	4.222324	0.654537
C	-2.267507	2.180478	-0.069864
C	-3.097929	1.205635	-0.651806
C	-4.994826	4.930862	0.349500
C	-4.016522	5.989483	0.901921
H	-3.537132	5.659909	1.846737
H	-4.564196	6.930549	1.118190
H	-3.213221	6.232056	0.175976
C	-5.644838	5.500878	-0.938303
H	-4.879642	5.688981	-1.720084
H	-6.159243	6.462189	-0.723021
H	-6.396454	4.805916	-1.365306
C	-6.093188	4.686384	1.416898
H	-6.860977	3.966948	1.066088
H	-6.612923	5.636656	1.665585
H	-5.654958	4.280178	2.352341
C	-6.698643	0.061745	-1.101577
H	-6.885708	0.064304	-0.008696
H	-7.203425	-0.822367	-1.540364
H	-7.184387	0.955986	-1.541319
C	-4.999175	0.042575	-2.970002
H	-5.457031	0.949051	-3.418037
H	-5.476626	-0.854149	-3.417208
H	-3.924824	0.030737	-3.242620
C	-5.100256	-4.840369	0.357145
C	-4.144463	-5.919879	0.908631
H	-3.656148	-5.600264	1.852289
H	-3.348042	-6.180690	0.181426
H	-4.712338	-6.848478	1.126686
C	-6.190395	-4.570273	1.426779
H	-6.942809	-3.834363	1.076849
H	-5.741020	-4.172810	2.360690
H	-6.730497	-5.508473	1.677886
C	-5.765719	-5.397345	-0.928472
H	-6.300547	-6.346920	-0.710900
H	-5.006777	-5.603001	-1.711923
H	-6.503016	-4.686594	-1.354441
C	-0.097837	-2.863803	0.565529
C	0.417961	-3.769400	-0.414690
C	1.216061	-4.851509	0.013587
H	1.608119	-5.558502	-0.735708
C	1.515609	-5.047340	1.367277
H	2.132941	-5.904291	1.682048
C	1.026345	-4.143145	2.319987
H	1.268504	-4.300036	3.382432
C	0.218354	-3.047645	1.948484
C	-0.339724	-2.118079	3.028651
H	-0.529049	-1.136970	2.540542
C	0.640198	-1.884469	4.192865
H	0.233099	-1.125283	4.891261
H	1.628365	-1.526532	3.837402
H	0.807379	-2.806862	4.788654
C	-1.696505	-2.625636	3.568157
H	-2.446556	-2.731715	2.760088
H	-2.106256	-1.922473	4.324355
H	-1.577349	-3.617310	4.056081
C	0.126440	-3.617655	-1.909150
H	-0.429940	-2.668829	-2.044031
C	1.424602	-3.507820	-2.735206
H	2.064835	-2.680194	-2.366082
H	1.193753	-3.295660	-3.799503
H	2.017794	-4.446908	-2.699512
C	-0.765931	-4.760736	-2.440497
H	-0.260263	-5.746536	-2.350184
H	-1.000934	-4.602565	-3.514333
H	-1.725339	-4.816411	-1.887622
C	-0.035511	2.845166	0.561543
C	0.278906	3.031140	1.944464
C	1.111410	4.109450	2.312207
H	1.351093	4.268616	3.374898
C	1.631899	4.991169	1.355043
H	2.269868	5.834043	1.666719
C	1.336251	4.791328	0.001006
H	1.751418	5.481325	-0.751719
C	0.509370	3.729050	-0.422937
C	0.216672	3.576106	-1.916894
H	-0.361090	2.639603	-2.047121
C	-0.649068	4.736510	-2.454776
H	-1.607646	4.816367	-1.903400
H	-0.886387	4.578570	-3.528148
H	-0.121521	5.711148	-2.368310
C	1.512570	3.431483	-2.740848
H	2.124370	4.358979	-2.715546
H	1.278461	3.212414	-3.803116
H	2.136392	2.595439	-2.362606
C	-0.301073	2.119463	3.028209
H	-0.520762	1.144239	2.541100
C	-1.639526	2.666419	3.574557
H	-1.489454	3.654670	4.060932
H	-2.065458	1.976017	4.333566
H	-2.390426	2.793256	2.770210
C	0.679359	1.857876	4.186344
H	1.653885	1.470382	3.824259
H	0.254180	1.112013	4.888432
H	0.877716	2.775499	4.779948
C	5.283431	-1.476481	1.085483
C	6.779302	-1.858904	1.080938
H	7.439202	-1.016269	1.364925
H	7.115670	-2.246480	0.099650
H	6.944639	-2.672103	1.821979

C	4.863063	-1.106835	2.526152
H	3.797425	-0.803484	2.581515
H	5.485596	-0.308137	2.973221
H	4.980546	-2.009079	3.164809
C	4.427553	-2.704062	0.687544
H	3.341491	-2.473788	0.705106
H	4.603659	-3.515274	1.426781
H	4.673015	-3.108895	-0.309663
C	5.364592	1.678323	0.660045
C	4.318710	2.062567	1.735516
H	4.560263	3.077480	2.119227
H	4.304388	1.377660	2.601601
H	3.292711	2.108097	1.313444
C	5.294889	2.782443	-0.419700
H	5.455783	3.763300	0.077986
H	4.299996	2.825859	-0.907621
H	6.073012	2.681400	-1.200325
C	6.777168	1.671258	1.282871
H	7.566565	1.409122	0.551979
H	6.859385	0.979956	2.144274
H	7.006481	2.690549	1.665223
C	5.694172	-0.275575	-1.836984
C	7.194728	0.086608	-1.847683
H	7.783595	-0.497244	-1.113659
H	7.375154	1.163125	-1.659456
H	7.609442	-0.136483	-2.855694
C	5.528291	-1.745936	-2.284210
H	5.880714	-1.828342	-3.335246
H	4.467483	-2.068451	-2.272288
H	6.126708	-2.457664	-1.683423
C	4.934756	0.580134	-2.881759
H	5.397428	0.406144	-3.878214
H	4.986519	1.665301	-2.684430
H	3.865401	0.291344	-2.967110
C	0.548359	-0.036434	-3.270105
O	1.588176	-0.066570	-3.873776
O	0.449009	-0.011534	-1.907442
Ag	2.346068	-0.010784	-0.468718

TS3′-Ag

E= -3407.6766959

P	-5.256509	-0.102044	-0.251017
Al	0.633392	-0.029356	-0.165000
O	2.428541	0.094859	-1.047844
N	1.323669	-1.760448	0.355245
N	1.090562	1.762229	0.397725
C	3.229426	-1.054878	-0.883924
C	2.591121	-2.069044	-0.139185
C	3.349256	-3.256327	0.023245
H	2.889596	-4.099569	0.559681
C	4.683790	-3.349222	-0.447470
C	5.284617	-2.233603	-1.077640
H	6.332648	-2.281608	-1.396487
C	4.555054	-1.041762	-1.300005
C	5.091336	0.264752	-1.931727
C	4.446069	1.436118	-1.152065
C	5.061406	2.652891	-0.789618
H	6.110091	2.826390	-1.069396
C	4.354628	3.650505	-0.068210
C	3.018626	3.413808	0.329125
H	2.464488	4.162193	0.910469
C	2.364961	2.191441	0.017772
C	3.116889	1.292896	-0.761844
C	5.081939	4.972865	0.283394
C	6.332217	4.660536	1.146434
H	6.045880	4.154756	2.092093
H	6.869697	5.597736	1.406942
H	7.048488	4.000080	0.616408
C	5.522514	5.680363	-1.024705
H	6.209847	5.049863	-1.624971
H	6.051041	6.630794	-0.795482
H	4.645638	5.920804	-1.661444
C	4.183225	5.946002	1.076063
H	3.275797	6.232439	0.505374
H	4.743630	6.877973	1.299241
H	3.857037	5.514600	2.044861
C	4.625772	0.335653	-3.416926
H	3.521583	0.288472	-3.498636
H	5.048482	-0.513473	-3.993495
H	4.965104	1.284140	-3.883238
C	6.627792	0.329435	-1.881797
H	6.995644	1.266395	-2.346682
H	7.074280	-0.508195	-2.454605
H	7.008607	0.282728	-0.841581
C	5.459481	-4.672955	-0.228132
C	5.559605	-4.972663	1.290052
H	4.559946	-5.076408	1.758494
H	6.097336	-4.159630	1.821128
H	6.112220	-5.920966	1.464539
C	4.708207	-5.832402	-0.932092
H	4.622637	-5.646911	-2.023194
H	3.682462	-5.963486	-0.531590
H	5.249747	-6.792176	-0.788352
C	6.892645	-4.614603	-0.799588
H	7.404838	-5.584634	-0.629259
H	7.503595	-3.827785	-0.310356
H	6.895698	-4.424227	-1.892960
C	0.589046	-2.830124	0.972314
C	0.614473	-2.988940	2.393490
C	-0.076622	-4.076320	2.968982
H	-0.058589	-4.210943	4.061712
C	-0.778201	-4.996429	2.178330
H	-1.301957	-5.845036	2.647695
C	-0.809677	-4.827398	0.788344
H	-1.366222	-5.549443	0.168497
C	-0.141789	-3.754017	0.161675
C	-0.209578	-3.628071	-1.361200
H	0.232265	-2.647072	-1.626537
C	-1.662970	-3.623632	-1.874025
H	-2.176297	-4.591501	-1.687685
H	-1.688770	-3.442422	-2.969207
H	-2.248819	-2.818011	-1.383326
C	0.614811	-4.728400	-2.064956
H	1.676779	-4.704463	-1.748513
H	0.586506	-4.593383	-3.167261
H	0.213611	-5.740596	-1.840144
C	1.399110	-2.029354	3.291545
H	1.468612	-1.065584	2.741691
C	0.705046	-1.760151	4.639771
H	-0.350928	-1.447335	4.508277
H	1.237554	-0.957652	5.191564
H	0.713163	-2.655833	5.297030
C	2.845500	-2.518552	3.529935
H	2.845990	-3.496050	4.059378
H	3.406464	-1.792942	4.156903
H	3.399112	-2.645207	2.579336
C	0.156479	2.775972	0.799544
C	-0.376748	3.678975	-0.175128
C	-1.249545	4.702387	0.249901
H	-1.652192	5.408114	-0.495331
C	-1.621493	4.835368	1.593275
H	-2.300665	5.644938	1.906269
C	-1.125577	3.925589	2.537315
H	-1.426623	4.029837	3.590635
C	-0.236078	2.893963	2.168914
C	0.329308	1.955472	3.237195
H	0.475725	0.964734	2.752361
C	1.720560	2.427075	3.717132
H	2.437779	2.505495	2.876533
H	2.143089	1.718745	4.461550
H	1.648958	3.425375	4.200394
C	-0.613160	1.754202	4.437360
H	-0.721931	2.680778	5.040454
H	-0.210043	0.976808	5.116435
H	-1.627742	1.436668	4.118486
C	-0.052894	3.570005	-1.667406
H	0.557691	2.655988	-1.809241
C	0.773669	4.771688	-2.174621
H	0.207391	5.722920	-2.074281
H	1.027028	4.645922	-3.248784
H	1.722819	4.876443	-1.611873
C	-1.332297	3.395821	-2.511494
H	-1.925955	2.530868	-2.150586
H	-1.076972	3.213364	-3.576636
H	-1.978600	4.298861	-2.474547

C	-6.608411	-0.851595	-1.443489
C	-7.919820	-1.273659	-0.747542
H	-8.416393	-0.434066	-0.223698
H	-7.769611	-2.094820	-0.019744
H	-8.631743	-1.649858	-1.515082
C	-6.931632	0.175992	-2.552251
H	-6.015163	0.531362	-3.066981
H	-7.497187	1.053401	-2.183845
H	-7.566545	-0.322895	-3.316493
C	-5.979935	-2.072749	-2.160044
H	-6.698261	-2.445540	-2.922747
H	-5.752198	-2.913987	-1.481911
H	-5.042732	-1.792948	-2.685071
C	-5.737662	1.728237	0.216710
C	-5.360012	2.639149	-0.977856
H	-5.951271	2.442842	-1.889625
H	-4.284948	2.544636	-1.236287
H	-5.540048	3.696743	-0.685568
C	-4.861960	2.190785	1.403768
H	-5.020056	3.281210	1.548445
H	-3.779562	2.045057	1.210381
H	-5.126100	1.695293	2.357545
C	-7.225506	1.929946	0.574334
H	-7.550367	1.301265	1.425745
H	-7.900994	1.730881	-0.280645
H	-7.383585	2.990705	0.869317
C	-5.145093	-1.183824	1.369963
C	-6.295024	-0.961334	2.375063
H	-7.292077	-1.174522	1.943023
H	-6.307830	0.067252	2.785546
H	-6.155995	-1.649678	3.237663
C	-5.097407	-2.678927	0.978769
H	-4.865824	-3.269053	1.891596
H	-4.293221	-2.892235	0.245352
H	-6.058154	-3.058434	0.580633
C	-3.786959	-0.872813	2.047950
H	-3.652538	-1.570230	2.903081
H	-3.719001	0.155441	2.444770
H	-2.935218	-1.026885	1.352918
C	-1.575464	-0.181824	-2.843642
O	-0.918367	-0.122203	-0.866284
O	-0.699759	-0.231174	-3.602518
Ag	-3.131903	-0.096799	-1.304658

IM2-Ag

E= -3407.6867179

P	5.039239	-0.030131	-0.080820
Al	-0.656931	-0.004128	-0.294270
O	-2.550036	0.015408	-0.971996
N	-1.151379	1.773722	0.276218
N	-1.194461	-1.764784	0.293726
C	-3.255935	1.197587	-0.669849
C	-2.461608	2.158758	-0.009590
C	-3.117889	3.380125	0.282041
H	-2.537516	4.183460	0.759710
C	-4.498017	3.557604	0.007991
C	-5.240523	2.499739	-0.568584
H	-6.316055	2.618427	-0.746446
C	-4.620403	1.276951	-0.920125
C	-5.308413	0.042223	-1.550675
C	-4.644117	-1.211600	-0.933169
C	-5.281733	-2.422913	-0.589422
H	-6.360657	-2.529737	-0.769883
C	-4.557144	-3.500251	-0.015787
C	-3.181178	-3.346076	0.270844
H	-2.612277	-4.154429	0.748290
C	-2.505311	-2.128916	-0.012956
C	-3.276379	-1.157266	-0.678996
C	-5.308030	-4.817492	0.304164
C	-6.458157	-4.527844	1.303593
H	-6.063578	-4.109303	2.252909
H	-7.010353	-5.462072	1.543174
H	-7.190355	-3.801632	0.895449
C	-5.898379	-5.401742	-1.005807
H	-6.609231	-4.700056	-1.488082
H	-6.445281	-6.347184	-0.800163
H	-5.095248	-5.622624	-1.739518
C	-4.385143	-5.883868	0.931789
H	-3.547854	-6.157605	0.257089
H	-4.963835	-6.809081	1.135613
H	-3.952044	-5.543974	1.895069
C	-5.038733	0.048001	-3.085165
H	-3.952481	0.037950	-3.304938
H	-5.474682	0.956765	-3.550248
H	-5.493290	-0.846290	-3.560391
C	-6.827881	0.054789	-1.306401
H	-7.307809	-0.827772	-1.775389
H	-7.291721	0.951427	-1.764672
H	-7.070675	0.050438	-0.224624
C	-5.163256	4.905857	0.384070
C	-5.055317	5.123952	1.915924
H	-4.000120	5.148312	2.256074
H	-5.568327	4.310267	2.469967
H	-5.525605	6.087882	2.207287
C	-4.442009	6.064361	-0.351681
H	-4.502764	5.936010	-1.452590
H	-3.369017	6.124390	-0.077929
H	-4.907855	7.039728	-0.093625
C	-6.657470	4.951129	-0.002175
H	-7.087695	5.936138	0.275257
H	-7.247243	4.172924	0.525225
H	-6.807651	4.817643	-1.093709
C	-0.213010	2.810150	0.602027
C	0.237143	2.974449	1.948738
C	1.164373	3.998843	2.235567
H	1.515005	4.138300	3.269458
C	1.638500	4.856276	1.232401
H	2.354679	5.656558	1.480169
C	1.192064	4.689432	-0.084628
H	1.569628	5.360843	-0.873398
C	0.272184	3.676435	-0.427288
C	-0.151264	3.532933	-1.891133
H	-0.906103	2.723665	-1.942971
C	1.035985	3.094225	-2.773427
H	1.848824	3.852395	-2.771427
H	0.708702	2.957520	-3.826314
H	1.439224	2.127269	-2.407781
C	-0.814270	4.815874	-2.434486
H	-1.691196	5.110236	-1.822930
H	-1.164873	4.659420	-3.476765
H	-0.105903	5.672149	-2.449892
C	-0.306097	2.092313	3.075551
H	-0.495828	1.088747	2.634257
C	0.675633	1.912682	4.246824
H	1.669064	1.554598	3.906183
H	0.277168	1.173858	4.971497
H	0.829493	2.858758	4.808558
C	-1.665134	2.614219	3.594398
H	-1.551877	3.629066	4.033384
H	-2.068333	1.945132	4.384385
H	-2.417781	2.674705	2.784236
C	-0.284003	-2.808873	0.669908
C	0.223791	-3.701156	-0.325195
C	1.123346	-4.714617	0.066962
H	1.519066	-5.405735	-0.695539
C	1.525775	-4.858354	1.400826
H	2.227113	-5.658813	1.687406
C	1.024044	-3.979848	2.371813
H	1.337925	-4.103300	3.419474
C	0.115651	-2.954088	2.035021
C	-0.463954	-2.051448	3.127177
H	-0.629946	-1.053804	2.663467
C	-1.844748	-2.558339	3.602329
H	-2.566530	-2.630325	2.765541
H	-2.273943	-1.873558	4.364690
H	-1.752940	-3.565832	4.062822
C	0.472099	-1.859844	4.333287
H	0.601581	-2.799421	4.911823
H	0.045499	-1.112896	5.033721
H	1.478908	-1.507000	4.028672
C	-0.161192	-3.591029	-1.802321
H	-0.894027	-2.765427	-1.895758
C	-0.844094	-4.874772	-2.319674
H	-0.156360	-5.747434	-2.292917
H	-1.170732	-4.743476	-3.373132

H	-1.739563	-5.128332	-1.716203
C	1.055196	-3.210040	-2.671000
H	1.473944	-2.241802	-2.327100
H	0.754851	-3.098244	-3.734655
H	1.849044	-3.986887	-2.627287
C	5.960124	1.316022	-1.149175
C	7.355204	1.712980	-0.621396
H	8.049852	0.853244	-0.554517
H	7.312855	2.202541	0.371249
H	7.808406	2.447724	-1.322898
C	6.094405	0.803936	-2.601902
H	5.119896	0.481710	-3.022337
H	6.823138	-0.022819	-2.706653
H	6.463349	1.642942	-3.231337
C	5.049743	2.567818	-1.216112
H	5.520866	3.309208	-1.898277
H	4.902046	3.063426	-0.240620
H	4.048207	2.321832	-1.625544
C	5.799955	-1.788470	-0.442717
C	5.220925	-2.285538	-1.790906
H	5.543817	-1.685193	-2.659394
H	4.111443	-2.297406	-1.777055
H	5.567513	-3.328733	-1.959442
C	5.304058	-2.784415	0.630976
H	5.603360	-3.808354	0.318178
H	4.198588	-2.782688	0.725957
H	5.750614	-2.608053	1.628468
C	7.342212	-1.823047	-0.492861
H	7.809970	-1.480145	0.450319
H	7.754965	-1.218402	-1.323959
H	7.673216	-2.871437	-0.662112
C	5.236826	0.389978	1.812409
C	6.621940	0.056688	2.406105
H	7.447839	0.586334	1.893168
H	6.842445	-1.028591	2.388542
H	6.641215	0.371858	3.472685
C	4.937753	1.891253	2.029585
H	4.892659	2.081038	3.124069
H	3.956417	2.189039	1.605710
H	5.721423	2.556176	1.618616
C	4.139034	-0.383812	2.584177
H	4.163197	-0.063835	3.648895
H	4.269732	-1.479928	2.564659
H	3.129432	-0.155710	2.182785
C	2.534087	-0.026012	-4.310123
O	0.807875	-0.026334	-1.158530
O	1.571195	-0.118566	-4.917707
Ag	2.754969	-0.032196	-0.626390

8-Ag

E= -3294.3225933

P	5.118087	-0.050338	-0.281157
Al	-0.647881	-0.013071	-0.353939
O	-2.570253	0.029938	-0.985876
N	-1.112160	1.776995	0.189648
N	-1.194421	-1.777525	0.198481
C	-3.254210	1.220072	-0.671211
C	-2.425315	2.179001	-0.051504
C	-3.051975	3.413950	0.245015
H	-2.442468	4.215374	0.688460
C	-4.437978	3.607963	0.015185
C	-5.214518	2.553332	-0.520976
H	-6.293525	2.686127	-0.664863
C	-4.624831	1.316699	-0.877824
C	-5.351708	0.089800	-1.478986
C	-4.677767	-1.174070	-0.893518
C	-5.313626	-2.386022	-0.547483
H	-6.398912	-2.481016	-0.693383
C	-4.579941	-3.479870	-0.018413
C	-3.193336	-3.343148	0.222663
H	-2.613892	-4.165625	0.661917
C	-2.518381	-2.126437	-0.062316
C	-3.302136	-1.135266	-0.683273
C	-5.331656	-4.795970	0.304112
C	-6.436887	-4.515538	1.355446
H	-5.999217	-4.121738	2.296419
H	-6.989944	-5.448559	1.597802
H	-7.176206	-3.772574	0.992770
C	-5.980877	-5.347205	-0.992199
H	-6.703923	-4.629376	-1.430568
H	-6.528413	-6.291619	-0.783590
H	-5.210658	-5.560135	-1.762615
C	-4.395571	-5.884448	0.871400
H	-3.588506	-6.150832	0.157997
H	-4.975126	-6.808488	1.078132
H	-3.921208	-5.569970	1.823810
C	-5.145577	0.095492	-3.023416
H	-4.069278	0.073431	-3.287195
H	-5.589354	1.010232	-3.469016
H	-5.629210	-0.792651	-3.481188
C	-6.859692	0.119292	-1.171866
H	-7.368975	-0.756749	-1.621679
H	-7.332048	1.021895	-1.609204
H	-7.057081	0.115674	-0.080882
C	-5.069303	4.972307	0.391153
C	-4.900974	5.216342	1.913628
H	-3.833592	5.230608	2.214000
H	-5.404848	4.420775	2.501361
H	-5.345793	6.192502	2.204274
C	-4.358684	6.105826	-0.392537
H	-4.463191	5.958770	-1.487814
H	-3.275161	6.152602	-0.160829
H	-4.798968	7.093154	-0.135015
C	-6.576034	5.033482	0.059793
H	-6.981563	6.029248	0.336036
H	-7.156702	4.272831	0.621808
H	-6.768603	4.884232	-1.023000
C	-0.137680	2.795144	0.458392
C	0.386323	2.953475	1.778221
C	1.359054	3.949619	2.008039
H	1.767265	4.085535	3.021186
C	1.807067	4.783209	0.973041
H	2.560628	5.561651	1.175602
C	1.286534	4.622340	-0.317781
H	1.644236	5.275381	-1.130648
C	0.317157	3.638368	-0.603231
C	-0.185828	3.489563	-2.042000
H	-1.032651	2.775200	-2.027054
C	0.901306	2.875393	-2.950948
H	1.803818	3.523395	-2.994508
H	0.521440	2.762003	-3.989363
H	1.188937	1.871723	-2.572551
C	-0.723634	4.816445	-2.615917
H	-1.521262	5.244266	-1.974542
H	-1.152456	4.653690	-3.627403
H	0.076823	5.580417	-2.719307
C	-0.126023	2.092626	2.935635
H	-0.364677	1.092465	2.510014
C	0.904692	1.888975	4.059756
H	1.870599	1.502799	3.673603
H	0.521004	1.162724	4.805127
H	1.111168	2.831859	4.609961
C	-1.443962	2.655432	3.514274
H	-1.281448	3.668338	3.942081
H	-1.829603	2.001432	4.325406
H	-2.230972	2.734592	2.738991
C	-0.266731	-2.828285	0.505001
C	0.188417	-3.698174	-0.534213
C	1.121484	-4.705869	-0.212621
H	1.480839	-5.378619	-1.008591
C	1.602805	-4.866629	1.093350
H	2.328916	-5.663084	1.324139
C	1.148261	-4.012070	2.108165
H	1.523628	-4.149876	3.133676
C	0.211629	-2.991108	1.841819
C	-0.313228	-2.108682	2.977406
H	-0.511511	-1.105542	2.538235
C	-1.663176	-2.633080	3.517627
H	-2.427193	-2.698577	2.718490
H	-2.056909	-1.962948	4.311543
H	-1.540018	-3.646473	3.957323
C	0.685379	-1.925094	4.133453
H	0.851411	-2.870448	4.693026
H	0.293623	-1.188157	4.864391
H	1.672178	-1.562920	3.778389

C	-0.281661	-3.559186	-1.984838
H	-1.101010	-2.813442	-1.999675
C	-0.860211	-4.877752	-2.538887
H	-0.087213	-5.672706	-2.613562
H	-1.269180	-4.721827	-3.559627
H	-1.681445	-5.261929	-1.899159
C	0.842636	-3.006115	-2.887415
H	1.157681	-2.004000	-2.527155
H	0.485802	-2.904879	-3.935213
H	1.722740	-3.685574	-2.898793
C	5.946640	1.310962	-1.406373
C	7.387478	1.690627	-1.003662
H	8.079350	0.826194	-1.016208
H	7.439155	2.161088	-0.002298
H	7.779338	2.436566	-1.729811
C	5.942398	0.822696	-2.873419
H	4.929954	0.510871	-3.203140
H	6.650808	-0.007040	-3.060600
H	6.253590	1.669585	-3.522871
C	5.043288	2.568992	-1.369418
H	5.449912	3.315536	-2.086525
H	4.996368	3.054293	-0.379003
H	4.003719	2.334290	-1.679154
C	5.840447	-1.804789	-0.735464
C	5.143039	-2.282914	-2.033840
H	5.393473	-1.674352	-2.920638
H	4.038578	-2.291702	-1.923476
H	5.468668	-3.325121	-2.244747
C	5.440146	-2.816465	0.363278
H	5.712657	-3.835247	0.011627
H	4.346846	-2.818821	0.551952
H	5.970344	-2.652983	1.321225
C	7.371995	-1.839865	-0.923682
H	7.923385	-1.511288	-0.021456
H	7.710188	-1.223415	-1.779496
H	7.684360	-2.885823	-1.137305
C	5.480613	0.342814	1.592639
C	6.910871	-0.004006	2.057594
H	7.689987	0.533317	1.482963
H	7.125182	-1.089143	2.001308
H	7.024790	0.292268	3.123632
C	5.210418	1.842070	1.856346
H	5.255930	2.014605	2.953675
H	4.200730	2.152799	1.516890
H	5.963397	2.508261	1.393294
C	4.449968	-0.438371	2.445105
H	4.571908	-0.139755	3.509296
H	4.569899	-1.534673	2.392917
H	3.410389	-0.195633	2.141162
O	0.847562	-0.051329	-1.161173
Ag	2.804157	-0.047334	-0.672617

TS4-Ag

E= -3483.0123313

P	5.162488	-0.028033	-0.066448
Al	-0.673325	-0.006044	-0.354450
O	-2.598130	0.013998	-0.911306
N	-1.136212	1.786039	0.194700
N	-1.175416	-1.784076	0.206010
C	-3.288166	1.201395	-0.622812
C	-2.458651	2.176744	-0.032470
C	-3.094568	3.408035	0.258927
H	-2.490764	4.220327	0.689795
C	-4.485091	3.587018	0.039065
C	-5.257367	2.519627	-0.476825
H	-6.338158	2.642561	-0.615905
C	-4.660580	1.282661	-0.820373
C	-5.378991	0.042257	-1.403541
C	-4.688854	-1.211356	-0.814792
C	-5.307298	-2.430468	-0.462375
H	-6.392745	-2.538215	-0.598075
C	-4.555951	-3.515244	0.059693
C	-3.166293	-3.365753	0.279299
H	-2.574840	-4.182748	0.712267
C	-2.505900	-2.145400	-0.021720
C	-3.312238	-1.158478	-0.617552
C	-5.290124	-4.838167	0.395210
C	-6.385363	-4.565976	1.459065
H	-5.940391	-4.165149	2.393654
H	-6.926042	-5.503789	1.710732
H	-7.136455	-3.831475	1.103402
C	-5.949433	-5.400133	-0.891394
H	-6.684996	-4.691118	-1.323198
H	-6.484725	-6.349400	-0.673219
H	-5.186642	-5.607798	-1.670600
C	-4.336045	-5.915314	0.954287
H	-3.535691	-6.176878	0.231676
H	-4.903971	-6.844024	1.172095
H	-3.852596	-5.592545	1.899365
C	-5.179961	0.036450	-2.949358
H	-4.104799	0.022920	-3.218052
H	-5.635808	0.942765	-3.400052
H	-5.657625	-0.860368	-3.396453
C	-6.884923	0.059507	-1.086234
H	-7.388475	-0.825386	-1.525036
H	-7.369221	0.953367	-1.528422
H	-7.075263	0.063655	0.006097
C	-5.123793	4.952910	0.397458
C	-4.930233	5.234603	1.910215
H	-3.857969	5.267753	2.190926
H	-5.415030	4.448453	2.526010
H	-5.380140	6.212803	2.185684
C	-4.440260	6.075324	-0.425497
H	-4.564496	5.902156	-1.514896
H	-3.352977	6.138116	-0.216572
H	-4.885863	7.063904	-0.182135
C	-6.637081	4.990882	0.093600
H	-7.048825	5.987050	0.358990
H	-7.198106	4.234100	0.680278
H	-6.848381	4.819648	-0.982390
C	-0.214402	2.819840	0.578180
C	0.124508	3.007763	1.954615
C	0.988002	4.068049	2.304215
H	1.247167	4.227454	3.362249
C	1.513363	4.932915	1.334581
H	2.174428	5.763294	1.631601
C	1.193027	4.730939	-0.013636
H	1.614128	5.405563	-0.777064
C	0.339630	3.682927	-0.418487
C	0.037625	3.515029	-1.908311
H	-0.542916	2.578049	-2.022156
C	1.329858	3.361366	-2.736903
H	1.945060	4.286749	-2.716841
H	1.089803	3.151493	-3.800525
H	1.949035	2.523658	-2.356932
C	-0.830229	4.669959	-2.454007
H	-1.786465	4.756378	-1.900156
H	-1.072269	4.503110	-3.525203
H	-0.301245	5.644825	-2.378233
C	-0.457802	2.113449	3.051951
H	-0.684947	1.133040	2.578529
C	0.522553	1.863324	4.212697
H	1.498108	1.473448	3.855721
H	0.097527	1.124637	4.922322
H	0.719431	2.787331	4.796942
C	-1.793106	2.671781	3.593873
H	-1.638801	3.666605	4.065265
H	-2.219107	1.993824	4.364065
H	-2.546036	2.788812	2.790160
C	-0.275816	-2.834859	0.596066
C	0.254211	-3.720738	-0.393584
C	1.091177	-4.779098	0.018133
H	1.495090	-5.470082	-0.739893
C	1.417836	-4.970443	1.366409
H	2.066830	-5.808426	1.668538
C	0.912821	-4.086312	2.329382
H	1.174487	-4.239174	3.387821
C	0.065615	-3.015439	1.972831
C	-0.501879	-2.104533	3.064080
H	-0.721079	-1.126046	2.583086
C	-1.840948	-2.645266	3.615223
H	-2.596720	-2.762624	2.814037
H	-2.258605	-1.956223	4.380150
H	-1.694421	-3.637254	4.095048
C	0.485274	-1.854359	4.218889

H	0.676613	-2.775476	4.809482
H	0.070299	-1.106097	4.924408
H	1.462827	-1.475929	3.854788
C	-0.058220	-3.567970	-1.882988
H	-0.624277	-2.623186	-2.005255
C	-0.950285	-4.716363	-2.403556
H	-0.435892	-5.698338	-2.319969
H	-1.201993	-4.560287	-3.474145
H	-1.901381	-4.779200	-1.837347
C	1.228365	-3.447767	-2.725883
H	1.863332	-2.613465	-2.365329
H	0.981009	-3.252324	-3.790589
H	1.830009	-4.381694	-2.695485
C	6.091631	-0.269660	-1.764276
C	7.591409	0.093294	-1.730176
H	8.159948	-0.494772	-0.983783
H	7.764721	1.168715	-1.529278
H	8.035664	-0.122572	-2.726751
C	5.934570	-1.738448	-2.220741
H	4.871874	-2.056052	-2.236430
H	6.513445	-2.452955	-1.604526
H	6.316826	-1.820760	-3.261279
C	5.367230	0.591000	-2.830239
H	5.836058	0.390953	-3.818639
H	5.442122	1.677075	-2.645875
H	4.291891	0.328537	-2.912407
C	5.607856	-1.489318	1.144084
C	4.777083	-2.726713	0.721960
H	5.057037	-3.130339	-0.266681
H	3.689155	-2.507772	0.706242
H	4.942891	-3.533925	1.468165
C	5.140166	-1.124414	2.571590
H	5.247249	-2.025829	3.213063
H	4.070302	-0.831920	2.593994
H	5.741235	-0.320280	3.037750
C	7.107189	-1.854717	1.180735
H	7.750059	-1.005173	1.482618
H	7.474356	-2.238885	0.209054
H	7.261383	-2.665669	1.926160
C	5.676608	1.673268	0.732907
C	7.072723	1.674756	1.391168
H	7.882358	1.414715	0.681937
H	7.136080	0.986476	2.256605
H	7.287441	2.695897	1.776147
C	5.624195	2.776376	-0.349000
H	5.754809	3.759793	0.152343
H	4.643936	2.802325	-0.867522
H	6.428358	2.686925	-1.104370
C	4.600777	2.049920	1.782006
H	4.834097	3.062159	2.178000
H	4.562636	1.360254	2.643480
H	3.587517	2.097235	1.331385
O	0.901836	-0.026504	-1.047071
C	0.559834	-0.038135	-3.192867
O	-0.633784	-0.024087	-3.161033
O	1.661708	-0.055092	-3.626202
Ag	2.856832	-0.027525	-0.504824

IM3-Ag

E= -3483.0532222

P	-5.428668	-0.027558	0.132480
Al	0.947927	-0.006313	-0.671141
O	2.944990	0.030349	-0.897930
N	1.357723	-1.799979	-0.105565
N	1.290592	1.800192	-0.105179
C	3.604382	-1.150405	-0.526624
C	2.705818	-2.163980	-0.129534
C	3.308653	-3.396481	0.221149
H	2.657120	-4.235862	0.506181
C	4.719996	-3.543279	0.248134
C	5.545724	-2.439845	-0.069990
H	6.636436	-2.537964	-0.013375
C	4.991202	-1.198032	-0.464883
C	5.780515	0.077520	-0.845628
C	4.949976	1.300718	-0.387338
C	5.455963	2.533182	0.081324
H	6.543919	2.667756	0.161989
C	4.591343	3.598293	0.443536
C	3.189664	3.418502	0.374834
H	2.503628	4.221360	0.674642
C	2.631586	2.187115	-0.056022
C	3.563199	1.214194	-0.466238
C	5.213573	4.934842	0.921219
C	6.075069	4.681274	2.185681
H	5.457748	4.268518	3.010750
H	6.534377	5.628840	2.541206
H	6.897229	3.962859	1.990551
C	6.108856	5.516261	-0.204155
H	6.932480	4.825152	-0.476565
H	6.567490	6.475949	0.118237
H	5.516438	5.710594	-1.122512
C	4.143041	5.989668	1.273759
H	3.501155	6.237047	0.403188
H	4.634755	6.929198	1.602392
H	3.484180	5.653337	2.100775
C	5.921185	0.129445	-2.396623
H	4.929313	0.128128	-2.891334
H	6.488114	-0.751983	-2.762673
H	6.457964	1.050561	-2.706040
C	7.183421	0.081142	-0.210743
H	7.745487	0.990216	-0.505218
H	7.776681	-0.786892	-0.562133
H	7.133195	0.044717	0.896275
C	5.317866	-4.914681	0.652994
C	4.865731	-5.274132	2.092263
H	3.761905	-5.340525	2.174926
H	5.212462	-4.510855	2.819903
H	5.285101	-6.256593	2.398978
C	4.819379	-6.005900	-0.329248
H	5.129386	-5.776414	-1.370091
H	3.714231	-6.097066	-0.319953
H	5.239672	-6.997862	-0.056333
C	6.861516	-4.913686	0.623294
H	7.243505	-5.915620	0.910649
H	7.290650	-4.179170	1.336041
H	7.256476	-4.684622	-0.388246
C	0.398207	-2.837471	0.152876
C	-0.109584	-3.029334	1.476418
C	-1.040911	-4.064870	1.702056
H	-1.433394	-4.227849	2.717926
C	-1.468825	-4.901971	0.661358
H	-2.188961	-5.711957	0.862173
C	-0.971207	-4.703951	-0.632638
H	-1.310059	-5.361283	-1.450048
C	-0.040994	-3.680924	-0.915400
C	0.478350	-3.528829	-2.347896
H	1.079709	-2.598741	-2.383739
C	-0.661820	-3.371707	-3.375859
H	-1.290012	-4.286654	-3.439523
H	-0.240741	-3.191238	-4.387155
H	-1.322952	-2.514940	-3.136875
C	1.404154	-4.702155	-2.738763
H	2.264473	-4.791737	-2.045715
H	1.808242	-4.554020	-3.762784
H	0.856137	-5.669485	-2.731707
C	0.364472	-2.163136	2.647039
H	0.671386	-1.185148	2.216066
C	-0.732832	-1.895762	3.693134
H	-1.651820	-1.477897	3.233738
H	-0.370355	-1.171958	4.452297
H	-1.016206	-2.817953	4.244012
C	1.609935	-2.765521	3.336397
H	1.372048	-3.759053	3.774671
H	1.959987	-2.106588	4.159605
H	2.449478	-2.897997	2.626938
C	0.303958	2.837012	0.016399
C	0.002674	3.665161	-1.112052
C	-0.948389	4.696219	-0.961503
H	-1.181574	5.339907	-1.825405
C	-1.599610	4.920405	0.258528
H	-2.331180	5.738972	0.356111
C	-1.310228	4.096317	1.354803
H	-1.819585	4.277367	2.315193
C	-0.367138	3.050999	1.258571
C	-0.057054	2.204452	2.493339

H	0.478306	1.299484	2.135331
C	0.895113	2.945001	3.458847
H	1.843420	3.220894	2.955566
H	1.146741	2.308243	4.333889
H	0.427653	3.878297	3.841132
C	-1.329597	1.742132	3.227792
H	-1.896539	2.595657	3.657194
H	-1.072883	1.067381	4.069570
H	-2.011451	1.190276	2.544946
C	0.685749	3.485229	-2.470572
H	1.296447	2.562597	-2.413441
C	1.638597	4.660666	-2.782546
H	1.082957	5.619575	-2.870387
H	2.164859	4.490002	-3.745774
H	2.407298	4.781863	-1.992636
C	-0.329675	3.285340	-3.615615
H	-1.006631	2.428760	-3.425008
H	0.204570	3.081593	-4.567417
H	-0.953777	4.191192	-3.775900
C	-6.212454	-0.298290	-1.632556
C	-7.695722	-0.724192	-1.604280
H	-8.345340	0.007819	-1.086138
H	-7.846126	-1.715886	-1.134249
H	-8.062702	-0.806421	-2.650733
C	-6.068381	1.002652	-2.455983
H	-5.017711	1.356307	-2.491369
H	-6.718284	1.824076	-2.097776
H	-6.370513	0.780985	-3.502292
C	-5.365987	-1.366250	-2.371494
H	-5.777216	-1.483097	-3.398052
H	-5.391599	-2.361463	-1.893990
H	-4.306591	-1.049762	-2.475692
C	-6.007106	1.680703	0.868380
C	-5.177174	2.796148	0.185325
H	-5.368775	2.890682	-0.897705
H	-4.086288	2.646981	0.327931
H	-5.442028	3.768489	0.654965
C	-5.655169	1.737188	2.372288
H	-5.848213	2.770371	2.733922
H	-4.583075	1.521898	2.557042
H	-6.270085	1.056724	2.992054
C	-7.514148	1.965882	0.691137
H	-8.155500	1.197682	1.165010
H	-7.808358	2.055481	-0.372608
H	-7.753650	2.938334	1.174598
C	-5.938363	-1.480212	1.323449
C	-7.382489	-1.378953	1.859923
H	-8.139131	-1.363670	1.051851
H	-7.538771	-0.487719	2.498658
H	-7.593789	-2.269172	2.492042
C	-5.762580	-2.828282	0.587148
H	-5.910247	-3.646331	1.325061
H	-4.741584	-2.946472	0.169691
H	-6.500958	-2.983528	-0.222364
C	-4.938549	-1.500302	2.505713
H	-5.165240	-2.381121	3.145101
H	-4.992447	-0.603711	3.147902
H	-3.893227	-1.607346	2.148497
O	-0.972317	-0.027989	-0.649229
C	-0.944637	-0.002377	-2.034417
O	0.308234	-0.001304	-2.434438
O	-1.979851	0.020124	-2.701063
Ag	-3.092519	-0.010819	-0.199262

TS5-Ag

E= -3483.0501757

P	-5.519862	-0.025924	0.116692
Al	0.972494	-0.005098	-0.627731
O	2.963411	0.026967	-0.902807
N	1.390128	-1.800698	-0.075675
N	1.332158	1.801729	-0.075084
C	3.628479	-1.155692	-0.548542
C	2.736883	-2.167340	-0.131529
C	3.344759	-3.401293	0.204991
H	2.698040	-4.238797	0.505944
C	4.755948	-3.551501	0.199468
C	5.576786	-2.450052	-0.137494
H	6.668274	-2.550848	-0.106099
C	5.016280	-1.206676	-0.518769
C	5.799843	0.067220	-0.916509
C	4.983049	1.292133	-0.438621
C	5.502692	2.523049	0.019082
H	6.592540	2.654779	0.075274
C	4.649282	3.590083	0.401648
C	3.245936	3.414196	0.364074
H	2.568835	4.218412	0.680104
C	2.675059	2.184709	-0.055156
C	3.594627	1.209275	-0.485982
C	5.285720	4.924383	0.866742
C	6.177069	4.666558	2.109476
H	5.579137	4.253203	2.948396
H	6.646677	5.612527	2.455679
H	6.992880	3.947038	1.892991
C	6.154727	5.506117	-0.278883
H	6.969910	4.813696	-0.572541
H	6.623200	6.464162	0.034138
H	5.540599	5.703578	-1.182180
C	4.226270	5.980707	1.247232
H	3.564315	6.231642	0.392866
H	4.727855	6.918336	1.566249
H	3.586585	5.643796	2.088933
C	5.905277	0.120019	-2.470269
H	4.902366	0.121184	-2.942229
H	6.461825	-0.762321	-2.849825
H	6.436812	1.040250	-2.791167
C	7.216838	0.066906	-0.313702
H	7.774344	0.974756	-0.620370
H	7.799765	-0.802393	-0.678975
H	7.191667	0.029921	0.794147
C	5.359389	-4.924138	0.591629
C	4.940053	-5.280487	2.041559
H	3.838242	-5.342360	2.150320
H	5.306750	-4.517785	2.759944
H	5.362852	-6.264229	2.339348
C	4.834992	-6.015110	-0.377297
H	5.121389	-5.787836	-1.425377
H	3.730112	-6.103073	-0.342138
H	5.258694	-7.007943	-0.112953
C	6.901909	-4.927514	0.525626
H	7.287764	-5.930224	0.805015
H	7.349793	-4.193497	1.227261
H	7.273596	-4.700607	-0.495191
C	0.437740	-2.838432	0.208799
C	-0.036767	-3.028584	1.544803
C	-0.956287	-4.068687	1.795914
H	-1.323704	-4.230124	2.821266
C	-1.404963	-4.911257	0.768536
H	-2.115428	-5.724557	0.989431
C	-0.941461	-4.713670	-0.538040
H	-1.297100	-5.374912	-1.345147
C	-0.024248	-3.686170	-0.846170
C	0.455790	-3.532693	-2.292115
H	1.054087	-2.601350	-2.344064
C	-0.713542	-3.376598	-3.286969
H	-1.340378	-4.293382	-3.335390
H	-0.322899	-3.190726	-4.309417
H	-1.370640	-2.523336	-3.024996
C	1.372584	-4.703537	-2.710328
H	2.252221	-4.791878	-2.041772
H	1.747441	-4.553534	-3.745153
H	0.827150	-5.672107	-2.688881
C	0.458343	-2.153855	2.700328
H	0.736051	-1.171408	2.259609
C	-0.611325	-1.902611	3.778739
H	-1.551277	-1.502898	3.346907
H	-0.238181	-1.170358	4.524474
H	-0.860203	-2.828166	4.340642
C	1.733337	-2.735033	3.352861
H	1.524083	-3.731200	3.799692
H	2.097142	-2.069023	4.164329
H	2.553595	-2.856335	2.619264
C	0.351255	2.841181	0.070597
C	0.027574	3.672222	-1.049397
C	-0.920326	4.702834	-0.877345
H	-1.171318	5.348320	-1.734925

C	-1.545992	4.924470	0.356538
H	-2.275709	5.742561	0.470926
C	-1.232353	4.099396	1.445321
H	-1.721252	4.278735	2.416584
C	-0.291711	3.054264	1.327526
C	0.044801	2.206249	2.554257
H	0.573369	1.302327	2.183806
C	1.016122	2.946780	3.500489
H	1.953508	3.224849	2.978211
H	1.286745	2.309063	4.369161
H	0.555601	3.878858	3.894059
C	-1.211909	1.740817	3.313557
H	-1.771791	2.593160	3.754411
H	-0.936203	1.067476	4.150540
H	-1.903455	1.187022	2.644222
C	0.683383	3.496081	-2.421856
H	1.297195	2.574686	-2.378847
C	1.627134	4.673950	-2.751478
H	1.067769	5.631767	-2.826505
H	2.134190	4.505895	-3.725421
H	2.411324	4.795607	-1.977081
C	-0.354916	3.296312	-3.546136
H	-1.026729	2.438997	-3.341339
H	0.159179	3.093787	-4.509223
H	-0.983570	4.201368	-3.692240
C	-6.534719	-0.369066	-1.514668
C	-8.000046	-0.791676	-1.277182
H	-8.580475	-0.034885	-0.714730
H	-8.082854	-1.759080	-0.744084
H	-8.499345	-0.923709	-2.262084
C	-6.504584	0.897444	-2.401160
H	-5.469194	1.252520	-2.582245
H	-7.105800	1.732492	-1.993128
H	-6.938214	0.637477	-3.390980
C	-5.793356	-1.470573	-2.314203
H	-6.308438	-1.600300	-3.291014
H	-5.788524	-2.454340	-1.812896
H	-4.742152	-1.184782	-2.528483
C	-6.020175	1.704607	0.858288
C	-5.293917	2.799248	0.037074
H	-5.617156	2.850505	-1.017393
H	-4.192200	2.662241	0.053682
H	-5.510396	3.787260	0.498666
C	-5.479975	1.819356	2.301925
H	-5.637083	2.864059	2.647432
H	-4.390618	1.619104	2.355944
H	-6.002780	1.158635	3.019751
C	-7.540165	1.975556	0.865460
H	-8.107309	1.220261	1.443411
H	-7.969304	2.026486	-0.154126
H	-7.726079	2.962741	1.342600
C	-5.859919	-1.434525	1.417728
C	-7.221558	-1.320074	2.136278
H	-8.078662	-1.339090	1.435652
H	-7.301459	-0.406028	2.756471
H	-7.338903	-2.186588	2.823439
C	-5.770577	-2.808248	0.714485
H	-5.803059	-3.599325	1.494565
H	-4.815680	-2.933182	0.163827
H	-6.612556	-2.999867	0.022196
C	-4.711830	-1.406604	2.456289
H	-4.847602	-2.259835	3.155989
H	-4.683728	-0.484143	3.062584
H	-3.722938	-1.529902	1.967934
O	-0.949380	-0.020851	-0.579398
C	-0.928225	0.004587	-1.943547
O	0.303684	0.001535	-2.390527
O	-1.991341	0.031359	-2.591255
Ag	-3.242662	0.006989	-0.503919

IM4-Ag

E= -3483.0509367

P	-5.793160	-0.024426	0.047644
Al	1.034910	-0.008757	-0.506105
O	3.003702	0.035724	-0.921073
N	1.504021	-1.805947	0.003911
N	1.427631	1.798653	0.036388
C	3.701969	-1.138231	-0.604252
C	2.849461	-2.156341	-0.125405
C	3.491202	-3.380194	0.183844
H	2.873263	-4.221573	0.530924
C	4.900624	-3.516345	0.088529
C	5.686074	-2.411600	-0.315543
H	6.778076	-2.502386	-0.358006
C	5.088756	-1.177781	-0.671467
C	5.829478	0.095739	-1.145109
C	5.041211	1.321040	-0.623170
C	5.584907	2.559981	-0.218432
H	6.674689	2.700550	-0.247579
C	4.754393	3.623425	0.219858
C	3.354321	3.435043	0.295480
H	2.698292	4.235799	0.660725
C	2.762169	2.196082	-0.065018
C	3.653879	1.226566	-0.563636
C	5.413179	4.967330	0.622024
C	6.415534	4.725938	1.780665
H	5.900689	4.306542	2.670162
H	6.900372	5.679568	2.082000
H	7.219432	4.017709	1.493767
C	6.168674	5.554035	-0.599031
H	6.961010	4.868523	-0.963100
H	6.653226	6.518254	-0.332684
H	5.473309	5.741369	-1.443727
C	4.379113	6.012141	1.093269
H	3.641886	6.254084	0.300157
H	4.895925	6.956125	1.366016
H	3.819743	5.668848	1.987880
C	5.815523	0.126289	-2.703021
H	4.779513	0.114253	-3.096851
H	6.348139	-0.757880	-3.111645
H	6.314041	1.045243	-3.076402
C	7.288493	0.114372	-0.653186
H	7.813563	1.021170	-1.015258
H	7.848284	-0.756555	-1.049531
H	7.349056	0.094141	0.453732
C	5.542619	-4.878792	0.454459
C	5.213603	-5.229480	1.928793
H	4.121012	-5.306058	2.101953
H	5.610505	-4.456308	2.619409
H	5.666709	-6.205070	2.208402
C	4.975257	-5.983103	-0.474414
H	5.197704	-5.760677	-1.538938
H	3.875291	-6.082333	-0.374379
H	5.424785	-6.969300	-0.228267
C	7.078592	-4.864736	0.298207
H	7.492585	-5.859807	0.564424
H	7.557254	-4.117751	0.964983
H	7.387390	-4.644394	-0.744767
C	0.581972	-2.860000	0.325619
C	0.181057	-3.073832	1.680735
C	-0.701829	-4.135677	1.969223
H	-1.009379	-4.314433	3.011787
C	-1.187899	-4.976156	0.958024
H	-1.868911	-5.806635	1.206359
C	-0.799047	-4.754009	-0.369252
H	-1.184128	-5.413679	-1.164146
C	0.080409	-3.705483	-0.713385
C	0.481119	-3.526519	-2.180325
H	1.051874	-2.579376	-2.252759
C	-0.741663	-3.389664	-3.111495
H	-1.348176	-4.320917	-3.138137
H	-0.410026	-3.182478	-4.150564
H	-1.403052	-2.555258	-2.803519
C	1.403900	-4.669583	-2.657880
H	2.317564	-4.742956	-2.034587
H	1.722871	-4.499782	-3.708340
H	0.883739	-5.651529	-2.620129
C	0.716580	-2.203367	2.819740
H	1.044340	-1.245607	2.360963
C	-0.348420	-1.876662	3.882773
H	-1.252693	-1.420051	3.431401
H	0.059731	-1.163870	4.629062
H	-0.665599	-2.780251	4.446216
C	1.958819	-2.838286	3.484376
H	1.701243	-3.815805	3.947088

H	2.357655	-2.178815	4.284724
H	2.770849	-3.011419	2.751288
C	0.453377	2.824875	0.284315
C	0.035869	3.679351	-0.784749
C	-0.899670	4.700160	-0.512449
H	-1.219171	5.366313	-1.330712
C	-1.429101	4.882830	0.770897
H	-2.152940	5.691351	0.964437
C	-1.030299	4.029017	1.809388
H	-1.449991	4.176517	2.816607
C	-0.093435	2.996686	1.593637
C	0.347969	2.110915	2.760663
H	0.642284	1.131658	2.324711
C	1.597514	2.686357	3.465298
H	2.442509	2.813165	2.760201
H	1.933756	2.014314	4.283767
H	1.373160	3.679564	3.911400
C	-0.770887	1.850912	3.785333
H	-1.051033	2.772032	4.339878
H	-0.435933	1.112765	4.542100
H	-1.686042	1.451791	3.301061
C	0.570235	3.533391	-2.212472
H	1.196970	2.620101	-2.239472
C	1.462689	4.729058	-2.612001
H	0.886419	5.679771	-2.620392
H	1.878151	4.581350	-3.631578
H	2.314729	4.851509	-1.913303
C	-0.564746	3.332821	-3.239135
H	-1.202520	2.463688	-2.982285
H	-0.140693	3.147281	-4.248382
H	-1.215947	4.230705	-3.313971
C	-7.170770	-0.746472	-1.125609
C	-8.454641	-1.190715	-0.392865
H	-8.933366	-0.366696	0.170758
H	-8.275721	-2.030801	0.306245
H	-9.192438	-1.548083	-1.144265
C	-7.533887	0.309838	-2.194530
H	-6.638501	0.679113	-2.735845
H	-8.085324	1.176911	-1.783467
H	-8.196730	-0.170762	-2.946250
C	-6.563209	-1.947275	-1.893710
H	-7.308586	-2.303213	-2.637964
H	-6.308531	-2.804568	-1.246276
H	-5.647887	-1.654068	-2.449850
C	-6.241313	1.797360	0.569643
C	-5.899868	2.731887	-0.618144
H	-6.521681	2.557597	-1.513814
H	-4.833571	2.641340	-0.913716
H	-6.067408	3.783085	-0.297480
C	-5.323725	2.230279	1.736176
H	-5.481954	3.315709	1.914658
H	-4.248951	2.094469	1.497914
H	-5.550244	1.709153	2.685876
C	-7.715862	1.991223	0.983334
H	-8.010664	1.345934	1.833515
H	-8.421536	1.809824	0.149114
H	-7.861996	3.045622	1.304878
C	-5.592814	-1.149487	1.622617
C	-6.702470	-0.961999	2.678994
H	-7.713903	-1.177922	2.283304
H	-6.709555	0.057019	3.112692
H	-6.519889	-1.667728	3.518633
C	-5.550746	-2.631680	1.184955
H	-5.276876	-3.244898	2.070211
H	-4.776518	-2.816850	0.412470
H	-6.525017	-3.007689	0.817859
C	-4.209439	-0.843951	2.250132
H	-4.040457	-1.556295	3.086356
H	-4.127457	0.175671	2.665416
H	-3.383078	-0.984595	1.522174
O	-0.871587	-0.040049	-0.343510
C	-0.923936	-0.011804	-1.665132
O	0.258405	0.004539	-2.232010
O	-2.013274	0.002464	-2.323376
Ag	-3.745972	-0.002230	-1.113661

6-Ag

E= -6966.153456

Ag	-0.385122	0.727043	1.708602
Al	-4.934279	-0.184631	-0.417632
P	-0.365078	1.637786	3.912199
O	-6.397200	0.354955	0.846137
O	-3.291568	-0.576554	-1.319606
O	-3.459656	0.199330	0.725545
N	-5.663616	-1.932431	-0.042268
O	-1.379925	-0.161378	-0.134925
N	-5.545580	1.410736	-1.321502
C	-7.093245	1.516895	0.485852
C	-7.205138	-0.700705	1.293219
C	-4.987589	2.042949	-2.487748
C	-6.770751	-1.950800	0.814056
C	-9.104590	-1.577234	2.411504
H	-10.021693	-1.441277	3.001929
C	-5.090873	-3.217806	-0.346379
C	-7.228576	3.302879	-1.086977
H	-6.857848	3.826046	-1.980631
C	-5.509582	1.748637	-3.786189
C	-6.586045	2.103285	-0.692820
C	-8.226358	1.906683	1.188800
C	-8.354708	-0.442063	2.034610
C	-8.854801	3.091097	0.733982
H	-9.747190	3.453280	1.258340
C	-8.696378	-2.885441	2.044323
C	-5.419462	-3.889258	-1.564435
C	-8.673514	1.031822	2.384704
C	-4.232920	-3.850005	0.607587
C	-7.539198	-3.062828	1.250144
H	-7.227001	-4.065495	0.931459
C	-4.908795	-5.186304	-1.784522
H	-5.165948	-5.716656	-2.714290
C	-2.646669	-0.182056	-0.241756
C	-10.169310	1.225268	2.694805
H	-10.382406	2.279523	2.963235
H	-10.473639	0.607805	3.563811
H	-10.806410	0.949600	1.830376
C	-8.350513	3.805375	-0.377895
C	-9.549207	-4.092013	2.511994
C	-7.837494	1.434800	3.636190
H	-6.751990	1.309078	3.449705
H	-8.112801	0.802240	4.505951
H	-8.021065	2.497966	3.897760
C	-3.750682	-5.148036	0.338238
H	-3.096264	-5.642195	1.074902
C	-4.989009	2.440523	-4.900819
H	-5.391917	2.227860	-5.903613
C	-3.357182	3.370093	-0.974220
H	-3.746032	2.631026	-0.245309
C	-4.089521	-5.821457	-0.841416
H	-3.712524	-6.839829	-1.029462
C	-3.947144	3.011906	-2.339473
C	-6.336823	-3.253380	-2.611419
H	-6.238713	-2.151807	-2.497693
C	-6.637768	0.735383	-3.994831
H	-6.641516	0.072318	-3.102894
C	0.441596	3.410680	3.873007
C	-3.825822	-3.174698	1.919167
H	-4.210991	-2.135804	1.890380
C	0.712223	0.448443	5.018845
C	-2.171314	1.758107	4.634141
C	-3.979201	3.402060	-4.759468
H	-3.596337	3.939109	-5.642473
C	-3.462744	3.675304	-3.486489
H	-2.665624	4.428412	-3.376067
C	-6.427252	-0.145905	-5.240541
H	-6.499968	0.440470	-6.181209
H	-7.212760	-0.928004	-5.293425
H	-5.439584	-0.649814	-5.229745
C	-9.005587	5.124697	-0.860286
C	-5.942134	-3.617339	-4.055044
H	-4.873820	-3.396562	-4.257748
H	-6.554143	-3.043186	-4.778915
H	-6.113777	-4.692640	-4.274344
C	-7.820515	-3.604379	-2.357633
H	-7.982861	-4.701048	-2.439439
H	-8.475911	-3.108248	-3.105120

H	-8.151245	-3.285978	-1.349450
C	2.202284	0.619899	4.645942
H	2.385403	0.520820	3.556113
H	2.778002	-0.189529	5.144696
H	2.625743	1.582607	4.991704
C	-3.812635	4.769851	-0.506380
H	-3.455878	5.562328	-1.199575
H	-3.404634	4.998234	0.501535
H	-4.917250	4.839245	-0.447544
C	-8.020946	1.421591	-4.060517
H	-8.234123	2.005479	-3.144130
H	-8.827229	0.666630	-4.179392
H	-8.075297	2.114124	-4.928481
C	-3.096173	2.305286	3.519264
H	-2.859914	3.341837	3.220948
H	-4.140224	2.306625	3.902823
H	-3.075448	1.662177	2.614992
C	-1.818375	3.264955	-0.962703
H	-1.473467	2.274206	-1.320438
H	-1.428763	3.399927	0.069159
H	-1.341209	4.042707	-1.596206
C	-10.988977	-3.954138	1.951950
H	-11.482288	-3.023256	2.298807
H	-11.618331	-4.809611	2.279365
H	-10.983325	-3.937503	0.842118
C	1.608528	3.362584	2.858471
H	2.401953	2.640729	3.114979
H	2.075919	4.368831	2.795643
H	1.248749	3.101058	1.842166
C	-2.292082	-3.088818	2.063743
H	-1.826592	-4.094377	2.144218
H	-2.020578	-2.528695	2.982981
H	-1.826014	-2.567020	1.203707
C	-9.599440	-4.116289	4.062075
H	-8.580720	-4.218169	4.491139
H	-10.209500	-4.973747	4.419530
H	-10.047022	-3.189925	4.476614
C	0.339189	-1.007537	4.645400
H	-0.695842	-1.283540	4.914699
H	1.024693	-1.696837	5.183944
H	0.476933	-1.194523	3.559774
C	-2.683491	0.336332	4.957145
H	-2.595905	-0.340510	4.083003
H	-3.763918	0.405094	5.209145
H	-2.175257	-0.125679	5.825417
C	-4.452444	-3.876091	3.144194
H	-5.558729	-3.898691	3.075780
H	-4.180713	-3.344681	4.081258
H	-4.093234	-4.923820	3.238503
C	-0.571740	4.437809	3.319234
H	-0.995503	4.119934	2.344828
H	-0.031957	5.393696	3.144584
H	-1.404962	4.654779	4.014992
C	-8.974468	-5.441229	2.030118
H	-8.941806	-5.507609	0.923130
H	-9.613087	-6.273183	2.394043
H	-7.948468	-5.616994	2.414206
C	0.536762	0.651811	6.538881
H	0.790872	1.678487	6.866283
H	1.223004	-0.042214	7.072023
H	-0.489385	0.420323	6.886120
C	-2.291318	2.638229	5.897081
H	-1.653181	2.285905	6.730359
H	-3.344602	2.610376	6.253664
H	-2.048231	3.700638	5.700197
C	0.951167	3.905923	5.244043
H	0.149340	3.961549	6.006096
H	1.362019	4.932219	5.122290
H	1.769996	3.278652	5.645737
C	-10.212281	5.533609	0.011862
H	-11.019304	4.772126	-0.011852
H	-10.643282	6.484525	-0.365288
H	-9.922704	5.696965	1.070732
C	-7.960179	6.268532	-0.801150
H	-7.586901	6.413283	0.234266
H	-8.409855	7.226329	-1.141005
H	-7.084261	6.061333	-1.448927

C	-9.502716	4.959305	-2.319713
H	-8.674683	4.711659	-3.014333
H	-9.972387	5.899803	-2.680095
H	-10.257956	4.149165	-2.393341
Ag	0.385085	-0.727156	-1.708561
Al	4.934262	0.184641	0.417616
P	0.365079	-1.637938	-3.912145
O	6.397190	-0.354859	-0.846211
O	3.291552	0.576492	1.319617
O	3.459647	-0.199306	-0.725567
N	5.663556	1.932477	0.042325
O	1.379912	0.161322	0.134932
N	5.545619	-1.410741	1.321403
C	7.093293	-1.516773	-0.485947
C	7.205093	0.700852	-1.293245
C	4.987669	-2.043001	2.487642
C	6.770675	1.950911	-0.814017
C	9.104526	1.577490	-2.411483
H	10.021637	1.441586	-3.001908
C	5.090747	3.217817	0.346458
C	7.228715	-3.302789	1.086837
H	6.858010	-3.826001	1.980476
C	5.509726	-1.748762	3.786072
C	6.586124	-2.103217	0.692710
C	8.226422	-1.906493	-1.188909
C	8.354674	0.442280	-2.034645
C	8.854922	-3.090888	-0.734122
H	9.747325	-3.453013	-1.258495
C	8.696277	2.885667	-2.044239
C	5.419192	3.889193	1.564598
C	8.673533	-1.031580	-2.384791
C	4.232866	3.850057	-0.607547
C	7.539092	3.062982	-1.250052
H	7.226870	4.065623	-0.931308
C	4.908476	5.186214	1.784715
H	5.165514	5.716511	2.714545
C	2.646657	0.182018	0.241756
C	10.169334	-1.224959	-2.694911
H	10.382469	-2.279198	-2.963372
H	10.473630	-0.607459	-3.563903
H	10.806431	-0.949289	-1.830481
C	8.350671	-3.805218	0.377738
C	9.549073	4.092287	-2.511848
C	7.837517	-1.434550	-3.636281
H	6.752010	-1.308877	-3.449780
H	8.112790	-0.801951	-4.506024
H	8.021127	-2.497701	-3.897888
C	3.750582	5.148065	-0.338166
H	3.096226	5.642258	-1.074862
C	4.989189	-2.440683	4.900697
H	5.392152	-2.228073	5.903482
C	3.357219	-3.370082	0.974113
H	3.746049	-2.630981	0.245226
C	4.089294	5.821418	0.841562
H	3.712263	6.839772	1.029636
C	3.947213	-3.011945	2.339365
C	6.336439	3.253242	2.611640
H	6.238099	2.151674	2.498038
C	6.637962	-0.735565	3.994711
H	6.641912	-0.072666	3.102654
C	-0.441566	-3.410842	-3.872925
C	3.825871	3.174825	-1.919196
H	4.211056	2.135935	-1.890459
C	-0.712196	-0.448606	-5.018832
C	2.171328	-1.758312	-4.634043
C	3.979354	-3.402191	4.759347
H	3.596511	-3.939267	5.642344
C	3.462846	-3.675379	3.486374
H	2.665716	-4.428476	3.375955
C	6.427300	0.145968	5.240221
H	6.499768	-0.440244	6.181009
H	7.212876	0.928000	5.293113
H	5.439683	0.649971	5.229138
C	9.005796	-5.124533	0.860081
C	5.941856	3.617436	4.055234
H	4.873487	3.396951	4.257975
H	6.553727	3.043199	4.779154

H	6.113775	4.692716	4.274430
C	7.820201	3.603905	2.357801
H	7.982768	4.700549	2.439499
H	8.475500	3.107714	3.105335
H	8.150861	3.285340	1.349649
C	-2.202270	-0.620036	-4.645972
H	-2.385422	-0.520928	-3.556151
H	-2.777958	0.189392	-5.144761
H	-2.625734	-1.582744	-4.991726
C	3.812682	-4.769816	0.506214
H	3.455939	-5.562325	1.199380
H	3.404679	-4.998165	-0.501708
H	4.917298	-4.839195	0.447369
C	8.021063	-1.421892	4.060748
H	8.234326	-2.005959	3.144494
H	8.827401	-0.666988	4.179614
H	8.075215	-2.114278	4.928842
C	3.096138	-2.305461	-3.519114
H	2.859823	-3.341987	-3.220748
H	4.140198	-2.306868	-3.902648
H	3.075418	-1.662300	-2.614879
C	1.818412	-3.264956	0.962629
H	1.473502	-2.274216	1.320387
H	1.428784	-3.399914	-0.069228
H	1.341262	-4.042722	1.596127
C	10.988853	3.954415	-1.951831
H	11.482180	3.023562	-2.298741
H	11.618182	4.809918	-2.279212
H	10.983215	3.937724	-0.842000
C	-1.608483	-3.362766	-2.858367
H	-2.401922	-2.640920	-3.114853
H	-2.075857	-4.369021	-2.795534
H	-1.248688	-3.101238	-1.842068
C	2.292140	3.088930	-2.063873
H	1.826639	4.094488	-2.144300
H	2.020702	2.528874	-2.983171
H	1.826027	2.567055	-1.203908
C	9.599285	4.116651	-4.061929
H	8.580557	4.218525	-4.490975
H	10.209317	4.974147	-4.419344
H	10.046889	3.190323	-4.476524
C	-0.339173	1.007378	-4.645390
H	0.695864	1.283382	-4.914665
H	-1.024665	1.696675	-5.183953
H	-0.476943	1.194369	-3.559768
C	2.683536	-0.336561	-4.957106
H	2.595924	0.340329	-4.083004
H	3.763973	-0.405352	-5.209056
H	2.175345	0.125407	-5.825425
C	4.452551	3.876313	-3.144140
H	5.558832	3.898929	-3.075659
H	4.180885	3.344961	-4.081256
H	4.093325	4.924041	-3.238400
C	0.571812	-4.437932	-3.319156
H	0.995579	-4.120029	-2.344761
H	0.032070	-5.393838	-3.144488
H	1.405034	-4.654875	-4.014925
C	8.974309	5.441462	-2.029888
H	8.941650	5.507772	-0.922896
H	9.612911	6.273451	-2.393764
H	7.948304	5.617232	-2.413961
C	-0.536698	-0.651992	-6.538861
H	-0.790812	-1.678671	-6.866252
H	-1.222918	0.042032	-7.072030
H	0.489462	-0.420521	-6.886076
C	2.291346	-2.638515	-5.896925
H	1.653238	-2.286233	-6.730242
H	3.344640	-2.610711	-6.253481
H	2.048227	-3.700905	-5.699974
C	-0.951141	-3.906126	-5.243947
H	-0.149318	-3.961759	-6.006004
H	-1.361975	-4.932425	-5.122164
H	-1.769983	-3.278879	-5.645651
C	10.212548	-5.533333	-0.012041
H	11.019525	-4.771804	0.011741
H	10.643588	-6.484242	0.365079
H	9.923022	-5.696656	-1.070931
C	7.960446	-6.268416	0.800834
H	7.587219	-6.413125	-0.234607
H	8.410154	-7.226211	1.140652
H	7.084491	-6.061293	1.448585
C	9.502856	-4.959207	2.319537
H	8.674781	-4.711663	3.014144
H	9.972571	-5.899700	2.679875
H	10.258044	-4.149025	2.393249

3-Cu

E= -4712.688482

P	4.588365	-0.037482	-0.606855
Al	-0.101985	-0.005665	-0.098547
O	-2.040202	0.012144	-0.961051
N	-0.755853	1.808837	0.332267
N	-0.791393	-1.804043	0.342236
C	-2.774341	1.190441	-0.751014
C	-2.047658	2.171086	-0.045269
C	-2.733338	3.390010	0.183826
H	-2.198920	4.199532	0.702830
C	-4.085160	3.559758	-0.207016
C	-4.765689	2.494375	-0.841447
H	-5.821302	2.605506	-1.116977
C	-4.112010	1.271640	-1.125123
C	-4.741496	0.036345	-1.807590
C	-4.138582	-1.207713	-1.117309
C	-4.812637	-2.411782	-0.821955
H	-5.873016	-2.510558	-1.094032
C	-4.152210	-3.489681	-0.178058
C	-2.801390	-3.345718	0.211172
H	-2.278459	-4.156308	0.735115
C	-2.091307	-2.139305	-0.032828
C	-2.796444	-1.151431	-0.744122
C	-4.943428	-4.792399	0.100516
C	-6.163587	-4.472278	1.002994
H	-5.837736	-4.044341	1.973946
H	-6.746587	-5.395040	1.212347
H	-6.850780	-3.742257	0.528626
C	-5.438861	-5.390169	-1.242170
H	-6.099537	-4.686932	-1.789227
H	-6.014262	-6.324538	-1.065512
H	-4.584597	-5.634193	-1.907709
C	-4.086806	-5.859797	0.814288
H	-3.203418	-6.155200	0.211251
H	-4.692240	-6.774089	0.987541
H	-3.724591	-5.508700	1.802458
C	-4.325980	0.026877	-3.309213
H	-3.223416	0.014490	-3.422272
H	-4.713577	0.931282	-3.823271
H	-4.733328	-0.872035	-3.817625
C	-6.277368	0.052655	-1.712242
H	-6.711369	-0.834020	-2.216945
H	-6.693100	0.944986	-2.222235
H	-6.624190	0.059402	-0.659205
C	-4.788765	4.904762	0.104395
C	-4.812661	5.129460	1.639022
H	-3.790261	5.161652	2.067311
H	-5.365618	4.314309	2.150844
H	-5.311975	6.091319	1.885668
C	-4.015296	6.065741	-0.572050
H	-3.982845	5.933704	-1.673811
H	-2.969559	6.132829	-0.208900
H	-4.506733	7.039168	-0.357656
C	-6.244820	4.937906	-0.407836
H	-6.704353	5.920570	-0.172059
H	-6.871648	4.156839	0.070243
H	-6.300272	4.799498	-1.507660
C	0.121400	2.876036	0.708045
C	0.499358	3.056345	2.074563
C	1.346101	4.134431	2.409372
H	1.637028	4.287788	3.459854
C	1.818128	5.023514	1.433632
H	2.471094	5.864078	1.719376
C	1.455604	4.834003	0.093050
H	1.835995	5.528649	-0.673818
C	0.613246	3.771977	-0.294785
C	0.280325	3.583048	-1.777593
H	-0.520789	2.821254	-1.843672

C	1.496709	3.018382	-2.539111
H	2.334622	3.748460	-2.562590
H	1.233665	2.770376	-3.589763
H	1.858737	2.088399	-2.044841
C	-0.254910	4.864711	-2.445421
H	-1.148271	5.252446	-1.915425
H	-0.547055	4.660175	-3.497220
H	0.506903	5.673387	-2.468775
C	-0.046989	2.135430	3.167457
H	-0.173619	1.132595	2.702895
C	0.897849	1.976396	4.371262
H	1.915875	1.662631	4.060654
H	0.502831	1.210483	5.069945
H	0.994882	2.917832	4.953221
C	-1.447041	2.593244	3.634621
H	-1.394733	3.603297	4.095699
H	-1.858172	1.893792	4.393572
H	-2.163971	2.640086	2.791336
C	0.059646	-2.887030	0.731421
C	0.511128	-3.821667	-0.254951
C	1.316329	-4.905357	0.150290
H	1.663182	-5.631281	-0.603247
C	1.690377	-5.073863	1.490614
H	2.316506	-5.930146	1.789382
C	1.265439	-4.142843	2.448523
H	1.564612	-4.279700	3.499094
C	0.448589	-3.046931	2.097343
C	-0.064847	-2.090932	3.175556
H	-0.185629	-1.097758	2.689397
C	-1.463690	-2.518461	3.674269
H	-2.192161	-2.578229	2.841480
H	-1.855773	-1.794374	4.420109
H	-1.417442	-3.517064	4.160233
C	0.902852	-1.916047	4.358588
H	0.999514	-2.844609	4.960878
H	0.531172	-1.128293	5.045538
H	1.918483	-1.622871	4.020991
C	0.180878	-3.648194	-1.740382
H	-0.607125	-2.874045	-1.819041
C	-0.376069	-4.929810	-2.390154
H	0.373525	-5.749852	-2.409766
H	-0.673490	-4.733512	-3.441954
H	-1.270749	-5.298546	-1.848040
C	1.408712	-3.114756	-2.508277
H	1.771466	-2.170297	-2.042535
H	1.156152	-2.901613	-3.569269
H	2.243381	-3.848775	-2.496202
C	5.354142	-0.302163	-2.380207
C	6.846489	0.068341	-2.509766
H	7.491268	-0.527976	-1.834998
H	7.041317	1.140277	-2.313494
H	7.181949	-0.135903	-3.551080
C	5.171292	-1.774320	-2.813531
H	4.119391	-2.109809	-2.719661
H	5.818427	-2.475952	-2.252986
H	5.451298	-1.859419	-3.886461
C	4.506986	0.544693	-3.362746
H	4.852984	0.346299	-4.401194
H	4.587051	1.632485	-3.189117
H	3.433131	0.269500	-3.306079
C	5.150702	-1.491121	0.558867
C	4.257006	-2.706918	0.213964
H	4.476724	-3.148927	-0.773800
H	3.179868	-2.434957	0.236030
H	4.412971	-3.501064	0.975355
C	4.811141	-1.115144	2.019799
H	4.964310	-2.013732	2.656157
H	3.748615	-0.813501	2.126638
H	5.453825	-0.311527	2.427572
C	6.639117	-1.884575	0.468639
H	7.320407	-1.049252	0.722081
H	6.918652	-2.266229	-0.533079
H	6.839752	-2.705801	1.192306
C	5.233593	1.651793	0.122195
C	6.673817	1.627628	0.673815
H	7.422395	1.352328	-0.094689
H	6.790886	0.938067	1.532467
H	6.936017	2.645096	1.040299
C	5.127814	2.754527	-0.955398
H	5.335046	3.733758	-0.471654
H	4.108186	2.813792	-1.383588
H	5.858023	2.637660	-1.779194
C	4.240273	2.053141	1.240921
H	4.481297	3.080819	1.588894
H	4.277925	1.387763	2.121755
H	3.193773	2.066501	0.867225
Cu	2.256354	-0.021259	-0.540628

TS1-Cu

E= -4901.372474

P	4.536783	-0.066510	-0.369133
Al	-0.132618	-0.020407	-0.164701
O	-2.057483	0.045684	-0.919411
N	-0.698013	1.803972	0.340272
N	-0.814400	-1.798586	0.345735
C	-2.743667	1.255289	-0.732755
C	-1.974989	2.215685	-0.046396
C	-2.613568	3.462630	0.168602
H	-2.049587	4.259197	0.675898
C	-3.962157	3.675283	-0.215075
C	-4.688582	2.624456	-0.821639
H	-5.742616	2.768475	-1.087541
C	-4.082679	1.373816	-1.088208
C	-4.764627	0.140760	-1.721636
C	-4.198038	-1.099338	-0.993823
C	-4.910476	-2.261909	-0.631301
H	-5.981795	-2.326907	-0.868396
C	-4.275125	-3.339122	0.038128
C	-2.903775	-3.243345	0.368080
H	-2.399329	-4.055439	0.907287
C	-2.148984	-2.084881	0.042572
C	-2.844926	-1.086695	-0.660206
C	-5.114026	-4.588199	0.408502
C	-6.280790	-4.167328	1.339759
H	-5.896747	-3.709462	2.275147
H	-6.896501	-5.050114	1.616782
H	-6.952390	-3.428954	0.856152
C	-5.690018	-5.223589	-0.883842
H	-6.339015	-4.515901	-1.439063
H	-6.300486	-6.119773	-0.640129
H	-4.875709	-5.539693	-1.568792
C	-4.280250	-5.661010	1.141176
H	-3.436205	-6.027530	0.521100
H	-4.920324	-6.535684	1.381318
H	-3.863613	-5.281622	2.096955
C	-4.363356	0.062997	-3.225587
H	-3.263365	0.011045	-3.349121
H	-4.728031	0.960596	-3.767592
H	-4.806758	-0.839138	-3.696787
C	-6.297309	0.216387	-1.610958
H	-6.768341	-0.671448	-2.079163
H	-6.686080	1.104505	-2.148774
H	-6.632425	0.273533	-0.555562
C	-4.613271	5.050934	0.077048
C	-4.620445	5.303076	1.607502
H	-3.595384	5.304257	2.030633
H	-5.201468	4.518906	2.136343
H	-5.081528	6.287439	1.839225
C	-3.801768	6.171057	-0.623052
H	-3.776472	6.017373	-1.722124
H	-2.753562	6.209079	-0.262849
H	-4.258464	7.164815	-0.425980
C	-6.070060	5.129422	-0.428352
H	-6.490963	6.132715	-0.207945
H	-6.723052	4.381304	0.067044
H	-6.136943	4.973460	-1.525155
C	0.166742	2.836228	0.832618
C	0.378025	2.992148	2.238567
C	1.204610	4.043023	2.689283
H	1.369088	4.175808	3.769783
C	1.815257	4.929889	1.791638
H	2.451900	5.747466	2.166779
C	1.605571	4.771792	0.415433
H	2.083925	5.472373	-0.288945
C	0.784320	3.741028	-0.087634

C	0.582522	3.624302	-1.600396
H	-0.149131	2.810505	-1.774816
C	1.890506	3.213131	-2.303380
H	2.684040	3.981978	-2.183058
H	1.731286	3.063139	-3.392551
H	2.273672	2.258143	-1.879558
C	-0.000703	4.910693	-2.219971
H	-0.961317	5.188386	-1.741857
H	-0.191927	4.766628	-3.304454
H	0.694555	5.771624	-2.118054
C	-0.317018	2.078110	3.249969
H	-0.480147	1.104889	2.737903
C	0.519062	1.812779	4.514867
H	1.532238	1.431650	4.271797
H	0.013712	1.061053	5.155998
H	0.639598	2.726843	5.134671
C	-1.711247	2.623095	3.634754
H	-1.620659	3.610071	4.138119
H	-2.224126	1.930220	4.335466
H	-2.360749	2.751708	2.746871
C	-0.005438	-2.918771	0.727747
C	0.341906	-3.911912	-0.245178
C	1.087424	-5.036496	0.166115
H	1.348956	-5.808510	-0.576323
C	1.505697	-5.190731	1.493579
H	2.083589	-6.079133	1.795686
C	1.182871	-4.204870	2.436949
H	1.513518	-4.332803	3.478724
C	0.426208	-3.067536	2.083875
C	0.022047	-2.050216	3.154427
H	0.032104	-1.049387	2.666735
C	-1.423703	-2.295909	3.642665
H	-2.150669	-2.266218	2.807908
H	-1.724141	-1.526504	4.385625
H	-1.504935	-3.290479	4.131935
C	0.983848	-1.995060	4.354355
H	0.940187	-2.923037	4.963600
H	0.708645	-1.157948	5.026967
H	2.037162	-1.845970	4.039828
C	-0.065498	-3.804604	-1.718771
H	-0.630435	-2.858887	-1.841125
C	-0.992657	-4.959909	-2.153113
H	-0.476982	-5.942303	-2.089043
H	-1.317179	-4.822951	-3.206408
H	-1.902428	-5.006989	-1.522258
C	1.164852	-3.723145	-2.646945
H	1.817132	-2.868893	-2.378054
H	0.848806	-3.592040	-3.703745
H	1.780177	-4.646994	-2.596918
C	5.683076	0.065290	-1.945105
C	7.173956	0.322907	-1.640518
H	7.626697	-0.463740	-1.007014
H	7.345092	1.301658	-1.151564
H	7.738239	0.339402	-2.599558
C	5.556186	-1.233820	-2.774404
H	4.499960	-1.479632	-3.001624
H	6.033732	-2.106936	-2.289707
H	6.074532	-1.077977	-3.745880
C	5.134335	1.204657	-2.839207
H	5.720084	1.223022	-3.784772
H	5.222862	2.204721	-2.380125
H	4.074410	1.033534	-3.106594
C	4.854878	-1.783310	0.493692
C	4.047034	-2.835837	-0.301632
H	4.413277	-2.993347	-1.331411
H	2.973196	-2.555427	-0.352357
H	4.100229	-3.810903	0.228775
C	4.226347	-1.750195	1.904664
H	4.228552	-2.783275	2.314045
H	3.168231	-1.419603	1.864631
H	4.779458	-1.109739	2.617992
C	6.328719	-2.224781	0.602369
H	6.955382	-1.503266	1.161628
H	6.796809	-2.396756	-0.386801
H	6.372083	-3.192448	1.149866
C	5.009985	1.361053	0.872731
C	6.306009	1.127059	1.677493
H	7.197447	1.008798	1.031425
H	6.242893	0.245207	2.344230
H	6.486245	2.010623	2.329315
C	5.136633	2.697193	0.106024
H	5.227225	3.516853	0.851267
H	4.236700	2.913479	-0.502898
H	6.032196	2.745447	-0.542932
C	3.813845	1.533375	1.839526
H	3.995908	2.421213	2.482796
H	3.658667	0.665840	2.505261
H	2.868498	1.714234	1.286627
Cu	2.202647	-0.046519	-0.784289
C	0.975626	-0.141916	-3.063434
O	-0.188806	0.011442	-3.250718
O	2.132268	-0.317855	-3.321554

IM1-Cu

E= -4901.409109

P	4.657120	0.018686	-0.030657
Al	-0.387077	0.003288	-0.690645
O	-2.363729	-0.002106	-1.010274
N	-0.806923	1.793846	-0.071063
N	-0.792758	-1.780094	-0.037600
C	-3.028546	1.183886	-0.690266
C	-2.150832	2.174464	-0.205631
C	-2.763218	3.415531	0.097166
H	-2.129077	4.243676	0.446014
C	-4.168082	3.590932	-0.014732
C	-4.981174	2.504234	-0.413109
H	-6.070144	2.623683	-0.462125
C	-4.413743	1.254616	-0.759446
C	-5.178741	-0.008636	-1.224400
C	-4.414081	-1.246798	-0.694816
C	-4.977164	-2.471706	-0.277439
H	-6.069045	-2.595946	-0.302222
C	-4.160933	-3.542696	0.168996
C	-2.757832	-3.374517	0.240994
H	-2.118749	-4.181767	0.620271
C	-2.144368	-2.149645	-0.135088
C	-3.024742	-1.176614	-0.640621
C	-4.837927	-4.873429	0.584681
C	-5.837434	-4.606848	1.740287
H	-5.317588	-4.186149	2.626237
H	-6.335181	-5.550864	2.050351
H	-6.631944	-3.890993	1.446220
C	-5.600836	-5.461315	-0.631240
H	-6.383595	-4.768676	-1.002489
H	-6.098768	-6.415953	-0.355635
H	-4.907858	-5.666797	-1.473660
C	-3.818557	-5.927629	1.066737
H	-3.084589	-6.188109	0.276535
H	-4.348708	-6.861360	1.348660
H	-3.254883	-5.583427	1.958314
C	-5.165064	-0.050021	-2.782123
H	-4.129138	-0.060528	-3.176152
H	-5.681009	0.841196	-3.196603
H	-5.681412	-0.961636	-3.149005
C	-6.637091	0.004527	-0.730713
H	-7.180424	-0.893928	-1.086539
H	-7.180038	0.883788	-1.132014
H	-6.696103	0.033099	0.376135
C	-4.774869	4.979893	0.311467
C	-4.432313	5.377028	1.770279
H	-3.338023	5.440691	1.937443
H	-4.842046	4.639001	2.491059
H	-4.864855	6.371088	2.014456
C	-4.185158	6.036260	-0.658731
H	-4.415074	5.779885	-1.713952
H	-3.082946	6.113820	-0.563817
H	-4.611512	7.040768	-0.448253
C	-6.311565	4.997573	0.161543
H	-6.700404	6.009442	0.400588
H	-6.804626	4.281552	0.851444
H	-6.630808	4.755678	-0.873379
C	0.085682	2.815737	0.401779
C	0.285703	3.001292	1.806927
C	1.131228	4.045643	2.238591
H	1.286228	4.202192	3.318287

C	1.765129	4.898402	1.324799
H	2.415455	5.712025	1.684982
C	1.553820	4.719159	-0.048971
H	2.038831	5.402410	-0.765074
C	0.713175	3.695490	-0.536133
C	0.452191	3.597520	-2.040999
H	-0.084849	2.644540	-2.218676
C	1.753458	3.554418	-2.863727
H	2.313683	4.512951	-2.809875
H	1.530208	3.360394	-3.933333
H	2.422142	2.745717	-2.511340
C	-0.461368	4.743554	-2.530257
H	-1.432577	4.748359	-1.996789
H	-0.671851	4.634603	-3.615356
H	0.019153	5.734523	-2.378360
C	-0.440544	2.147606	2.851280
H	-0.838266	1.257204	2.319249
C	0.484422	1.654117	3.979697
H	1.342128	1.071913	3.589944
H	-0.080687	1.003107	4.678807
H	0.889676	2.494660	4.582494
C	-1.646279	2.900142	3.458646
H	-1.312475	3.812641	3.998777
H	-2.181775	2.254245	4.186677
H	-2.371684	3.207254	2.681310
C	0.104480	-2.845790	0.320109
C	0.523071	-3.778804	-0.681906
C	1.307085	-4.886487	-0.294429
H	1.621888	-5.614663	-1.059617
C	1.685874	-5.080892	1.038850
H	2.288449	-5.958602	1.323903
C	1.294619	-4.149812	2.011482
H	1.600279	-4.309299	3.056450
C	0.503106	-3.028006	1.683847
C	0.049401	-2.067142	2.789692
H	-0.000924	-1.054285	2.330910
C	-1.367761	-2.403216	3.308248
H	-2.125738	-2.382009	2.502892
H	-1.677435	-1.676661	4.089444
H	-1.383822	-3.416758	3.764257
C	1.020903	-2.000836	3.982840
H	1.038225	-2.952523	4.555363
H	0.702957	-1.209747	4.690617
H	2.060685	-1.777519	3.669053
C	0.124453	-3.639609	-2.153929
H	-0.383979	-2.661829	-2.268301
C	-0.872566	-4.739263	-2.582405
H	-0.416196	-5.750280	-2.507831
H	-1.183675	-4.589897	-3.638048
H	-1.786026	-4.728188	-1.954685
C	1.351765	-3.629419	-3.089503
H	2.072508	-2.835588	-2.810598
H	1.035701	-3.434798	-4.135552
H	1.887064	-4.603461	-3.080034
C	5.737382	0.429694	-1.602702
C	7.208226	0.773560	-1.286775
H	7.737713	-0.039145	-0.753031
H	7.312161	1.703368	-0.693653
H	7.746647	0.942811	-2.245309
C	5.686641	-0.762721	-2.585842
H	4.641530	-1.043985	-2.825457
H	6.239502	-1.652372	-2.228405
H	6.164920	-0.443138	-3.537213
C	5.064361	1.609859	-2.343493
H	5.648310	1.824298	-3.265473
H	5.023934	2.543038	-1.755222
H	4.037930	1.336739	-2.659637
C	5.088894	-1.775408	0.578824
C	4.325132	-2.757347	-0.342771
H	4.674278	-2.745389	-1.389878
H	3.233657	-2.551306	-0.341185
H	4.459079	-3.790117	0.044817
C	4.510087	-1.977675	1.995103
H	4.600613	-3.052792	2.261045
H	3.430842	-1.728027	2.030601
H	5.042839	-1.399615	2.774601
C	6.592028	-2.123104	0.593848
H	7.182276	-1.448101	1.243847
H	7.043740	-2.113582	-0.416998
H	6.715156	-3.154462	0.992013
C	5.037363	1.307748	1.371682
C	6.368746	1.081794	2.117974
H	7.248346	1.130724	1.447484
H	6.397658	0.114398	2.656334
H	6.491789	1.880491	2.882448
C	5.025194	2.733955	0.775900
H	5.081857	3.459646	1.615911
H	4.085349	2.950638	0.228227
H	5.886377	2.936608	0.110953
C	3.854559	1.246674	2.366674
H	3.966793	2.064393	3.111197
H	3.797769	0.295474	2.925203
H	2.887030	1.403622	1.846119
Cu	2.436928	0.029274	-0.693295
C	1.283083	-0.046851	-2.273890
O	-0.008467	-0.031859	-2.497566
O	2.168240	-0.109129	-3.134644

TS2-Cu

E= -4901.3917964

P	5.725309	-0.070660	-0.058541
Al	-0.893344	-0.111891	-0.458888
O	-2.804097	0.049344	-1.047920
N	-1.479268	-1.826831	0.083102
N	-1.158411	1.718511	-0.019386
C	-3.610675	-1.010348	-0.576539
C	-2.862348	-2.034090	0.039068
C	-3.616470	-3.120240	0.537318
H	-3.076788	-3.954025	1.010649
C	-5.032673	-3.122204	0.464125
C	-5.706091	-2.025536	-0.126567
H	-6.802069	-2.014956	-0.165197
C	-4.994719	-0.927809	-0.667260
C	-5.601839	0.315920	-1.361931
C	-4.730156	1.530629	-0.964886
C	-5.179134	2.841939	-0.699418
H	-6.248394	3.072637	-0.806431
C	-4.279941	3.862952	-0.295541
C	-2.917524	3.550710	-0.079403
H	-2.216834	4.311503	0.287711
C	-2.429120	2.235728	-0.292127
C	-3.360327	1.325116	-0.823754
C	-4.825636	5.297192	-0.081280
C	-5.424619	5.817758	-1.414176
H	-4.652995	5.848723	-2.211478
H	-5.826731	6.845735	-1.285523
H	-6.253541	5.174747	-1.774283
C	-5.930121	5.276331	1.007217
H	-6.781543	4.624359	0.724161
H	-6.332470	6.298828	1.172558
H	-5.529614	4.905306	1.973642
C	-3.726579	6.283886	0.367621
H	-3.271432	5.988995	1.335634
H	-4.162388	7.295964	0.501617
H	-2.913285	6.368573	-0.382292
C	-7.073834	0.524268	-0.963745
H	-7.187632	0.673860	0.128905
H	-7.687806	-0.349007	-1.262698
H	-7.500034	1.406048	-1.483137
C	-5.513630	0.115183	-2.904489
H	-5.915314	1.004971	-3.432644
H	-6.100736	-0.774928	-3.212533
H	-4.466243	-0.034375	-3.234697
C	-5.806083	-4.323419	1.063942
C	-5.374034	-5.628554	0.347155
H	-4.287958	-5.822819	0.459140
H	-5.597424	-5.579077	-0.738952
H	-5.915897	-6.502096	0.769122
C	-5.484518	-4.437091	2.577282
H	-5.784442	-3.515212	3.118087
H	-4.402299	-4.596814	2.759201
H	-6.030794	-5.293287	3.028250
C	-7.334876	-4.174347	0.909049
H	-7.844683	-5.055347	1.351750
H	-7.640289	-4.115160	-0.156330

H	-7.720014	-3.273187	1.429889
C	-0.628370	-2.981337	0.181375
C	-0.571116	-3.908322	-0.908882
C	0.233128	-5.057732	-0.775204
H	0.279792	-5.777680	-1.608516
C	0.989951	-5.295010	0.380683
H	1.608305	-6.203315	0.463784
C	0.968832	-4.355698	1.418619
H	1.582660	-4.528451	2.318061
C	0.174553	-3.191720	1.339413
C	0.237319	-2.177325	2.479560
H	-0.455963	-1.349048	2.217126
C	1.660061	-1.588713	2.600445
H	1.706125	-0.804275	3.385669
H	1.994822	-1.139179	1.639307
H	2.394212	-2.375285	2.877155
C	-0.247764	-2.766420	3.820069
H	-1.282226	-3.158354	3.736415
H	-0.238398	-1.993536	4.617741
H	0.401932	-3.602050	4.158184
C	-1.302349	-3.677071	-2.235209
H	-1.903736	-2.751903	-2.132609
C	-0.305010	-3.441638	-3.392288
H	0.358032	-2.571746	-3.202315
H	-0.856569	-3.252561	-4.338608
H	0.338426	-4.332500	-3.558055
C	-2.283342	-4.821209	-2.566812
H	-1.750761	-5.783588	-2.725314
H	-2.840828	-4.596479	-3.500783
H	-3.024426	-4.971811	-1.755647
C	-0.186478	2.616115	0.546770
C	-0.182763	2.860858	1.955243
C	0.755138	3.774630	2.480936
H	0.763048	3.975495	3.564738
C	1.671277	4.435074	1.652319
H	2.390331	5.151560	2.081536
C	1.665981	4.181220	0.273643
H	2.386894	4.704267	-0.374457
C	0.749087	3.279291	-0.306110
C	0.746335	3.074160	-1.822239
H	0.221594	2.117505	-2.018283
C	-0.044693	4.196070	-2.533019
H	-1.089067	4.257022	-2.165242
H	-0.079050	4.016664	-3.628734
H	0.432391	5.186377	-2.366665
C	2.161194	2.941603	-2.415869
H	2.732103	3.893880	-2.360633
H	2.102447	2.652462	-3.485448
H	2.739897	2.149790	-1.893338
C	-1.169407	2.180757	2.908779
H	-1.723883	1.421792	2.317902
C	-2.206010	3.176143	3.475176
H	-1.713534	3.964471	4.084536
H	-2.933443	2.651383	4.130377
H	-2.776711	3.673469	2.666306
C	-0.452403	1.445586	4.059591
H	0.301996	0.729123	3.678277
H	-1.183091	0.880068	4.675563
H	0.070289	2.153410	4.737839
C	6.233632	-1.574468	1.065577
C	7.742557	-1.892434	1.095827
H	8.349367	-1.044522	1.470128
H	8.136495	-2.186636	0.103288
H	7.918942	-2.751705	1.780426
C	5.742373	-1.312040	2.507586
H	4.661814	-1.064919	2.534933
H	6.305725	-0.510532	3.023025
H	5.883709	-2.242509	3.099649
C	5.436246	-2.802913	0.556176
H	5.561265	-3.638234	1.279757
H	5.772634	-3.167996	-0.430312
H	4.351095	-2.576054	0.480615
C	6.138008	1.602809	0.839882
C	5.016358	1.847927	1.880876
H	5.033721	1.134064	2.723366
H	4.011767	1.801532	1.410752
H	5.140584	2.867089	2.307912
C	6.035287	2.759462	-0.180822
H	6.112078	3.720616	0.372816
H	5.059224	2.760157	-0.707418
H	6.846926	2.751286	-0.933374
C	7.520372	1.651651	1.522100
H	8.352367	1.490843	0.808876
H	7.618864	0.910046	2.338927
H	7.665885	2.655900	1.978543
C	6.666789	-0.166856	-1.761994
C	8.141198	0.281016	-1.716879
H	8.748482	-0.322435	-1.013786
H	8.253457	1.347999	-1.441022
H	8.589434	0.160029	-2.727945
C	6.586532	-1.615489	-2.296955
H	6.973700	-1.622768	-3.339106
H	5.539781	-1.981428	-2.334795
H	7.200011	-2.331738	-1.717204
C	5.867586	0.698339	-2.771081
H	6.305191	0.558063	-3.783961
H	5.895794	1.779528	-2.547238
H	4.803319	0.379662	-2.817163
C	1.923429	-0.487247	-1.639978
O	2.023755	-0.813823	-2.823491
O	0.673526	-0.321411	-1.115142
Cu	3.556728	-0.228300	-0.635979

4-Cu

E= -4901.4312272

P	5.703642	-0.052186	-0.531486
Al	-0.723305	-0.008130	0.107073
O	-2.446271	0.022748	-0.943050
N	-1.244327	1.823854	0.388051
N	-1.325936	-1.810426	0.424408
C	-3.178080	1.215697	-0.846239
C	-2.483351	2.209889	-0.123675
C	-3.157008	3.448003	0.000399
H	-2.651681	4.265576	0.535750
C	-4.465291	3.626749	-0.519051
C	-5.114276	2.551399	-1.169829
H	-6.135249	2.677967	-1.549612
C	-4.471185	1.301342	-1.345885
C	-5.058509	0.065996	-2.069654
C	-4.500816	-1.200642	-1.377809
C	-5.170088	-2.435496	-1.228223
H	-6.192506	-2.539429	-1.618121
C	-4.549964	-3.540500	-0.590502
C	-3.251552	-3.398414	-0.047355
H	-2.769084	-4.229469	0.483207
C	-2.552513	-2.165996	-0.137010
C	-3.207100	-1.153619	-0.866400
C	-5.327702	-4.877109	-0.489655
C	-6.647725	-4.650052	0.292250
H	-6.442419	-4.285369	1.320308
H	-7.221628	-5.598470	0.372246
H	-7.301326	-3.903859	-0.203946
C	-5.655746	-5.388958	-1.916639
H	-6.274778	-4.664146	-2.483873
H	-6.217385	-6.346920	-1.868764
H	-4.727054	-5.564590	-2.498802
C	-4.521522	-5.973363	0.239241
H	-3.569860	-6.205063	-0.282103
H	-5.114590	-6.910943	0.281551
H	-4.279045	-5.686169	1.283121
C	-4.565586	0.079919	-3.547837
H	-3.458507	0.065957	-3.602470
H	-4.923245	0.993962	-4.066565
H	-4.947127	-0.809647	-4.091522
C	-6.598503	0.083321	-2.051574
H	-7.008354	-0.793430	-2.592236
H	-6.987846	0.984382	-2.566778
H	-6.996567	0.073100	-1.016831
C	-5.154549	5.003087	-0.339494
C	-5.280447	5.327520	1.171796
H	-4.290249	5.372168	1.669129
H	-5.885641	4.557626	1.694480
H	-5.774345	6.312052	1.320030
C	-4.304201	6.100109	-1.030901
H	-4.201036	5.897291	-2.117442

H	-3.283432	6.163103	-0.602063
H	-4.780782	7.097045	-0.911497
C	-6.570279	5.035890	-0.954564
H	-7.021708	6.039157	-0.805935
H	-7.246499	4.294730	-0.480137
H	-6.552916	4.837894	-2.046475
C	-0.389495	2.863853	0.889188
C	-0.325580	3.135156	2.288154
C	0.496536	4.187455	2.743680
H	0.542792	4.405901	3.823229
C	1.250532	4.962206	1.853103
H	1.879695	5.785951	2.227854
C	1.200047	4.677657	0.481535
H	1.799567	5.282844	-0.218571
C	0.395795	3.635711	-0.025402
C	0.381190	3.378874	-1.534582
H	-0.191850	2.445671	-1.703320
C	1.797071	3.144451	-2.101179
H	2.435716	4.049829	-2.010578
H	1.742822	2.882817	-3.179219
H	2.305290	2.305810	-1.578862
C	-0.332814	4.515224	-2.299026
H	-1.371462	4.653905	-1.937068
H	-0.378795	4.289225	-3.385886
H	0.202473	5.482255	-2.179394
C	-1.123377	2.321163	3.305503
H	-1.594110	1.484594	2.747284
C	-0.207380	1.711089	4.385162
H	0.586016	1.086217	3.927080
H	-0.791833	1.073663	5.080579
H	0.286345	2.496074	4.997333
C	-2.261270	3.150723	3.938090
H	-1.861609	4.016197	4.509863
H	-2.857054	2.530739	4.641679
H	-2.952378	3.544785	3.165460
C	-0.531575	-2.860131	0.999694
C	0.259356	-3.695573	0.150348
C	1.003858	-4.744190	0.731140
H	1.606539	-5.400098	0.081311
C	0.992953	-4.967523	2.113377
H	1.577914	-5.794268	2.548260
C	0.237484	-4.126604	2.942423
H	0.242708	-4.299992	4.029493
C	-0.530580	-3.068406	2.413835
C	-1.361053	-2.185970	3.349168
H	-1.439671	-1.190695	2.859844
C	-2.799749	-2.726452	3.514487
H	-3.321698	-2.814325	2.541464
H	-3.399919	-2.054055	4.164279
H	-2.787173	-3.732604	3.987132
C	-0.709563	-1.979959	4.728806
H	-0.697806	-2.916290	5.326925
H	-1.285550	-1.235619	5.316600
H	0.334264	-1.615582	4.642611
C	0.317077	-3.500261	-1.367363
H	-0.219394	-2.558853	-1.599353
C	-0.395038	-4.646803	-2.118524
H	0.105310	-5.622705	-1.936330
H	-0.385198	-4.463964	-3.214367
H	-1.452852	-4.741001	-1.800308
C	1.763159	-3.330599	-1.878815
H	2.274159	-2.485505	-1.370128
H	1.763271	-3.115416	-2.968536
H	2.369736	-4.248460	-1.720400
C	6.300322	-0.521734	-2.319824
C	7.777789	-0.190496	-2.614394
H	8.474694	-0.708516	-1.926710
H	7.989602	0.895650	-2.566390
H	8.025443	-0.522413	-3.646800
C	6.062086	-2.031620	-2.552342
H	5.013784	-2.324460	-2.338505
H	6.740521	-2.676813	-1.961858
H	6.253227	-2.252855	-3.624899
C	5.374195	0.218555	-3.318421
H	5.601034	-0.137307	-4.347252
H	5.505194	1.314932	-3.312673
H	4.304318	0.001129	-3.114136
C	6.329124	-1.366142	0.758410
C	5.401339	-2.601805	0.632060
H	5.531890	-3.156840	-0.313572
H	4.330810	-2.314970	0.715150
H	5.625889	-3.305039	1.463423
C	6.111024	-0.811929	2.185441
H	6.318331	-1.628703	2.910416
H	5.062571	-0.487741	2.348545
H	6.788342	0.026389	2.437642
C	7.803970	-1.785914	0.594806
H	8.503116	-0.932277	0.691919
H	8.000358	-2.284650	-0.374655
H	8.063437	-2.517127	1.391891
C	6.337632	1.722383	-0.051826
C	7.819790	1.786737	0.369529
H	8.505432	1.444482	-0.430469
H	8.029841	1.194157	1.281403
H	8.084924	2.841691	0.602162
C	6.104743	2.682556	-1.241098
H	6.320273	3.717794	-0.898259
H	5.050959	2.665540	-1.586765
H	6.768879	2.481483	-2.103564
C	5.428272	2.232377	1.095708
H	5.667104	3.299970	1.294240
H	5.562920	1.682816	2.043954
H	4.353964	2.174754	0.817672
Cu	3.460963	-0.046733	-0.420650
C	1.550230	-0.042395	-0.194675
O	0.981171	-0.025457	0.983010
O	0.681523	-0.047479	-1.179456

TS3-Cu

E= -4901.3403463

P	4.617935	-0.037494	-0.290743
Al	-0.241689	-0.009334	-0.217905
O	-2.175442	0.028595	-0.869897
N	-0.815594	-1.813432	0.256198
N	-0.746127	1.816191	0.255328
C	-2.914469	-1.136051	-0.636118
C	-2.144756	-2.146111	-0.031291
C	-2.831545	-3.362218	0.224992
H	-2.269674	-4.192209	0.671913
C	-4.211908	-3.493725	-0.055903
C	-4.926226	-2.394780	-0.597909
H	-6.004185	-2.490761	-0.790332
C	-4.281014	-1.176334	-0.900803
C	-4.933428	0.082803	-1.518363
C	-4.229728	1.319193	-0.911531
C	-4.823738	2.566230	-0.620169
H	-5.896364	2.705752	-0.815718
C	-4.064804	3.638860	-0.086172
C	-2.692213	3.451075	0.201035
H	-2.096947	4.260068	0.643306
C	-2.057678	2.204238	-0.041543
C	-2.866615	1.223744	-0.643806
C	-4.770752	4.991784	0.183566
C	-3.812422	6.048992	0.772431
H	-3.389202	5.729572	1.747065
H	-4.360263	6.999288	0.943382
H	-2.967534	6.271328	0.088359
C	-5.341488	5.547116	-1.147527
H	-4.531708	5.713880	-1.888225
H	-5.856264	6.517306	-0.977298
H	-6.075754	4.852696	-1.604558
C	-5.929781	4.778359	1.191782
H	-6.685177	4.061335	0.810544
H	-6.451264	5.738475	1.395096
H	-5.549205	4.383532	2.156854
C	-6.451554	0.115118	-1.265912
H	-6.687860	0.124525	-0.182657
H	-6.944882	-0.766007	-1.723396
H	-6.908388	1.011843	-1.730906
C	-4.669003	0.070738	-3.054430
H	-5.096713	0.979464	-3.527257
H	-5.133592	-0.823722	-3.519593
H	-3.583112	0.047572	-3.276433
C	-4.973990	-4.812829	0.228347
C	-4.060230	-5.903657	0.826658

H	-3.621973	-5.592437	1.797282
H	-3.226808	-6.168561	0.143697
H	-4.647473	-6.828126	1.008103
C	-6.121406	-4.540591	1.235701
H	-6.846662	-3.796406	0.848070
H	-5.723089	-4.152355	2.196278
H	-6.682383	-5.475879	1.449305
C	-5.569809	-5.356702	-1.096487
H	-6.124612	-6.302686	-0.916083
H	-4.769008	-5.564574	-1.836549
H	-6.275034	-4.636540	-1.559404
C	0.020193	-2.897063	0.690568
C	0.501281	-3.843287	-0.268184
C	1.254811	-4.947655	0.181341
H	1.621437	-5.683950	-0.552730
C	1.546173	-5.125866	1.539704
H	2.129993	-5.998950	1.873538
C	1.091333	-4.183422	2.472544
H	1.325284	-4.328306	3.538487
C	0.323893	-3.067000	2.077441
C	-0.217308	-2.101088	3.133375
H	-0.334740	-1.114189	2.633865
C	0.729513	-1.912025	4.331934
H	0.340228	-1.124267	5.008486
H	1.748744	-1.613253	4.011206
H	0.822236	-2.836602	4.940612
C	-1.620200	-2.531127	3.619485
H	-2.339379	-2.608056	2.780437
H	-2.024571	-1.799499	4.351187
H	-1.573258	-3.522380	4.120285
C	0.242846	-3.684102	-1.767881
H	-0.405354	-2.795063	-1.898887
C	1.559171	-3.400701	-2.520912
H	2.068379	-2.513562	-2.089680
H	1.369311	-3.189839	-3.593963
H	2.259867	-4.261308	-2.460092
C	-0.499086	-4.892415	-2.374628
H	0.104504	-5.823322	-2.310257
H	-0.718094	-4.714015	-3.448608
H	-1.463201	-5.073328	-1.857485
C	0.133209	2.863300	0.693601
C	0.406946	3.045390	2.085114
C	1.220507	4.128201	2.481047
H	1.430274	4.283168	3.550677
C	1.759489	5.020053	1.543416
H	2.383199	5.865106	1.877212
C	1.499180	4.828315	0.180529
H	1.927205	5.527408	-0.556787
C	0.690592	3.762977	-0.268454
C	0.443670	3.600103	-1.769257
H	-0.245814	2.742486	-1.898094
C	-0.232929	4.836932	-2.395195
H	-1.193591	5.067716	-1.891602
H	-0.447222	4.659773	-3.470363
H	0.413188	5.738825	-2.330276
C	1.751554	3.244896	-2.504410
H	2.487132	4.076977	-2.458735
H	1.562634	3.016221	-3.573995
H	2.219008	2.346500	-2.048181
C	-0.204104	2.124938	3.143362
H	-0.382725	1.145668	2.647595
C	-1.577707	2.649178	3.620192
H	-1.468656	3.638511	4.115214
H	-2.032224	1.950289	4.354445
H	-2.286562	2.766988	2.777076
C	0.723692	1.881156	4.347259
H	1.720257	1.508492	4.032619
H	0.277335	1.129291	5.029837
H	0.879161	2.802392	4.948135
C	5.032375	-1.529397	0.887685
C	6.514847	-1.954356	0.919956
H	7.188664	-1.141206	1.252851
H	6.872442	-2.319510	-0.062537
H	6.632036	-2.795796	1.638392
C	4.568994	-1.184202	2.321411
H	3.507420	-0.861616	2.339610
H	5.187985	-0.407661	2.810185
H	4.643486	-2.103264	2.942213
C	4.151669	-2.716576	0.430086
H	3.078110	-2.431598	0.389707
H	4.243384	-3.542563	1.167530
H	4.433950	-3.121243	-0.557409
C	5.204574	1.622972	0.541044
C	4.117072	2.009909	1.572502
H	4.343842	3.020028	1.976423
H	4.056490	1.315947	2.429518
H	3.112468	2.061409	1.101041
C	5.221836	2.756957	-0.508768
H	5.400441	3.716704	0.022925
H	4.249999	2.852981	-1.031614
H	6.025723	2.646328	-1.261529
C	6.586055	1.556539	1.225306
H	7.400339	1.295643	0.521397
H	6.612596	0.837631	2.067015
H	6.824357	2.557313	1.649069
C	5.543478	-0.274413	-1.989769
C	7.051741	0.050655	-1.949775
H	7.605188	-0.571093	-1.219270
H	7.254569	1.115154	-1.721712
H	7.488892	-0.151787	-2.952828
C	5.359046	-1.727218	-2.483715
H	5.741843	-1.789066	-3.525698
H	4.292322	-2.026661	-2.511357
H	5.923604	-2.468103	-1.885346
C	4.831687	0.627754	-3.028502
H	5.318096	0.476569	-4.017529
H	4.895753	1.704956	-2.794894
H	3.761411	0.359681	-3.150150
C	0.576623	-0.055123	-3.253521
O	1.507611	-0.098505	-4.008406
O	0.458642	-0.021896	-1.961856
Cu	2.305010	-0.017008	-0.491669

TS3′-Cu

E= -4901.3851824

P	-5.154769	-0.121249	-0.418006
Al	0.476306	-0.036085	-0.126286
O	2.240893	0.094719	-1.044081
N	1.163114	-1.767063	0.364027
N	0.913627	1.762138	0.402312
C	3.055590	-1.046188	-0.886568
C	2.433790	-2.064394	-0.135281
C	3.203777	-3.242861	0.026458
H	2.756942	-4.088268	0.570181
C	4.535010	-3.324178	-0.456292
C	5.116608	-2.207112	-1.101762
H	6.160759	-2.247969	-1.434105
C	4.374105	-1.023308	-1.323569
C	4.886527	0.279775	-1.981579
C	4.241475	1.456143	-1.209750
C	4.847154	2.686665	-0.879226
H	5.887939	2.868882	-1.181968
C	4.141156	3.687889	-0.162089
C	2.816632	3.440713	0.266745
H	2.263886	4.191978	0.845546
C	2.175016	2.204519	-0.010435
C	2.922092	1.304375	-0.791458
C	4.856745	5.026320	0.150313
C	6.128725	4.747056	0.992779
H	5.869248	4.254791	1.953201
H	6.657810	5.696256	1.225286
H	6.843639	4.087350	0.459983
C	5.260095	5.715141	-1.179585
H	5.944279	5.083402	-1.782139
H	5.779195	6.677077	-0.978901
H	4.367012	5.931093	-1.802395
C	3.960608	6.001273	0.943587
H	3.037841	6.264730	0.386591
H	4.512130	6.944812	1.138182
H	3.660478	5.583435	1.926643
C	4.395577	0.323337	-3.459868
H	3.290733	0.263909	-3.523226
H	4.817850	-0.529998	-4.030425
H	4.717796	1.267989	-3.945583
C	6.422805	0.361123	-1.956783

H	6.773406	1.294376	-2.441911
H	6.869136	-0.480494	-2.523777
H	6.820409	0.334604	-0.922122
C	5.329117	-4.635850	-0.231047
C	5.455600	-4.910994	1.289949
H	4.464239	-5.018196	1.775058
H	5.992364	-4.084154	1.800273
H	6.021188	-5.850455	1.470338
C	4.581433	-5.814629	-0.905913
H	4.475061	-5.646144	-1.997902
H	3.564641	-5.954301	-0.485917
H	5.138125	-6.764910	-0.757772
C	6.752568	-4.569072	-0.825399
H	7.278176	-5.530950	-0.650140
H	7.362249	-3.769294	-0.356043
H	6.736664	-4.393444	-1.921134
C	0.425437	-2.850360	0.955164
C	0.401200	-3.010652	2.375932
C	-0.306037	-4.101587	2.924104
H	-0.329816	-4.236857	4.016368
C	-0.971473	-5.025636	2.106995
H	-1.509991	-5.875791	2.556214
C	-0.946230	-4.859650	0.716781
H	-1.472124	-5.586016	0.075525
C	-0.260431	-3.782552	0.116260
C	-0.272825	-3.661275	-1.408571
H	0.240040	-2.714668	-1.671684
C	-1.709518	-3.566414	-1.957507
H	-2.296042	-4.487335	-1.750736
H	-1.697956	-3.416241	-3.057447
H	-2.241328	-2.703110	-1.506751
C	0.500168	-4.814778	-2.084181
H	1.551049	-4.858677	-1.734231
H	0.515594	-4.679583	-3.186508
H	0.028273	-5.799094	-1.874312
C	1.155827	-2.052683	3.301236
H	1.205244	-1.073841	2.775777
C	0.452579	-1.830219	4.652696
H	-0.606092	-1.524128	4.526264
H	0.974245	-1.038505	5.229361
H	0.468574	-2.743891	5.284354
C	2.612960	-2.512920	3.532840
H	2.634483	-3.502049	4.039554
H	3.155144	-1.789133	4.178104
H	3.172163	-2.605093	2.581540
C	-0.015009	2.767048	0.842116
C	-0.606387	3.651709	-0.114532
C	-1.476512	4.663968	0.341799
H	-1.926203	5.355066	-0.390008
C	-1.785391	4.804648	1.700282
H	-2.464600	5.604272	2.037504
C	-1.226098	3.916180	2.628818
H	-1.476881	4.027681	3.694448
C	-0.337798	2.895434	2.228446
C	0.296331	1.981374	3.279271
H	0.449984	0.991221	2.795587
C	1.692543	2.493226	3.700243
H	2.375026	2.583354	2.832369
H	2.161625	1.802395	4.433084
H	1.613510	3.493384	4.178481
C	-0.591175	1.765504	4.518227
H	-0.696720	2.693328	5.119878
H	-0.141743	1.002781	5.184866
H	-1.609955	1.421933	4.243477
C	-0.345399	3.536409	-1.618216
H	0.279262	2.635416	-1.781235
C	0.431892	4.751176	-2.169956
H	-0.150208	5.690854	-2.053338
H	0.641916	4.620584	-3.252728
H	1.401668	4.881867	-1.649135
C	-1.656355	3.325615	-2.402178
H	-2.210518	2.448309	-2.008299
H	-1.445516	3.135759	-3.475397
H	-2.322670	4.212713	-2.341997
C	-6.352718	-1.294453	-1.410880
C	-7.606582	-1.750653	-0.637351
H	-8.239073	-0.901960	-0.311230
H	-7.358092	-2.358234	0.254732
H	-8.229871	-2.390576	-1.300545
C	-6.787851	-0.585708	-2.713793
H	-5.914679	-0.215473	-3.289404
H	-7.486150	0.255760	-2.541251
H	-7.318889	-1.324044	-3.353409
C	-5.520384	-2.523859	-1.851450
H	-6.125802	-3.136690	-2.554851
H	-5.222307	-3.178044	-1.013194
H	-4.597617	-2.205010	-2.381145
C	-5.893230	1.678420	-0.364942
C	-5.621583	2.336131	-1.741123
H	-6.213462	1.898037	-2.563986
H	-4.549024	2.263202	-2.015492
H	-5.884770	3.414891	-1.679337
C	-5.102275	2.506571	0.673336
H	-5.408739	3.570945	0.579072
H	-4.008110	2.464218	0.497037
H	-5.305134	2.201746	1.717747
C	-7.398684	1.751312	-0.037062
H	-7.644884	1.297100	0.942271
H	-8.026814	1.266708	-0.810303
H	-7.707914	2.819079	0.007188
C	-4.926427	-0.795637	1.396609
C	-6.107087	-0.529257	2.352231
H	-7.055913	-0.979910	2.000169
H	-6.279938	0.551556	2.523348
H	-5.877043	-0.978844	3.343550
C	-4.656175	-2.316508	1.332056
H	-4.355019	-2.660647	2.344977
H	-3.818474	-2.561296	0.647790
H	-5.545384	-2.906974	1.038181
C	-3.630748	-0.152221	1.953583
H	-3.376258	-0.648427	2.915735
H	-3.728744	0.929138	2.155420
H	-2.775934	-0.293561	1.258046
Cu	-3.169951	-0.095931	-1.412722
C	-1.597793	-0.151353	-2.509975
O	-1.132297	-0.134960	-0.750025
O	-0.693025	-0.179004	-3.261237

IM2-Cu

E= -4901.408704

P	-4.841085	-0.056828	-0.323983
Al	0.394570	-0.041768	-0.025616
O	2.173985	0.047723	-1.017340
N	1.086499	-1.786057	0.420440
N	0.923822	1.759437	0.437834
C	2.997150	-1.077943	-0.805944
C	2.376545	-2.075571	-0.026556
C	3.158471	-3.234496	0.202884
H	2.711628	-4.059168	0.777772
C	4.497240	-3.322218	-0.255349
C	5.067012	-2.237413	-0.963265
H	6.112124	-2.285966	-1.291685
C	4.314031	-1.074680	-1.251647
C	4.812258	0.181492	-2.004345
C	4.172383	1.401090	-1.301437
C	4.786151	2.646101	-1.048858
H	5.817816	2.812848	-1.389163
C	4.101292	3.678348	-0.356700
C	2.796029	3.445028	0.133109
H	2.266536	4.215301	0.708606
C	2.150968	2.193994	-0.062945
C	2.862588	1.266649	-0.846191
C	4.821455	5.031806	-0.131829
C	6.126335	4.795685	0.672668
H	5.906495	4.344802	1.662819
H	6.657862	5.756634	0.843147
H	6.824751	4.116634	0.142361
C	5.170006	5.661090	-1.505999
H	5.832113	5.003663	-2.105768
H	5.693107	6.632133	-1.369555
H	4.252433	5.847040	-2.102389
C	3.951802	6.038493	0.651083
H	3.006720	6.273241	0.119202
H	4.505281	6.991925	0.781382
H	3.691837	5.664285	1.662681

C	4.302412	0.121449	-3.475226
H	3.196642	0.059780	-3.517947
H	4.716268	-0.769920	-3.991321
H	4.617057	1.029926	-4.030107
C	6.348387	0.270060	-2.008337
H	6.687827	1.168933	-2.561534
H	6.790430	-0.607214	-2.522272
H	6.760765	0.316584	-0.980272
C	5.314463	-4.597826	0.071158
C	5.442561	-4.743621	1.610213
H	4.451472	-4.828508	2.100455
H	5.961001	-3.866274	2.050284
H	6.026010	-5.653407	1.869159
C	4.592709	-5.843370	-0.503929
H	4.486868	-5.769213	-1.606405
H	3.577977	-5.969578	-0.074760
H	5.168167	-6.766303	-0.276243
C	6.737410	-4.552007	-0.526363
H	7.279683	-5.488118	-0.277621
H	7.332525	-3.708092	-0.119977
H	6.719676	-4.461150	-1.632397
C	0.273444	-2.896888	0.828080
C	-0.058698	-3.080732	2.205277
C	-0.865526	-4.179406	2.569235
H	-1.127913	-4.332188	3.626914
C	-1.333112	-5.091247	1.612581
H	-1.956622	-5.946296	1.920088
C	-0.999613	-4.907257	0.264580
H	-1.370741	-5.623220	-0.487264
C	-0.202482	-3.821706	-0.153843
C	0.101492	-3.665173	-1.646803
H	0.797640	-2.811321	-1.761117
C	-1.172014	-3.313538	-2.441866
H	-1.928707	-4.125113	-2.383938
H	-0.934597	-3.150790	-3.515191
H	-1.626202	-2.382774	-2.042648
C	0.801492	-4.906498	-2.237309
H	1.737295	-5.140680	-1.691149
H	1.064362	-4.734434	-3.302688
H	0.149548	-5.805577	-2.199572
C	0.486228	-2.135499	3.278400
H	0.519399	-1.119476	2.825651
C	-0.392835	-2.055954	4.538532
H	-1.447644	-1.813384	4.293982
H	-0.013211	-1.270375	5.222618
H	-0.386675	-3.008550	5.110078
C	1.938658	-2.499949	3.660921
H	1.981090	-3.517019	4.107001
H	2.341172	-1.783946	4.409048
H	2.610554	-2.484883	2.780489
C	0.050905	2.761550	0.980162
C	-0.641249	3.651390	0.100854
C	-1.484045	4.637528	0.654915
H	-2.016965	5.330360	-0.017335
C	-1.656752	4.755077	2.039983
H	-2.318688	5.532584	2.454367
C	-0.976687	3.878256	2.896685
H	-1.112979	3.979151	3.984113
C	-0.114197	2.880989	2.394937
C	0.665786	1.983876	3.359173
H	0.791022	1.001556	2.852203
C	2.085375	2.537680	3.619057
H	2.660441	2.654157	2.679805
H	2.656303	1.857294	4.286370
H	2.031534	3.532156	4.112642
C	-0.056893	1.733687	4.694404
H	-0.125442	2.656386	5.309304
H	0.504360	0.991715	5.298954
H	-1.086002	1.347073	4.545471
C	-0.500459	3.573887	-1.421484
H	0.173294	2.724124	-1.650151
C	0.140013	4.849127	-2.010842
H	-0.497368	5.743055	-1.838557
H	0.279525	4.745702	-3.107998
H	1.133671	5.045366	-1.560261
C	-1.850945	3.276150	-2.101299
H	-2.280478	2.329671	-1.705026
H	-1.724750	3.162061	-3.199074
H	-2.590031	4.088298	-1.932511
C	-5.837096	-1.627930	-0.925855
C	-6.957810	-2.098990	0.024430
H	-7.728454	-1.323070	0.197396
H	-6.568527	-2.425898	1.007963
H	-7.469650	-2.977281	-0.428329
C	-6.446588	-1.359767	-2.321509
H	-5.686097	-1.018035	-3.052069
H	-7.275883	-0.626854	-2.305696
H	-6.864699	-2.314755	-2.708556
C	-4.807857	-2.768357	-1.118708
H	-5.319328	-3.643194	-1.577496
H	-4.341379	-3.109738	-0.177978
H	-3.990890	-2.460565	-1.803191
C	-5.922261	1.533778	-0.652152
C	-5.781535	1.898927	-2.149910
H	-6.186994	1.132420	-2.834292
H	-4.720339	2.073123	-2.418432
H	-6.336320	2.843517	-2.342841
C	-5.335350	2.724803	0.139599
H	-5.846461	3.652981	-0.198335
H	-4.250112	2.859396	-0.040873
H	-5.500729	2.641174	1.230435
C	-7.414319	1.384926	-0.285648
H	-7.564615	1.121900	0.779280
H	-7.935888	0.629300	-0.904624
H	-7.928119	2.356198	-0.461445
C	-4.490048	-0.209018	1.582569
C	-5.704190	0.016549	2.506543
H	-6.535686	-0.689467	2.316942
H	-6.102872	1.048116	2.438017
H	-5.380230	-0.131268	3.560657
C	-3.887486	-1.608947	1.840729
H	-3.518909	-1.639594	2.888905
H	-3.014697	-1.801472	1.183485
H	-4.620124	-2.432265	1.734695
C	-3.368804	0.805133	1.920590
H	-3.025185	0.604353	2.959339
H	-3.696197	1.859032	1.890136
H	-2.501194	0.674762	1.237873
Cu	-2.786259	-0.054288	-1.490515
C	-2.902299	-0.054850	-3.326064
O	-1.252861	-0.119899	-0.413857
O	-3.108992	-0.035411	-4.464440

8-Cu

E= -4788.0278161

P	5.014544	-0.029786	-0.449173
Al	-0.445338	-0.007782	-0.242204
O	-2.297381	0.006904	-1.029017
N	-0.968394	1.771183	0.278481
N	-1.000197	-1.772415	0.292208
C	-3.017669	1.191985	-0.778592
C	-2.258908	2.158138	-0.085027
C	-2.924697	3.385590	0.153330
H	-2.368012	4.193776	0.650551
C	-4.286812	3.563596	-0.199575
C	-5.000992	2.499266	-0.799583
H	-6.065117	2.618208	-1.036208
C	-4.366920	1.270218	-1.101005
C	-5.024461	0.028521	-1.749814
C	-4.392117	-1.219263	-1.087892
C	-5.044667	-2.429914	-0.770758
H	-6.113586	-2.538250	-1.002666
C	-4.348186	-3.505502	-0.159953
C	-2.986722	-3.351149	0.189357
H	-2.439203	-4.159552	0.691130
C	-2.299196	-2.134781	-0.065807
C	-3.038632	-1.163374	-0.766583
C	-5.113631	-4.821773	0.127798
C	-6.311955	-4.528588	1.067795
H	-5.965050	-4.106473	2.033998
H	-6.875331	-5.462025	1.283127
H	-7.023115	-3.804052	0.621200
C	-5.638338	-5.411543	-1.207440
H	-6.324638	-4.712116	-1.727133
H	-6.194551	-6.356363	-1.025272

H	-4.799875	-5.635157	-1.899628
C	-4.222464	-5.884985	0.804593
H	-3.353473	-6.162054	0.172725
H	-4.810612	-6.809109	0.984830
H	-3.836584	-5.540093	1.785993
C	-4.680271	0.016683	-3.269324
H	-3.584499	0.004368	-3.435315
H	-5.092412	0.920085	-3.765521
H	-5.111297	-0.883089	-3.755986
C	-6.554035	0.044445	-1.579836
H	-7.011503	-0.843135	-2.061609
H	-6.994327	0.935939	-2.070211
H	-6.849059	0.052514	-0.511151
C	-4.964467	4.921682	0.113424
C	-4.930488	5.175997	1.643078
H	-3.893285	5.206974	2.034148
H	-5.472554	4.376919	2.190675
H	-5.411457	6.147496	1.888023
C	-4.202715	6.059722	-0.613656
H	-4.212074	5.906020	-1.712987
H	-3.143472	6.120640	-0.290941
H	-4.674683	7.042987	-0.400564
C	-6.437845	4.962404	-0.346090
H	-6.877551	5.954174	-0.110869
H	-7.055288	4.196958	0.168191
H	-6.535170	4.806279	-1.440584
C	-0.055124	2.810064	0.661482
C	0.275470	3.004787	2.037820
C	1.156146	4.051828	2.383370
H	1.412510	4.216647	3.441176
C	1.704262	4.896024	1.407515
H	2.381353	5.714801	1.700512
C	1.387273	4.689066	0.058657
H	1.828067	5.347027	-0.708489
C	0.515930	3.654489	-0.341538
C	0.228583	3.465645	-1.832916
H	-0.491572	2.628995	-1.928046
C	1.501756	3.047567	-2.597526
H	2.283048	3.837438	-2.554410
H	1.271965	2.863051	-3.668826
H	1.907758	2.108458	-2.167671
C	-0.424811	4.713392	-2.462992
H	-1.362149	4.989122	-1.938417
H	-0.675212	4.524772	-3.528621
H	0.253982	5.593028	-2.433896
C	-0.341660	2.128794	3.130709
H	-0.539227	1.135857	2.669938
C	0.587070	1.911451	4.338773
H	1.582195	1.527209	4.033464
H	0.138414	1.179397	5.041222
H	0.746834	2.847834	4.914818
C	-1.706713	2.684745	3.595262
H	-1.585140	3.692142	4.049039
H	-2.163443	2.020429	4.359734
H	-2.420538	2.774012	2.752906
C	-0.102404	-2.826535	0.669645
C	0.428551	-3.696956	-0.333157
C	1.288550	-4.742329	0.062847
H	1.699525	-5.419514	-0.704047
C	1.633661	-4.935043	1.407012
H	2.302392	-5.761932	1.696320
C	1.123975	-4.066534	2.382216
H	1.400350	-4.221330	3.436549
C	0.254597	-3.008875	2.040824
C	-0.327615	-2.110343	3.134198
H	-0.523182	-1.120926	2.665046
C	-1.689842	-2.644350	3.632108
H	-2.419406	-2.737097	2.803471
H	-2.125340	-1.964705	4.395536
H	-1.570853	-3.647048	4.096816
C	0.629001	-1.888932	4.319407
H	0.790091	-2.819628	4.904210
H	0.205665	-1.141566	5.021228
H	1.622003	-1.521512	3.987079
C	0.113174	-3.523072	-1.820914
H	-0.612953	-2.691214	-1.911918
C	-0.547241	-4.778934	-2.427105
H	0.136513	-5.654931	-2.409125
H	-0.824887	-4.598909	-3.487404
H	-1.469913	-5.055997	-1.877044
C	1.371191	-3.105522	-2.611724
H	1.775071	-2.156602	-2.201546
H	1.122620	-2.940330	-3.682114
H	2.159846	-3.887894	-2.567149
C	5.859483	-0.294113	-2.185223
C	7.356600	0.073225	-2.243000
H	7.966300	-0.507930	-1.523827
H	7.539075	1.150179	-2.058727
H	7.746072	-0.150035	-3.260985
C	5.681821	-1.769989	-2.609558
H	4.621259	-2.089974	-2.552909
H	6.298125	-2.471738	-2.015362
H	6.002844	-1.870563	-3.669365
C	5.065628	0.546389	-3.216769
H	5.440771	0.308630	-4.236544
H	5.169849	1.636072	-3.071553
H	3.983190	0.300421	-3.185552
C	5.524840	-1.473689	0.754881
C	4.666538	-2.707471	0.380547
H	4.944728	-3.158927	-0.588028
H	3.586182	-2.454256	0.340586
H	4.799389	-3.488036	1.160767
C	5.113782	-1.087364	2.194159
H	5.249047	-1.976769	2.847184
H	4.044192	-0.798011	2.249235
H	5.727883	-0.271537	2.621611
C	7.022021	-1.844498	0.736281
H	7.678395	-0.994628	1.007789
H	7.351101	-2.234818	-0.246884
H	7.203837	-2.652053	1.479597
C	5.569473	1.681316	0.298369
C	6.989157	1.699835	0.900698
H	7.771053	1.434893	0.162132
H	7.091590	1.021217	1.770343
H	7.214347	2.726509	1.265270
C	5.465547	2.764647	-0.799301
H	5.608145	3.758929	-0.322945
H	4.463447	2.769269	-1.274387
H	6.237699	2.666942	-1.586696
C	4.526043	2.067723	1.376093
H	4.742617	3.098622	1.731377
H	4.536998	1.404605	2.259164
H	3.497611	2.069232	0.957406
Cu	2.851244	-0.031861	-0.698802
O	1.073600	-0.026842	-0.995715

TS4-Cu

E= -4976.7166656

P	5.011298	-0.040476	-0.119296
Al	-0.498663	-0.010011	-0.390313
O	-2.435276	0.020424	-0.900869
N	-0.950517	1.788223	0.156858
N	-1.007365	-1.788712	0.168996
C	-3.115156	1.213838	-0.632976
C	-2.270387	2.192085	-0.069888
C	-2.888816	3.438427	0.191620
H	-2.273863	4.254988	0.597649
C	-4.278208	3.628915	-0.029379
C	-5.066521	2.557924	-0.512177
H	-6.146085	2.691820	-0.651101
C	-4.487053	1.304671	-0.826987
C	-5.223476	0.063247	-1.386535
C	-4.529432	-1.196697	-0.814414
C	-5.145007	-2.423603	-0.483084
H	-6.231009	-2.530041	-0.615750
C	-4.389925	-3.519070	0.010203
C	-2.998349	-3.374763	0.224631
H	-2.404692	-4.201951	0.634422
C	-2.340929	-2.147637	-0.052899
C	-3.152281	-1.149358	-0.622270
C	-5.121489	-4.850187	0.318284
C	-6.214693	-4.603512	1.390424
H	-5.768146	-4.223513	2.332928
H	-6.753721	-5.547435	1.621952
H	-6.967518	-3.862069	1.053264

C	-5.783012	-5.384445	-0.978970
H	-6.520659	-4.667122	-1.393158
H	-6.316334	-6.339195	-0.780816
H	-5.021672	-5.573431	-1.764349
C	-4.164717	-5.938108	0.851168
H	-3.366645	-6.183762	0.120503
H	-4.731226	-6.871660	1.051177
H	-3.678196	-5.634918	1.801124
C	-5.062179	0.052586	-2.936704
H	-3.993502	0.032264	-3.229988
H	-5.522958	0.960565	-3.379002
H	-5.554992	-0.842651	-3.370363
C	-6.721575	0.090025	-1.032369
H	-7.241277	-0.792831	-1.456093
H	-7.211879	0.985364	-1.464743
H	-6.884335	0.098064	0.064377
C	-4.895917	5.015305	0.284234
C	-4.683969	5.353389	1.782796
H	-3.608798	5.385393	2.052250
H	-5.171899	4.598008	2.433628
H	-5.119798	6.346900	2.023810
C	-4.205375	6.095309	-0.588435
H	-4.343229	5.881418	-1.668945
H	-3.115176	6.149731	-0.392482
H	-4.634323	7.099047	-0.379584
C	-6.411524	5.062062	-0.006472
H	-6.807969	6.072345	0.226866
H	-6.976007	4.333911	0.612287
H	-6.635904	4.855440	-1.073543
C	-0.040484	2.813417	0.590507
C	0.207104	3.015359	1.984458
C	1.044109	4.080953	2.379823
H	1.230672	4.251146	3.451693
C	1.634868	4.935379	1.439491
H	2.274923	5.768824	1.771683
C	1.405968	4.718687	0.074999
H	1.876767	5.385680	-0.665929
C	0.576834	3.669119	-0.373687
C	0.371722	3.486759	-1.877228
H	-0.260400	2.586918	-2.013426
C	1.714448	3.224438	-2.589768
H	2.393718	4.101478	-2.521467
H	1.558227	3.010289	-3.667945
H	2.224309	2.348874	-2.138151
C	-0.377152	4.678570	-2.510406
H	-1.362423	4.839371	-2.027722
H	-0.554435	4.497903	-3.591983
H	0.204940	5.621299	-2.420981
C	-0.440127	2.133744	3.054981
H	-0.714852	1.177510	2.558898
C	0.515273	1.809163	4.218657
H	1.464514	1.359821	3.861197
H	0.039570	1.093241	4.919543
H	0.770065	2.712934	4.811883
C	-1.746899	2.757236	3.595119
H	-1.542087	3.730711	4.091320
H	-2.220909	2.088125	4.344674
H	-2.481270	2.935192	2.785342
C	-0.126547	-2.844999	0.588536
C	0.431717	-3.731386	-0.383889
C	1.230678	-4.809083	0.052059
H	1.655764	-5.499457	-0.694977
C	1.490067	-5.021759	1.411609
H	2.107223	-5.876014	1.734068
C	0.960100	-4.136197	2.359349
H	1.170920	-4.303939	3.426950
C	0.151674	-3.044170	1.976975
C	-0.437923	-2.132236	3.055455
H	-0.659640	-1.158440	2.566564
C	-1.777174	-2.682385	3.596728
H	-2.523391	-2.812578	2.788530
H	-2.209036	-1.992204	4.352659
H	-1.626375	-3.669501	4.085230
C	0.537710	-1.870347	4.217958
H	0.734124	-2.788836	4.810783
H	0.111197	-1.124932	4.919287
H	1.514172	-1.483798	3.859709
C	0.198329	-3.550201	-1.883555
H	-0.403518	-2.628711	-2.011635
C	-0.607408	-4.717606	-2.492016
H	-0.057129	-5.680041	-2.411540
H	-0.804960	-4.535804	-3.569878
H	-1.585433	-4.840405	-1.983780
C	1.533114	-3.340431	-2.628650
H	2.086720	-2.483470	-2.193619
H	1.358111	-3.126061	-3.704001
H	2.180735	-4.241812	-2.571874
C	5.991854	-0.248289	-1.790888
C	7.489271	0.113202	-1.707596
H	8.038200	-0.501048	-0.967186
H	7.656362	1.180407	-1.462940
H	7.960118	-0.067529	-2.699172
C	5.847963	-1.706377	-2.283582
H	4.785825	-2.021424	-2.335495
H	6.407741	-2.434417	-1.665421
H	6.260081	-1.767872	-3.314330
C	5.289576	0.634058	-2.853566
H	5.771494	0.450394	-3.839162
H	5.365336	1.716313	-2.648114
H	4.214866	0.376398	-2.954501
C	5.414192	-1.527925	1.071790
C	4.585189	-2.742885	0.586080
H	4.921944	-3.146473	-0.384919
H	3.507486	-2.491253	0.495903
H	4.674801	-3.560112	1.334307
C	4.890272	-1.192005	2.486738
H	4.968189	-2.106112	3.114394
H	3.820685	-0.897813	2.466610
H	5.470771	-0.397328	2.993491
C	6.906955	-1.906338	1.157721
H	7.541163	-1.071011	1.513333
H	7.311221	-2.259010	0.188681
H	7.027087	-2.743105	1.880836
C	5.505019	1.640794	0.731825
C	6.869237	1.626289	1.451600
H	7.709150	1.383455	0.771737
H	6.895649	0.915369	2.300534
H	7.066835	2.637183	1.871753
C	5.501024	2.765642	-0.328250
H	5.615574	3.738735	0.196851
H	4.541225	2.804681	-0.882303
H	6.332822	2.686773	-1.054386
C	4.377573	1.995636	1.733081
H	4.574303	3.007296	2.149755
H	4.306312	1.295892	2.584543
H	3.388136	2.031729	1.230558
Cu	2.872535	-0.035547	-0.545212
O	1.113877	-0.040853	-1.007732
C	0.655238	-0.037050	-3.148549
O	-0.533190	-0.018578	-3.012777
O	1.716112	-0.054696	-3.667544

IM3-Cu

E= -4976.7529083

P	5.226294	-0.002156	0.124357
Al	-0.760076	-0.009017	-0.638874
O	-2.753501	-0.000426	-0.888619
N	-1.130400	1.793859	-0.085695
N	-1.142558	-1.812048	-0.105478
C	-3.388240	1.196883	-0.525464
C	-2.469203	2.190768	-0.125715
C	-3.044950	3.437995	0.217629
H	-2.375760	4.261691	0.507416
C	-4.452575	3.617277	0.236916
C	-5.301507	2.531643	-0.081281
H	-6.389916	2.654615	-0.029291
C	-4.774011	1.275896	-0.468749
C	-5.591236	0.016452	-0.843859
C	-4.789434	-1.222119	-0.376014
C	-5.323179	-2.439749	0.100576
H	-6.413945	-2.548315	0.182742
C	-4.483922	-3.522373	0.471022
C	-3.078513	-3.377361	0.397488
H	-2.410157	-4.192445	0.704411
C	-2.493377	-2.164019	-0.046177

C	-3.401030	-1.168888	-0.455477
C	-5.137462	-4.838547	0.962922
C	-5.992529	-4.550945	2.224487
H	-5.365739	-4.142876	3.044727
H	-6.473126	-5.483679	2.590789
H	-6.798264	-3.816365	2.021221
C	-6.046345	-5.410690	-0.156325
H	-6.853158	-4.703113	-0.436692
H	-6.527903	-6.355390	0.176590
H	-5.458667	-5.629562	-1.072225
C	-4.091996	-5.914404	1.326769
H	-3.456282	-6.186236	0.459010
H	-4.605644	-6.838408	1.665617
H	-3.425263	-5.584883	2.150272
C	-5.729330	-0.042213	-2.394849
H	-4.736592	-0.066161	-2.887230
H	-6.275609	0.849272	-2.767832
H	-6.285811	-0.953026	-2.699823
C	-6.995429	0.048364	-0.212656
H	-7.576770	-0.849908	-0.502826
H	-7.568370	0.926931	-0.571375
H	-6.947317	0.090908	0.894229
C	-5.021014	5.001825	0.638982
C	-4.576291	5.344183	2.084818
H	-3.472066	5.380420	2.180321
H	-4.951622	4.587778	2.805387
H	-4.973011	6.336332	2.390410
C	-4.484784	6.084041	-0.333160
H	-4.787586	5.865837	-1.378532
H	-3.378027	6.149519	-0.310133
H	-4.884755	7.084641	-0.061490
C	-6.563774	5.038936	0.591441
H	-6.924198	6.049696	0.875584
H	-7.019457	4.314890	1.298348
H	-6.952323	4.820582	-0.424961
C	-0.142870	2.807698	0.161574
C	0.388490	2.983398	1.478641
C	1.352256	3.992064	1.690046
H	1.765511	4.144113	2.698825
C	1.788172	4.817979	0.643179
H	2.533512	5.607127	0.834388
C	1.267554	4.634969	-0.643522
H	1.613709	5.282596	-1.465526
C	0.305641	3.637928	-0.913261
C	-0.233877	3.499009	-2.339945
H	-0.865314	2.588831	-2.367085
C	0.889450	3.303856	-3.380162
H	1.545247	4.198426	-3.452172
H	0.451565	3.135889	-4.386466
H	1.526547	2.427130	-3.148375
C	-1.124862	4.700789	-2.725350
H	-1.974903	4.820585	-2.024438
H	-1.543651	4.562804	-3.744838
H	-0.545475	5.649595	-2.727130
C	-0.089863	2.122805	2.652848
H	-0.325679	1.118427	2.236247
C	0.970938	1.940213	3.753090
H	1.938583	1.581887	3.346930
H	0.619925	1.202771	4.504459
H	1.163839	2.885861	4.303341
C	-1.392780	2.670557	3.278717
H	-1.224701	3.684736	3.701346
H	-1.736238	2.011922	4.104822
H	-2.212000	2.742364	2.538150
C	-0.184115	-2.877976	-0.012803
C	0.077506	-3.699092	-1.156390
C	0.999626	-4.759298	-1.032043
H	1.202477	-5.397622	-1.907447
C	1.661631	-5.018625	0.175842
H	2.370797	-5.858821	0.251748
C	1.412450	-4.200532	1.285769
H	1.931281	-4.405384	2.236878
C	0.497356	-3.128652	1.214993
C	0.255232	-2.273285	2.456787
H	-0.402245	-1.434703	2.144284
C	-0.489519	-3.055328	3.559649
H	-1.460419	-3.442963	3.189773
H	-0.694713	-2.404883	4.436617
H	0.108112	-3.922052	3.916077
C	1.569689	-1.670658	2.987867
H	2.265855	-2.456519	3.352421
H	1.380847	-0.978884	3.833981
H	2.094631	-1.101470	2.188463
C	-0.613059	-3.475383	-2.504142
H	-1.194250	-2.535500	-2.424584
C	-1.605400	-4.614290	-2.827288
H	-1.081722	-5.588974	-2.934899
H	-2.133522	-4.411405	-3.783222
H	-2.371120	-4.723520	-2.032633
C	0.399641	-3.285914	-3.653443
H	1.111682	-2.462484	-3.445433
H	-0.132993	-3.036822	-4.595271
H	0.986906	-4.210706	-3.841723
C	5.896031	0.886895	-1.474534
C	7.376569	1.311735	-1.397784
H	8.059743	0.456698	-1.227472
H	7.565535	2.068557	-0.610981
H	7.665834	1.775506	-2.366383
C	5.682384	-0.038157	-2.695775
H	4.621967	-0.348708	-2.788147
H	6.336503	-0.931296	-2.690510
H	5.933697	0.541860	-3.610399
C	4.993546	2.123051	-1.719780
H	5.292112	2.590652	-2.683495
H	5.072352	2.898259	-0.937652
H	3.929161	1.822929	-1.814969
C	5.838901	-1.848408	0.170606
C	4.924478	-2.652266	-0.787964
H	4.974188	-2.316224	-1.838244
H	3.862452	-2.610119	-0.465924
H	5.233815	-3.720198	-0.761295
C	5.606705	-2.438948	1.579667
H	5.802100	-3.532385	1.533532
H	4.557154	-2.308267	1.912331
H	6.283908	-2.020022	2.348899
C	7.320363	-2.036021	-0.217839
H	8.009229	-1.476254	0.444937
H	7.527800	-1.738068	-1.263827
H	7.582103	-3.113534	-0.130621
C	5.827674	0.942733	1.713296
C	7.303102	0.688433	2.087889
H	8.005006	0.987550	1.285459
H	7.501692	-0.370650	2.343762
H	7.555575	1.290905	2.988211
C	5.607376	2.460599	1.518230
H	5.809069	2.967178	2.487187
H	4.561669	2.699481	1.234315
H	6.290025	2.907124	0.770361
C	4.903302	0.518266	2.879552
H	5.129565	1.148842	3.766912
H	5.027548	-0.536467	3.181694
H	3.837484	0.674126	2.613874
Cu	3.050421	-0.011529	-0.111050
O	1.165423	-0.021597	-0.569444
C	1.164650	-0.039246	-1.961381
O	-0.074995	-0.019259	-2.386239
O	2.217774	-0.068023	-2.597450

TS5-Cu

E= -4976.7474597

P	5.376455	-0.037160	0.025818
Al	-0.777574	-0.032838	-0.579011
O	-2.751132	0.022676	-0.940765
N	-1.127744	1.778974	-0.046632
N	-1.231165	-1.817645	-0.042121
C	-3.370844	1.242434	-0.632048
C	-2.450890	2.212626	-0.181463
C	-3.010052	3.480630	0.108909
H	-2.337223	4.287786	0.434057
C	-4.409781	3.701671	0.032792
C	-5.269095	2.635849	-0.322381
H	-6.354647	2.790113	-0.339432
C	-4.754852	1.360281	-0.658977
C	-5.581714	0.113787	-1.055461
C	-4.842995	-1.132270	-0.509375

C	-5.435164	-2.320626	-0.028465
H	-6.531358	-2.397630	-0.000810
C	-4.646960	-3.413624	0.416892
C	-3.236058	-3.310014	0.410261
H	-2.607750	-4.133597	0.774055
C	-2.594067	-2.127296	-0.037407
C	-3.451337	-1.118578	-0.515241
C	-5.362505	-4.694956	0.914520
C	-6.281370	-4.341511	2.112923
H	-5.692684	-3.918885	2.953764
H	-6.806141	-5.248738	2.482590
H	-7.055101	-3.596084	1.837506
C	-6.219777	-5.284249	-0.236079
H	-6.988927	-4.566829	-0.588206
H	-6.745734	-6.203760	0.100198
H	-5.585761	-5.551047	-1.107238
C	-4.368672	-5.781877	1.376646
H	-3.692804	-6.102816	0.557353
H	-4.925264	-6.679130	1.719520
H	-3.740539	-5.437257	2.223975
C	-5.625142	0.019212	-2.610238
H	-4.604926	-0.041157	-3.039283
H	-6.124331	0.913890	-3.037620
H	-6.185042	-0.885283	-2.927469
C	-7.020728	0.197492	-0.514292
H	-7.606776	-0.692899	-0.818926
H	-7.546365	1.081031	-0.928976
H	-7.040377	0.266989	0.592097
C	-4.959169	5.110926	0.370061
C	-4.587016	5.478885	1.829861
H	-3.489509	5.486424	1.988030
H	-5.025282	4.753406	2.546673
H	-4.970454	6.489819	2.086863
C	-4.336371	6.151154	-0.596619
H	-4.587291	5.914305	-1.651681
H	-3.231058	6.183596	-0.512789
H	-4.719584	7.170009	-0.372766
C	-6.494990	5.189980	0.233438
H	-6.841350	6.216725	0.474513
H	-7.010608	4.495376	0.928674
H	-6.831879	4.959052	-0.798486
C	-0.160573	2.771534	0.336965
C	0.180675	2.946099	1.715497
C	1.091286	3.966512	2.062881
H	1.355839	4.117566	3.120776
C	1.660988	4.801137	1.090841
H	2.359753	5.600220	1.387435
C	1.336992	4.610519	-0.258096
H	1.791173	5.262410	-1.022225
C	0.434299	3.603260	-0.661615
C	0.115871	3.448501	-2.150606
H	-0.489083	2.526919	-2.264556
C	1.391393	3.265096	-2.999617
H	2.020078	4.181714	-3.004626
H	1.124896	3.041092	-4.053601
H	2.014126	2.425822	-2.629400
C	-0.727752	4.628739	-2.680261
H	-1.677932	4.733729	-2.119160
H	-0.981259	4.476922	-3.751003
H	-0.174451	5.589651	-2.599535
C	-0.434000	2.071536	2.812888
H	-0.670797	1.091612	2.342951
C	0.524985	1.812570	3.990172
H	1.512458	1.441823	3.648491
H	0.091361	1.056882	4.677847
H	0.696046	2.729395	4.593982
C	-1.763422	2.654406	3.343888
H	-1.593166	3.647426	3.813515
H	-2.202539	1.985949	4.114964
H	-2.511766	2.783469	2.538560
C	-0.311056	-2.913957	0.088583
C	-0.056531	-3.764150	-1.035786
C	0.831966	-4.848053	-0.877757
H	1.030391	-5.507427	-1.738492
C	1.467346	-5.104330	0.344994
H	2.151878	-5.962053	0.446735
C	1.222584	-4.260696	1.436338

H	1.720419	-4.462583	2.399189
C	0.340553	-3.164158	1.332173
C	0.104881	-2.281989	2.556115
H	-0.542128	-1.442043	2.226100
C	-0.651188	-3.035206	3.671146
H	-1.623987	-3.422743	3.305755
H	-0.854325	-2.365223	4.533808
H	-0.063104	-3.899465	4.048787
C	1.426498	-1.684664	3.077909
H	2.118123	-2.476594	3.437285
H	1.243453	-0.997142	3.928536
H	1.943909	-1.115083	2.277447
C	-0.718420	-3.547412	-2.399469
H	-1.275508	-2.591049	-2.348297
C	-1.735199	-4.665532	-2.719313
H	-1.235672	-5.655364	-2.799679
H	-2.241291	-4.466878	-3.688001
H	-2.516952	-4.739553	-1.936429
C	0.317758	-3.407863	-3.534918
H	1.047546	-2.599363	-3.330155
H	-0.191988	-3.164348	-4.490816
H	0.883905	-4.350987	-3.694168
C	6.408192	0.333387	-1.590140
C	7.851810	0.818216	-1.346134
H	8.457614	0.091562	-0.770461
H	7.890253	1.793148	-0.821242
H	8.355523	0.961901	-2.327518
C	6.432862	-0.939370	-2.468000
H	5.409758	-1.325717	-2.654370
H	7.054697	-1.752995	-2.047679
H	6.868240	-0.673280	-3.455715
C	5.621996	1.391701	-2.405041
H	6.128607	1.531904	-3.385202
H	5.573123	2.380623	-1.916121
H	4.585197	1.050934	-2.608927
C	5.937563	-1.740688	0.784580
C	5.228450	-2.852986	-0.028062
H	5.544343	-2.899994	-1.085225
H	4.125154	-2.728599	-0.003599
H	5.461813	-3.837641	0.433340
C	5.406865	-1.862952	2.230632
H	5.593649	-2.901051	2.582128
H	4.313672	-1.690547	2.285662
H	5.913348	-1.183487	2.942736
C	7.463154	-1.972028	0.790819
H	8.008119	-1.197448	1.365399
H	7.895258	-2.015166	-0.227804
H	7.677390	-2.951126	1.273411
C	5.648084	1.389323	1.321086
C	7.001544	1.338333	2.059843
H	7.865994	1.404205	1.370821
H	7.118652	0.423870	2.673478
H	7.067536	2.204248	2.754990
C	5.509865	2.753569	0.607781
H	5.500974	3.551705	1.381404
H	4.555859	2.834244	0.047586
H	6.350119	2.973866	-0.078260
C	4.480932	1.314038	2.336029
H	4.536726	2.195433	3.011379
H	4.504491	0.409665	2.969509
H	3.499183	1.348362	1.819498
Cu	3.304579	-0.158293	-0.635004
O	1.149794	-0.096987	-0.487657
C	1.144139	-0.165796	-1.843225
O	-0.059512	-0.107016	-2.334057
O	2.237130	-0.273756	-2.451200

IM4-Cu

E= -4976.7515743

P	5.678449	-0.081282	-0.106863
Al	-0.815163	-0.044789	-0.491350
O	-2.759116	0.051644	-0.976304
N	-1.163256	1.785232	-0.002860
N	-1.352299	-1.807029	0.069263
C	-3.374086	1.284661	-0.720444
C	-2.467425	2.241302	-0.215378
C	-3.020882	3.521150	0.030593
H	-2.355834	4.318736	0.393397

C	-4.406668	3.769850	-0.150221
C	-5.259893	2.719024	-0.560367
H	-6.337530	2.895752	-0.660484
C	-4.749049	1.430397	-0.850662
C	-5.569412	0.200280	-1.307991
C	-4.898398	-1.062673	-0.714770
C	-5.550359	-2.242568	-0.294073
H	-6.646301	-2.299203	-0.357117
C	-4.820340	-3.353680	0.201780
C	-3.412538	-3.274254	0.316252
H	-2.833070	-4.114077	0.721022
C	-2.712923	-2.097252	-0.058474
C	-3.510832	-1.074564	-0.607054
C	-5.595644	-4.627805	0.622950
C	-6.594809	-4.273648	1.755068
H	-6.062570	-3.876169	2.644377
H	-7.163457	-5.175340	2.069479
H	-7.330789	-3.508141	1.434892
C	-6.375376	-5.182820	-0.597583
H	-7.102684	-4.445315	-0.994231
H	-6.942038	-6.096690	-0.316391
H	-5.683258	-5.448992	-1.423562
C	-4.660319	-5.741564	1.140527
H	-3.929305	-6.061816	0.369681
H	-5.258079	-6.633444	1.422966
H	-4.093145	-5.423560	2.039552
C	-5.503727	0.109441	-2.862181
H	-4.456852	0.027549	-3.217030
H	-5.951726	1.015397	-3.321458
H	-6.058728	-0.782339	-3.221407
C	-7.041483	0.312592	-0.870205
H	-7.622967	-0.564573	-1.218778
H	-7.518013	1.207935	-1.317687
H	-7.137930	0.379986	0.232278
C	-4.947957	5.197014	0.118981
C	-4.646313	5.606929	1.583470
H	-3.558368	5.610737	1.797681
H	-5.128984	4.909363	2.299461
H	-5.030431	6.629333	1.788912
C	-4.256587	6.195318	-0.845763
H	-4.459670	5.930359	-1.904436
H	-3.156274	6.207020	-0.707472
H	-4.628885	7.228116	-0.672388
C	-6.473070	5.297211	-0.099647
H	-6.814009	6.334614	0.100148
H	-7.035193	4.626119	0.582581
H	-6.760240	5.048550	-1.142365
C	-0.217893	2.750591	0.487105
C	-0.071050	2.959314	1.893415
C	0.820693	3.955128	2.345964
H	0.929676	4.128966	3.428941
C	1.562464	4.733295	1.447236
H	2.243985	5.515232	1.820297
C	1.427453	4.511093	0.070070
H	2.012250	5.122808	-0.636248
C	0.547886	3.530049	-0.434923
C	0.420517	3.353868	-1.949351
H	-0.152530	2.421594	-2.125559
C	1.790089	3.188512	-2.639940
H	2.399660	4.116373	-2.583413
H	1.653444	2.948216	-3.714911
H	2.376890	2.361762	-2.189372
C	-0.377425	4.513241	-2.586394
H	-1.388782	4.601240	-2.140810
H	-0.500394	4.350398	-3.678334
H	0.143169	5.485664	-2.446317
C	-0.861959	2.146947	2.920286
H	-1.323207	1.298703	2.371458
C	0.044964	1.560747	4.019990
H	0.883462	0.977165	3.589170
H	-0.537449	0.888893	4.685063
H	0.480375	2.354813	4.663799
C	-2.009260	2.968717	3.547765
H	-1.613456	3.845261	4.105460
H	-2.589894	2.348483	4.263593
H	-2.710935	3.342444	2.776471
C	-0.463135	-2.911239	0.306027
C	-0.138714	-3.801976	-0.766353
C	0.711503	-4.897635	-0.505292
H	0.958440	-5.591554	-1.325543
C	1.250120	-5.117738	0.767843
H	1.908337	-5.982468	0.952036
C	0.947342	-4.227287	1.807754
H	1.376574	-4.403880	2.805841
C	0.095371	-3.121450	1.604961
C	-0.246229	-2.195295	2.774647
H	-0.410824	-1.183443	2.344339
C	-1.565926	-2.615644	3.460546
H	-2.412502	-2.630491	2.746280
H	-1.824803	-1.913696	4.282086
H	-1.472024	-3.632462	3.899683
C	0.882956	-2.083995	3.814850
H	1.039240	-3.037045	4.364024
H	0.632706	-1.315854	4.574082
H	1.847681	-1.800338	3.345233
C	-0.678426	-3.617128	-2.187877
H	-1.231775	-2.657720	-2.211606
C	-1.665786	-4.740257	-2.574183
H	-1.165013	-5.732681	-2.588356
H	-2.081244	-4.561659	-3.588800
H	-2.515846	-4.795945	-1.864696
C	0.456435	-3.508318	-3.228787
H	1.163350	-2.691168	-2.983529
H	0.035342	-3.293505	-4.233538
H	1.034617	-4.454427	-3.307863
C	7.092114	0.434806	-1.338543
C	8.378086	0.948708	-0.659484
H	8.833448	0.196980	0.014624
H	8.209511	1.876475	-0.078431
H	9.130959	1.189272	-1.442059
C	7.435970	-0.772238	-2.240754
H	6.532596	-1.187584	-2.733306
H	7.954915	-1.587249	-1.700915
H	8.120534	-0.424848	-3.044758
C	6.513008	1.522164	-2.278862
H	7.249588	1.726314	-3.086495
H	6.304641	2.480503	-1.771549
H	5.571710	1.177655	-2.757701
C	6.053245	-1.825972	0.664164
C	5.698341	-2.891577	-0.403804
H	6.379242	-2.892088	-1.273312
H	4.661022	-2.755277	-0.777311
H	5.755998	-3.898382	0.064133
C	5.082102	-2.061657	1.843480
H	5.178626	-3.120045	2.168465
H	4.025131	-1.905512	1.545250
H	5.304309	-1.428497	2.723605
C	7.505286	-2.016471	1.147531
H	7.797273	-1.277673	1.919511
H	8.240920	-1.961849	0.320948
H	7.605763	-3.026223	1.602827
C	5.468720	1.253656	1.290587
C	6.544313	1.199879	2.394491
H	7.569487	1.349806	2.002485
H	6.525462	0.247367	2.959658
H	6.348691	2.013204	3.127304
C	5.470048	2.655805	0.640051
H	5.189588	3.398791	1.417055
H	4.715376	2.735395	-0.168942
H	6.459613	2.953945	0.243160
C	4.058535	1.062726	1.905181
H	3.835724	1.932314	2.560133
H	3.969586	0.152206	2.523537
H	3.267340	1.023502	1.126944
Cu	3.800910	-0.215397	-1.206659
O	1.088413	-0.130148	-0.315018
C	1.146435	-0.215871	-1.633345
O	-0.025197	-0.171876	-2.212082
O	2.242527	-0.330687	-2.275573

6-Cu

E= -9953.5553856

Cu	0.239653	-0.722769	-1.474352
P	0.371299	-1.665959	-3.488540
Al	-4.845686	-0.191654	-0.396005

O	-6.285382	0.380819	0.871713
O	-3.190801	-0.591206	-1.286060
O	-1.281663	-0.233648	-0.074740
O	-3.365141	0.098228	0.783583
N	-5.652641	-1.913914	-0.084623
N	-5.376287	1.445933	-1.271110
C	-7.153671	-0.646305	1.270610
C	-6.919244	1.587065	0.537103
C	-6.775407	-1.902897	0.754560
C	-8.041911	2.011474	1.242381
C	-6.383130	2.172371	-0.625178
C	-5.137947	-3.221982	-0.401502
C	-4.818642	2.055158	-2.450826
C	-8.610010	3.228851	0.808617
H	-9.492500	3.624380	1.330973
C	-8.295482	-0.353806	2.006473
C	-9.111659	-1.461466	2.338956
H	-10.024400	-1.291421	2.922580
C	-8.064477	3.947389	-0.286320
C	-8.541942	1.121717	2.405823
C	-10.028126	1.381473	2.713087
H	-10.188394	2.435736	3.016117
H	-10.370668	0.751643	3.558603
H	-10.671849	1.168643	1.835907
C	-5.476459	-3.859038	-1.635341
C	-4.865415	2.465231	-4.861620
H	-5.312184	2.284784	-5.852177
C	-3.716777	2.957025	-2.331560
C	-7.606748	-2.981692	1.143217
H	-7.348626	-3.992641	0.795614
C	-2.556861	-0.247735	-0.191231
C	-3.217691	3.586606	-3.491030
H	-2.370250	4.285554	-3.401261
C	-5.399914	1.806849	-3.733477
C	-5.041661	-5.183331	-1.856949
H	-5.307740	-5.687870	-2.798216
C	-8.707722	5.299351	-0.686730
C	-6.965619	3.412432	-0.999021
H	-6.562527	3.930915	-1.878268
C	-0.516590	-0.418665	-4.701325
C	-7.696248	1.438382	3.675335
H	-6.616915	1.264930	3.491045
H	-8.008881	0.789993	4.520340
H	-7.828562	2.499376	3.973826
C	-8.768379	-2.771559	1.930356
C	-0.558631	-3.382320	-3.546235
C	-3.785399	3.350926	-4.749555
H	-3.389631	3.860133	-5.643179
C	2.206728	-1.937026	-4.088858
C	2.988997	-2.593689	-2.927022
H	2.893748	-1.996319	-1.999366
H	4.068194	-2.615663	-3.194637
H	2.682660	-3.634637	-2.725354
C	-6.603208	0.879387	-3.914848
H	-6.659361	0.243936	-3.004995
C	-6.320186	-3.155927	-2.701470
H	-6.137640	-2.065089	-2.587200
C	-4.284602	-5.876616	-0.902962
H	-3.966926	-6.914461	-1.093706
C	-4.337769	-3.912804	0.561880
C	-10.198505	5.076355	-1.051812
H	-10.297708	4.375156	-1.906490
H	-10.677084	6.037295	-1.339060
H	-10.776109	4.655432	-0.203667
C	2.859651	-0.555707	-4.321701
H	2.458830	-0.028061	-5.208691
H	3.946772	-0.708562	-4.497616
H	2.763353	0.092892	-3.427702
C	-9.645163	-3.994565	2.300845
C	-7.928486	1.669707	-4.006110
H	-8.097386	2.292277	-3.105715
H	-8.790874	0.976938	-4.108412
H	-7.926627	2.340572	-4.892571
C	-2.381125	-3.264488	2.048380
H	-1.963878	-4.292827	2.099791
H	-2.094173	-2.746664	2.986849
H	-1.881231	-2.738680	1.210023
C	-3.085423	3.291162	-0.980341
H	-3.519600	2.593044	-0.236675
C	-3.928742	-5.235052	0.289099
H	-3.319944	-5.774083	1.033344
C	-6.456135	-0.049020	-5.135438
H	-6.490926	0.515031	-6.091770
H	-7.291706	-0.778926	-5.165614
H	-5.502899	-0.614821	-5.111231
C	-3.915195	-3.275900	1.887468
H	-4.251148	-2.219873	1.870699
C	-5.924808	-3.554204	-4.136129
H	-4.838189	-3.420532	-4.316305
H	-6.473864	-2.935650	-4.873420
H	-6.177428	-4.612856	-4.357740
C	-2.041649	-0.481453	-4.462997
H	-2.503151	-1.409380	-4.852339
H	-2.516069	0.370257	-4.997065
H	-2.299959	-0.381174	-3.389995
C	-7.831862	-3.387785	-2.479858
H	-8.078634	-4.468207	-2.565400
H	-8.429327	-2.843355	-3.242095
H	-8.159535	-3.042820	-1.479596
C	-8.611481	6.284824	0.507280
H	-9.133707	5.898011	1.406135
H	-9.071173	7.262066	0.244940
H	-7.552951	6.468049	0.786923
C	0.324201	-4.497851	-2.942391
H	1.185218	-4.773521	-3.581011
H	-0.304098	-5.407820	-2.827584
H	0.699609	-4.234494	-1.933038
C	-1.788997	-3.258689	-2.620624
H	-1.486265	-3.014533	-1.582081
H	-2.324995	-4.231835	-2.590984
H	-2.510048	-2.485469	-2.933381
C	-8.008596	5.947595	-1.900780
H	-6.937935	6.158441	-1.699951
H	-8.497703	6.913935	-2.144312
H	-8.068099	5.308449	-2.805727
C	-0.221653	-0.626187	-6.201030
H	0.842996	-0.456772	-6.456125
H	-0.815147	0.111940	-6.784556
H	-0.509220	-1.633251	-6.560054
C	-1.557939	3.083074	-0.979813
H	-1.278868	2.072246	-1.337766
H	-1.153625	3.186641	0.050172
H	-1.032959	3.826867	-1.615981
C	2.355398	-2.794790	-5.363081
H	2.006833	-3.836068	-5.218006
H	3.434030	-2.852032	-5.629854
H	1.823658	-2.371096	-6.236266
C	-4.585880	-3.963219	3.096617
H	-5.691007	-3.936906	3.016331
H	-4.301328	-3.454826	4.042463
H	-4.274184	-5.026688	3.182803
C	-3.442110	4.726042	-0.533021
H	-3.041172	5.480327	-1.244120
H	-3.010725	4.945086	0.466838
H	-4.539559	4.868036	-0.466770
C	-0.999532	-3.822965	-4.958685
H	-1.733159	-3.130143	-5.413280
H	-1.497473	-4.814834	-4.882992
H	-0.148725	-3.935719	-5.658749
C	-0.076494	1.004586	-4.282323
H	-0.302976	1.191434	-3.211437
H	-0.653976	1.747954	-4.873644
H	0.997999	1.201247	-4.445416
C	-10.862672	-3.603295	3.165769
H	-10.557005	-3.135824	4.124855
H	-11.455555	-4.508988	3.411509
H	-11.538678	-2.898873	2.638062
C	-8.796642	-5.017183	3.099911
H	-7.925767	-5.378698	2.516292
H	-9.409075	-5.903707	3.371798
H	-8.410421	-4.567157	4.038343
C	-10.171253	-4.664896	1.004927
H	-10.787937	-3.956466	0.413275
H	-10.801398	-5.546789	1.250223

H	-9.343802	-5.014213	0.354639
Cu	-0.239664	0.722405	1.474409
P	-0.371360	1.665732	3.488524
Al	4.845696	0.191556	0.396042
O	6.285441	-0.380822	-0.871625
O	3.190783	0.591069	1.286074
O	1.281676	0.233341	0.074762
O	3.365172	-0.098572	-0.783501
N	5.652449	1.913916	0.084501
N	5.376464	-1.445897	1.271269
C	7.153631	0.646356	-1.270605
C	6.919407	-1.587000	-0.536969
C	6.775227	1.902947	-0.754649
C	8.042112	-2.011344	-1.242227
C	6.383358	-2.172292	0.625350
C	5.137647	3.221954	0.401284
C	4.818854	-2.055124	2.451003
C	8.610318	-3.228649	-0.808402
H	9.492844	-3.624123	-1.330740
C	8.295468	0.353920	-2.006449
C	9.111529	1.461641	-2.339012
H	10.024280	1.291652	-2.922635
C	8.064853	-3.947182	0.286572
C	8.542066	-1.121604	-2.405716
C	10.028276	-1.381238	-2.712960
H	10.188643	-2.435502	-3.015936
H	10.370760	-0.751421	-3.558509
H	10.671977	-1.168303	-1.835790
C	5.476052	3.859107	1.635100
C	4.865449	-2.464908	4.861850
H	5.312112	-2.284295	5.852426
C	3.717089	-2.957105	2.331744
C	7.606466	2.981799	-1.143375
H	7.348244	3.992744	-0.795829
C	2.556867	0.247438	0.191281
C	3.217948	-3.586592	3.491243
H	2.370576	-4.285623	3.401474
C	5.399994	-1.806618	3.733675
C	5.041277	5.183432	1.856554
H	5.307307	5.688050	2.797793
C	8.708217	-5.299065	0.687050
C	6.965954	-3.412282	0.999252
H	6.562908	-3.930759	1.878526
C	0.516556	0.418525	4.701388
C	7.696409	-1.438417	-3.675216
H	6.617058	-1.265059	-3.490941
H	8.008985	-0.790043	-4.520253
H	7.828828	-2.499414	-3.973649
C	8.768116	2.771729	-1.930500
C	0.558575	3.382086	3.546082
C	3.785511	-3.350700	4.749794
H	3.389690	-3.859824	5.643442
C	-2.206778	1.936852	4.088799
C	-2.988991	2.593567	2.926952
H	-2.893716	1.996237	1.999273
H	-4.068197	2.615541	3.194530
H	-2.682635	3.634523	2.725348
C	6.603154	-0.878993	3.915078
H	6.659374	-0.243681	3.005135
C	6.319706	3.156075	2.701328
H	6.136989	2.065241	2.587275
C	4.284342	5.876664	0.902428
H	3.966717	6.914550	1.093035
C	4.337588	3.912722	-0.562233
C	10.198995	-5.075929	1.052065
H	10.298174	-4.374669	1.906696
H	10.677659	-6.036814	1.339357
H	10.776540	-4.655015	0.203875
C	-2.859716	0.555531	4.321587
H	-2.458891	0.027831	5.208545
H	-3.946835	0.708377	4.497514
H	-2.763399	-0.093014	3.427547
C	9.644780	3.994798	-2.301057
C	7.928507	-1.669145	4.006677
H	8.097635	-2.291819	3.106396
H	8.790795	-0.976258	4.109026
H	7.926581	-2.339885	4.893231

C	2.381071	3.264276	-2.048808
H	1.963835	4.292615	-2.100304
H	2.094170	2.746385	-2.987256
H	1.881118	2.738532	-1.210445
C	3.085908	-3.291450	0.980499
H	3.520213	-2.593466	0.236784
C	3.928584	5.235010	-0.289617
H	3.319885	5.773994	-1.033978
C	6.455771	0.049576	5.135509
H	6.490604	-0.514320	6.091932
H	7.291179	0.779673	5.165638
H	5.502409	0.615157	5.111135
C	3.915134	3.275706	-1.887804
H	4.251106	2.219686	-1.870922
C	5.924440	3.554691	4.135922
H	4.837796	3.421277	4.316149
H	6.473384	2.936152	4.873308
H	6.177280	4.613332	4.357340
C	2.041620	0.481329	4.463075
H	2.503105	1.409290	4.852355
H	2.516045	-0.370337	4.997210
H	2.299951	0.380992	3.390086
C	7.831414	3.387662	2.479616
H	8.078341	4.468064	2.564954
H	8.428827	2.843280	3.241931
H	8.158993	3.042473	1.479401
C	8.612012	-6.284623	-0.506894
H	9.134177	-5.897825	-1.405790
H	9.071787	-7.261813	-0.244508
H	7.553487	-6.467947	-0.786489
C	-0.324283	4.497604	2.942256
H	-1.185230	4.773332	3.580947
H	0.304030	5.407549	2.827340
H	-0.699801	4.234216	1.932953
C	1.788878	3.258363	2.620405
H	1.486067	3.014216	1.581882
H	2.324956	4.231461	2.590725
H	2.509881	2.485083	2.933124
C	8.009186	-5.947282	1.901169
H	6.938538	-6.158238	1.700390
H	8.498386	-6.913562	2.144755
H	8.068662	-5.308064	2.806066
C	0.221574	0.626146	6.201074
H	-0.843085	0.456718	6.456130
H	0.815068	-0.111916	6.784681
H	0.509091	1.633246	6.560036
C	1.558427	-3.083311	0.979719
H	1.279339	-2.072460	1.337596
H	1.154280	-3.186872	-0.050339
H	1.033315	-3.827070	1.615819
C	-2.355457	2.794620	5.363021
H	-2.006824	3.835876	5.217953
H	-3.434098	2.851928	5.629741
H	-1.823783	2.370896	6.236230
C	4.585909	3.962925	-3.096959
H	5.691028	3.936668	-3.016541
H	4.301483	3.454417	-4.042781
H	4.274183	5.026371	-3.183302
C	3.442610	-4.726417	0.533467
H	3.041671	-5.480557	1.244719
H	3.011228	-4.945668	-0.466346
H	4.540061	-4.868416	0.467253
C	0.999582	3.822805	4.958480
H	1.733165	3.129964	5.413112
H	1.497604	4.814624	4.882676
H	0.148811	3.935700	5.658568
C	0.076492	-1.004769	4.282492
H	0.302977	-1.191700	3.211621
H	0.654005	-1.748068	4.873870
H	-0.997993	-1.201456	4.445611
C	10.862310	3.603606	-3.165985
H	10.556670	3.136071	-4.125049
H	11.455108	4.509343	-3.411769
H	11.538388	2.899264	-2.638263
C	8.796153	5.017307	-3.100150
H	7.925255	5.378769	-2.516530
H	9.408500	5.903875	-3.372085

H	8.409956	4.567207	-4.038556
C	10.170828	4.665226	-1.005171
H	10.787591	3.956877	-0.413506
H	10.800882	5.547172	-1.250507
H	9.343352	5.014480	-0.354880

3-Au

E= -3207.8156206

Au	2.032269	-0.021038	-0.417378
P	4.570106	-0.024662	-0.484794
Al	-0.393637	-0.001061	-0.061961
O	-2.288092	0.003758	-0.948058
N	-1.012264	1.807850	0.336454
N	-1.030148	-1.799373	0.357448
C	-3.030138	1.180041	-0.742256
C	-2.306802	2.165695	-0.041191
C	-2.999662	3.378868	0.192064
H	-2.469586	4.190940	0.711438
C	-4.354634	3.537802	-0.193489
C	-5.028816	2.468418	-0.828408
H	-6.085896	2.572725	-1.100810
C	-4.368339	1.250205	-1.115184
C	-4.990581	0.010888	-1.797661
C	-4.378403	-1.230825	-1.110887
C	-5.042303	-2.441051	-0.817941
H	-6.101521	-2.548240	-1.091215
C	-4.374104	-3.514727	-0.174667
C	-3.024947	-3.361203	0.217451
H	-2.496726	-4.167311	0.742919
C	-2.325159	-2.149421	-0.024960
C	-3.037672	-1.167121	-0.735581
C	-5.155499	-4.824140	0.100146
C	-6.383616	-4.514701	0.995493
H	-6.067480	-4.083388	1.968152
H	-6.958830	-5.442903	1.201945
H	-7.075396	-3.791786	0.516935
C	-5.637552	-5.426154	-1.245573
H	-6.301423	-4.728889	-1.796426
H	-6.205478	-6.365612	-1.072137
H	-4.777323	-5.662564	-1.906105
C	-4.293424	-5.883587	0.819186
H	-3.404819	-6.172526	0.220759
H	-4.892050	-6.802709	0.990152
H	-3.939167	-5.528680	1.808882
C	-4.578539	0.006621	-3.300306
H	-3.476299	0.001517	-3.416917
H	-4.973362	0.909284	-3.811776
H	-4.981457	-0.894015	-3.809052
C	-6.526312	0.016636	-1.698384
H	-6.955410	-0.871980	-2.203761
H	-6.949245	0.906953	-2.205884
H	-6.870708	0.019077	-0.644544
C	-5.070663	4.874059	0.127685
C	-5.093899	5.087026	1.664035
H	-4.071061	5.127112	2.090613
H	-5.637282	4.262471	2.171012
H	-5.602838	6.041717	1.918475
C	-4.310771	6.047907	-0.541505
H	-4.278167	5.924436	-1.644185
H	-3.265519	6.125123	-0.179110
H	-4.813036	7.013980	-0.319508
C	-6.527960	4.895986	-0.381732
H	-6.996342	5.872686	-0.138969
H	-7.146594	4.106231	0.092713
H	-6.584190	4.764147	-1.482328
C	-0.131283	2.874523	0.715541
C	0.236221	3.055107	2.084866
C	1.089889	4.126223	2.423302
H	1.375389	4.278874	3.475229
C	1.575665	5.009591	1.449088
H	2.233544	5.845262	1.737735
C	1.221353	4.819621	0.107145
H	1.612469	5.509494	-0.658867
C	0.375928	3.761513	-0.286425
C	0.048333	3.592450	-1.772830
H	-0.660569	2.745566	-1.863337
C	1.304943	3.215939	-2.584473
H	2.064207	4.027338	-2.561933
H	1.045732	3.030571	-3.648843
H	1.770836	2.292971	-2.177201
C	-0.644093	4.836561	-2.366951
H	-1.571041	5.086950	-1.813232
H	-0.920464	4.658368	-3.427924
H	0.019707	5.727556	-2.343761
C	-0.321197	2.140951	3.178068
H	-0.450465	1.135788	2.719024
C	0.617118	1.981103	4.387053
H	1.635373	1.663054	4.082144
H	0.215414	1.218307	5.085228
H	0.713799	2.923578	4.967320
C	-1.720929	2.607197	3.638028
H	-1.665628	3.619577	4.093548
H	-2.137245	1.914002	4.399894
H	-2.435686	2.652453	2.792833
C	-0.162546	-2.868004	0.759735
C	0.325037	-3.790703	-0.219199
C	1.154168	-4.851579	0.199997
H	1.530331	-5.568814	-0.548010
C	1.514678	-5.008247	1.544637
H	2.161459	-5.845506	1.852961
C	1.049737	-4.089385	2.495801
H	1.338645	-4.216906	3.550179
C	0.208698	-3.016654	2.131902
C	-0.339466	-2.070962	3.202402
H	-0.457224	-1.074920	2.720698
C	-1.745412	-2.512766	3.668015
H	-2.456317	-2.573933	2.820429
H	-2.159040	-1.796605	4.409857
H	-1.700806	-3.513738	4.149145
C	0.597434	-1.893509	4.409712
H	0.684695	-2.824002	5.010416
H	0.201741	-1.111861	5.090159
H	1.619170	-1.591309	4.100113
C	-0.002540	-3.653222	-1.708985
H	-0.732059	-2.826343	-1.816542
C	-0.663296	-4.922090	-2.286363
H	0.022622	-5.795868	-2.257706
H	-0.950263	-4.763001	-3.347453
H	-1.580594	-5.189803	-1.723526
C	1.249248	-3.256956	-2.520090
H	1.682133	-2.310001	-2.128969
H	0.993675	-3.104575	-3.590629
H	2.033391	-4.042972	-2.469327
C	5.267333	-0.226016	-2.297903
C	6.746351	0.175019	-2.480518
H	7.430601	-0.419163	-1.844031
H	6.926455	1.247704	-2.273239
H	7.041994	-0.003321	-3.538593
C	5.087691	-1.690392	-2.758731
H	4.045160	-2.043466	-2.627737
H	5.768794	-2.395691	-2.245011
H	5.321382	-1.745009	-3.844666
C	4.370409	0.630028	-3.226625
H	4.675856	0.454211	-4.281745
H	4.447631	1.715126	-3.036051
H	3.303115	0.342192	-3.128171
C	5.217258	-1.498064	0.615444
C	4.348637	-2.737809	0.288347
H	4.501190	-3.125567	-0.734175
H	3.266787	-2.525768	0.416890
H	4.614501	-3.554416	0.994467
C	4.947424	-1.168445	2.101832
H	5.152579	-2.080554	2.703679
H	3.887477	-0.890633	2.275151
H	5.597938	-0.364447	2.496506
C	6.710487	-1.844468	0.437573
H	7.380368	-0.995605	0.675404
H	6.946088	-2.193079	-0.587019
H	6.972908	-2.676286	1.128717
C	5.211896	1.660812	0.262582
C	6.687219	1.659877	0.715956
H	7.390236	1.421599	-0.105760
H	6.875906	0.953611	1.548149
H	6.949029	2.674700	1.090205
C	5.004495	2.786189	-0.776730

H	5.212400	3.759546	-0.281321
H	3.958710	2.824219	-1.141963
H	5.686938	2.711665	-1.645256
C	4.297457	2.013300	1.462848
H	4.565406	3.029243	1.826333
H	4.404277	1.318961	2.315061
H	3.227284	2.035448	1.168331

TS1-Au

E= -3396.4945447

Au	2.009243	-0.011264	-0.393693
P	4.598517	-0.017101	-0.085654
Al	-0.478995	-0.004637	-0.313288
O	-2.493808	0.020380	-0.819936
N	-0.999857	1.831814	0.114878
N	-1.043400	-1.828670	0.116004
C	-3.178909	1.212338	-0.593895
C	-2.328571	2.217752	-0.089768
C	-2.948008	3.466049	0.159156
H	-2.329469	4.293015	0.537508
C	-4.342597	3.646008	-0.034792
C	-5.131746	2.561290	-0.481284
H	-6.213374	2.688584	-0.609867
C	-4.554483	1.300738	-0.770942
C	-5.306047	0.055890	-1.297387
C	-4.588903	-1.201282	-0.752069
C	-5.201580	-2.439550	-0.440392
H	-6.287194	-2.536703	-0.561465
C	-4.442644	-3.539669	0.020554
C	-3.041673	-3.399711	0.200106
H	-2.445794	-4.239094	0.587253
C	-2.385927	-2.175114	-0.073075
C	-3.210420	-1.150430	-0.581346
C	-5.104631	-4.898602	0.362547
C	-4.881465	-5.217589	1.863924
H	-3.803639	-5.278692	2.117466
H	-5.345502	-6.191992	2.129333
H	-5.334562	-4.435129	2.507870
C	-6.625187	-4.894164	0.094889
H	-7.153715	-4.138248	0.712003
H	-7.054338	-5.886239	0.347692
H	-6.857991	-4.693050	-0.971507
C	-4.468795	-6.019392	-0.499613
H	-4.612054	-5.819064	-1.582043
H	-4.934732	-7.001374	-0.268030
H	-3.379469	-6.115632	-0.315597
C	-5.207518	0.042445	-2.852639
H	-4.150621	0.025326	-3.186757
H	-5.687930	0.947784	-3.279438
H	-5.712783	-0.855832	-3.265427
C	-6.790473	0.079787	-0.885762
H	-7.324930	-0.804590	-1.287115
H	-7.299883	0.972888	-1.300136
H	-6.910152	0.089635	0.216485
C	-4.963994	5.032235	0.271939
C	-4.736919	5.382612	1.765592
H	-3.658780	5.420045	2.022338
H	-5.214499	4.630124	2.427319
H	-5.173809	6.376267	2.004268
C	-4.290514	6.110531	-0.615618
H	-4.435441	5.886697	-1.693204
H	-3.199417	6.177431	-0.428885
H	-4.726581	7.112132	-0.411459
C	-6.483010	5.067741	-0.002169
H	-6.882517	6.078214	0.225057
H	-7.036545	4.343203	0.630488
H	-6.718108	4.848417	-1.064439
C	-0.107631	2.872406	0.547523
C	0.152894	3.065289	1.940947
C	0.975996	4.141485	2.335474
H	1.175022	4.303843	3.406292
C	1.539373	5.015534	1.395976
H	2.171011	5.855227	1.728290
C	1.292787	4.812558	0.032274
H	1.738306	5.499015	-0.706490
C	0.475434	3.753499	-0.416039
C	0.227400	3.598287	-1.917542
H	-0.365985	2.671509	-2.051793
C	1.546629	3.428844	-2.698955
H	2.171596	4.346614	-2.654561
H	1.346916	3.220927	-3.771719
H	2.149111	2.590608	-2.291742
C	-0.603321	4.766155	-2.491797
H	-1.579225	4.861263	-1.975451
H	-0.805468	4.607615	-3.572612
H	-0.066149	5.734033	-2.391120
C	-0.472866	2.164896	3.008967
H	-0.718994	1.202102	2.510521
C	0.481320	1.859374	4.178535
H	1.442944	1.432356	3.828281
H	0.015039	1.129936	4.872381
H	0.709302	2.766179	4.778235
C	-1.798187	2.754145	3.543056
H	-1.619395	3.730029	4.044587
H	-2.259819	2.070621	4.287292
H	-2.532060	2.918541	2.730055
C	-0.179891	-2.900544	0.530723
C	0.318658	-3.826593	-0.438358
C	1.085272	-4.927302	-0.001373
H	1.461593	-5.650048	-0.743901
C	1.375617	-5.120168	1.354796
H	1.968949	-5.990527	1.678702
C	0.910260	-4.193304	2.297628
H	1.148268	-4.345676	3.361668
C	0.131334	-3.080447	1.915398
C	-0.399376	-2.124508	2.986328
H	-0.552039	-1.141265	2.489141
C	-1.774770	-2.580508	3.524346
H	-2.523988	-2.674447	2.714194
H	-2.165136	-1.854877	4.269507
H	-1.689667	-3.568806	4.026060
C	0.582420	-1.913501	4.153182
H	0.709518	-2.833410	4.762709
H	0.202318	-1.128318	4.837691
H	1.585602	-1.600258	3.798523
C	0.044950	-3.668367	-1.935306
H	-0.502150	-2.712627	-2.063435
C	-0.855234	-4.794329	-2.487519
H	-0.365477	-5.787670	-2.393393
H	-1.070398	-4.629727	-3.564890
H	-1.824011	-4.838108	-1.951408
C	1.356649	-3.572870	-2.743415
H	2.013856	-2.768982	-2.352187
H	1.146760	-3.357208	-3.812592
H	1.930338	-4.523735	-2.706840
C	5.602452	0.010791	-1.760354
C	7.099651	0.360828	-1.620279
H	7.645662	-0.340141	-0.960006
H	7.262857	1.389384	-1.243085
H	7.576957	0.307603	-2.624308
C	5.470640	-1.364563	-2.453785
H	4.411998	-1.678345	-2.555190
H	6.033732	-2.167781	-1.940780
H	5.887751	-1.278884	-3.481083
C	4.917149	1.032619	-2.700420
H	5.426810	1.000772	-3.688810
H	4.972865	2.073136	-2.335710
H	3.852496	0.777758	-2.867177
C	5.053809	-1.641219	0.894290
C	4.244420	-2.801215	0.263426
H	4.557140	-3.047114	-0.766710
H	3.156277	-2.581658	0.249648
H	4.389411	-3.714361	0.880651
C	4.545525	-1.507055	2.347889
H	4.641843	-2.496865	2.844737
H	3.472945	-1.226549	2.383916
H	5.125797	-0.781515	2.949723
C	6.552894	-2.006939	0.918795
H	7.182493	-1.212838	1.364859
H	6.950733	-2.239101	-0.088578
H	6.689643	-2.922657	1.536261
C	5.108114	1.535485	0.982137
C	6.479706	1.432601	1.682497
H	7.315742	1.290457	0.970595
H	6.517511	0.613800	2.427343

H	6.674060	2.379017	2.234823
C	5.093855	2.798620	0.091062
H	5.212686	3.687808	0.747828
H	4.130322	2.919075	-0.444352
H	5.921506	2.824371	-0.643695
C	3.995712	1.747033	2.038862
H	4.207737	2.684836	2.597341
H	3.930798	0.929367	2.778518
H	2.999657	1.863851	1.562229
C	0.737544	-0.072969	-2.865895
O	1.698276	-0.132925	-3.546980
O	-0.435384	-0.020123	-2.584549

IM1-Au

E= -3396.5211499

Au	2.188389	-0.023513	-0.769827
P	4.516992	-0.023039	0.192501
Al	-0.613362	-0.004116	-0.635900
O	-2.579272	0.023273	-0.926356
N	-0.983717	1.804180	-0.029818
N	-1.035738	-1.791865	0.004138
C	-3.228969	1.213217	-0.595989
C	-2.330954	2.193584	-0.129088
C	-2.925113	3.439843	0.186849
H	-2.277560	4.261523	0.525274
C	-4.329222	3.631437	0.092493
C	-5.160191	2.557818	-0.303889
H	-6.247620	2.693376	-0.346254
C	-4.612980	1.301716	-0.660359
C	-5.399593	0.059831	-1.147981
C	-4.644692	-1.206371	-0.673292
C	-5.224617	-2.449989	-0.325407
H	-6.315002	-2.558035	-0.370550
C	-4.421554	-3.545237	0.069462
C	-3.014147	-3.388044	0.171068
H	-2.389989	-4.224103	0.518008
C	-2.387927	-2.153215	-0.129441
C	-3.258708	-1.152534	-0.604836
C	-5.040582	-4.923857	0.414296
C	-4.707110	-5.293690	1.882782
H	-3.613645	-5.351115	2.057512
H	-5.141012	-6.283078	2.143282
H	-5.121582	-4.541784	2.586410
C	-6.576423	-4.932772	0.256412
H	-7.068217	-4.204248	0.934006
H	-6.973853	-5.938462	0.507144
H	-6.888205	-4.702356	-0.783472
C	-4.453671	-6.002481	-0.532696
H	-4.679229	-5.765936	-1.593528
H	-4.886858	-6.999950	-0.303659
H	-3.352286	-6.084886	-0.433144
C	-5.415728	0.068716	-2.706224
H	-4.387274	0.057292	-3.119501
H	-5.927041	0.979365	-3.082536
H	-5.950864	-0.823948	-3.092363
C	-6.848468	0.075475	-0.625226
H	-7.410823	-0.804190	-0.997771
H	-7.387745	0.973676	-0.987568
H	-6.884969	0.069617	0.482960
C	-4.913115	5.027601	0.428748
C	-4.539466	5.420505	1.881079
H	-3.441624	5.470990	2.028275
H	-4.945232	4.687339	2.609121
H	-4.955473	6.419660	2.133008
C	-4.326623	6.075597	-0.552718
H	-4.582954	5.823877	-1.603004
H	-3.221489	6.134352	-0.481168
H	-4.732289	7.086676	-0.332880
C	-6.451800	5.064525	0.306158
H	-6.823984	6.080460	0.554322
H	-6.941194	4.352558	1.002874
H	-6.792329	4.829460	-0.723538
C	-0.093646	2.851694	0.396918
C	0.099463	3.103542	1.791175
C	0.873753	4.216673	2.182194
H	1.013455	4.425377	3.255529
C	1.459990	5.067182	1.235701
H	2.052813	5.937074	1.562132
C	1.283457	4.804086	-0.129061
H	1.742212	5.475278	-0.873301
C	0.506488	3.713277	-0.574953
C	0.285704	3.527843	-2.077183
H	-0.228775	2.556641	-2.215314
C	1.611364	3.468080	-2.862264
H	2.157796	4.435345	-2.830998
H	1.421463	3.225811	-3.928228
H	2.281416	2.680311	-2.460140
C	-0.642061	4.622754	-2.649628
H	-1.621111	4.637551	-2.129656
H	-0.832974	4.444768	-3.729159
H	-0.186670	5.632032	-2.550112
C	-0.532506	2.226707	2.874105
H	-0.983143	1.352160	2.359276
C	0.517624	1.700901	3.872028
H	1.306183	1.111378	3.361726
H	0.041148	1.046704	4.630821
H	1.015856	2.528062	4.421106
C	-1.666673	2.959937	3.622955
H	-1.280876	3.849965	4.165746
H	-2.137765	2.288882	4.372392
H	-2.458649	3.300195	2.927552
C	-0.176206	-2.861663	0.441062
C	0.371780	-3.765996	-0.522235
C	1.108290	-4.881138	-0.067986
H	1.525266	-5.585684	-0.805975
C	1.301239	-5.120751	1.297718
H	1.863621	-6.007211	1.633171
C	0.776380	-4.221879	2.236326
H	0.937752	-4.412229	3.308666
C	0.040743	-3.084936	1.838996
C	-0.528898	-2.148972	2.910577
H	-0.665657	-1.156704	2.427601
C	-1.916338	-2.609370	3.413132
H	-2.654247	-2.681219	2.591656
H	-2.312282	-1.897445	4.168444
H	-1.844824	-3.608241	3.895708
C	0.417169	-1.970356	4.113427
H	0.511937	-2.904275	4.707236
H	0.020929	-1.196691	4.802061
H	1.435676	-1.662837	3.802810
C	0.153040	-3.593358	-2.026595
H	-0.340167	-2.613183	-2.177799
C	-0.792437	-4.675298	-2.593949
H	-0.357907	-5.692508	-2.482640
H	-0.972319	-4.504021	-3.676438
H	-1.775168	-4.665614	-2.081280
C	1.483518	-3.569454	-2.806541
H	2.171068	-2.796457	-2.405784
H	1.303973	-3.328951	-3.874698
H	2.005834	-4.549580	-2.766142
C	5.643466	-0.517542	-1.329474
C	7.142893	-0.208605	-1.125088
H	7.572713	-0.707068	-0.235280
H	7.345916	0.877443	-1.047440
H	7.704546	-0.576543	-2.011992
C	5.473159	-2.026867	-1.617800
H	4.408726	-2.309900	-1.746822
H	5.923064	-2.674819	-0.841499
H	5.990146	-2.256443	-2.574658
C	5.137742	0.224028	-2.592966
H	5.749013	-0.115611	-3.457744
H	5.243577	1.321392	-2.531786
H	4.078413	-0.007313	-2.829608
C	4.757580	-1.322740	1.618221
C	3.915716	-2.569276	1.255294
H	4.282667	-3.105313	0.362899
H	2.849235	-2.315633	1.084779
H	3.952514	-3.282789	2.106373
C	4.161102	-0.754002	2.924802
H	4.140149	-1.568659	3.680656
H	3.117578	-0.405900	2.787600
H	4.757120	0.073823	3.354490
C	6.221133	-1.739060	1.879352
H	6.871803	-0.883991	2.147027
H	6.674520	-2.264352	1.016652

```
H      6.241295   -2.449582    2.735061
C      5.021763    1.747131    0.818623
C      6.327965    1.793241    1.640120
H      7.205788    1.423585    1.076132
H      6.257500    1.221210    2.585784
H      6.535790    2.849917    1.918880
C      5.153022    2.698692   -0.392190
H      5.265054    3.734863   -0.005976
H      4.247196    2.687218   -1.031626
H      6.039313    2.486853   -1.020367
C      3.848615    2.287192    1.669353
H      4.063561    3.342711    1.941615
H      3.693664    1.731582    2.610585
H      2.894394    2.284304    1.103763
C      0.993585   -0.047943   -2.511336
O      1.690260   -0.077294   -3.512094
O     -0.312538   -0.030471   -2.457759
```

TS2-Au

E= -3396.505857

```
Au     3.256590   -0.207034   -0.834513
P      5.450302   -0.074845    0.163126
Al    -1.190010   -0.080238   -0.460098
O     -3.104476    0.096206   -0.996072
N     -1.766508   -1.802012    0.083728
N     -1.423218    1.745083    0.008150
C     -3.904175   -0.982782   -0.558785
C     -3.147479   -2.020742    0.020763
C     -3.895384   -3.133273    0.471569
H     -3.352191   -3.985665    0.905716
C     -5.312050   -3.140798    0.401745
C     -5.993465   -2.022781   -0.136258
H     -7.089590   -2.014519   -0.167261
C     -5.288942   -0.901269   -0.634771
C     -5.905590    0.372760   -1.261344
C     -5.028248    1.567789   -0.819493
C     -5.478202    2.869477   -0.495048
H     -6.548618    3.093990   -0.573721
C     -4.575567    3.872984   -0.070055
C     -3.202159    3.558220    0.095225
H     -2.498300    4.315209    0.470895
C     -2.710681    2.261089   -0.185270
C     -3.658443    1.361593   -0.712755
C     -5.050586    5.316150    0.234612
C     -4.329492    6.300552   -0.722726
H     -3.227767    6.260069   -0.602709
H     -4.652126    7.345085   -0.523338
H     -4.561061    6.066425   -1.782770
C     -6.572904    5.485442    0.041135
H     -6.885623    5.271045   -1.001833
H     -6.865583    6.532224    0.266052
H     -7.153986    4.826111    0.718995
C     -4.709590    5.683503    1.701777
H     -5.207064    4.990662    2.412190
H     -5.051271    6.715127    1.933112
H     -3.619019    5.642949    1.897973
C     -7.371929    0.560470   -0.832654
H     -7.470409    0.656666    0.267463
H     -7.990110   -0.297564   -1.165080
H     -7.805369    1.466166   -1.302418
C     -5.839507    0.246693   -2.812932
H     -6.247252    1.161253   -3.291794
H     -6.432473   -0.626933   -3.155076
H     -4.797233    0.111988   -3.164981
C     -6.078359   -4.366604    0.959834
C     -5.601237   -5.655205    0.242422
H     -4.517876   -5.838444    0.391968
H     -5.785637   -5.595307   -0.850396
H     -6.145803   -6.541059    0.633747
C     -5.799432   -4.491329    2.480830
H     -6.139849   -3.584454    3.022992
H     -4.718791   -4.623412    2.692236
H     -6.335625   -5.367237    2.905397
C     -7.604492   -4.245481    0.759396
H     -8.108191   -5.150502    1.158403
H     -7.876056   -4.158962   -0.313270
H     -8.027345   -3.370357    1.295062
C     -0.905450   -2.947292    0.214588
C     -0.758521   -3.847979   -0.886491
C      0.088948   -4.964949   -0.731365
H      0.209889   -5.665939   -1.573222
C      0.788109   -5.193549    0.459492
H      1.446361   -6.071912    0.557102
C      0.652344   -4.293548    1.525820
H      1.209686   -4.478086    2.456367
C     -0.188164   -3.165174    1.430082
C     -0.351526   -2.220567    2.624354
H     -0.473243   -1.192927    2.212303
C      0.867577   -2.196335    3.562387
H      1.002536   -3.163044    4.092455
H      0.736641   -1.419546    4.342609
H      1.805262   -1.975628    3.011709
C     -1.636963   -2.537252    3.421517
H     -2.539950   -2.482857    2.782225
H     -1.769338   -1.820704    4.260229
H     -1.584980   -3.559775    3.853625
C     -1.480464   -3.658525   -2.225144
H     -2.075641   -2.725423   -2.158680
C     -0.499033   -3.487681   -3.404938
H      0.148074   -2.594876   -3.287767
H     -1.066036   -3.370354   -4.353468
H      0.154920   -4.378228   -3.523217
C     -2.469989   -4.812951   -2.498532
H     -1.938840   -5.781751   -2.618264
H     -3.033648   -4.626559   -3.437246
H     -3.205799   -4.927128   -1.677303
C     -0.412886    2.674377    0.442338
C     -0.190464    2.881035    1.837966
C      0.747277    3.856931    2.237391
H      0.917901    4.033631    3.311674
C      1.458730    4.610740    1.294819
H      2.178154    5.376880    1.626573
C      1.252919    4.381928   -0.072533
H      1.822476    4.968666   -0.810772
C      0.326963    3.419597   -0.527346
C      0.124729    3.227136   -2.031766
H     -0.417878    2.269271   -2.166059
C     -0.752428    4.348860   -2.632426
H     -1.742508    4.406727   -2.137018
H     -0.922731    4.172636   -3.715827
H     -0.259357    5.339557   -2.525904
C      1.457346    3.108049   -2.796973
H      2.016289    4.068615   -2.810755
H      1.270087    2.820107   -3.852066
H      2.110614    2.331601   -2.349002
C     -0.949884    2.093436    2.907836
H     -1.456827    1.251208    2.390013
C     -2.049338    2.944474    3.580496
H     -1.609073    3.817690    4.108893
H     -2.607470    2.343572    4.329826
H     -2.779615    3.326072    2.840029
C     -0.006138    1.494437    3.969509
H      0.808789    0.902577    3.505672
H     -0.568825    0.828575    4.657219
H      0.464583    2.282494    4.595204
C      5.997791   -1.864588    0.709357
C      7.511833   -2.028945    0.958876
H      7.895985   -1.342927    1.738596
H      8.112453   -1.880033    0.040143
H      7.708530   -3.067194    1.307048
C      5.227629   -2.253169    1.992205
H      4.132483   -2.127653    1.869492
H      5.556088   -1.689731    2.886583
H      5.414957   -3.329585    2.197941
C      5.536519   -2.859747   -0.385923
H      5.745124   -3.893134   -0.031645
H      6.059622   -2.729869   -1.349513
H      4.446057   -2.777482   -0.575498
C      5.451541    1.111370    1.708642
C      4.159570    0.831567    2.516965
H      4.148798   -0.161631    2.999697
H      3.257963    0.914944    1.875660
H      4.075437    1.593270    3.322604
C      5.341209    2.573402    1.217577
H      5.175782    3.224937    2.103049
```

H	4.476045	2.719091	0.539095
H	6.259038	2.934915	0.715241
C	6.683209	0.978689	2.629211
H	7.636722	1.177537	2.102393
H	6.753205	-0.018301	3.106864
H	6.599460	1.723788	3.451267
C	6.685623	0.602146	-1.184666
C	8.046881	1.076547	-0.634468
H	8.594482	0.279150	-0.095706
H	7.950023	1.947635	0.042794
H	8.687041	1.399461	-1.485206
C	6.919495	-0.498559	-2.245208
H	7.481601	-0.050629	-3.093480
H	5.964304	-0.889501	-2.651825
H	7.523012	-1.346379	-1.867991
C	5.985576	1.769395	-1.926284
H	6.634177	2.089254	-2.771231
H	5.810527	2.655304	-1.290745
H	5.009389	1.450593	-2.347014
C	1.501594	-0.416895	-1.902523
O	1.526462	-0.688771	-3.095578
O	0.315390	-0.268530	-1.266838

4-Au

E= -3396.5414854

Au	3.240285	-0.030721	-0.308601
P	5.628873	-0.022009	-0.442731
Al	-1.081629	-0.004299	0.121502
O	-2.786401	0.006578	-0.960645
O	0.615331	-0.011888	1.039356
O	0.374985	-0.029743	-1.130633
N	-1.622181	1.818225	0.398377
N	-1.660377	-1.811824	0.426377
C	-3.531372	1.193813	-0.868812
C	-2.858927	2.191141	-0.131247
C	-3.549071	3.420679	-0.008896
H	-3.060129	4.241741	0.536048
C	-4.854067	3.586324	-0.540037
C	-5.482516	2.506357	-1.203124
H	-6.501858	2.621044	-1.590790
C	-4.821613	1.266242	-1.379036
C	-5.390109	0.022896	-2.102883
C	-4.836191	-1.230888	-1.386195
C	-5.507270	-2.461046	-1.211968
H	-6.528921	-2.571627	-1.601959
C	-4.891467	-3.552175	-0.546571
C	-3.593070	-3.400726	-0.006743
H	-3.112192	-4.220639	0.542139
C	-2.890954	-2.172809	-0.124999
C	-3.543937	-1.173316	-0.871341
C	-5.674111	-4.882832	-0.412173
C	-6.991763	-4.630883	0.366149
H	-7.643380	-3.894822	-0.147487
H	-6.783174	-4.241378	1.384394
H	-7.569185	-5.574706	0.470869
C	-6.006800	-5.428101	-1.825630
H	-5.080030	-5.622243	-2.404937
H	-6.623931	-4.715000	-2.409549
H	-6.572446	-6.382092	-1.753072
C	-4.871001	-5.964069	0.342132
H	-5.467443	-6.898132	0.407637
H	-4.626355	-5.652865	1.378599
H	-3.920791	-6.211857	-0.174417
C	-6.929911	0.031344	-2.112910
H	-7.347142	0.029840	-1.085701
H	-7.324347	-0.853711	-2.651508
H	-7.314773	0.924480	-2.644990
C	-4.870793	0.024424	-3.572057
H	-5.225243	0.930546	-4.106561
H	-5.237279	-0.873156	-4.112837
H	-3.762908	0.017150	-3.607662
C	-5.564644	4.951128	-0.355548
C	-5.717877	5.254855	1.157579
H	-6.227454	6.230690	1.310076
H	-4.736232	5.306218	1.670815
H	-6.320577	4.470495	1.661310
C	-4.719975	6.068544	-1.020403
H	-4.595927	5.880332	-2.107357
H	-3.707602	6.142874	-0.573937
H	-5.213936	7.056469	-0.897373
C	-6.971160	4.971437	-0.991982
H	-6.934526	4.784647	-2.085405
H	-7.437928	5.967159	-0.840305
H	-7.644969	4.217101	-0.535239
C	-0.794426	2.869893	0.922816
C	-0.045114	3.692945	0.023518
C	0.723561	4.750556	0.553070
H	1.296177	5.394964	-0.134253
C	0.774643	4.998310	1.931012
H	1.377080	5.833289	2.324257
C	0.057836	4.171917	2.806390
H	0.106603	4.363829	3.890130
C	-0.729886	3.103322	2.329484
C	-1.515454	2.246711	3.322726
H	-1.803308	1.317312	2.786632
C	-0.674714	1.839990	4.547523
H	0.260942	1.328780	4.242950
H	-1.248727	1.148509	5.198315
H	-0.399397	2.715726	5.173285
C	-2.823294	2.945175	3.756794
H	-2.607349	3.899052	4.285333
H	-3.405817	2.299485	4.448376
H	-3.468086	3.177740	2.885549
C	-0.054817	3.473090	-1.491915
H	-0.603957	2.529818	-1.684251
C	1.367498	3.291827	-2.062047
H	1.975868	4.216001	-1.955678
H	1.320644	3.049168	-3.144813
H	1.904601	2.463865	-1.553966
C	-0.797027	4.608305	-2.230683
H	-1.840149	4.712147	-1.870716
H	-0.832787	4.407065	-3.322735
H	-0.286474	5.585275	-2.086621
C	-0.857123	-2.865162	0.984466
C	-0.117292	-3.726206	0.114552
C	0.632791	-4.779172	0.679803
H	1.198212	-5.453194	0.015336
C	0.676298	-4.982530	2.064438
H	1.266212	-5.812250	2.486692
C	-0.030913	-4.118201	2.911643
H	0.015243	-4.277060	3.999983
C	-0.803556	-3.055451	2.399459
C	-1.590786	-2.154853	3.353825
H	-1.691091	-1.167880	2.852172
C	-0.878226	-1.923706	4.698570
H	-0.831270	-2.850307	5.309846
H	-1.430859	-1.173727	5.301043
H	0.157531	-1.553757	4.556253
C	-3.021015	-2.692640	3.586658
H	-3.580398	-2.797252	2.635980
H	-3.596184	-2.009571	4.247833
H	-2.989040	-3.690763	4.075001
C	-0.114173	-3.552147	-1.407057
H	-0.656263	-2.612285	-1.633159
C	-0.855862	-4.705717	-2.117791
H	-0.351690	-5.680430	-1.940233
H	-0.881559	-4.537158	-3.215618
H	-1.902749	-4.792764	-1.763759
C	1.314207	-3.396453	-1.970099
H	1.851019	-2.554974	-1.484840
H	1.277927	-3.189919	-3.060816
H	1.916897	-4.319266	-1.826909
C	1.200411	-0.025783	-0.119985
C	6.188125	-0.467829	-2.253036
C	5.956046	-1.977693	-2.493830
H	4.916080	-2.282035	-2.257230
H	6.655561	-2.622429	-1.927898
H	6.122697	-2.185692	-3.573063
C	5.249676	0.276502	-3.236680
H	5.478539	-0.066763	-4.269214
H	5.373053	1.373593	-3.219309
H	4.183357	0.048638	-3.030192
C	7.659020	-0.124943	-2.571025
H	8.374692	-0.636568	-1.898476
H	7.861745	0.963166	-2.529456

H	7.888058	-0.455371	-3.608067
C	6.315119	-1.339642	0.815292
C	6.128446	-0.802010	2.253136
H	6.803389	0.041238	2.494935
H	6.365421	-1.623377	2.963623
H	5.081168	-0.491530	2.446108
C	5.430550	-2.608068	0.711150
H	5.525209	-3.134726	-0.254561
H	4.357991	-2.370875	0.869326
H	5.741312	-3.318610	1.507872
C	7.796073	-1.716547	0.599787
H	8.102152	-2.434965	1.391783
H	8.476414	-0.845457	0.666350
H	7.969899	-2.218488	-0.372259
C	6.268328	1.754487	0.035896
C	7.768682	1.815495	0.392423
H	8.421502	1.467128	-0.431310
H	8.013945	1.230577	1.300496
H	8.043426	2.871827	0.606291
C	5.984032	2.723950	-1.134919
H	6.203632	3.757292	-0.789055
H	4.919374	2.700916	-1.444808
H	6.620097	2.537250	-2.021384
C	5.424543	2.262522	1.232783
H	5.592931	1.694447	2.164341
H	4.338207	2.236153	1.007665
H	5.703517	3.319744	1.434450

6. References for supporting information

s1. Hicks, J.; Vasko, P.; Goicoechea, J. M.; Aldridge, S. Synthesis, structure and reaction chemistry of a nucleophilic aluminyl anion. *Nature* **2018**, *557*, 92-95.

s2. Goel, R. G. ; Beauchamp A. L., Preparation, characterization, and solution behavior of tri- tert-butylphosphine complexes of copper(I) halides and crystal structure of bromo(tri-tert-butylphosphine)copper(I) tetramer, *Inorg. Chem.* **1983***, 22, 395–400*

s3. Cosier, J.; Glazer, A. M. A nitrogen-gas-stream cryostat for general X-ray diffraction studies. *J. Appl. Cryst.* **1986**, *19*, 105–107.

s4. CrysAlisPro v.1.171.35.8, Agilent Technologies (2011)

s5. Sheldrick, G. M. A short history of *SHELX. Acta Cryst.* **2008**, *A64*, 112–122.

s6. Barbour, L. J. X-Seed - A Software Tool for Supramolecular Crystallography. *J. Supramol. Chem.* **2001**, *1*, 189–191.

s7. Frisch, M. J.; Trucks, G. W.; Schlegel, H. B.; Scuseria, G. E.; Robb, M. A.; Cheeseman, J. R.; Scalmani, G.; Barone, V.; Mennucci, B.; Petersson, G. A.; Nakatsuji, H.; Caricato, M.; Li, X.; Hratchian, H. P.; Izmaylov, A. F.; Bloino, J.; Zheng, G.; Sonnenberg, J. L.; Hada, M.; Ehara, M.; Toyota, K.; Fukuda, R.; Hasegawa, J.; Ishida, M.; Nakajima, T.; Honda, Y.; Kitao, O.; Nakai, H.; Vreven, T.; Montgomery, J. A., Jr.; Peralta, J. E.; Ogliaro, F.; Bearpark, M.; Heyd, J. J.; Brothers, E.; Kudin, K. N.; Staroverov, V. N.; Kobayashi, R.; Normand, J.; Raghavachari, K.; Rendell, A.; Burant, J. C.; Iyengar, S. S.; Tomasi, J.; Cossi, M.; Rega, N.; Millam, J. M.; Klene, M.; Knox, J. E.; Cross, J. B.; Bakken, V.; Adamo, C.; Jaramillo, J.; Gomperts, R.; Stratmann, R. E.; Yazyev, O.; Austin, A. J.; Cammi, R.; Pomelli, C.; Ochterski, J. W.; Martin, R. L.; Morokuma, K.; Zakrzewski, V. G.; Voth, G. A.; Salvador, P.; Dannenberg, J. J.; Dapprich, S.; Daniels, A. D.; Farkas, Ö.; Foresman, J. B.; Ortiz, J. V.; Cioslowski, J.; Fox, D. J., Gaussian, Inc. , Wallingford, CT, **2009**.

s8. Becke, A. D. *Phys. Rev. A*. **1988**, *38*, 3098 – 3100.

s9. Perdew, J. P. *Phys. Rev. B*. **1986**, *33*, 8822 – 8824.

s10. Zhao, Y.; Truhlar, D. G. *Theor. Chem. Acc.* **2008**, *120*, 215 – 241.

s11. Weigend, F. *Phys. Chem. Chem. Phys.* **2006**, *8*, 1057 – 1065.

s12. Weigend, F.; Ahlrichs, R. *Phys. Chem. Chem. Phys.* **2005**, *7*, 3297 – 3305.

s13. Weigend, F.; Häser, M.; Patzelt, H.; Ahlrichs, R. *Chem. Phys. Lett.* **1998**, *294*, 143-152.

s14. Grimme, S.; Ehrlich, S.; Goerigk, L. *J. Comput. Chem.* **2011**, *32*, 1456-1465.

s15. Grimme, S.; Antony, J.; Ehrlich, S.; Krieg, H. *J. Chem. Phys.* **2010**, *132*, 154104.

s16. Marenich, A. V. ; Cramer, C. J. ; Truhlar, D. G. *J. Phys. Chem. B*. **2009**, *113*, 6378 – 6396.

s17. Fukui, K. *Acc. Chem. Res.* **1981**, *14*, 363 – 368.

s18. Legault, C. Y. CYLview, 1.0b; Université de Sherbrooke, Sherbrooke (Québec) Canada, **2009**, http://www.cylview.org.
